# Supplementary material for: Adolescent anxiety and pain problems: A joint, genome-wide investigation and pathway-based analysis
Source: PLoS One. 2023 May 5;18(5):e0285263. doi: 10.1371/journal.pone.0285263 (PMC10162554; doi:10.1371/journal.pone.0285263)
Supplement: S1 Table — (DOCX) [file pone.0285263.s001.docx]

| **S1 Table. Descriptive statistics of the phenotype's distribution in the QNTS and QLSCD cohorts.** | | | | | | | | |
| --- | --- | --- | --- | --- | --- | --- | --- | --- |
|  | **N** | **Min** | **Max** | **Mean(±SD)** | **Skewness** | **Kurtosis** | **Shapiro-Wilk test** | |
|  |  |  |  |  |  |  | **W** | ***p*** |
| **QNTS_Mean Pain** | 352 | 0.000 | 2.000 | 0.601(±0.409) | 0.990 | 0.994 | 0.936 | <0.001 |
| **QNTS_Mean Anxiety** |  | 1.000 | 3.571 | 1.629(±0.432) | 1.029 | 1.026 | 0.928 | <0.001 |
| **QLSCD_Mean Pain** | 754 | 0.167 | 1.278 | 0.546(±0.190) | 0.415 | 0.076 | .0979 | <0.001 |
| **QLSCD_Mean Anxiety** |  | 1.048 | 2.905 | 1.604(±0.414) | 0.748 | 0.076 | 0.936 | <0.001 |
