# Supplementary material for: Adolescent anxiety and pain problems: A joint, genome-wide investigation and pathway-based analysis
Source: PLoS One. 2023 May 5;18(5):e0285263. doi: 10.1371/journal.pone.0285263 (PMC10162554; doi:10.1371/journal.pone.0285263)
Supplement: S2 Table — (DOCX) [file pone.0285263.s002.docx]

| **S2a Table. Top associations (*p*<1x10^-5^) of the genome-wide analysis** | | | | | | | |
| --- | --- | --- | --- | --- | --- | --- | --- |
| **QNTS_Mean Pain** | | | | | | | |
| **Chromosome** | **SNP** | **Position (hg37)** | **Gene** | **Allele** | **Nr. of subjects** | **β** | **p-value** |
| 2 | rs2944782 | 47896974 | Intergenic | G | 348 | 0,227 | 1,83E-06 |
| 2 | rs2705775 | 47897866 | Intergenic | T | 352 | 0,215 | 5,90E-06 |
| 3 | rs17026243 | 30725592 | *TGFBR2* | T | 345 | 0,528 | 5,77E-06 |
| 5 | rs28569910 | 25894634 | Intergenic | G | 347 | 0,144 | 9,04E-06 |
| 7 | rs995384 | 135939080 | *LOC105375523* | G | 352 | -0,130 | 6,81E-06 |
| 7 | rs7790457 | 135939285 | *LOC105375523* | G | 352 | -0,130 | 6,81E-06 |
| 15 | rs138911338 | 84245819 | *SH3GL3* | A | 349 | 0,604 | 7,21E-06 |
| 16 | rs8060756 | 63202385 | *LOC105371308* | A | 348 | 0,137 | 9,21E-06 |
| 18 | rs2445454 | 48840753 | Intergenic | A | 348 | 0,179 | 6,89E-07 |
| 18 | rs2955469 | 48875684 | Intergenic | A | 345 | 0,170 | 2,01E-06 |
| 18 | rs2445473 | 48864058 | Intergenic | A | 351 | 0,165 | 3,18E-06 |
| 18 | rs2442959 | 48844773 | Intergenic | C | 352 | 0,160 | 5,88E-06 |
| 18 | rs35124355 | 48845256 | Intergenic | T | 352 | 0,160 | 5,88E-06 |
| 18 | rs2442955 | 48853128 | Intergenic | A | 352 | 0,160 | 5,88E-06 |
| 18 | rs2445438 | 48832884 | Intergenic | C | 350 | 0,160 | 5,95E-06 |
| 18 | rs2445448 | 48834364 | Intergenic | T | 350 | 0,16’ | 5,95E-06 |
| 18 | rs2262581 | 48835157 | Intergenic | T | 350 | 0,16’ | 5,95E-06 |
| 18 | rs2445471 | 48831360 | Intergenic | T | 348 | 0,157 | 8,31E-06 |
| 20 | rs73094118 | 13703980 | *ESF1* | T | 348 | 0,217 | 4,88E-06 |
| 20 | rs73094125 | 13706875 | *ESF1* | T | 348 | 0,217 | 4,88E-06 |
| 20 | rs56024260 | 13708796 | *ESF1* | G | 348 | 0,217 | 4,88E-06 |
| 20 | rs6079161 | 13731813 | *ESF1* | G | 348 | 0,217 | 4,88E-06 |
| 20 | rs7353586 | 13732220 | *ESF1* | G | 348 | 0,217 | 4,88E-06 |
| 20 | rs6079160 | 13730556 | *ESF1* | T | 346 | 0,221 | 6,83E-06 |
| 20 | rs6079164 | 13738762 | *ESF1* | A | 346 | 0,221 | 6,83E-06 |
| 20 | rs6079146 | 13695498 | *ESF1* | A | 351 | 0,210 | 7,33E-06 |
| 20 | rs34414644 | 13695607 | *ESF1* | G | 351 | 0,210 | 7,33E-06 |
| 20 | rs11696371 | 13696439 | *ESF1* | T | 351 | 0,210 | 7,33E-06 |
| 20 | rs34175600 | 13696931 | *ESF1* | A | 351 | 0,210 | 7,33E-06 |
| 20 | rs112225594 | 13697532 | *ESF1* | A | 351 | 0,210 | 7,33E-06 |
| 20 | rs6079147 | 13697810 | *ESF1* | A | 351 | 0,210 | 7,33E-06 |
| 20 | rs6079148 | 13697811 | *ESF1* | A | 351 | 0,210 | 7,33E-06 |
| 20 | rs2031934 | 13698885 | *ESF1* | A | 351 | 0,210 | 7,33E-06 |
| 20 | rs112181934 | 13690328 | Intergenic | A | 351 | 0,210 | 8,05E-06 |

| **S2b Table. Top associations (*p*<1x10^-5^) of the genome-wide analysis** | | | | | | | |
| --- | --- | --- | --- | --- | --- | --- | --- |
| **QNTS_Mean Anxiety** | | | | | | | |
| **Chromosome** | **SNP** | **Position (hg37)** | **Gene** | **Allele** | **Nr. of subjects** | **β** | **p-value** |
| 1 | rs404100 | 25366987 | *LINC02793* | T | 352 | -0,148 | 4,81E-06 |
| 2 | rs115571376 | 52977127 | *LOC105369165* | T | 351 | 0,459 | 6,15E-06 |
| 2 | rs114695627 | 123164375 | *LOC105373592* | G | 345 | 0,463 | 8,74E-06 |
| 6 | rs74316489 | 92825891 | Intergenic | T | 347 | 0,221 | 8,85E-06 |
| 6 | rs74369993 | 92818071 | Intergenic | A | 346 | 0,220 | 9,52E-06 |
| 10 | rs146244003 | 85534104 | Intergenic | G | 348 | 1,095 | 3,83E-06 |
| 10 | rs187390348 | 85607850 | Intergenic | A | 348 | 0,935 | 5,27E-06 |
| 12 | rs12321232 | 9315668 | *PZP/KLRG1* | T | 352 | 0,166 | 5,73E-06 |
| 12 | rs7311982 | 9314857 | *PZP/KLRG1* | T | 352 | 0,156 | 9,59E-06 |
| 14 | rs10132268 | 35947614 | *LOC105370450/LOC107984681* | T | 348 | 0,145 | 8,90E-06 |
| 14 | rs62003651 | 58015285 | Intergenic | G | 350 | -0,183 | 9,48E-06 |
| 14 | rs62003652 | 58018176 | Intergenic | C | 350 | -0,183 | 9,48E-06 |
| 14 | rs62003655 | 58019814 | Intergenic | C | 350 | -0,183 | 9,48E-06 |
| 17 | rs117780285 | 76999194 | *CANT1* | A | 352 | 0,462 | 7,91E-06 |
| 19 | rs73039354 | 35922031 | *LOC101927522* | G | 350 | 0,422 | 1,46E-06 |
| 19 | rs73039344 | 35921507 | *LOC101927522* | A | 352 | 0,415 | 1,80E-06 |
| 19 | rs77275017 | 35917093 | *LOC101927522* | T | 349 | 0,424 | 1,88E-06 |
| 19 | rs56405407 | 35918454 | *LOC101927522* | A | 350 | 0,422 | 1,95E-06 |
| 19 | rs143749497 | 35904024 | *LINC01531* | T | 347 | 0,424 | 2,21E-06 |
| 19 | rs12975617 | 46208023 | Intergenic | T | 352 | 0,142 | 5,80E-06 |
| 19 | rs34568773 | 46208235 | Intergenic | G | 350 | 0,142 | 6,16E-06 |
| 19 | rs6509233 | 46208669 | Intergenic | A | 350 | 0,142 | 7,37E-06 |
| 19 | rs4806156 | 35921429 | *LOC101927522* | C | 352 | 0,302 | 8,41E-06 |
| 19 | rs8103139 | 35921441 | *LOC101927522* | A | 352 | 0,302 | 8,41E-06 |
| 19 | rs8107905 | 35921697 | *LOC101927522* | G | 352 | 0,302 | 8,41E-06 |
| 19 | rs56352245 | 35918542 | *LOC101927522* | G | 350 | 0,304 | 9,59E-06 |
| 19 | rs55751627 | 35918610 | *LOC101927522* | G | 350 | 0,304 | 9,59E-06 |
| 19 | rs61665846 | 35919187 | *LOC101927522* | C | 350 | 0,304 | 9,85E-06 |
| 22 | rs117132518 | 44069804 | *EFCAB6* | A | 348 | 0,796 | 3,80E-06 |
| 22 | rs137809 | 44092800 | *EFCAB6* | A | 348 | 0,796 | 3,80E-06 |
| 22 | rs73174361 | 44143638 | *EFCAB6* | T | 347 | 0,796 | 3,82E-06 |
