# Supplementary material for: Adolescent anxiety and pain problems: A joint, genome-wide investigation and pathway-based analysis
Source: PLoS One. 2023 May 5;18(5):e0285263. doi: 10.1371/journal.pone.0285263 (PMC10162554; doi:10.1371/journal.pone.0285263)
Supplement: S3 Table — (DOCX) [file pone.0285263.s003.docx]

| **S3a Table 3. Results of the pathway-based analysis of QNTS_Mean Pain and QNTS_Mean Anxiety (uncorrected *p-value* < 0.05).** | | | | | | | |
| --- | --- | --- | --- | --- | --- | --- | --- |
| **QNTS_Mean Pain** | | | | | | | |
| **GO set ID** | **Description** | **Pathway size (nr of genes)** | **Status** | **Significant Genes (nr of)** | **Genes** | ***p-value*** | **FDR** |
| GO:0031424 | keratinization | 181 | enriched | 20 | *KRT39, KRT40, KRT82, KRTAP1-1, KRTAP1-3, KRTAP1-4, KRTAP1-5, KRTAP2-1, KRTAP2-2, KRTAP2-3, KRTAP2-4, KRTAP3-1, KRTAP3-2, KRTAP3-3, KRTAP4-11, KRTAP4-5, KRTAP4-6, KRTAP4-7, KRTAP4-8, KRTAP4-9* | <0.0001 | <0.0001 |
| GO:0042633 | hair cycle | 15 | enriched | 5 | *KRTAP4-5, KRTAP4-7, KRTAP4-8, KRTAP4-9, PTGS2* | <0.0001 | 0.0001 |
| GO:0070988 | demethylation | 4 | enriched | 2 | *CYP1A1, CYP51A1* | <0.0001 | 0.0127 |
| GO:0002480 | antigen processing and presentation of exogenous peptide antigen via MHC class I, TAP-independent | 8 | enriched | 2 | *HLA-A, HLA-G* | <0.0001 | 0.0475 |
| GO:0010389 | regulation of G2/M transition of mitotic cell cycle | 80 | enriched | 6 | *DYNLL1, PPP2R1A, AKAP9, CEP192, CNTRL, KCNH5* | <0.0001 | 0.0475 |
| GO:0097711 | ciliary basal body-plasma membrane docking | 95 | enriched | 6 | *DYNLL1, PPP2R1A, AKAP9, CCDC41, CEP192, CNTRL* | <0.0001 | 0.0475 |
| GO:0002476 | antigen processing and presentation of endogenous peptide antigen via MHC class Ib | 11 | enriched | 2 | *HLA-A, HLA-G* | <0.0001 | 0.0528 |
| GO:0002486 | antigen processing and presentation of endogenous peptide antigen via MHC class I via ER pathway, TAP-independent | 11 | enriched | 2 | *HLA-A, HLA-G* | <0.0001 | 0.0528 |
| GO:0007568 | aging | 170 | enriched | 13 | *APOD, CTNNA1, CTSC, CYP1A1, KRTAP4-5, KRTAP4-7, KRTAP4-8, KRTAP4-9, MAPK1, NPY2R, PTGS2, TGFBR2, VCAM1* | <0.0001 | 0.0528 |
| GO:0010518 | positive regulation of phospholipase activity | 8 | enriched | 2 | *CYR61, PLA2G5* | <0.0001 | 0.0528 |
| GO:0051149 | positive regulation of muscle cell differentiation | 25 | enriched | 3 | *CTNNA1, MAPK11, MAPK12* | <0.0001 | 0.0528 |
| GO:0032543 | mitochondrial translation | 31 | enriched | 3 | *GATC, IARS2, NOA1* | <0.0001 | 0.0730 |
| GO:0019370 | leukotriene biosynthetic process | 10 | enriched | 2 | *ALOX5, PLA2G5* | 0.0001 | 0.1176 |
| GO:0030330 | DNA damage response, signal transduction by p53 class mediator | 16 | enriched | 2 | *MYO6, TRIAP1* | 0.0001 | 0.1259 |
| GO:0042376 | phylloquinone catabolic process | 3 | enriched | 1 | *CBR3* | 0.0001 | 0.1259 |
| GO:0001916 | positive regulation of T cell mediated cytotoxicity | 27 | enriched | 3 | *FAM49B, HLA-A, HLA-G* | 0.0001 | 0.1431 |
| GO:2000353 | positive regulation of endothelial cell apoptotic process | 18 | enriched | 3 | *PRKCI, HLA-G, THBS1* | 0.0002 | 0.1557 |
| GO:0010596 | negative regulation of endothelial cell migration | 22 | enriched | 3 | *KRIT1, SP100, THBS1* | 0.0002 | 0.1663 |
| GO:1900087 | positive regulation of G1/S transition of mitotic cell cycle | 32 | enriched | 3 | *RPTOR, CYP1A1, KMT2E* | 0.0002 | 0.1663 |
| GO:0002666 | positive regulation of T cell tolerance induction | 4 | enriched | 2 | *HLA-G, TGFBR2* | 0.0003 | 0.1724 |
| GO:0010754 | negative regulation of cGMP-mediated signaling | 6 | enriched | 3 | *PDE11A, PDE2A, THBS1* | 0.0002 | 0.1724 |
| GO:0042759 | long-chain fatty acid biosynthetic process | 22 | enriched | 3 | *ALOX5, CYP1A1, PTGS2* | 0.0003 | 0.1724 |
| GO:0034198 | cellular response to amino acid starvation | 40 | enriched | 4 | *C7orf60, GCN1L1, MAPK1, SEH1L* | 0.0004 | 0.2136 |
| GO:0060155 | platelet dense granule organization | 8 | enriched | 2 | *DTNBP1, RAB38* | 0.0004 | 0.2136 |
| GO:0000086 | G2/M transition of mitotic cell cycle | 130 | enriched | 7 | *DYNLL1, PPP2R1A, AKAP9, CCNY, CEP192, CNTRL, PPP1R12B* | 0.0004 | 0.2284 |
| GO:0060044 | negative regulation of cardiac muscle cell proliferation | 13 | enriched | 3 | *KCNK2, MAPK11, TGFBR2* | 0.0005 | 0.2375 |
| GO:0006428 | isoleucyl-tRNA aminoacylation | 2 | enriched | 1 | *IARS2* | 0.0005 | 0.2386 |
| GO:0010748 | negative regulation of long-chain fatty acid import across plasma membrane | 4 | enriched | 1 | *THBS1* | 0.0006 | 0.2503 |
| GO:0051046 | regulation of secretion | 2 | enriched | 1 | *MYO6* | 0.0006 | 0.2503 |
| GO:0070681 | glutaminyl-tRNAGln biosynthesis via transamidation | 3 | enriched | 1 | *GATC* | 0.0006 | 0.2503 |
| GO:1905437 | positive regulation of histone H3-K4 trimethylation | 2 | enriched | 1 | *KMT2E* | 0.0006 | 0.2503 |
| GO:0002495 | antigen processing and presentation of peptide antigen via MHC class II | 2 | enriched | 1 | *marzo8* | 0.0011 | 0.3168 |
| GO:0002544 | chronic inflammatory response | 8 | enriched | 2 | *THBS1, VCAM1* | 0.0012 | 0.3168 |
| GO:0002767 | immune response-inhibiting cell surface receptor signaling pathway | 3 | enriched | 1 | *HLA-G* | 0.0011 | 0.3168 |
| GO:0010751 | negative regulation of nitric oxide mediated signal transduction | 3 | enriched | 1 | *THBS1* | 0.0008 | 0.3168 |
| GO:0042461 | photoreceptor cell development | 4 | enriched | 1 | *ARL3* | 0.0010 | 0.3168 |
| GO:0043506 | regulation of JUN kinase activity | 2 | enriched | 1 | *DTNBP1* | 0.0011 | 0.3168 |
| GO:0050808 | synapse organization | 46 | enriched | 4 | *GRM5, CTNND2, LRRTM2, SNCB* | 0.0012 | 0.3168 |
| GO:0051823 | regulation of synapse structural plasticity | 5 | enriched | 2 | *CAMK2B, DMPK* | 0.0010 | 0.3168 |
| GO:0061470 | T follicular helper cell differentiation | 4 | enriched | 1 | *RC3H2* | 0.0009 | 0.3168 |
| GO:0071539 | protein localization to centrosome | 19 | enriched | 2 | *CCDC41, CEP192* | 0.0012 | 0.3168 |
| GO:0090169 | regulation of spindle assembly | 2 | enriched | 1 | *SENP6* | 0.0010 | 0.3168 |
| GO:0090222 | centrosome-templated microtubule nucleation | 2 | enriched | 1 | *CEP192* | 0.0011 | 0.3168 |
| GO:0090234 | regulation of kinetochore assembly | 3 | enriched | 1 | *SENP6* | 0.0011 | 0.3168 |
| GO:1903588 | negative regulation of blood vessel endothelial cell proliferation involved in sprouting angiogenesis | 4 | enriched | 1 | *THBS1* | 0.0008 | 0.3168 |
| GO:1905799 | regulation of intraciliary retrograde transport | 2 | enriched | 1 | *TCTEX1D2* | 0.0009 | 0.3168 |
| GO:2000628 | regulation of miRNA metabolic process | 4 | enriched | 1 | *RC3H2* | 0.0010 | 0.3168 |
| GO:2001236 | regulation of extrinsic apoptotic signaling pathway | 4 | enriched | 1 | *TRAF1* | 0.0011 | 0.3168 |
| GO:2001241 | positive regulation of extrinsic apoptotic signaling pathway in absence of ligand | 11 | enriched | 2 | *PPP2R1A, CTNNA1* | 0.0012 | 0.3168 |
| GO:0001913 | T cell mediated cytotoxicity | 11 | enriched | 2 | *CTSC, HLA-A* | 0.0013 | 0.3316 |
| GO:0060841 | venous blood vessel development | 4 | enriched | 1 | *ACVR2B* | 0.0013 | 0.3316 |
| GO:0009308 | amine metabolic process | 8 | enriched | 2 | *CYP1A1, VCAM1* | 0.0015 | 0.3504 |
| GO:0010041 | response to iron(III) ion | 3 | enriched | 1 | *CYP1A1* | 0.0015 | 0.3504 |
| GO:0035063 | nuclear speck organization | 4 | enriched | 1 | *SRPK2* | 0.0015 | 0.3504 |
| GO:0002605 | negative regulation of dendritic cell antigen processing and presentation | 4 | enriched | 1 | *THBS1* | 0.0018 | 0.3522 |
| GO:0002645 | positive regulation of tolerance induction | 2 | enriched | 1 | *HLA-G* | 0.0020 | 0.3522 |
| GO:0006448 | regulation of translational elongation | 2 | enriched | 1 | *GRM5* | 0.0019 | 0.3522 |
| GO:0007206 | phospholipase C-activating G protein-coupled glutamate receptor signaling pathway | 2 | enriched | 1 | *GRM5* | 0.0019 | 0.3522 |
| GO:0010830 | regulation of myotube differentiation | 5 | enriched | 1 | *DMPK* | 0.0017 | 0.3522 |
| GO:0038018 | Wnt receptor catabolic process | 2 | enriched | 1 | *ZNRF3* | 0.0017 | 0.3522 |
| GO:0051315 | attachment of mitotic spindle microtubules to kinetochore | 10 | enriched | 2 | *CENPE, SEH1L* | 0.0017 | 0.3522 |
| GO:0070940 | dephosphorylation of RNA polymerase II C-terminal domain | 5 | enriched | 1 | *RPRD1A* | 0.0018 | 0.3522 |
| GO:0090327 | negative regulation of locomotion involved in locomotory behavior | 2 | enriched | 1 | *ARRDC3* | 0.0016 | 0.3522 |
| GO:0097051 | establishment of protein localization to endoplasmic reticulum membrane | 3 | enriched | 1 | *RAB3GAP2* | 0.0019 | 0.3522 |
| GO:1902938 | regulation of intracellular calcium activated chloride channel activity | 2 | enriched | 1 | *GRM5* | 0.0020 | 0.3522 |
| GO:1903061 | positive regulation of protein lipidation | 4 | enriched | 1 | *RAB3GAP2* | 0.0020 | 0.3522 |
| GO:1903373 | positive regulation of endoplasmic reticulum tubular network organization | 4 | enriched | 1 | *RAB3GAP2* | 0.0020 | 0.3522 |
| GO:1903441 | protein localization to ciliary membrane | 5 | enriched | 1 | *ARL3* | 0.0020 | 0.3522 |
| GO:2000582 | positive regulation of ATP-dependent microtubule motor activity, plus-end-directed | 5 | enriched | 1 | *DYNLL1* | 0.0019 | 0.3522 |
| GO:2001045 | negative regulation of integrin-mediated signaling pathway | 2 | enriched | 1 | *CTNNA1* | 0.0016 | 0.3522 |
| GO:2001140 | positive regulation of phospholipid transport | 4 | enriched | 1 | *TRIAP1* | 0.0019 | 0.3522 |
| GO:2001184 | positive regulation of interleukin-12 secretion | 4 | enriched | 1 | *MAPK11* | 0.0018 | 0.3522 |
| GO:0032914 | positive regulation of transforming growth factor beta1 production | 6 | enriched | 1 | *THBS1* | 0.0021 | 0.3526 |
| GO:0036037 | CD8-positive, alpha-beta T cell activation | 3 | enriched | 1 | *HLA-A* | 0.0021 | 0.3532 |
| GO:0043154 | negative regulation of cysteine-type endopeptidase activity involved in apoptotic process | 71 | enriched | 5 | *TRIAP1, BIRC7, PTGS2, THBS1, TNFAIP8* | 0.0022 | 0.3532 |
| GO:0060159 | regulation of dopamine receptor signaling pathway | 4 | enriched | 1 | *DTNBP1* | 0.0022 | 0.3532 |
| GO:0042904 | 9-cis-retinoic acid biosynthetic process | 4 | enriched | 1 | *CYP1A1* | 0.0022 | 0.3552 |
| GO:0010626 | negative regulation of Schwann cell proliferation | 5 | enriched | 1 | *RNF10* | 0.0023 | 0.3594 |
| GO:0061084 | negative regulation of protein refolding | 4 | enriched | 1 | *PDCL* | 0.0023 | 0.3594 |
| GO:1903671 | negative regulation of sprouting angiogenesis | 9 | enriched | 2 | *SEMA6A, THBS1* | 0.0023 | 0.3622 |
| GO:1902723 | negative regulation of skeletal muscle satellite cell proliferation | 4 | enriched | 1 | *SIX5* | 0.0024 | 0.3724 |
| GO:0010757 | negative regulation of plasminogen activation | 6 | enriched | 1 | *THBS1* | 0.0025 | 0.3826 |
| GO:0017143 | insecticide metabolic process | 2 | enriched | 1 | *CYP1A1* | 0.0027 | 0.4032 |
| GO:0006778 | porphyrin-containing compound metabolic process | 2 | enriched | 1 | *CYP1A1* | 0.0027 | 0.4039 |
| GO:0002474 | antigen processing and presentation of peptide antigen via MHC class I | 28 | enriched | 2 | *HLA-A, HLA-G* | 0.0028 | 0.4117 |
| GO:0060591 | chondroblast differentiation | 5 | enriched | 1 | *CYR61* | 0.0029 | 0.4174 |
| GO:0001915 | negative regulation of T cell mediated cytotoxicity | 6 | enriched | 1 | *HLA-G* | 0.0030 | 0.4206 |
| GO:0006450 | regulation of translational fidelity | 6 | enriched | 1 | *GATC* | 0.0030 | 0.4206 |
| GO:0048254 | snoRNA localization | 6 | enriched | 1 | *ZNHIT6* | 0.0031 | 0.4206 |
| GO:0051661 | maintenance of centrosome location | 6 | enriched | 1 | *AKAP9* | 0.0031 | 0.4206 |
| GO:1901018 | positive regulation of potassium ion transmembrane transporter activity | 6 | enriched | 2 | *AKAP9, ANK2* | 0.0030 | 0.4206 |
| GO:0031087 | deadenylation-independent decapping of nuclear-transcribed mRNA | 5 | enriched | 1 | *EDC3* | 0.0032 | 0.4216 |
| GO:2001301 | lipoxin biosynthetic process | 5 | enriched | 1 | *ALOX5* | 0.0032 | 0.4216 |
| GO:0099553 | trans-synaptic signaling by endocannabinoid, modulating synaptic transmission | 3 | enriched | 1 | *GRM5* | 0.0034 | 0.4451 |
| GO:0045860 | positive regulation of protein kinase activity | 52 | enriched | 4 | *CENPE, CACUL1, CYR61, TAB2* | 0.0035 | 0.4492 |
| GO:0060294 | cilium movement involved in cell motility | 10 | enriched | 2 | *RSPH6A, TEKT5* | 0.0035 | 0.4492 |
| GO:0060333 | interferon-gamma-mediated signaling pathway | 69 | enriched | 7 | *CAMK2B, HCK, HLA-A, HLA-G, SP100, TRIM8, VCAM1* | 0.0035 | 0.4492 |
| GO:0003278 | apoptotic process involved in heart morphogenesis | 3 | enriched | 1 | *CYR61* | 0.0036 | 0.4493 |
| GO:0002762 | negative regulation of myeloid leukocyte differentiation | 4 | enriched | 3 | *NME1, NME1-NME2, NME2* | 0.0040 | 0.4795 |
| GO:0042590 | antigen processing and presentation of exogenous peptide antigen via MHC class I | 4 | enriched | 1 | *HLA-A* | 0.0039 | 0.4795 |
| GO:0060836 | lymphatic endothelial cell differentiation | 6 | enriched | 1 | *ACVR2B* | 0.0039 | 0.4795 |
| GO:0099151 | regulation of postsynaptic density assembly | 12 | enriched | 3 | *NRXN1, GRID2, LRRTM2* | 0.0041 | 0.4795 |
| GO:1903538 | regulation of meiotic cell cycle process involved in oocyte maturation | 2 | enriched | 1 | *PPP2R1A* | 0.0041 | 0.4795 |
| GO:2001199 | negative regulation of dendritic cell differentiation | 6 | enriched | 1 | *HLA-G* | 0.0040 | 0.4795 |
| GO:0051271 | negative regulation of cellular component movement | 7 | enriched | 2 | *ACTN4, SP100* | 0.0042 | 0.4891 |
| GO:0043279 | response to alkaloid | 6 | enriched | 1 | *SRSF9* | 0.0042 | 0.4906 |
| GO:0106048 | spermidine deacetylation | 2 | enriched | 1 | *HDAC10* | 0.0043 | 0.4906 |
| GO:0002534 | cytokine production involved in inflammatory response | 3 | enriched | 1 | *SEH1L* | 0.0045 | 0.5080 |
| GO:0009624 | response to nematode | 3 | enriched | 1 | *CYP1A1* | 0.0045 | 0.5099 |
| GO:0009812 | flavonoid metabolic process | 5 | enriched | 1 | *CYP1A1* | 0.0048 | 0.5131 |
| GO:0015866 | ADP transport | 6 | enriched | 1 | *SLC25A42* | 0.0048 | 0.5131 |
| GO:0016926 | protein desumoylation | 7 | enriched | 1 | *SENP6* | 0.0047 | 0.5131 |
| GO:0032425 | positive regulation of mismatch repair | 3 | enriched | 1 | *HDAC10* | 0.0046 | 0.5131 |
| GO:0032927 | positive regulation of activin receptor signaling pathway | 7 | enriched | 1 | *ACVR2B* | 0.0048 | 0.5131 |
| GO:0042766 | nucleosome mobilization | 4 | enriched | 1 | *BPTF* | 0.0049 | 0.5198 |
| GO:0071901 | negative regulation of protein serine/threonine kinase activity | 17 | enriched | 2 | *RPTOR, DTNBP1* | 0.0049 | 0.5206 |
| GO:0035655 | interleukin-18-mediated signaling pathway | 7 | enriched | 1 | *ALOX5* | 0.0051 | 0.5317 |
| GO:0001937 | negative regulation of endothelial cell proliferation | 30 | enriched | 2 | *KRIT1, THBS1* | 0.0052 | 0.5353 |
| GO:0002933 | lipid hydroxylation | 7 | enriched | 1 | *CYP1A1* | 0.0053 | 0.5366 |
| GO:0006694 | steroid biosynthetic process | 28 | enriched | 2 | *CYP1A1, CYP51A1* | 0.0053 | 0.5366 |
| GO:0060840 | artery development | 7 | enriched | 1 | *ACVR2B* | 0.0053 | 0.5366 |
| GO:0010038 | response to metal ion | 13 | enriched | 2 | *GPHN, SNCB* | 0.0054 | 0.5386 |
| GO:0061298 | retina vasculature development in camera-type eye | 7 | enriched | 1 | *ACVR2B* | 0.0054 | 0.5386 |
| GO:2000095 | regulation of Wnt signaling pathway, planar cell polarity pathway | 4 | enriched | 1 | *ZNRF3* | 0.0055 | 0.5417 |
| GO:0060613 | fat pad development | 7 | enriched | 1 | *ARRDC3* | 0.0056 | 0.5513 |
| GO:0010760 | negative regulation of macrophage chemotaxis | 7 | enriched | 1 | *C5* | 0.0060 | 0.5827 |
| GO:0060716 | labyrinthine layer blood vessel development | 21 | enriched | 3 | *CYR61, HEY1, MAPK1* | 0.0062 | 0.5992 |
| GO:0075506 | entry of viral genome into host nucleus through nuclear pore complex via importin | 2 | enriched | 1 | *KPNA2* | 0.0064 | 0.6112 |
| GO:0000492 | box C/D snoRNP assembly | 8 | enriched | 1 | *ZNHIT6* | 0.0066 | 0.6226 |
| GO:0006663 | platelet activating factor biosynthetic process | 4 | enriched | 1 | *PLA2G5* | 0.0069 | 0.6312 |
| GO:0010941 | regulation of cell death | 7 | enriched | 1 | *NOA1* | 0.0068 | 0.6312 |
| GO:0042270 | protection from natural killer cell mediated cytotoxicity | 5 | enriched | 1 | *HLA-G* | 0.0069 | 0.6312 |
| GO:0098909 | regulation of cardiac muscle cell action potential involved in regulation of contraction | 8 | enriched | 1 | *AKAP9* | 0.0067 | 0.6312 |
| GO:2001027 | negative regulation of endothelial cell chemotaxis | 3 | enriched | 1 | *THBS1* | 0.0068 | 0.6312 |
| GO:0060399 | positive regulation of growth hormone receptor signaling pathway | 3 | enriched | 1 | *MBD5* | 0.0071 | 0.6397 |
| GO:0007406 | negative regulation of neuroblast proliferation | 5 | enriched | 1 | *CTNNA1* | 0.0072 | 0.6432 |
| GO:0035265 | organ growth | 8 | enriched | 1 | *ACVR2B* | 0.0072 | 0.6432 |
| GO:0000187 | activation of MAPK activity | 119 | enriched | 7 | *MAPK10, C5, KIT, MAPK1, MAPK11, TAB2, THBS1* | 0.0073 | 0.6493 |
| GO:0072593 | reactive oxygen species metabolic process | 31 | enriched | 2 | *CYR61, NDUFS4* | 0.0075 | 0.6552 |
| GO:0060710 | chorio-allantoic fusion | 7 | enriched | 1 | *CYR61* | 0.0075 | 0.6565 |
| GO:0031124 | mRNA 3'-end processing | 59 | enriched | 3 | *SRSF9, RPRD1A, SYMPK* | 0.0077 | 0.6629 |
| GO:0003181 | atrioventricular valve morphogenesis | 13 | enriched | 2 | *CYR61, TGFBR2* | 0.0079 | 0.6752 |
| GO:0043457 | regulation of cellular respiration | 8 | enriched | 1 | *NOA1* | 0.0080 | 0.6752 |
| GO:0060173 | limb development | 31 | enriched | 3 | *RC3H2, SMOC1, ZNRF3* | 0.0081 | 0.6752 |
| GO:1902952 | positive regulation of dendritic spine maintenance | 4 | enriched | 1 | *ZNF804A* | 0.0080 | 0.6752 |
| GO:1904684 | negative regulation of metalloendopeptidase activity | 2 | enriched | 1 | *TIMP3* | 0.0081 | 0.6752 |
| GO:0006379 | mRNA cleavage | 8 | enriched | 1 | *ZNRD1* | 0.0082 | 0.6782 |
| GO:0071681 | cellular response to indole-3-methanol | 5 | enriched | 1 | *CTNNA1* | 0.0082 | 0.6782 |
| GO:0090502 | RNA phosphodiester bond hydrolysis, endonucleolytic | 58 | enriched | 3 | *POP5, EDC3, EXOG* | 0.0085 | 0.6926 |
| GO:0002729 | positive regulation of natural killer cell cytokine production | 8 | enriched | 1 | *HLA-G* | 0.0087 | 0.7046 |
| GO:1990001 | inhibition of cysteine-type endopeptidase activity involved in apoptotic process | 7 | enriched | 1 | *BIRC7* | 0.0088 | 0.7116 |
| GO:0002446 | neutrophil mediated immunity | 8 | enriched | 1 | *KMT2E* | 0.0091 | 0.7282 |
| GO:0097035 | regulation of membrane lipid distribution | 5 | enriched | 1 | *TRIAP1* | 0.0091 | 0.7282 |
| GO:0043666 | regulation of phosphoprotein phosphatase activity | 41 | enriched | 3 | *PPP2R1A, DMPK, PPP6R2* | 0.0093 | 0.7343 |
| GO:0002028 | regulation of sodium ion transport | 18 | enriched | 2 | *DMPK, NKAIN4* | 0.0094 | 0.7370 |
| GO:0007041 | lysosomal transport | 18 | enriched | 2 | *ARSB, USE1* | 0.0094 | 0.7370 |
| GO:0035308 | negative regulation of protein dephosphorylation | 8 | enriched | 1 | *PPP1R11* | 0.0097 | 0.7384 |
| GO:0051918 | negative regulation of fibrinolysis | 10 | enriched | 1 | *THBS1* | 0.0096 | 0.7384 |
| GO:1900186 | negative regulation of clathrin-dependent endocytosis | 4 | enriched | 1 | *SH3GL3* | 0.0095 | 0.7384 |
| GO:1990542 | mitochondrial transmembrane transport | 8 | enriched | 1 | *SFXN2* | 0.0096 | 0.7384 |
| GO:2000320 | negative regulation of T-helper 17 cell differentiation | 8 | enriched | 1 | *RC3H2* | 0.0098 | 0.7402 |
| GO:1900222 | negative regulation of amyloid-beta clearance | 8 | enriched | 1 | *CYP51A1* | 0.0102 | 0.7689 |
| GO:0006163 | purine nucleotide metabolic process | 7 | enriched | 3 | *NME1, NME1-NME2, NME2* | 0.0108 | 0.7942 |
| GO:0045880 | positive regulation of smoothened signaling pathway | 31 | enriched | 2 | *CTNNA1, PDCL* | 0.0107 | 0.7942 |
| GO:0050709 | negative regulation of protein secretion | 17 | enriched | 2 | *CYP51A1, SERGEF* | 0.0109 | 0.7942 |
| GO:0099606 | microtubule plus-end directed mitotic chromosome migration | 1 | enriched | 1 | *CENPE* | 0.0109 | 0.7942 |
| GO:0099607 | lateral attachment of mitotic spindle microtubules to kinetochore | 1 | enriched | 1 | *CENPE* | 0.0109 | 0.7942 |
| GO:0002091 | negative regulation of receptor internalization | 10 | enriched | 1 | *LRRTM2* | 0.0110 | 0.7967 |
| GO:0009635 | response to herbicide | 7 | enriched | 1 | *CYP1A1* | 0.0111 | 0.7983 |
| GO:0048617 | embryonic foregut morphogenesis | 9 | enriched | 1 | *ACVR2B* | 0.0111 | 0.7983 |
| GO:0051403 | stress-activated MAPK cascade | 27 | enriched | 3 | *MAP3K8, MAPK1, MAPK11* | 0.0115 | 0.8144 |
| GO:0060137 | maternal process involved in parturition | 7 | enriched | 1 | *CYP1A1* | 0.0115 | 0.8144 |
| GO:2000568 | positive regulation of memory T cell activation | 1 | enriched | 1 | *FAM49B* | 0.0119 | 0.8393 |
| GO:0060907 | positive regulation of macrophage cytokine production | 9 | enriched | 1 | *HLA-G* | 0.0120 | 0.8429 |
| GO:0001932 | regulation of protein phosphorylation | 43 | enriched | 3 | *GRM5, NDUFS4, PLXNB2* | 0.0121 | 0.8442 |
| GO:0009804 | coumarin metabolic process | 7 | enriched | 1 | *CYP1A1* | 0.0122 | 0.8442 |
| GO:2000051 | negative regulation of non-canonical Wnt signaling pathway | 4 | enriched | 1 | *ZNRF3* | 0.0123 | 0.8442 |
| GO:0051414 | response to cortisol | 4 | enriched | 1 | *IGFBP7* | 0.0124 | 0.8490 |
| GO:0072001 | renal system development | 2 | enriched | 1 | *TBX18* | 0.0125 | 0.8497 |
| GO:0048549 | positive regulation of pinocytosis | 4 | enriched | 1 | *ACTN4* | 0.0128 | 0.8673 |
| GO:0030048 | actin filament-based movement | 19 | enriched | 2 | *MYO6, MYO9B* | 0.0130 | 0.8752 |
| GO:0001956 | positive regulation of neurotransmitter secretion | 7 | enriched | 1 | *DTNBP1* | 0.0131 | 0.8769 |
| GO:0007020 | microtubule nucleation | 17 | enriched | 2 | *AKAP9, TUBGCP6* | 0.0132 | 0.8769 |
| GO:0099601 | regulation of neurotransmitter receptor activity | 9 | enriched | 1 | *PATE4* | 0.0132 | 0.8769 |
| GO:0060306 | regulation of membrane repolarization | 10 | enriched | 2 | *AKAP9, KCNQ1* | 0.0136 | 0.8950 |
| GO:0007178 | transmembrane receptor protein serine/threonine kinase signaling pathway | 10 | enriched | 1 | *ACVR2B* | 0.0137 | 0.8989 |
| GO:0019372 | lipoxygenase pathway | 16 | enriched | 2 | *ALOX5, PTGS2* | 0.0139 | 0.9027 |
| GO:0051090 | regulation of DNA-binding transcription factor activity | 27 | enriched | 4 | *MAPK10, HCK, MAPK1, MAPK11* | 0.0141 | 0.9155 |
| GO:0016525 | negative regulation of angiogenesis | 84 | enriched | 4 | *HLA-G, KRIT1, SEMA6A, THBS1* | 0.0146 | 0.9331 |
| GO:1903984 | positive regulation of TRAIL-activated apoptotic signaling pathway | 3 | enriched | 1 | *TIMP3* | 0.0145 | 0.9331 |
| GO:0019932 | second-messenger-mediated signaling | 3 | enriched | 1 | *PPP2R1A* | 0.0149 | 0.9462 |
| GO:0032940 | secretion by cell | 10 | enriched | 1 | *USE1* | 0.0149 | 0.9462 |
| GO:0000018 | regulation of DNA recombination | 6 | enriched | 1 | *KPNA2* | 0.0403 | 0.9871 |
| GO:0000463 | maturation of LSU-rRNA from tricistronic rRNA transcript (SSU-rRNA, 5.8S rRNA, LSU-rRNA) | 13 | enriched | 1 | *ZNHIT6* | 0.0309 | 0.9871 |
| GO:0001682 | tRNA 5'-leader removal | 11 | enriched | 1 | *POP5* | 0.0273 | 0.9871 |
| GO:0001946 | lymphangiogenesis | 12 | enriched | 1 | *ACVR2B* | 0.0246 | 0.9871 |
| GO:0002479 | antigen processing and presentation of exogenous peptide antigen via MHC class I, TAP-dependent | 73 | enriched | 4 | *HLA-A, HLA-G, PSMA1, PSMD12* | 0.0166 | 0.9871 |
| GO:0002541 | activation of plasma proteins involved in acute inflammatory response | 1 | enriched | 1 | *F3* | 0.0449 | 0.9871 |
| GO:0005998 | xylulose catabolic process | 1 | enriched | 1 | *XYLB* | 0.0397 | 0.9871 |
| GO:0006139 | nucleobase-containing compound metabolic process | 31 | enriched | 2 | *BPNT1, ZNRD1* | 0.0474 | 0.9871 |
| GO:0006183 | GTP biosynthetic process | 11 | enriched | 3 | *NME1, NME1-NME2, NME2* | 0.0474 | 0.9871 |
| GO:0006220 | pyrimidine nucleotide metabolic process | 9 | enriched | 3 | *NME1, NME1-NME2, NME2* | 0.0279 | 0.9871 |
| GO:0006228 | UTP biosynthetic process | 10 | enriched | 3 | *NME1, NME1-NME2, NME2* | 0.0368 | 0.9871 |
| GO:0006241 | CTP biosynthetic process | 11 | enriched | 3 | *NME1, NME1-NME2, NME2* | 0.0437 | 0.9871 |
| GO:0006417 | regulation of translation | 63 | enriched | 4 | *GRM5, DDX25, EIF4E1B, GCN1L1* | 0.0182 | 0.9871 |
| GO:0006488 | dolichol-linked oligosaccharide biosynthetic process | 14 | enriched | 1 | *ALG10* | 0.0379 | 0.9871 |
| GO:0006691 | leukotriene metabolic process | 15 | enriched | 1 | *ALOX5* | 0.0449 | 0.9871 |
| GO:0006744 | ubiquinone biosynthetic process | 15 | enriched | 1 | *COQ5* | 0.0304 | 0.9871 |
| GO:0006957 | complement activation, alternative pathway | 13 | enriched | 2 | *C5, CFB* | 0.0292 | 0.9871 |
| GO:0006975 | DNA damage induced protein phosphorylation | 8 | enriched | 1 | *MAPK12* | 0.0215 | 0.9871 |
| GO:0006998 | nuclear envelope organization | 17 | enriched | 2 | *DMPK, REEP3* | 0.0429 | 0.9871 |
| GO:0006999 | nuclear pore organization | 7 | enriched | 1 | *SEH1L* | 0.0254 | 0.9871 |
| GO:0007050 | cell cycle arrest | 130 | enriched | 5 | *RPTOR, BRINP3, KMT2E, MAPK12, THBS1* | 0.0162 | 0.9871 |
| GO:0007079 | mitotic chromosome movement towards spindle pole | 4 | enriched | 1 | *CENPE* | 0.0258 | 0.9871 |
| GO:0010033 | response to organic substance | 33 | enriched | 4 | *PPP2R1A, CTSC, TIMP3, XRCC1* | 0.0274 | 0.9871 |
| GO:0010457 | centriole-centriole cohesion | 11 | enriched | 1 | *CNTLN* | 0.0205 | 0.9871 |
| GO:0010468 | regulation of gene expression | 137 | enriched | 8 | *MAPK10, MAPK1, MAPK11, MAPK12, NOV, REST, SRPK2, TGFBR2* | 0.0383 | 0.9871 |
| GO:0010575 | positive regulation of vascular endothelial growth factor production | 28 | enriched | 3 | *RORA, C5, PTGS2* | 0.0415 | 0.9871 |
| GO:0010759 | positive regulation of macrophage chemotaxis | 15 | enriched | 1 | *THBS1* | 0.0456 | 0.9871 |
| GO:0010763 | positive regulation of fibroblast migration | 11 | enriched | 1 | *THBS1* | 0.0167 | 0.9871 |
| GO:0010923 | negative regulation of phosphatase activity | 49 | enriched | 3 | *FARP1, CCDC8, CEP192* | 0.0320 | 0.9871 |
| GO:0015867 | ATP transport | 12 | enriched | 1 | *SLC25A42* | 0.0235 | 0.9871 |
| GO:0015966 | diadenosine tetraphosphate biosynthetic process | 3 | enriched | 1 | *MAPK1* | 0.0442 | 0.9871 |
| GO:0016126 | sterol biosynthetic process | 15 | enriched | 1 | *CYP51A1* | 0.0358 | 0.9871 |
| GO:0016264 | gap junction assembly | 8 | enriched | 1 | *CTNNA1* | 0.0309 | 0.9871 |
| GO:0018106 | peptidyl-histidine phosphorylation | 2 | enriched | 2 | *NME1-NME2, NME2* | 0.0224 | 0.9871 |
| GO:0018315 | molybdenum incorporation into molybdenum-molybdopterin complex | 1 | enriched | 1 | *GPHN* | 0.0468 | 0.9871 |
| GO:0019054 | modulation by virus of host cellular process | 7 | enriched | 1 | *KPNA2* | 0.0444 | 0.9871 |
| GO:0019858 | cytosine metabolic process | 2 | enriched | 1 | *MAPK1* | 0.0261 | 0.9871 |
| GO:0030050 | vesicle transport along actin filament | 5 | enriched | 1 | *ACTN4* | 0.0224 | 0.9871 |
| GO:0030194 | positive regulation of blood coagulation | 14 | enriched | 1 | *THBS1* | 0.0253 | 0.9871 |
| GO:0030501 | positive regulation of bone mineralization | 38 | enriched | 2 | *ACVR2B, CYR61* | 0.0160 | 0.9871 |
| GO:0032008 | positive regulation of TOR signaling | 29 | enriched | 2 | *RPTOR, SEH1L* | 0.0348 | 0.9871 |
| GO:0032011 | ARF protein signal transduction | 1 | enriched | 1 | *MYO9B* | 0.0243 | 0.9871 |
| GO:0032026 | response to magnesium ion | 13 | enriched | 1 | *THBS1* | 0.0301 | 0.9871 |
| GO:0032417 | positive regulation of sodium:proton antiporter activity | 4 | enriched | 1 | *ACTN4* | 0.0191 | 0.9871 |
| GO:0032438 | melanosome organization | 22 | enriched | 2 | *DTNBP1, RAB38* | 0.0161 | 0.9871 |
| GO:0032695 | negative regulation of interleukin-12 production | 12 | enriched | 1 | *THBS1* | 0.0271 | 0.9871 |
| GO:0032924 | activin receptor signaling pathway | 18 | enriched | 2 | *ACVR2B, TGFBR2* | 0.0260 | 0.9871 |
| GO:0033690 | positive regulation of osteoblast proliferation | 10 | enriched | 1 | *CYR61* | 0.0174 | 0.9871 |
| GO:0033962 | cytoplasmic mRNA processing body assembly | 13 | enriched | 1 | *EDC3* | 0.0240 | 0.9871 |
| GO:0034629 | cellular protein-containing complex localization | 9 | enriched | 1 | *SEH1L* | 0.0489 | 0.9871 |
| GO:0034968 | histone lysine methylation | 13 | enriched | 2 | *KMT2E, SMYD3* | 0.0297 | 0.9871 |
| GO:0034983 | peptidyl-lysine deacetylation | 8 | enriched | 1 | *HDAC10* | 0.0220 | 0.9871 |
| GO:0035357 | peroxisome proliferator activated receptor signaling pathway | 7 | enriched | 1 | *ACTN4* | 0.0455 | 0.9871 |
| GO:0035385 | Roundabout signaling pathway | 5 | enriched | 2 | *MYO9B, ROBO1* | 0.0385 | 0.9871 |
| GO:0035825 | homologous recombination | 1 | enriched | 1 | *HDAC10* | 0.0181 | 0.9871 |
| GO:0038202 | TORC1 signaling | 6 | enriched | 1 | *RPTOR* | 0.0196 | 0.9871 |
| GO:0040037 | negative regulation of fibroblast growth factor receptor signaling pathway | 14 | enriched | 1 | *THBS1* | 0.0411 | 0.9871 |
| GO:0042119 | neutrophil activation | 12 | enriched | 1 | *KMT2E* | 0.0269 | 0.9871 |
| GO:0042359 | vitamin D metabolic process | 12 | enriched | 1 | *CYP1A1* | 0.0389 | 0.9871 |
| GO:0042417 | dopamine metabolic process | 13 | enriched | 2 | *COMT, SNCB* | 0.0292 | 0.9871 |
| GO:0042994 | cytoplasmic sequestering of transcription factor | 11 | enriched | 1 | *MXI1* | 0.0394 | 0.9871 |
| GO:0043032 | positive regulation of macrophage activation | 16 | enriched | 1 | *THBS1* | 0.0375 | 0.9871 |
| GO:0043086 | negative regulation of catalytic activity | 52 | enriched | 4 | *PDC, PINLYP, PPP1R12B, SNCB* | 0.0296 | 0.9871 |
| GO:0043297 | apical junction assembly | 8 | enriched | 1 | *CTNNA1* | 0.0249 | 0.9871 |
| GO:0043652 | engulfment of apoptotic cell | 12 | enriched | 1 | *THBS1* | 0.0235 | 0.9871 |
| GO:0044319 | wound healing, spreading of cells | 15 | enriched | 1 | *CYR61* | 0.0446 | 0.9871 |
| GO:0044773 | mitotic DNA damage checkpoint | 3 | enriched | 1 | *STK33* | 0.0408 | 0.9871 |
| GO:0045591 | positive regulation of regulatory T cell differentiation | 10 | enriched | 1 | *HLA-G* | 0.0166 | 0.9871 |
| GO:0045953 | negative regulation of natural killer cell mediated cytotoxicity | 10 | enriched | 1 | *HLA-G* | 0.0188 | 0.9871 |
| GO:0046685 | response to arsenic-containing substance | 13 | enriched | 1 | *CYP1A1* | 0.0441 | 0.9871 |
| GO:0048266 | behavioral response to pain | 11 | enriched | 1 | *THBS1* | 0.0214 | 0.9871 |
| GO:0050434 | positive regulation of viral transcription | 27 | enriched | 3 | *MDFIC, NELFE, POLR2B* | 0.0483 | 0.9871 |
| GO:0050665 | hydrogen peroxide biosynthetic process | 8 | enriched | 1 | *CYP1A1* | 0.0171 | 0.9871 |
| GO:0050684 | regulation of mRNA processing | 13 | enriched | 1 | *SRPK2* | 0.0349 | 0.9871 |
| GO:0050710 | negative regulation of cytokine secretion | 15 | enriched | 1 | *PPP1R11* | 0.0385 | 0.9871 |
| GO:0050777 | negative regulation of immune response | 13 | enriched | 1 | *HLA-G* | 0.0312 | 0.9871 |
| GO:0050790 | regulation of catalytic activity | 49 | enriched | 3 | *COX6A1, KRIT1, SIL1* | 0.0249 | 0.9871 |
| GO:0050890 | cognition | 45 | enriched | 3 | *GRM5, CBR3, DOPEY2* | 0.0384 | 0.9871 |
| GO:0051298 | centrosome duplication | 16 | enriched | 1 | *CEP192* | 0.0493 | 0.9871 |
| GO:0051306 | mitotic sister chromatid separation | 4 | enriched | 1 | *PPP2R1A* | 0.0264 | 0.9871 |
| GO:0051591 | response to cAMP | 45 | enriched | 3 | *NDUFS4, NME1, PLA2G5* | 0.0207 | 0.9871 |
| GO:0051660 | establishment of centrosome localization | 9 | enriched | 1 | *CCDC41* | 0.0351 | 0.9871 |
| GO:0051754 | meiotic sister chromatid cohesion, centromeric | 4 | enriched | 1 | *PPP2R1A* | 0.0235 | 0.9871 |
| GO:0051895 | negative regulation of focal adhesion assembly | 18 | enriched | 2 | *APOD, THBS1* | 0.0497 | 0.9871 |
| GO:0060005 | vestibular reflex | 3 | enriched | 1 | *TMC1* | 0.0331 | 0.9871 |
| GO:0060243 | negative regulation of cell growth involved in contact inhibition | 1 | enriched | 1 | *CDHR2* | 0.0453 | 0.9871 |
| GO:0060271 | cilium assembly | 172 | enriched | 10 | *ABCC4, ARL3, BBS9, CCDC41, HYLS1, IFT43, RAB8B, RSPH6A, TCTEX1D2, TEKT5* | 0.0159 | 0.9871 |
| GO:0060413 | atrial septum morphogenesis | 13 | enriched | 1 | *CYR61* | 0.0309 | 0.9871 |
| GO:0060440 | trachea formation | 7 | enriched | 2 | *MAPK1, TGFBR2* | 0.0157 | 0.9871 |
| GO:0060829 | negative regulation of canonical Wnt signaling pathway involved in neural plate anterior/posterior pattern formation | 1 | enriched | 1 | *TBX18* | 0.0189 | 0.9871 |
| GO:0061308 | cardiac neural crest cell development involved in heart development | 2 | enriched | 1 | *MAPK1* | 0.0278 | 0.9871 |
| GO:0061580 | colon epithelial cell migration | 1 | enriched | 1 | *ARSB* | 0.0245 | 0.9871 |
| GO:0070365 | hepatocyte differentiation | 13 | enriched | 1 | *CYP1A1* | 0.0495 | 0.9871 |
| GO:0071310 | cellular response to organic substance | 18 | enriched | 2 | *MAPK1, TIMP3* | 0.0449 | 0.9871 |
| GO:0071363 | cellular response to growth factor stimulus | 59 | enriched | 3 | *ACVR2B, TGFBR2, THBS1* | 0.0352 | 0.9871 |
| GO:0072089 | stem cell proliferation | 9 | enriched | 1 | *ZNRF3* | 0.0348 | 0.9871 |
| GO:0072579 | glycine receptor clustering | 1 | enriched | 1 | *GPHN* | 0.0468 | 0.9871 |
| GO:0090051 | negative regulation of cell migration involved in sprouting angiogenesis | 15 | enriched | 1 | *THBS1* | 0.0422 | 0.9871 |
| GO:0090136 | epithelial cell-cell adhesion | 10 | enriched | 1 | *CTNNA1* | 0.0412 | 0.9871 |
| GO:0090197 | positive regulation of chemokine secretion | 12 | enriched | 1 | *C5* | 0.0307 | 0.9871 |
| GO:0090271 | positive regulation of fibroblast growth factor production | 5 | enriched | 2 | *CCM2L, PTGS2* | 0.0411 | 0.9871 |
| GO:0090647 | modulation of age-related behavioral decline | 9 | enriched | 1 | *GRM5* | 0.0493 | 0.9871 |
| GO:0090675 | intermicrovillar adhesion | 2 | enriched | 1 | *CDHR2* | 0.0374 | 0.9871 |
| GO:0097267 | omega-hydroxylase P450 pathway | 10 | enriched | 1 | *CYP1A1* | 0.0275 | 0.9871 |
| GO:0098962 | regulation of postsynaptic neurotransmitter receptor activity | 13 | enriched | 2 | *AKAP9, SHISA9* | 0.0296 | 0.9871 |
| GO:0099170 | postsynaptic modulation of chemical synaptic transmission | 9 | enriched | 1 | *GRM5* | 0.0341 | 0.9871 |
| GO:0106074 | aminoacyl-tRNA metabolism involved in translational fidelity | 13 | enriched | 1 | *IARS2* | 0.0199 | 0.9871 |
| GO:0120163 | negative regulation of cold-induced thermogenesis | 46 | enriched | 2 | *ACVR2B, ARRDC3* | 0.0292 | 0.9871 |
| GO:1900039 | positive regulation of cellular response to hypoxia | 1 | enriched | 1 | *KCNK2* | 0.0205 | 0.9871 |
| GO:1902043 | positive regulation of extrinsic apoptotic signaling pathway via death domain receptors | 11 | enriched | 1 | *THBS1* | 0.0182 | 0.9871 |
| GO:1902044 | regulation of Fas signaling pathway | 1 | enriched | 1 | *SP100* | 0.0476 | 0.9871 |
| GO:1902396 | protein localization to bicellular tight junction | 5 | enriched | 1 | *ACTN4* | 0.0230 | 0.9871 |
| GO:1903232 | melanosome assembly | 5 | enriched | 1 | *RAB38* | 0.0416 | 0.9871 |
| GO:1903358 | regulation of Golgi organization | 13 | enriched | 1 | *AKAP9* | 0.0265 | 0.9871 |
| GO:1903902 | positive regulation of viral life cycle | 6 | enriched | 1 | *KPNA2* | 0.0287 | 0.9871 |
| GO:1904262 | negative regulation of TORC1 signaling | 13 | enriched | 1 | *C7orf60* | 0.0307 | 0.9871 |
| GO:2000146 | negative regulation of cell motility | 12 | enriched | 2 | *CTNNA1, SPOCK3* | 0.0301 | 0.9871 |
| GO:2000304 | positive regulation of ceramide biosynthetic process | 11 | enriched | 1 | *CYR61* | 0.0230 | 0.9871 |
| GO:2000774 | positive regulation of cellular senescence | 13 | enriched | 1 | *HLA-G* | 0.0307 | 0.9871 |
| GO:2000786 | positive regulation of autophagosome assembly | 11 | enriched | 1 | *RAB3GAP2* | 0.0244 | 0.9871 |
| GO:2001247 | positive regulation of phosphatidylcholine biosynthetic process | 3 | enriched | 1 | *RAB38* | 0.0225 | 0.9871 |

| **S3b Table. Results of the pathway-based analysis of QNTS_Mean Pain and QNTS_Mean Anxiety (uncorrected p-value < 0.05).** | | | | | | | |
| --- | --- | --- | --- | --- | --- | --- | --- |
| **QNTS_Mean Anxiety** | | | | | | | |
| **GO set ID** | **Description** | **Pathway size (nr of genes)** | **Status** | **Significant Genes (nr of)** | **Genes** | ***p-value*** | **FDR** |
| GO:0014809 | regulation of skeletal muscle contraction by regulation of release of sequestered calcium ion | 3 | depleted | 0 |  | <0.0001 | <0.0001 |
| GO:0086001 | cardiac muscle cell action potential | 1 | depleted | 0 |  | <0.0001 | <0.0001 |
| GO:0043408 | regulation of MAPK cascade | 22 | enriched | 5 | *ID1, MUSK, SYNGAP1, ULK4, VRK2* | <0.0001 | 0.0040 |
| GO:0046902 | regulation of mitochondrial membrane permeability | 11 | enriched | 3 | *BCL2L1, BAK1, BNIP3* | <0.0001 | 0.0091 |
| GO:0000423 | mitophagy | 6 | enriched | 2 | *AMBRA1, ATG13* | <0.0001 | 0.0157 |
| GO:0043279 | response to alkaloid | 6 | enriched | 2 | *BCHE, SRSF9* | <0.0001 | 0.0157 |
| GO:0098780 | response to mitochondrial depolarisation | 6 | enriched | 2 | *AMBRA1, ATG13* | <0.0001 | 0.0157 |
| GO:0042742 | defense response to bacterium | 124 | enriched | 7 | *DEFB115, DEFB116, DEFB118, DEFB119, DEFB121, DEFB123, DEFB124* | <0.0001 | 0.0292 |
| GO:0061640 | cytoskeleton-dependent cytokinesis | 22 | enriched | 4 | *CECR2, ROPN1B, SEPT14, SEPT2* | <0.0001 | 0.0292 |
| GO:0008582 | regulation of synaptic growth at neuromuscular junction | 4 | enriched | 2 | *MUSK, SIX4* | <0.0001 | 0.0327 |
| GO:0007030 | Golgi organization | 112 | enriched | 9 | *DYM, CIT, CLASP2, GBF1, MYO18A, RAB2A, RAB7L1, SURF4, TRAPPC8* | <0.0001 | 0.0447 |
| GO:0051451 | myoblast migration | 9 | enriched | 2 | *ITGB1BP1, SIX4* | <0.0001 | 0.0447 |
| GO:1903896 | positive regulation of IRE1-mediated unfolded protein response | 7 | enriched | 2 | *BAK1, PTPN1* | <0.0001 | 0.0447 |
| GO:0008645 | hexose transmembrane transport | 13 | enriched | 3 | *SLC5A9, SLC2A8, SLC45A3* | <0.0001 | 0.0755 |
| GO:0010225 | response to UV-C | 10 | enriched | 2 | *BAK1, HMGN1* | <0.0001 | 0.0755 |
| GO:0014819 | regulation of skeletal muscle contraction | 4 | depleted | 0 |  | 0.0001 | 0.0915 |
| GO:0032012 | regulation of ARF protein signal transduction | 17 | enriched | 5 | *FBXO8, GBF1, IQSEC3, PSD, PSD3* | 0.0001 | 0.0915 |
| GO:0032233 | positive regulation of actin filament bundle assembly | 13 | enriched | 3 | *PFN1, ID1, SYNPO2* | 0.0001 | 0.1044 |
| GO:0048597 | post-embryonic camera-type eye morphogenesis | 3 | enriched | 2 | *BAK1, HMGN1* | 0.0001 | 0.1109 |
| GO:0032330 | regulation of chondrocyte differentiation | 10 | enriched | 2 | *KIAA1715, MDK* | 0.0001 | 0.1154 |
| GO:0006355 | regulation of transcription, DNA-templated | 781 | enriched | 33 | *ESRRG, BACH1, RORA, ACVR2B, CHD7, CHEK2, CREM, INS, NFKB2, PHF1, PITX3, SOX7, ZNF124, ZNF232, ZNF253, ZNF266, ZNF282, ZNF398, ZNF426, ZNF439, ZNF506, ZNF559, ZNF560, ZNF669, ZNF670, ZNF679, ZNF682, ZNF695, ZNF699, ZNF713, ZNF727, ZNF736, ZNF786* | 0.0002 | 0.1258 |
| GO:0030193 | regulation of blood coagulation | 8 | enriched | 2 | *F2, GP1BA* | 0.0003 | 0.1762 |
| GO:0031532 | actin cytoskeleton reorganization | 55 | enriched | 6 | *CDC42BPA, FARP2, GPR65, MINK1, PHACTR1, PTPN1* | 0.0003 | 0.1762 |
| GO:0050795 | regulation of behavior | 10 | enriched | 3 | *RELN, AHI1, MDK* | 0.0003 | 0.1762 |
| GO:0071599 | otic vesicle development | 6 | enriched | 2 | *AHI1, WRB* | 0.0003 | 0.1762 |
| GO:0010458 | exit from mitosis | 12 | enriched | 2 | *CLASP2, PPP2R2D* | 0.0004 | 0.1907 |
| GO:0010996 | response to auditory stimulus | 14 | enriched | 2 | *ABHD12, MDK* | 0.0004 | 0.1907 |
| GO:0050678 | regulation of epithelial cell proliferation | 14 | enriched | 2 | *HMGN1, SIX4* | 0.0004 | 0.1907 |
| GO:0051895 | negative regulation of focal adhesion assembly | 18 | enriched | 3 | *CLASP2, ITGB1BP1, MMP14* | 0.0004 | 0.2016 |
| GO:0002268 | follicular dendritic cell differentiation | 2 | enriched | 1 | *NFKB2* | 0.0015 | 0.2530 |
| GO:0002352 | B cell negative selection | 2 | enriched | 1 | *BAK1* | 0.0009 | 0.2530 |
| GO:0002521 | leukocyte differentiation | 3 | enriched | 1 | *RRAS* | 0.0015 | 0.2530 |
| GO:0002732 | positive regulation of dendritic cell cytokine production | 2 | enriched | 1 | *SCIMP* | 0.0016 | 0.2530 |
| GO:0006045 | N-acetylglucosamine biosynthetic process | 2 | enriched | 1 | *NANP* | 0.0012 | 0.2530 |
| GO:0006123 | mitochondrial electron transport, cytochrome c to oxygen | 20 | enriched | 2 | *COX4I2, COX6A1* | 0.0010 | 0.2530 |
| GO:0006660 | phosphatidylserine catabolic process | 2 | enriched | 1 | *ABHD12* | 0.0013 | 0.2530 |
| GO:0010046 | response to mycotoxin | 3 | enriched | 1 | *BAK1* | 0.0015 | 0.2530 |
| GO:0010621 | negative regulation of transcription by transcription factor localization | 3 | enriched | 1 | *ID1* | 0.0011 | 0.2530 |
| GO:0014016 | neuroblast differentiation | 3 | enriched | 1 | *BCHE* | 0.0015 | 0.2530 |
| GO:0014816 | skeletal muscle satellite cell differentiation | 5 | enriched | 2 | *CDON, MYLK2* | 0.0013 | 0.2530 |
| GO:0019046 | release from viral latency | 2 | enriched | 1 | *NUCKS1* | 0.0015 | 0.2530 |
| GO:0019050 | suppression by virus of host apoptotic process | 2 | enriched | 1 | *BCL2L1* | 0.0012 | 0.2530 |
| GO:0021681 | cerebellar granular layer development | 3 | enriched | 1 | *MDK* | 0.0016 | 0.2530 |
| GO:0030910 | olfactory placode formation | 3 | enriched | 1 | *SIX4* | 0.0016 | 0.2530 |
| GO:0032971 | regulation of muscle filament sliding | 2 | enriched | 1 | *MYLK2* | 0.0013 | 0.2530 |
| GO:0034453 | microtubule anchoring | 13 | enriched | 2 | *CEP350, CLASP2* | 0.0008 | 0.2530 |
| GO:0034497 | protein localization to phagophore assembly site | 12 | enriched | 2 | *ATG13, TRAPPC8* | 0.0007 | 0.2530 |
| GO:0034727 | piecemeal microautophagy of the nucleus | 3 | enriched | 1 | *ATG13* | 0.0015 | 0.2530 |
| GO:0036151 | phosphatidylcholine acyl-chain remodeling | 27 | enriched | 4 | *DBI, PLA2G6, PLB1, PNPLA8* | 0.0017 | 0.2530 |
| GO:0039530 | MDA-5 signaling pathway | 3 | enriched | 1 | *IRF3* | 0.0015 | 0.2530 |
| GO:0042048 | olfactory behavior | 7 | enriched | 2 | *UBR3, CHD7* | 0.0012 | 0.2530 |
| GO:0043666 | regulation of phosphoprotein phosphatase activity | 41 | enriched | 4 | *DMPK, PPP1R7, PPP2R2D, TSC1* | 0.0011 | 0.2530 |
| GO:0044346 | fibroblast apoptotic process | 3 | enriched | 1 | *BAK1* | 0.0016 | 0.2530 |
| GO:0044830 | modulation by host of viral RNA genome replication | 2 | enriched | 1 | *FBXL2* | 0.0012 | 0.2530 |
| GO:0045299 | otolith mineralization | 2 | enriched | 1 | *OTOL1* | 0.0013 | 0.2530 |
| GO:0045351 | type I interferon biosynthetic process | 2 | enriched | 1 | *IRF3* | 0.0008 | 0.2530 |
| GO:0061055 | myotome development | 3 | enriched | 1 | *SIX4* | 0.0016 | 0.2530 |
| GO:0061162 | establishment of monopolar cell polarity | 2 | enriched | 1 | *GBF1* | 0.0015 | 0.2530 |
| GO:0061551 | trigeminal ganglion development | 5 | enriched | 2 | *SEMA3A, SIX4* | 0.0016 | 0.2530 |
| GO:0070084 | protein initiator methionine removal | 3 | enriched | 1 | *METAP1* | 0.0016 | 0.2530 |
| GO:0070681 | glutaminyl-tRNAGln biosynthesis via transamidation | 3 | enriched | 1 | *GATC* | 0.0016 | 0.2530 |
| GO:0070838 | divalent metal ion transport | 2 | enriched | 1 | *SLC41A1* | 0.0012 | 0.2530 |
| GO:0070895 | negative regulation of transposon integration | 2 | enriched | 1 | *ZNF93* | 0.0015 | 0.2530 |
| GO:0071423 | malate transmembrane transport | 2 | enriched | 1 | *SLC25A11* | 0.0012 | 0.2530 |
| GO:0071888 | macrophage apoptotic process | 2 | enriched | 1 | *IRF3* | 0.0008 | 0.2530 |
| GO:0072095 | regulation of branch elongation involved in ureteric bud branching | 3 | enriched | 1 | *SIX4* | 0.0016 | 0.2530 |
| GO:0090201 | negative regulation of release of cytochrome c from mitochondria | 19 | enriched | 2 | *BCL2L1, TRIAP1* | 0.0016 | 0.2530 |
| GO:0097111 | endoplasmic reticulum-Golgi intermediate compartment organization | 2 | enriched | 1 | *GBF1* | 0.0015 | 0.2530 |
| GO:0098528 | skeletal muscle fiber differentiation | 2 | enriched | 1 | *SIX4* | 0.0010 | 0.2530 |
| GO:0098880 | maintenance of postsynaptic specialization structure | 2 | enriched | 1 | *SYNGAP1* | 0.0012 | 0.2530 |
| GO:0106015 | negative regulation of inflammatory response to wounding | 2 | enriched | 1 | *MDK* | 0.0008 | 0.2530 |
| GO:0106016 | positive regulation of inflammatory response to wounding | 2 | enriched | 1 | *MDK* | 0.0008 | 0.2530 |
| GO:0106091 | glial cell projection elongation | 2 | enriched | 1 | *MDK* | 0.0010 | 0.2530 |
| GO:0140014 | mitotic nuclear division | 3 | enriched | 1 | *USP16* | 0.0016 | 0.2530 |
| GO:1901666 | positive regulation of NAD+ ADP-ribosyltransferase activity | 3 | enriched | 1 | *HMGN1* | 0.0015 | 0.2530 |
| GO:1903039 | positive regulation of leukocyte cell-cell adhesion | 3 | enriched | 1 | *MDK* | 0.0016 | 0.2530 |
| GO:1903409 | reactive oxygen species biosynthetic process | 2 | enriched | 1 | *GBF1* | 0.0013 | 0.2530 |
| GO:1904036 | negative regulation of epithelial cell apoptotic process | 3 | enriched | 1 | *MDK* | 0.0010 | 0.2530 |
| GO:1904211 | membrane protein proteolysis involved in retrograde protein transport, ER to cytosol | 2 | enriched | 1 | *HM13* | 0.0012 | 0.2530 |
| GO:1904313 | response to methamphetamine hydrochloride | 2 | enriched | 1 | *PITX3* | 0.0010 | 0.2530 |
| GO:1905279 | regulation of retrograde transport, endosome to Golgi | 2 | enriched | 1 | *RAB7L1* | 0.0013 | 0.2530 |
| GO:1905555 | positive regulation blood vessel branching | 3 | enriched | 1 | *MDK* | 0.0016 | 0.2530 |
| GO:1904515 | positive regulation of TORC2 signaling | 2 | enriched | 1 | *SIK3* | 0.0017 | 0.2599 |
| GO:0097681 | double-strand break repair via alternative nonhomologous end joining | 3 | enriched | 1 | *POLQ* | 0.0018 | 0.2619 |
| GO:0009236 | cobalamin biosynthetic process | 3 | enriched | 1 | *MMAB* | 0.0018 | 0.2686 |
| GO:0043392 | negative regulation of DNA binding | 32 | enriched | 3 | *ID1, IFI16, WFIKKN2* | 0.0019 | 0.2691 |
| GO:0009967 | positive regulation of signal transduction | 63 | enriched | 5 | *TOB1, ARHGAP1, BLNK, ROPN1B, SH3BGR* | 0.0020 | 0.2709 |
| GO:0030330 | DNA damage response, signal transduction by p53 class mediator | 16 | enriched | 2 | *MYO6, TRIAP1* | 0.0020 | 0.2709 |
| GO:0070537 | histone H2A K63-linked deubiquitination | 3 | enriched | 1 | *USP16* | 0.0020 | 0.2709 |
| GO:0071596 | ubiquitin-dependent protein catabolic process via the N-end rule pathway | 3 | enriched | 1 | *UBR3* | 0.0020 | 0.2709 |
| GO:1902166 | negative regulation of intrinsic apoptotic signaling pathway in response to DNA damage by p53 class mediator | 16 | enriched | 2 | *BCL2L12, TRIAP1* | 0.0019 | 0.2709 |
| GO:0032232 | negative regulation of actin filament bundle assembly | 4 | enriched | 1 | *PFN1* | 0.0021 | 0.2712 |
| GO:0043651 | linoleic acid metabolic process | 17 | enriched | 2 | *ELOVL3, PNPLA8* | 0.0020 | 0.2712 |
| GO:0048713 | regulation of oligodendrocyte differentiation | 3 | enriched | 1 | *SLC45A3* | 0.0020 | 0.2712 |
| GO:0046716 | muscle cell cellular homeostasis | 21 | depleted | 0 |  | 0.0021 | 0.2718 |
| GO:0046338 | phosphatidylethanolamine catabolic process | 2 | enriched | 1 | *PNPLA8* | 0.0021 | 0.2727 |
| GO:0051881 | regulation of mitochondrial membrane potential | 28 | enriched | 3 | *BCL2L1, BAK1, NDUFS1* | 0.0022 | 0.2809 |
| GO:1902725 | negative regulation of satellite cell differentiation | 4 | enriched | 1 | *SIX4* | 0.0024 | 0.2990 |
| GO:0002367 | cytokine production involved in immune response | 3 | enriched | 1 | *SCIMP* | 0.0024 | 0.3004 |
| GO:0051823 | regulation of synapse structural plasticity | 5 | enriched | 2 | *CAMK2B, DMPK* | 0.0024 | 0.3004 |
| GO:0051046 | regulation of secretion | 2 | enriched | 1 | *MYO6* | 0.0025 | 0.3025 |
| GO:0032100 | positive regulation of appetite | 4 | enriched | 1 | *NPY* | 0.0025 | 0.3051 |
| GO:0045901 | positive regulation of translational elongation | 4 | enriched | 1 | *USP16* | 0.0025 | 0.3051 |
| GO:0006511 | ubiquitin-dependent protein catabolic process | 274 | enriched | 12 | *FBXL2, RNF150, RNF167, UBR3, ZNRF3, CUL2, FBXL15, FBXO8, KCTD10, UBE3B, USP16, USP6* | 0.0031 | 0.3087 |
| GO:0006933 | negative regulation of cell adhesion involved in substrate-bound cell migration | 4 | enriched | 1 | *ITGB1BP1* | 0.0029 | 0.3087 |
| GO:0009395 | phospholipid catabolic process | 9 | enriched | 2 | *ABHD12, LIPG* | 0.0028 | 0.3087 |
| GO:0010667 | negative regulation of cardiac muscle cell apoptotic process | 21 | enriched | 2 | *AMBRA1, MDK* | 0.0031 | 0.3087 |
| GO:0015742 | alpha-ketoglutarate transport | 4 | enriched | 1 | *SLC25A11* | 0.0026 | 0.3087 |
| GO:0031365 | N-terminal protein amino acid modification | 4 | enriched | 1 | *METAP1* | 0.0030 | 0.3087 |
| GO:0032147 | activation of protein kinase activity | 92 | enriched | 7 | *PAK7, ACVR2B, ATG13, MAP3K8, MINK1, TOM1L1, TPX2* | 0.0030 | 0.3087 |
| GO:0032258 | cytoplasm to vacuole transport by the Cvt pathway | 2 | enriched | 1 | *TRAPPC8* | 0.0028 | 0.3087 |
| GO:0038123 | toll-like receptor TLR1:TLR2 signaling pathway | 4 | enriched | 1 | *SCIMP* | 0.0031 | 0.3087 |
| GO:0044528 | regulation of mitochondrial mRNA stability | 4 | enriched | 1 | *TBRG4* | 0.0027 | 0.3087 |
| GO:0046661 | male sex differentiation | 4 | enriched | 1 | *SIX4* | 0.0029 | 0.3087 |
| GO:0055065 | metal ion homeostasis | 3 | enriched | 1 | *COX11* | 0.0026 | 0.3087 |
| GO:0060841 | venous blood vessel development | 4 | enriched | 1 | *ACVR2B* | 0.0030 | 0.3087 |
| GO:0071673 | positive regulation of smooth muscle cell chemotaxis | 4 | enriched | 1 | *MDK* | 0.0029 | 0.3087 |
| GO:0097035 | regulation of membrane lipid distribution | 5 | enriched | 1 | *TRIAP1* | 0.0028 | 0.3087 |
| GO:1902262 | apoptotic process involved in blood vessel morphogenesis | 4 | enriched | 1 | *BAK1* | 0.0029 | 0.3087 |
| GO:1902723 | negative regulation of skeletal muscle satellite cell proliferation | 4 | enriched | 1 | *SIX5* | 0.0031 | 0.3087 |
| GO:1905775 | negative regulation of DNA helicase activity | 2 | enriched | 1 | *MNAT1* | 0.0029 | 0.3087 |
| GO:2001044 | regulation of integrin-mediated signaling pathway | 4 | enriched | 1 | *ITGB1BP1* | 0.0027 | 0.3087 |
| GO:2001140 | positive regulation of phospholipid transport | 4 | enriched | 1 | *TRIAP1* | 0.0028 | 0.3087 |
| GO:2001184 | positive regulation of interleukin-12 secretion | 4 | enriched | 1 | *MDK* | 0.0028 | 0.3087 |
| GO:1902202 | regulation of hepatocyte growth factor receptor signaling pathway | 2 | enriched | 1 | *PTPN1* | 0.0032 | 0.3108 |
| GO:0000720 | pyrimidine dimer repair by nucleotide-excision repair | 4 | enriched | 1 | *HMGN1* | 0.0033 | 0.3161 |
| GO:0010830 | regulation of myotube differentiation | 5 | enriched | 1 | *DMPK* | 0.0034 | 0.3161 |
| GO:0033132 | negative regulation of glucokinase activity | 5 | enriched | 1 | *COX11* | 0.0033 | 0.3161 |
| GO:0035522 | monoubiquitinated histone H2A deubiquitination | 4 | enriched | 1 | *USP16* | 0.0034 | 0.3161 |
| GO:0072107 | positive regulation of ureteric bud formation | 4 | enriched | 1 | *SIX4* | 0.0033 | 0.3161 |
| GO:0090164 | asymmetric Golgi ribbon formation | 2 | enriched | 1 | *MYO18A* | 0.0033 | 0.3161 |
| GO:0001836 | release of cytochrome c from mitochondria | 22 | enriched | 2 | *BCL2L1, BAK1* | 0.0035 | 0.3233 |
| GO:0019695 | choline metabolic process | 4 | enriched | 1 | *BCHE* | 0.0035 | 0.3239 |
| GO:1902236 | negative regulation of endoplasmic reticulum stress-induced intrinsic apoptotic signaling pathway | 19 | enriched | 2 | *BCL2L1, PTPN1* | 0.0035 | 0.3243 |
| GO:0015770 | sucrose transport | 4 | enriched | 1 | *SLC45A3* | 0.0037 | 0.3253 |
| GO:0045943 | positive regulation of transcription by RNA polymerase I | 12 | enriched | 2 | *HEATR1, RASL11A* | 0.0036 | 0.3253 |
| GO:0046464 | acylglycerol catabolic process | 4 | enriched | 1 | *ABHD12* | 0.0037 | 0.3253 |
| GO:0071226 | cellular response to molecule of fungal origin | 4 | enriched | 1 | *SCIMP* | 0.0037 | 0.3253 |
| GO:0097202 | activation of cysteine-type endopeptidase activity | 14 | enriched | 2 | *BAK1, IFI16* | 0.0037 | 0.3253 |
| GO:0098885 | modification of postsynaptic actin cytoskeleton | 5 | enriched | 1 | *PFN1* | 0.0037 | 0.3253 |
| GO:1904659 | glucose transmembrane transport | 25 | enriched | 3 | *SLC5A9, MFSD4, SLC2A8* | 0.0037 | 0.3253 |
| GO:0045590 | negative regulation of regulatory T cell differentiation | 5 | enriched | 1 | *MDK* | 0.0038 | 0.3312 |
| GO:0061086 | negative regulation of histone H3-K27 methylation | 4 | enriched | 1 | *PHF1* | 0.0039 | 0.3356 |
| GO:0043496 | regulation of protein homodimerization activity | 5 | enriched | 1 | *BAK1* | 0.0040 | 0.3391 |
| GO:0038018 | Wnt receptor catabolic process | 2 | enriched | 1 | *ZNRF3* | 0.0042 | 0.3543 |
| GO:2000646 | positive regulation of receptor catabolic process | 3 | enriched | 1 | *PTPN1* | 0.0043 | 0.3633 |
| GO:1901842 | negative regulation of high voltage-gated calcium channel activity | 5 | enriched | 1 | *REM1* | 0.0046 | 0.3876 |
| GO:0099074 | mitochondrion to lysosome transport | 3 | depleted | 0 |  | 0.0047 | 0.3911 |
| GO:0019287 | isopentenyl diphosphate biosynthetic process, mevalonate pathway | 3 | enriched | 1 | *MVK* | 0.0048 | 0.3920 |
| GO:0000462 | maturation of SSU-rRNA from tricistronic rRNA transcript (SSU-rRNA, 5.8S rRNA, LSU-rRNA) | 26 | enriched | 4 | *NOL10, DHX37, DIEXF, HEATR1* | 0.0048 | 0.3936 |
| GO:0001974 | blood vessel remodeling | 31 | enriched | 3 | *ACVR2B, BAK1, CHD7* | 0.0049 | 0.3936 |
| GO:2000582 | positive regulation of ATP-dependent microtubule motor activity, plus-end-directed | 5 | enriched | 1 | *DYNLL1* | 0.0049 | 0.3936 |
| GO:0000422 | autophagy of mitochondrion | 37 | enriched | 3 | *AMBRA1, ATG13, BNIP3* | 0.0051 | 0.3982 |
| GO:0002232 | leukocyte chemotaxis involved in inflammatory response | 5 | enriched | 1 | *MDK* | 0.0050 | 0.3982 |
| GO:0016071 | mRNA metabolic process | 5 | enriched | 1 | *TBRG4* | 0.0052 | 0.3982 |
| GO:0018206 | peptidyl-methionine modification | 6 | enriched | 1 | *METAP1* | 0.0052 | 0.3982 |
| GO:0046850 | regulation of bone remodeling | 5 | enriched | 1 | *MDK* | 0.0052 | 0.3982 |
| GO:0070242 | thymocyte apoptotic process | 5 | enriched | 1 | *BAK1* | 0.0052 | 0.3982 |
| GO:0097300 | programmed necrotic cell death | 5 | enriched | 1 | *IRF3* | 0.0052 | 0.3982 |
| GO:1900103 | positive regulation of endoplasmic reticulum unfolded protein response | 5 | enriched | 1 | *BAK1* | 0.0051 | 0.3982 |
| GO:1903441 | protein localization to ciliary membrane | 5 | enriched | 1 | *RAB7L1* | 0.0052 | 0.3982 |
| GO:0021842 | chemorepulsion involved in interneuron migration from the subpallium to the cortex | 2 | depleted | 0 |  | 0.0054 | 0.4060 |
| GO:0044773 | mitotic DNA damage checkpoint | 3 | enriched | 2 | *CHEK2, STK33* | 0.0054 | 0.4060 |
| GO:0021987 | cerebral cortex development | 64 | enriched | 9 | *CDON, BNIP3, CNTNAP2, FOXP2, MDK, NPY, PHACTR1, TH, TSC1* | 0.0057 | 0.4244 |
| GO:0052651 | monoacylglycerol catabolic process | 5 | enriched | 1 | *ABHD12* | 0.0057 | 0.4244 |
| GO:0060154 | cellular process regulating host cell cycle in response to virus | 3 | enriched | 1 | *BCL2L1* | 0.0058 | 0.4250 |
| GO:0043243 | positive regulation of protein complex disassembly | 5 | enriched | 2 | *BNIP3, IGF1R* | 0.0059 | 0.4334 |
| GO:0043043 | peptide biosynthetic process | 4 | depleted | 0 |  | 0.0060 | 0.4343 |
| GO:0061087 | positive regulation of histone H3-K27 methylation | 5 | enriched | 1 | *PHF1* | 0.0060 | 0.4343 |
| GO:0086024 | adenylate cyclase-activating adrenergic receptor signaling pathway involved in positive regulation of heart rate | 1 | depleted | 0 |  | 0.0061 | 0.4377 |
| GO:0006206 | pyrimidine nucleobase metabolic process | 2 | enriched | 1 | *TYMP* | 0.0062 | 0.4391 |
| GO:0034154 | toll-like receptor 7 signaling pathway | 5 | enriched | 1 | *SCIMP* | 0.0062 | 0.4391 |
| GO:0006398 | mRNA 3'-end processing by stem-loop binding and cleavage | 5 | enriched | 1 | *CPSF3* | 0.0063 | 0.4440 |
| GO:1903377 | negative regulation of oxidative stress-induced neuron intrinsic apoptotic signaling pathway | 4 | depleted | 0 |  | 0.0064 | 0.4466 |
| GO:1990048 | anterograde neuronal dense core vesicle transport | 5 | enriched | 1 | *KIF1C* | 0.0064 | 0.4466 |
| GO:0060426 | lung vasculature development | 6 | enriched | 1 | *ID1* | 0.0065 | 0.4496 |
| GO:0098779 | positive regulation of mitophagy in response to mitochondrial depolarization | 4 | depleted | 0 |  | 0.0066 | 0.4496 |
| GO:0098958 | retrograde axonal transport of mitochondrion | 2 | depleted | 0 |  | 0.0066 | 0.4496 |
| GO:1990049 | retrograde neuronal dense core vesicle transport | 5 | enriched | 1 | *KIF1C* | 0.0065 | 0.4496 |
| GO:0010961 | cellular magnesium ion homeostasis | 5 | enriched | 1 | *SLC41A1* | 0.0066 | 0.4508 |
| GO:0006323 | DNA packaging | 6 | enriched | 1 | *KAT6A* | 0.0067 | 0.4514 |
| GO:0071711 | basement membrane organization | 13 | enriched | 2 | *NID2, RIC8A* | 0.0067 | 0.4514 |
| GO:0006027 | glycosaminoglycan catabolic process | 25 | depleted | 0 |  | 0.0068 | 0.4544 |
| GO:0001952 | regulation of cell-matrix adhesion | 12 | enriched | 2 | *MINK1, TSC1* | 0.0069 | 0.4558 |
| GO:0061178 | regulation of insulin secretion involved in cellular response to glucose stimulus | 16 | enriched | 3 | *SIDT2, ADCY5, STXBP4* | 0.0069 | 0.4558 |
| GO:0042780 | tRNA 3'-end processing | 4 | enriched | 1 | *SSB* | 0.0070 | 0.4562 |
| GO:1901653 | cellular response to peptide | 15 | enriched | 2 | *ATP5H, ID1* | 0.0070 | 0.4562 |
| GO:1902283 | negative regulation of primary amine oxidase activity | 2 | depleted | 0 |  | 0.0070 | 0.4562 |
| GO:1903690 | negative regulation of wound healing, spreading of epidermal cells | 4 | enriched | 1 | *CLASP2* | 0.0071 | 0.4584 |
| GO:0061734 | parkin-mediated stimulation of mitophagy in response to mitochondrial depolarization | 5 | depleted | 0 |  | 0.0071 | 0.4587 |
| GO:0009117 | nucleotide metabolic process | 10 | depleted | 0 |  | 0.0072 | 0.4628 |
| GO:0006888 | endoplasmic reticulum to Golgi vesicle-mediated transport | 190 | enriched | 13 | *ANK2, BET1L, DYNLL1, F2, GBF1, GOSR2, INS, RAB2A, RAB7L1, RP11-156P1.2, SEC22A, TGFA, TRAPPC8* | 0.0074 | 0.4661 |
| GO:0007158 | neuron cell-cell adhesion | 16 | depleted | 0 |  | 0.0074 | 0.4661 |
| GO:0060314 | regulation of ryanodine-sensitive calcium-release channel activity | 16 | depleted | 0 |  | 0.0074 | 0.4661 |
| GO:1905333 | regulation of gastric motility | 3 | enriched | 1 | *TYMP* | 0.0074 | 0.4661 |
| GO:1901214 | regulation of neuron death | 17 | enriched | 2 | *RAB7L1, TSC1* | 0.0075 | 0.4673 |
| GO:0007597 | blood coagulation, intrinsic pathway | 17 | enriched | 2 | *F2, GP1BA* | 0.0077 | 0.4784 |
| GO:1903382 | negative regulation of endoplasmic reticulum stress-induced neuron intrinsic apoptotic signaling pathway | 2 | depleted | 0 |  | 0.0078 | 0.4784 |
| GO:0090315 | negative regulation of protein targeting to membrane | 6 | enriched | 1 | *ITGB1BP1* | 0.0079 | 0.4866 |
| GO:1902749 | regulation of cell cycle G2/M phase transition | 5 | enriched | 1 | *NEK10* | 0.0080 | 0.4874 |
| GO:0006450 | regulation of translational fidelity | 6 | enriched | 1 | *GATC* | 0.0085 | 0.4943 |
| GO:0009620 | response to fungus | 6 | enriched | 1 | *BAK1* | 0.0083 | 0.4943 |
| GO:0031396 | regulation of protein ubiquitination | 15 | depleted | 0 |  | 0.0085 | 0.4943 |
| GO:0040012 | regulation of locomotion | 6 | enriched | 1 | *CHRM4* | 0.0084 | 0.4943 |
| GO:0043113 | receptor clustering | 28 | enriched | 3 | *DLG2, ITGB1BP1, SYNGAP1* | 0.0085 | 0.4943 |
| GO:0043524 | negative regulation of neuron apoptotic process | 137 | enriched | 8 | *BCL2L1, AMBRA1, ANGPT1, MDK, OXR1, PRKCI, SIX4, SYNGAP1* | 0.0082 | 0.4943 |
| GO:0045602 | negative regulation of endothelial cell differentiation | 6 | enriched | 1 | *ID1* | 0.0085 | 0.4943 |
| GO:0048205 | COPI coating of Golgi vesicle | 6 | enriched | 1 | *GBF1* | 0.0085 | 0.4943 |
| GO:0060836 | lymphatic endothelial cell differentiation | 6 | enriched | 1 | *ACVR2B* | 0.0085 | 0.4943 |
| GO:0071816 | tail-anchored membrane protein insertion into ER membrane | 6 | enriched | 1 | *WRB* | 0.0084 | 0.4943 |
| GO:0090166 | Golgi disassembly | 6 | enriched | 1 | *GBF1* | 0.0085 | 0.4943 |
| GO:0070124 | mitochondrial translational initiation | 2 | enriched | 1 | *MTIF3* | 0.0089 | 0.5121 |
| GO:2000327 | positive regulation of nuclear receptor transcription coactivator activity | 3 | enriched | 1 | *CNOT6* | 0.0090 | 0.5135 |
| GO:1901379 | regulation of potassium ion transmembrane transport | 16 | depleted | 0 |  | 0.0094 | 0.5333 |
| GO:1905475 | regulation of protein localization to membrane | 6 | depleted | 0 |  | 0.0094 | 0.5333 |
| GO:0007127 | meiosis I | 6 | enriched | 1 | *SCIMP* | 0.0095 | 0.5377 |
| GO:0048194 | Golgi vesicle budding | 4 | enriched | 2 | *MYO18A, PRKCI* | 0.0096 | 0.5381 |
| GO:0051582 | positive regulation of neurotransmitter uptake | 1 | depleted | 0 |  | 0.0097 | 0.5381 |
| GO:0071786 | endoplasmic reticulum tubular network organization | 9 | enriched | 2 | *KIAA1715, RTN4* | 0.0097 | 0.5381 |
| GO:1905281 | positive regulation of retrograde transport, endosome to Golgi | 1 | depleted | 0 |  | 0.0097 | 0.5381 |
| GO:0034638 | phosphatidylcholine catabolic process | 5 | enriched | 1 | *PNPLA8* | 0.0098 | 0.5395 |
| GO:0010524 | positive regulation of calcium ion transport into cytosol | 12 | enriched | 3 | *BAK1, CD4, TRPC3* | 0.0098 | 0.5399 |
| GO:0034316 | negative regulation of Arp2/3 complex-mediated actin nucleation | 6 | depleted | 0 |  | 0.0100 | 0.5438 |
| GO:0044829 | positive regulation by host of viral genome replication | 6 | enriched | 1 | *NUCKS1* | 0.0100 | 0.5438 |
| GO:1903373 | positive regulation of endoplasmic reticulum tubular network organization | 4 | enriched | 1 | *KIAA1715* | 0.0100 | 0.5438 |
| GO:1904996 | positive regulation of leukocyte adhesion to vascular endothelial cell | 10 | enriched | 2 | *ETS1, MDK* | 0.0104 | 0.5583 |
| GO:0038129 | ERBB3 signaling pathway | 1 | depleted | 0 |  | 0.0104 | 0.5593 |
| GO:0031954 | positive regulation of protein autophosphorylation | 23 | enriched | 3 | *INS, NEK10, TOM1L1* | 0.0107 | 0.5658 |
| GO:0086097 | phospholipase C-activating angiotensin-activated signaling pathway | 2 | enriched | 1 | *ACTN2* | 0.0107 | 0.5658 |
| GO:0099542 | trans-synaptic signaling by endocannabinoid | 1 | depleted | 0 |  | 0.0107 | 0.5658 |
| GO:1904893 | negative regulation of receptor signaling pathway via STAT | 1 | depleted | 0 |  | 0.0106 | 0.5658 |
| GO:0030099 | myeloid cell differentiation | 18 | enriched | 2 | *KAT6A, IFI16* | 0.0109 | 0.5703 |
| GO:0006213 | pyrimidine nucleoside metabolic process | 3 | enriched | 1 | *TYMP* | 0.0111 | 0.5736 |
| GO:0032091 | negative regulation of protein binding | 75 | enriched | 6 | *AES, DISC1, ID1, ITGB1BP1, SYMPK, WFIKKN2* | 0.0111 | 0.5736 |
| GO:0034620 | cellular response to unfolded protein | 25 | enriched | 2 | *BAK1, PTPN1* | 0.0112 | 0.5736 |
| GO:1904261 | positive regulation of basement membrane assembly involved in embryonic body morphogenesis | 5 | enriched | 1 | *CLASP2* | 0.0111 | 0.5736 |
| GO:1990116 | ribosome-associated ubiquitin-dependent protein catabolic process | 2 | enriched | 1 | *LTN1* | 0.0112 | 0.5736 |
| GO:1990264 | peptidyl-tyrosine dephosphorylation involved in inactivation of protein kinase activity | 5 | enriched | 1 | *PTPN1* | 0.0112 | 0.5736 |
| GO:0003402 | planar cell polarity pathway involved in axis elongation | 4 | depleted | 0 |  | 0.0114 | 0.5826 |
| GO:0061197 | fungiform papilla morphogenesis | 2 | enriched | 1 | *SIX4* | 0.0116 | 0.5848 |
| GO:0071286 | cellular response to magnesium ion | 7 | enriched | 1 | *SLC41A1* | 0.0116 | 0.5848 |
| GO:0002043 | blood vessel endothelial cell proliferation involved in sprouting angiogenesis | 7 | enriched | 1 | *ITGB1BP1* | 0.0119 | 0.5856 |
| GO:0008635 | activation of cysteine-type endopeptidase activity involved in apoptotic process by cytochrome c | 7 | enriched | 1 | *BAK1* | 0.0119 | 0.5856 |
| GO:0021781 | glial cell fate commitment | 4 | depleted | 0 |  | 0.0117 | 0.5856 |
| GO:0070586 | cell-cell adhesion involved in gastrulation | 1 | enriched | 1 | *RIC8A* | 0.0118 | 0.5856 |
| GO:2000095 | regulation of Wnt signaling pathway, planar cell polarity pathway | 4 | enriched | 1 | *ZNRF3* | 0.0117 | 0.5856 |
| GO:2000809 | positive regulation of synaptic vesicle clustering | 5 | depleted | 0 |  | 0.0118 | 0.5856 |
| GO:0006696 | ergosterol biosynthetic process | 5 | enriched | 1 | *C14orf1* | 0.0119 | 0.5864 |
| GO:0055069 | zinc ion homeostasis | 5 | depleted | 0 |  | 0.0121 | 0.5911 |
| GO:0002467 | germinal center formation | 8 | enriched | 2 | *ADAM17, NFKB2* | 0.0127 | 0.6039 |
| GO:0019367 | fatty acid elongation, saturated fatty acid | 7 | enriched | 1 | *ELOVL3* | 0.0129 | 0.6039 |
| GO:0032471 | negative regulation of endoplasmic reticulum calcium ion concentration | 7 | enriched | 1 | *BAK1* | 0.0126 | 0.6039 |
| GO:0034625 | fatty acid elongation, monounsaturated fatty acid | 7 | enriched | 1 | *ELOVL3* | 0.0129 | 0.6039 |
| GO:0034626 | fatty acid elongation, polyunsaturated fatty acid | 7 | enriched | 1 | *ELOVL3* | 0.0129 | 0.6039 |
| GO:0050774 | negative regulation of dendrite morphogenesis | 9 | enriched | 1 | *ID1* | 0.0129 | 0.6039 |
| GO:0051155 | positive regulation of striated muscle cell differentiation | 5 | depleted | 0 |  | 0.0127 | 0.6039 |
| GO:0051593 | response to folic acid | 7 | enriched | 1 | *BCHE* | 0.0126 | 0.6039 |
| GO:0060840 | artery development | 7 | enriched | 1 | *ACVR2B* | 0.0127 | 0.6039 |
| GO:2000278 | regulation of DNA biosynthetic process | 5 | enriched | 1 | *TOM1L1* | 0.0125 | 0.6039 |
| GO:2000391 | positive regulation of neutrophil extravasation | 7 | enriched | 1 | *MDK* | 0.0125 | 0.6039 |
| GO:0061298 | retina vasculature development in camera-type eye | 7 | enriched | 1 | *ACVR2B* | 0.0130 | 0.6046 |
| GO:1900383 | regulation of synaptic plasticity by receptor localization to synapse | 1 | depleted | 0 |  | 0.0132 | 0.6115 |
| GO:0006552 | leucine catabolic process | 6 | enriched | 1 | *AUH* | 0.0134 | 0.6142 |
| GO:0010764 | negative regulation of fibroblast migration | 7 | enriched | 1 | *ITGB1BP1* | 0.0135 | 0.6142 |
| GO:0099170 | postsynaptic modulation of chemical synaptic transmission | 9 | depleted | 0 |  | 0.0133 | 0.6142 |
| GO:1901844 | regulation of cell communication by electrical coupling involved in cardiac conduction | 8 | depleted | 0 |  | 0.0134 | 0.6142 |
| GO:1903898 | negative regulation of PERK-mediated unfolded protein response | 5 | enriched | 1 | *PTPN1* | 0.0134 | 0.6142 |
| GO:0098586 | cellular response to virus | 21 | enriched | 2 | *GBF1, LGALS8* | 0.0138 | 0.6253 |
| GO:0036503 | ERAD pathway | 16 | depleted | 0 |  | 0.0140 | 0.6264 |
| GO:0051571 | positive regulation of histone H3-K4 methylation | 15 | depleted | 0 |  | 0.0140 | 0.6264 |
| GO:0051838 | cytolysis by host of symbiont cells | 1 | enriched | 1 | *F2* | 0.0140 | 0.6264 |
| GO:1904977 | lymphatic endothelial cell migration | 1 | enriched | 1 | *LGALS8* | 0.0139 | 0.6264 |
| GO:0071875 | adrenergic receptor signaling pathway | 7 | depleted | 0 |  | 0.0142 | 0.6301 |
| GO:0090216 | positive regulation of 1-phosphatidylinositol-4-phosphate 5-kinase activity | 1 | enriched | 1 | *DGKZ* | 0.0142 | 0.6301 |
| GO:0007342 | fusion of sperm to egg plasma membrane involved in single fertilization | 12 | enriched | 2 | *ROPN1B, SPACA3* | 0.0143 | 0.6302 |
| GO:0010634 | positive regulation of epithelial cell migration | 35 | enriched | 3 | *PFN1, CLASP2, RTN4* | 0.0143 | 0.6302 |
| GO:0051969 | regulation of transmission of nerve impulse | 4 | enriched | 1 | *TYMP* | 0.0142 | 0.6302 |
| GO:0046931 | pore complex assembly | 11 | enriched | 2 | *CCT8, PLEKHA7* | 0.0144 | 0.6309 |
| GO:0032927 | positive regulation of activin receptor signaling pathway | 7 | enriched | 1 | *ACVR2B* | 0.0145 | 0.6318 |
| GO:0006163 | purine nucleotide metabolic process | 7 | depleted | 0 |  | 0.0148 | 0.6334 |
| GO:0043379 | memory T cell differentiation | 1 | enriched | 1 | *TSC1* | 0.0146 | 0.6334 |
| GO:0045822 | negative regulation of heart contraction | 7 | depleted | 0 |  | 0.0148 | 0.6334 |
| GO:0090218 | positive regulation of lipid kinase activity | 2 | enriched | 1 | *F2* | 0.0147 | 0.6334 |
| GO:1902254 | negative regulation of intrinsic apoptotic signaling pathway by p53 class mediator | 5 | depleted | 0 |  | 0.0147 | 0.6334 |
| GO:0051695 | actin filament uncapping | 1 | enriched | 1 | *ACTN2* | 0.0149 | 0.6335 |
| GO:0060596 | mammary placode formation | 3 | depleted | 0 |  | 0.0148 | 0.6335 |
| GO:0071654 | positive regulation of chemokine (C-C motif) ligand 1 production | 1 | enriched | 1 | *TSLP* | 0.0150 | 0.6365 |
| GO:0000045 | autophagosome assembly | 56 | enriched | 4 | *AMBRA1, ATG13, SYNPO2, TRAPPC8* | 0.0151 | 0.6369 |
| GO:0048513 | animal organ development | 17 | depleted | 0 |  | 0.0151 | 0.6369 |
| GO:0032435 | negative regulation of proteasomal ubiquitin-dependent protein catabolic process | 29 | depleted | 0 |  | 0.0152 | 0.6372 |
| GO:0051497 | negative regulation of stress fiber assembly | 26 | enriched | 2 | *PFN1, CLASP2* | 0.0152 | 0.6372 |
| GO:0006407 | rRNA export from nucleus | 2 | enriched | 1 | *TSC1* | 0.0154 | 0.6397 |
| GO:0035791 | platelet-derived growth factor receptor-beta signaling pathway | 5 | enriched | 1 | *PTPN1* | 0.0154 | 0.6397 |
| GO:0031279 | regulation of cyclase activity | 1 | depleted | 0 |  | 0.0157 | 0.6464 |
| GO:0070966 | nuclear-transcribed mRNA catabolic process, no-go decay | 4 | enriched | 1 | *CNOT6* | 0.0157 | 0.6464 |
| GO:0060087 | relaxation of vascular smooth muscle | 7 | depleted | 0 |  | 0.0158 | 0.6466 |
| GO:0098792 | xenophagy | 3 | enriched | 1 | *LGALS8* | 0.0158 | 0.6466 |
| GO:0007274 | neuromuscular synaptic transmission | 30 | enriched | 2 | *CHRNE, MYLK2* | 0.0161 | 0.6532 |
| GO:0050953 | sensory perception of light stimulus | 9 | depleted | 0 |  | 0.0161 | 0.6532 |
| GO:0051673 | membrane disruption in other organism | 9 | enriched | 1 | *DEFB118* | 0.0161 | 0.6532 |
| GO:0043378 | positive regulation of CD8-positive, alpha-beta T cell differentiation | 4 | depleted | 0 |  | 0.0164 | 0.6569 |
| GO:0048854 | brain morphogenesis | 18 | depleted | 0 |  | 0.0163 | 0.6569 |
| GO:0070973 | protein localization to endoplasmic reticulum exit site | 7 | enriched | 1 | *GBF1* | 0.0163 | 0.6569 |
| GO:0097192 | extrinsic apoptotic signaling pathway in absence of ligand | 33 | enriched | 3 | *BCL2L1, BAK1, MOAP1* | 0.0166 | 0.6648 |
| GO:0045494 | photoreceptor cell maintenance | 38 | depleted | 0 |  | 0.0167 | 0.6658 |
| GO:2000541 | positive regulation of protein geranylgeranylation | 1 | enriched | 1 | *MUSK* | 0.0167 | 0.6658 |
| GO:0010038 | response to metal ion | 13 | enriched | 2 | *CUTA, MT1A* | 0.0169 | 0.6716 |
| GO:0042730 | fibrinolysis | 21 | enriched | 2 | *F2, GP1BA* | 0.0172 | 0.6790 |
| GO:0051668 | localization within membrane | 3 | depleted | 0 |  | 0.0172 | 0.6790 |
| GO:0035265 | organ growth | 8 | enriched | 1 | *ACVR2B* | 0.0174 | 0.6819 |
| GO:0035721 | intraciliary retrograde transport | 9 | enriched | 2 | *IFT43, DYNC2LI1* | 0.0174 | 0.6819 |
| GO:0070372 | regulation of ERK1 and ERK2 cascade | 29 | enriched | 2 | *RRAS, NEK10* | 0.0176 | 0.6867 |
| GO:0071312 | cellular response to alkaloid | 8 | enriched | 2 | *BCL2L1, TH* | 0.0177 | 0.6867 |
| GO:0045723 | positive regulation of fatty acid biosynthetic process | 12 | enriched | 2 | *LPGAT1, SLC45A3* | 0.0179 | 0.6921 |
| GO:0007197 | adenylate cyclase-inhibiting G protein-coupled acetylcholine receptor signaling pathway | 8 | enriched | 1 | *CHRM4* | 0.0180 | 0.6938 |
| GO:0030242 | autophagy of peroxisome | 5 | enriched | 1 | *TRAPPC8* | 0.0180 | 0.6940 |
| GO:0050805 | negative regulation of synaptic transmission | 8 | enriched | 1 | *BCHE* | 0.0181 | 0.6957 |
| GO:0050806 | positive regulation of synaptic transmission | 18 | depleted | 0 |  | 0.0183 | 0.6961 |
| GO:0051153 | regulation of striated muscle cell differentiation | 1 | depleted | 0 |  | 0.0184 | 0.6961 |
| GO:1901385 | regulation of voltage-gated calcium channel activity | 7 | depleted | 0 |  | 0.0184 | 0.6961 |
| GO:1990678 | histone H4-K16 deacetylation | 1 | depleted | 0 |  | 0.0184 | 0.6961 |
| GO:2000347 | positive regulation of hepatocyte proliferation | 8 | enriched | 1 | *MDK* | 0.0183 | 0.6961 |
| GO:0072075 | metanephric mesenchyme development | 8 | enriched | 1 | *SIX4* | 0.0185 | 0.6970 |
| GO:1904049 | negative regulation of spontaneous neurotransmitter secretion | 2 | depleted | 0 |  | 0.0186 | 0.6970 |
| GO:0002690 | positive regulation of leukocyte chemotaxis | 12 | enriched | 3 | *ADAM17, EDN2, MDK* | 0.0187 | 0.6993 |
| GO:0050908 | detection of light stimulus involved in visual perception | 18 | depleted | 0 |  | 0.0187 | 0.6993 |
| GO:0070100 | negative regulation of chemokine-mediated signaling pathway | 7 | depleted | 0 |  | 0.0188 | 0.6993 |
| GO:0006379 | mRNA cleavage | 8 | enriched | 1 | *CPSF3* | 0.0189 | 0.6997 |
| GO:0001783 | B cell apoptotic process | 8 | enriched | 1 | *BAK1* | 0.0191 | 0.7011 |
| GO:0035773 | insulin secretion involved in cellular response to glucose stimulus | 7 | depleted | 0 |  | 0.0190 | 0.7011 |
| GO:0043534 | blood vessel endothelial cell migration | 9 | enriched | 1 | *ID1* | 0.0191 | 0.7011 |
| GO:2000051 | negative regulation of non-canonical Wnt signaling pathway | 4 | enriched | 1 | *ZNRF3* | 0.0190 | 0.7011 |
| GO:0060379 | cardiac muscle cell myoblast differentiation | 3 | depleted | 0 |  | 0.0193 | 0.7034 |
| GO:0061300 | cerebellum vasculature development | 1 | depleted | 0 |  | 0.0193 | 0.7034 |
| GO:0014737 | positive regulation of muscle atrophy | 2 | enriched | 1 | *FOXO3* | 0.0195 | 0.7070 |
| GO:0048789 | cytoskeletal matrix organization at active zone | 1 | depleted | 0 |  | 0.0197 | 0.7081 |
| GO:0097115 | neurexin clustering involved in presynaptic membrane assembly | 1 | depleted | 0 |  | 0.0197 | 0.7081 |
| GO:1905520 | positive regulation of presynaptic active zone assembly | 1 | depleted | 0 |  | 0.0197 | 0.7081 |
| GO:0006620 | posttranslational protein targeting to endoplasmic reticulum membrane | 8 | enriched | 1 | *WRB* | 0.0201 | 0.7096 |
| GO:0007160 | cell-matrix adhesion | 94 | enriched | 7 | *DEFB118, NID2, ADAMTS12, EPDR1, ITGB1BP1, TECTA, TSC1* | 0.0200 | 0.7096 |
| GO:0031666 | positive regulation of lipopolysaccharide-mediated signaling pathway | 8 | enriched | 1 | *SCIMP* | 0.0200 | 0.7096 |
| GO:0070886 | positive regulation of calcineurin-NFAT signaling cascade | 15 | depleted | 0 |  | 0.0199 | 0.7096 |
| GO:1902530 | positive regulation of protein linear polyubiquitination | 2 | depleted | 0 |  | 0.0200 | 0.7096 |
| GO:1904220 | regulation of serine C-palmitoyltransferase activity | 1 | enriched | 1 | *SPTSSB* | 0.0199 | 0.7096 |
| GO:0030961 | peptidyl-arginine hydroxylation | 1 | enriched | 1 | *NDUFAF5* | 0.0202 | 0.7116 |
| GO:0045765 | regulation of angiogenesis | 32 | enriched | 4 | *ETS1, ID1, TSPAN12, VASH2* | 0.0203 | 0.7116 |
| GO:0090200 | positive regulation of release of cytochrome c from mitochondria | 29 | enriched | 4 | *BAK1, BNIP3, MOAP1, PLA2G6* | 0.0203 | 0.7116 |
| GO:0031023 | microtubule organizing center organization | 7 | enriched | 1 | *CLASP2* | 0.0205 | 0.7121 |
| GO:0033227 | dsRNA transport | 5 | enriched | 1 | *SIDT2* | 0.0205 | 0.7121 |
| GO:0099178 | regulation of retrograde trans-synaptic signaling by endocanabinoid | 2 | depleted | 0 |  | 0.0204 | 0.7121 |
| GO:0009060 | aerobic respiration | 30 | enriched | 3 | *COX6A1, SIRT3, SURF1* | 0.0208 | 0.7220 |
| GO:0045837 | negative regulation of membrane potential | 2 | enriched | 1 | *BNIP3* | 0.0210 | 0.7255 |
| GO:1904744 | positive regulation of telomeric DNA binding | 1 | enriched | 1 | *PINX1* | 0.0211 | 0.7264 |
| GO:0098742 | cell-cell adhesion via plasma-membrane adhesion molecules | 11 | depleted | 0 |  | 0.0214 | 0.7346 |
| GO:2000377 | regulation of reactive oxygen species metabolic process | 18 | depleted | 0 |  | 0.0216 | 0.7408 |
| GO:0034454 | microtubule anchoring at centrosome | 8 | enriched | 1 | *NINL* | 0.0222 | 0.7578 |
| GO:0043497 | regulation of protein heterodimerization activity | 9 | enriched | 1 | *BAK1* | 0.0222 | 0.7578 |
| GO:0050957 | equilibrioception | 6 | depleted | 0 |  | 0.0224 | 0.7589 |
| GO:0070945 | neutrophil mediated killing of gram-negative bacterium | 3 | enriched | 1 | *F2* | 0.0224 | 0.7589 |
| GO:0099054 | presynapse assembly | 13 | depleted | 0 |  | 0.0226 | 0.7615 |
| GO:1990144 | intrinsic apoptotic signaling pathway in response to hypoxia | 2 | enriched | 1 | *BNIP3* | 0.0226 | 0.7615 |
| GO:0060060 | post-embryonic retina morphogenesis in camera-type eye | 1 | depleted | 0 |  | 0.0230 | 0.7660 |
| GO:0060466 | activation of meiosis involved in egg activation | 1 | depleted | 0 |  | 0.0230 | 0.7660 |
| GO:2000438 | negative regulation of monocyte extravasation | 1 | depleted | 0 |  | 0.0230 | 0.7660 |
| GO:2000560 | positive regulation of CD24 biosynthetic process | 1 | depleted | 0 |  | 0.0230 | 0.7660 |
| GO:0010636 | positive regulation of mitochondrial fusion | 3 | depleted | 0 |  | 0.0233 | 0.7738 |
| GO:0032368 | regulation of lipid transport | 2 | depleted | 0 |  | 0.0235 | 0.7756 |
| GO:2000659 | regulation of interleukin-1-mediated signaling pathway | 1 | enriched | 1 | *VRK2* | 0.0234 | 0.7756 |
| GO:0039694 | viral RNA genome replication | 9 | enriched | 2 | *PIK3C2G, RAB7L1* | 0.0237 | 0.7810 |
| GO:0021836 | chemorepulsion involved in postnatal olfactory bulb interneuron migration | 2 | depleted | 0 |  | 0.0240 | 0.7870 |
| GO:0030035 | microspike assembly | 4 | enriched | 1 | *ACTN2* | 0.0240 | 0.7870 |
| GO:0070493 | thrombin-activated receptor signaling pathway | 9 | enriched | 1 | *GP1BA* | 0.0241 | 0.7891 |
| GO:0090307 | mitotic spindle assembly | 31 | enriched | 3 | *CHEK2, KIFC1, TPX2* | 0.0243 | 0.7932 |
| GO:0043616 | keratinocyte proliferation | 11 | depleted | 0 |  | 0.0244 | 0.7935 |
| GO:0090522 | vesicle tethering involved in exocytosis | 1 | depleted | 0 |  | 0.0245 | 0.7953 |
| GO:0060173 | limb development | 31 | enriched | 3 | *ZNRF3, CHD7, KIAA1715* | 0.0246 | 0.7955 |
| GO:0002262 | myeloid cell homeostasis | 9 | enriched | 1 | *BAK1* | 0.0249 | 0.7985 |
| GO:0006417 | regulation of translation | 63 | enriched | 4 | *METAP1, CNOT6, FOXO3, TSC1* | 0.0251 | 0.7985 |
| GO:0007614 | short-term memory | 9 | enriched | 1 | *MDK* | 0.0252 | 0.7985 |
| GO:0016239 | positive regulation of macroautophagy | 33 | enriched | 3 | *PAFAH1B2, BNIP3, TSC1* | 0.0250 | 0.7985 |
| GO:0045333 | cellular respiration | 17 | enriched | 2 | *COX4I2, NDUFS1* | 0.0250 | 0.7985 |
| GO:0060575 | intestinal epithelial cell differentiation | 9 | enriched | 1 | *NPY* | 0.0249 | 0.7985 |
| GO:0072553 | terminal button organization | 3 | depleted | 0 |  | 0.0252 | 0.7985 |
| GO:0002088 | lens development in camera-type eye | 39 | enriched | 3 | *CDON, PITX3, SIX5* | 0.0253 | 0.8011 |
| GO:0032481 | positive regulation of type I interferon production | 51 | enriched | 3 | *IRF3, IFI16, NFKB2* | 0.0255 | 0.8042 |
| GO:0072599 | establishment of protein localization to endoplasmic reticulum | 1 | depleted | 0 |  | 0.0262 | 0.8244 |
| GO:0002084 | protein depalmitoylation | 9 | enriched | 1 | *ABHD12* | 0.0277 | 0.8333 |
| GO:0003025 | regulation of systemic arterial blood pressure by baroreceptor feedback | 1 | depleted | 0 |  | 0.0278 | 0.8333 |
| GO:0008343 | adult feeding behavior | 10 | enriched | 1 | *NPY* | 0.0271 | 0.8333 |
| GO:0010994 | free ubiquitin chain polymerization | 6 | depleted | 0 |  | 0.0271 | 0.8333 |
| GO:0021563 | glossopharyngeal nerve development | 1 | depleted | 0 |  | 0.0278 | 0.8333 |
| GO:0021564 | vagus nerve development | 1 | depleted | 0 |  | 0.0278 | 0.8333 |
| GO:0030862 | positive regulation of polarized epithelial cell differentiation | 1 | enriched | 1 | *AHI1* | 0.0276 | 0.8333 |
| GO:0033622 | integrin activation | 9 | enriched | 1 | *ITGB1BP1* | 0.0275 | 0.8333 |
| GO:0035844 | cloaca development | 1 | enriched | 1 | *AHI1* | 0.0276 | 0.8333 |
| GO:0039008 | pronephric nephron tubule morphogenesis | 1 | enriched | 1 | *AHI1* | 0.0276 | 0.8333 |
| GO:0039023 | pronephric duct morphogenesis | 1 | enriched | 1 | *AHI1* | 0.0276 | 0.8333 |
| GO:0045184 | establishment of protein localization | 40 | depleted | 0 |  | 0.0268 | 0.8333 |
| GO:0045214 | sarcomere organization | 40 | enriched | 3 | *ACTN2, MYOZ2, SIX4* | 0.0273 | 0.8333 |
| GO:0048617 | embryonic foregut morphogenesis | 9 | enriched | 1 | *ACVR2B* | 0.0277 | 0.8333 |
| GO:0051496 | positive regulation of stress fiber assembly | 51 | enriched | 3 | *GPR65, ITGB1BP1, TSC1* | 0.0277 | 0.8333 |
| GO:0061743 | motor learning | 5 | depleted | 0 |  | 0.0266 | 0.8333 |
| GO:0070121 | Kupffer's vesicle development | 1 | enriched | 1 | *AHI1* | 0.0276 | 0.8333 |
| GO:1903608 | protein localization to cytoplasmic stress granule | 7 | enriched | 1 | *SSB* | 0.0277 | 0.8333 |
| GO:2000234 | positive regulation of rRNA processing | 9 | enriched | 1 | *HEATR1* | 0.0271 | 0.8333 |
| GO:0016601 | Rac protein signal transduction | 21 | enriched | 2 | *FARP2, NCKAP1* | 0.0279 | 0.8355 |
| GO:0046579 | positive regulation of Ras protein signal transduction | 29 | depleted | 0 |  | 0.0281 | 0.8377 |
| GO:0044314 | protein K27-linked ubiquitination | 5 | depleted | 0 |  | 0.0282 | 0.8388 |
| GO:0097118 | neuroligin clustering involved in postsynaptic membrane assembly | 4 | depleted | 0 |  | 0.0285 | 0.8470 |
| GO:2001137 | positive regulation of endocytic recycling | 4 | enriched | 1 | *ACTN2* | 0.0286 | 0.8470 |
| GO:0035519 | protein K29-linked ubiquitination | 6 | depleted | 0 |  | 0.0289 | 0.8544 |
| GO:0048839 | inner ear development | 44 | depleted | 0 |  | 0.0301 | 0.8827 |
| GO:0048856 | anatomical structure development | 48 | enriched | 5 | *SPOCK3, IGF1R, SIX4, SIX5, SPOCK1* | 0.0300 | 0.8827 |
| GO:0071425 | hematopoietic stem cell proliferation | 14 | depleted | 0 |  | 0.0299 | 0.8827 |
| GO:1901030 | positive regulation of mitochondrial outer membrane permeabilization involved in apoptotic signaling pathway | 10 | enriched | 1 | *BAK1* | 0.0305 | 0.8922 |
| GO:0007512 | adult heart development | 13 | enriched | 2 | *CHD7, MNAT1* | 0.0309 | 0.9028 |
| GO:1990001 | inhibition of cysteine-type endopeptidase activity involved in apoptotic process | 7 | enriched | 1 | *BCL2L12* | 0.0312 | 0.9085 |
| GO:0035148 | tube formation | 10 | enriched | 1 | *ITGB1BP1* | 0.0313 | 0.9107 |
| GO:0002769 | natural killer cell inhibitory signaling pathway | 2 | enriched | 1 | *CLEC12B* | 0.0315 | 0.9109 |
| GO:0033563 | dorsal/ventral axon guidance | 3 | depleted | 0 |  | 0.0315 | 0.9109 |
| GO:2000601 | positive regulation of Arp2/3 complex-mediated actin nucleation | 8 | enriched | 1 | *NCKAP1* | 0.0316 | 0.9109 |
| GO:0007178 | transmembrane receptor protein serine/threonine kinase signaling pathway | 10 | enriched | 1 | *ACVR2B* | 0.0316 | 0.9110 |
| GO:0032259 | methylation | 54 | enriched | 3 | *COQ5, METTL5, NDUFAF5* | 0.0319 | 0.9150 |
| GO:0006304 | DNA modification | 2 | enriched | 1 | *DNTT* | 0.0321 | 0.9170 |
| GO:0034622 | cellular protein-containing complex assembly | 22 | depleted | 0 |  | 0.0320 | 0.9170 |
| GO:0022011 | myelination in peripheral nervous system | 17 | depleted | 0 |  | 0.0322 | 0.9196 |
| GO:0034465 | response to carbon monoxide | 3 | depleted | 0 |  | 0.0323 | 0.9196 |
| GO:0010224 | response to UV-B | 10 | enriched | 1 | *HMGN1* | 0.0327 | 0.9260 |
| GO:0070842 | aggresome assembly | 6 | depleted | 0 |  | 0.0327 | 0.9260 |
| GO:0008299 | isoprenoid biosynthetic process | 8 | enriched | 1 | *MVK* | 0.0329 | 0.9288 |
| GO:0010650 | positive regulation of cell communication by electrical coupling | 1 | depleted | 0 |  | 0.0334 | 0.9317 |
| GO:0060068 | vagina development | 10 | enriched | 1 | *BAK1* | 0.0332 | 0.9317 |
| GO:0070050 | neuron cellular homeostasis | 15 | depleted | 0 |  | 0.0333 | 0.9317 |
| GO:0072344 | rescue of stalled ribosome | 5 | enriched | 1 | *LTN1* | 0.0333 | 0.9317 |
| GO:0072660 | maintenance of protein location in plasma membrane | 1 | depleted | 0 |  | 0.0334 | 0.9317 |
| GO:1900827 | positive regulation of membrane depolarization during cardiac muscle cell action potential | 1 | depleted | 0 |  | 0.0334 | 0.9317 |
| GO:1904871 | positive regulation of protein localization to Cajal body | 9 | enriched | 1 | *CCT8* | 0.0335 | 0.9317 |
| GO:0014032 | neural crest cell development | 21 | depleted | 0 |  | 0.0341 | 0.9459 |
| GO:0050658 | RNA transport | 11 | enriched | 3 | *SIDT2, C14orf166, CKAP5* | 0.0343 | 0.9481 |
| GO:1902109 | negative regulation of mitochondrial membrane permeability involved in apoptotic process | 3 | enriched | 1 | *BNIP3* | 0.0343 | 0.9481 |
| GO:0060541 | respiratory system development | 3 | depleted | 0 |  | 0.0345 | 0.9490 |
| GO:2000114 | regulation of establishment of cell polarity | 18 | depleted | 0 |  | 0.0345 | 0.9490 |
| GO:0045087 | innate immune response | 394 | enriched | 14 | *DEFB115, DEFB116, DEFB118, DEFB119, DEFB121, DEFB123, DEFB124, AIM2, ALPK1, IFI16, LGR4, MARCO, NFKB2, PGLYRP1* | 0.0350 | 0.9572 |
| GO:0060294 | cilium movement involved in cell motility | 10 | enriched | 2 | *RSPH4A, RSPH6A* | 0.0350 | 0.9572 |
| GO:0071345 | cellular response to cytokine stimulus | 54 | depleted | 0 |  | 0.0351 | 0.9572 |
| GO:0033157 | regulation of intracellular protein transport | 7 | enriched | 1 | *PTPN1* | 0.0352 | 0.9581 |
| GO:0045839 | negative regulation of mitotic nuclear division | 8 | enriched | 1 | *TOM1L1* | 0.0355 | 0.9645 |
| GO:2000741 | positive regulation of mesenchymal stem cell differentiation | 4 | depleted | 0 |  | 0.0358 | 0.9710 |
| GO:0006903 | vesicle targeting | 8 | enriched | 1 | *CLASP2* | 0.0363 | 0.9742 |
| GO:0010907 | positive regulation of glucose metabolic process | 10 | enriched | 1 | *SLC45A3* | 0.0363 | 0.9742 |
| GO:0033623 | regulation of integrin activation | 3 | enriched | 1 | *FARP2* | 0.0362 | 0.9742 |
| GO:0090091 | positive regulation of extracellular matrix disassembly | 8 | enriched | 1 | *CLASP2* | 0.0363 | 0.9742 |
| GO:2000008 | regulation of protein localization to cell surface | 10 | enriched | 1 | *GBF1* | 0.0362 | 0.9742 |
| GO:1900738 | positive regulation of phospholipase C-activating G protein-coupled receptor signaling pathway | 4 | enriched | 1 | *F2* | 0.0365 | 0.9758 |
| GO:0018315 | molybdenum incorporation into molybdenum-molybdopterin complex | 1 | depleted | 0 |  | 0.0369 | 0.9797 |
| GO:0043586 | tongue development | 11 | enriched | 1 | *SIX4* | 0.0367 | 0.9797 |
| GO:0072579 | glycine receptor clustering | 1 | depleted | 0 |  | 0.0369 | 0.9797 |
| GO:2000050 | regulation of non-canonical Wnt signaling pathway | 3 | depleted | 0 |  | 0.0370 | 0.9814 |
| GO:2001224 | positive regulation of neuron migration | 14 | enriched | 2 | *SEMA3A, MDK* | 0.0371 | 0.9821 |
| GO:0010469 | regulation of signaling receptor activity | 18 | depleted | 0 |  | 0.0372 | 0.9823 |
| GO:0001886 | endothelial cell morphogenesis | 11 | enriched | 1 | *ID1* | 0.0434 | 0.9825 |
| GO:0001946 | lymphangiogenesis | 12 | enriched | 2 | *ACVR2B, CCBE1* | 0.0401 | 0.9825 |
| GO:0002093 | auditory receptor cell morphogenesis | 2 | depleted | 0 |  | 0.0493 | 0.9825 |
| GO:0002286 | T cell activation involved in immune response | 22 | enriched | 1 | *MDK* | 0.0474 | 0.9825 |
| GO:0003011 | involuntary skeletal muscle contraction | 1 | depleted | 0 |  | 0.0392 | 0.9825 |
| GO:0006091 | generation of precursor metabolites and energy | 49 | enriched | 3 | *COX4I2, COX6A1, XYLB* | 0.0376 | 0.9825 |
| GO:0006465 | signal peptide processing | 11 | enriched | 1 | *HM13* | 0.0403 | 0.9825 |
| GO:0006893 | Golgi to plasma membrane transport | 20 | depleted | 0 |  | 0.0381 | 0.9825 |
| GO:0007064 | mitotic sister chromatid cohesion | 10 | enriched | 1 | *PDS5A* | 0.0424 | 0.9825 |
| GO:0008637 | apoptotic mitochondrial changes | 18 | enriched | 2 | *BCL2L1, NDUFS1* | 0.0427 | 0.9825 |
| GO:0010606 | positive regulation of cytoplasmic mRNA processing body assembly | 6 | enriched | 1 | *CNOT6* | 0.0373 | 0.9825 |
| GO:0010838 | positive regulation of keratinocyte proliferation | 14 | enriched | 2 | *MDK, STXBP4* | 0.0411 | 0.9825 |
| GO:0010841 | positive regulation of circadian sleep/wake cycle, wakefulness | 3 | depleted | 0 |  | 0.0475 | 0.9825 |
| GO:0014050 | negative regulation of glutamate secretion | 5 | depleted | 0 |  | 0.0450 | 0.9825 |
| GO:0018095 | protein polyglutamylation | 11 | enriched | 1 | *TTLL5* | 0.0422 | 0.9825 |
| GO:0030238 | male sex determination | 12 | enriched | 1 | *SIX4* | 0.0484 | 0.9825 |
| GO:0031124 | mRNA 3'-end processing | 59 | enriched | 3 | *CPSF3, SRSF9, SYMPK* | 0.0429 | 0.9825 |
| GO:0032230 | positive regulation of synaptic transmission, GABAergic | 12 | depleted | 0 |  | 0.0417 | 0.9825 |
| GO:0032479 | regulation of type I interferon production | 12 | enriched | 1 | *IRF3* | 0.0430 | 0.9825 |
| GO:0032886 | regulation of microtubule-based process | 9 | enriched | 2 | *CLASP2, MACF1* | 0.0430 | 0.9825 |
| GO:0032963 | collagen metabolic process | 11 | enriched | 1 | *ID1* | 0.0469 | 0.9825 |
| GO:0033600 | negative regulation of mammary gland epithelial cell proliferation | 4 | depleted | 0 |  | 0.0444 | 0.9825 |
| GO:0034384 | high-density lipoprotein particle clearance | 11 | enriched | 1 | *HDLBP* | 0.0476 | 0.9825 |
| GO:0035735 | intraciliary transport involved in cilium assembly | 39 | enriched | 3 | *IFT43, DYNC2LI1, DYNLL1* | 0.0448 | 0.9825 |
| GO:0043030 | regulation of macrophage activation | 9 | enriched | 2 | *RORA, MYO18A* | 0.0395 | 0.9825 |
| GO:0043604 | amide biosynthetic process | 1 | enriched | 1 | *PM20D1* | 0.0436 | 0.9825 |
| GO:0043605 | cellular amide catabolic process | 1 | enriched | 1 | *PM20D1* | 0.0436 | 0.9825 |
| GO:0044782 | cilium organization | 10 | enriched | 1 | *ROPN1B* | 0.0377 | 0.9825 |
| GO:0044804 | autophagy of nucleus | 10 | enriched | 1 | *ATG13* | 0.0396 | 0.9825 |
| GO:0044828 | negative regulation by host of viral genome replication | 6 | depleted | 0 |  | 0.0411 | 0.9825 |
| GO:0044849 | estrous cycle | 16 | enriched | 2 | *ETS1, MDK* | 0.0437 | 0.9825 |
| GO:0045655 | regulation of monocyte differentiation | 2 | depleted | 0 |  | 0.0382 | 0.9825 |
| GO:0045920 | negative regulation of exocytosis | 5 | depleted | 0 |  | 0.0393 | 0.9825 |
| GO:0048169 | regulation of long-term neuronal synaptic plasticity | 21 | enriched | 3 | *CAMK2B, NETO1, SYNGAP1* | 0.0413 | 0.9825 |
| GO:0048699 | generation of neurons | 7 | enriched | 2 | *CIT, SIX4* | 0.0399 | 0.9825 |
| GO:0048935 | peripheral nervous system neuron development | 10 | depleted | 0 |  | 0.0429 | 0.9825 |
| GO:0050803 | regulation of synapse structure or activity | 11 | enriched | 1 | *SYNGAP1* | 0.0459 | 0.9825 |
| GO:0051292 | nuclear pore complex assembly | 10 | enriched | 2 | *AHCTF1, RTN4* | 0.0455 | 0.9825 |
| GO:0051894 | positive regulation of focal adhesion assembly | 25 | enriched | 2 | *ITGB1BP1, TSC1* | 0.0424 | 0.9825 |
| GO:0060074 | synapse maturation | 11 | enriched | 1 | *PFN1* | 0.0404 | 0.9825 |
| GO:0060425 | lung morphogenesis | 12 | enriched | 1 | *ID1* | 0.0450 | 0.9825 |
| GO:0061589 | calcium activated phosphatidylserine scrambling | 4 | enriched | 1 | *ANO7* | 0.0379 | 0.9825 |
| GO:0065004 | protein-DNA complex assembly | 5 | enriched | 1 | *TCF4* | 0.0475 | 0.9825 |
| GO:0071481 | cellular response to X-ray | 10 | enriched | 1 | *NUCKS1* | 0.0377 | 0.9825 |
| GO:0071657 | positive regulation of granulocyte colony-stimulating factor production | 3 | enriched | 1 | *TSLP* | 0.0387 | 0.9825 |
| GO:0071788 | endoplasmic reticulum tubular network maintenance | 1 | enriched | 1 | *KIAA1715* | 0.0380 | 0.9825 |
| GO:0071911 | synchronous neurotransmitter secretion | 1 | depleted | 0 |  | 0.0482 | 0.9825 |
| GO:0072619 | interleukin-21 secretion | 1 | depleted | 0 |  | 0.0422 | 0.9825 |
| GO:0090630 | activation of GTPase activity | 92 | enriched | 7 | *ARHGAP42, GPR65, TBC1D29, TBC1D7, TBC1D8, TSC1, USP6* | 0.0437 | 0.9825 |
| GO:0097284 | hepatocyte apoptotic process | 11 | enriched | 1 | *BCL2L1* | 0.0412 | 0.9825 |
| GO:0098746 | fast, calcium ion-dependent exocytosis of neurotransmitter | 1 | depleted | 0 |  | 0.0482 | 0.9825 |
| GO:0098789 | pre-mRNA cleavage required for polyadenylation | 12 | depleted | 0 |  | 0.0375 | 0.9825 |
| GO:0120163 | negative regulation of cold-induced thermogenesis | 46 | enriched | 4 | *ACVR2B, ADAM17, ID1, LGR4* | 0.0382 | 0.9825 |
| GO:1900118 | negative regulation of execution phase of apoptosis | 14 | enriched | 1 | *BCL2L1* | 0.0379 | 0.9825 |
| GO:1900165 | negative regulation of interleukin-6 secretion | 10 | depleted | 0 |  | 0.0453 | 0.9825 |
| GO:1900407 | regulation of cellular response to oxidative stress | 5 | depleted | 0 |  | 0.0400 | 0.9825 |
| GO:1901256 | regulation of macrophage colony-stimulating factor production | 1 | depleted | 0 |  | 0.0422 | 0.9825 |
| GO:1902803 | regulation of synaptic vesicle transport | 3 | depleted | 0 |  | 0.0455 | 0.9825 |
| GO:1903028 | positive regulation of opsonization | 1 | enriched | 1 | *MYO18A* | 0.0394 | 0.9825 |
| GO:1903214 | regulation of protein targeting to mitochondrion | 7 | depleted | 0 |  | 0.0443 | 0.9825 |
| GO:1903235 | positive regulation of calcium ion-dependent exocytosis of neurotransmitter | 1 | depleted | 0 |  | 0.0482 | 0.9825 |
| GO:1903542 | negative regulation of exosomal secretion | 3 | depleted | 0 |  | 0.0412 | 0.9825 |
| GO:1904851 | positive regulation of establishment of protein localization to telomere | 10 | enriched | 1 | *CCT8* | 0.0421 | 0.9825 |
| GO:1990145 | maintenance of translational fidelity | 2 | depleted | 0 |  | 0.0377 | 0.9825 |
| GO:2000275 | regulation of oxidative phosphorylation uncoupler activity | 1 | enriched | 1 | *PM20D1* | 0.0436 | 0.9825 |
| GO:2000821 | regulation of grooming behavior | 5 | depleted | 0 |  | 0.0495 | 0.9825 |
| GO:2001182 | regulation of interleukin-12 secretion | 1 | depleted | 0 |  | 0.0422 | 0.9825 |
| GO:2001258 | negative regulation of cation channel activity | 3 | depleted | 0 |  | 0.0434 | 0.9825 |
