# Supplementary material for: Adolescent anxiety and pain problems: A joint, genome-wide investigation and pathway-based analysis
Source: PLoS One. 2023 May 5;18(5):e0285263. doi: 10.1371/journal.pone.0285263 (PMC10162554; doi:10.1371/journal.pone.0285263)
Supplement: S4 Table — (DOCX) [file pone.0285263.s004.docx]

| **S4 Table. Overlapping enriched pathways between QNTS_Mean Pain and QNTS_Mean Anxiety (uncorrected p-value < 0.05).** | | | | | | | | | | |
| --- | --- | --- | --- | --- | --- | --- | --- | --- | --- | --- |
| **GO set ID** | **Description** | **Pathway size (nr. of genes)** | **QNTS_Mean Pain** | | | | **QNTS_Mean Anxiety** | | | |
|  |  |  | **Enriched Genes (nr.)** | **Genes** | ***p-value*** | **FDR** | **Enriched Genes (nr.)** | **Genes** | ***p-value*** | **FDR** |
| GO:0001946 | lymphangiogenesis | 12 | 1 | *ACVR2B* | 0.0246 | 0.9871 | 2 | *ACVR2B, CCBE1* | 0.0401 | 0.9825 |
| GO:0006379 | mRNA cleavage | 8 | 1 | *ZNRD1* | 0.0082 | 0.6782 | 1 | *CPSF3* | 0.0189 | 0.6997 |
| GO:0006417 | regulation of translation | 63 | 4 | *GRM5, DDX25, EIF4E1B, GCN1L1* | 0.0182 | 0.9871 | 4 | *METAP1, CNOT6, FOXO3, TSC1* | 0.0251 | 0.7985 |
| GO:0006450 | regulation of translational fidelity | 6 | 1 | *GATC* | 0.0030 | 0.4206 | 1 | *GATC* | 0.0085 | 0.4943 |
| GO:0007178 | transmembrane receptor protein serine/threonine kinase signaling pathway | 10 | 1 | *ACVR2B* | 0.0137 | 0.8989 | 1 | *ACVR2B* | 0.0316 | 0.9110 |
| GO:0010038 | response to metal ion | 13 | 2 | *GPHN, SNCB* | 0.0054 | 0.5386 | 2 | *CUTA, MT1A* | 0.0169 | 0.6716 |
| GO:0010830 | regulation of myotube differentiation | 5 | 1 | *DMPK* | 0.0017 | 0.3522 | 1 | *DMPK* | 0.0034 | 0.3161 |
| GO:0030330 | DNA damage response, signal transduction by p53 class mediator | 16 | 2 | *MYO6, TRIAP1* | 0.0001 | 0.1259 | 2 | *MYO6, TRIAP1* | 0.0020 | 0.2709 |
| GO:0031124 | mRNA 3'-end processing | 59 | 3 | *SRSF9, RPRD1A, SYMPK* | 0.0077 | 0.6629 | 3 | *CPSF3, SRSF9, SYMPK* | 0.0429 | 0.9825 |
| GO:0032927 | positive regulation of activin receptor signaling pathway | 7 | 1 | *ACVR2B* | 0.0048 | 0.5131 | 1 | *ACVR2B* | 0.0145 | 0.6318 |
| GO:0035265 | organ growth | 8 | 1 | *ACVR2B* | 0.0072 | 0.6432 | 1 | *ACVR2B* | 0.0174 | 0.6819 |
| GO:0038018 | Wnt receptor catabolic process | 2 | 1 | *ZNRF3* | 0.0017 | 0.3522 | 1 | *ZNRF3* | 0.0042 | 0.3543 |
| GO:0043279 | response to alkaloid | 6 | 1 | *SRSF9* | 0.0042 | 0.4906 | 2 | *BCHE, SRSF9* | <0.0001 | 0.0157 |
| GO:0043666 | regulation of phosphoprotein phosphatase activity | 41 | 3 | *PPP2R1A, DMPK, PPP6R2* | 0.0093 | 0.7343 | 4 | *DMPK, PPP1R7, PPP2R2D, TSC1* | 0.0011 | 0.2530 |
| GO:0044773 | mitotic DNA damage checkpoint | 3 | 1 | *STK33* | 0.0408 | 0.9871 | 2 | *CHEK2, STK33* | 0.0054 | 0.4060 |
| GO:0048617 | embryonic foregut morphogenesis | 9 | 1 | *ACVR2B* | 0.0111 | 0.7983 | 1 | *ACVR2B* | 0.0277 | 0.8333 |
| GO:0051046 | regulation of secretion | 2 | 1 | *MYO6* | 0.0006 | 0.2503 | 1 | *MYO6* | 0.0025 | 0.3025 |
| GO:0051823 | regulation of synapse structural plasticity | 5 | 2 | *CAMK2B, DMPK* | 0.0010 | 0.3168 | 2 | *CAMK2B, DMPK* | 0.0024 | 0.3004 |
| GO:0051895 | negative regulation of focal adhesion assembly | 18 | 2 | *APOD, THBS1* | 0.0497 | 0.9871 | 3 | *CLASP2, ITGB1BP1, MMP14* | 0.0004 | 0.2016 |
| GO:0060173 | limb development | 31 | 3 | *RC3H2, SMOC1, ZNRF3* | 0.0081 | 0.6752 | 3 | *ZNRF3, CHD7, KIAA1715* | 0.0246 | 0.7955 |
| GO:0060294 | cilium movement involved in cell motility | 10 | 2 | *RSPH6A, TEKT5* | 0.0035 | 0.4492 | 2 | *RSPH4A, RSPH6A* | 0.0350 | 0.9572 |
| GO:0060836 | lymphatic endothelial cell differentiation | 6 | 1 | *ACVR2B* | 0.0039 | 0.4795 | 1 | *ACVR2B* | 0.0085 | 0.4943 |
| GO:0060840 | artery development | 7 | 1 | *ACVR2B* | 0.0053 | 0.5366 | 1 | *ACVR2B* | 0.0127 | 0.6039 |
| GO:0060841 | venous blood vessel development | 4 | 1 | *ACVR2B* | 0.0013 | 0.3316 | 1 | *ACVR2B* | 0.0030 | 0.3087 |
| GO:0061298 | retina vasculature development in camera-type eye | 7 | 1 | *ACVR2B* | 0.0054 | 0.5386 | 1 | *ACVR2B* | 0.0130 | 0.6046 |
| GO:0070681 | glutaminyl-tRNAGln biosynthesis via transamidation | 3 | 1 | *GATC* | 0.0006 | 0.2503 | 1 | *GATC* | 0.0016 | 0.2530 |
| GO:0097035 | regulation of membrane lipid distribution | 5 | 1 | *TRIAP1* | 0.0091 | 0.7282 | 1 | *TRIAP1* | 0.0028 | 0.3087 |
| GO:0120163 | negative regulation of cold-induced thermogenesis | 46 | 2 | *ACVR2B, ARRDC3* | 0.0292 | 0.9871 | 4 | *ACVR2B, ADAM17, ID1, LGR4* | 0.0382 | 0.9825 |
| GO:1902723 | negative regulation of skeletal muscle satellite cell proliferation | 4 | 1 | *SIX5* | 0.0024 | 0.3724 | 1 | *SIX5* | 0.0031 | 0.3087 |
| GO:1903373 | positive regulation of endoplasmic reticulum tubular network organization | 4 | 1 | *RAB3GAP2* | 0.0020 | 0.3522 | 1 | *KIAA1715* | 0.0100 | 0.5438 |
| GO:1903441 | protein localization to ciliary membrane | 5 | 1 | *ARL3* | 0.0020 | 0.3522 | 1 | *RAB7L1* | 0.0052 | 0.3982 |
| GO:1990001 | inhibition of cysteine-type endopeptidase activity involved in apoptotic process | 7 | 1 | *BIRC7* | 0.0088 | 0.7116 | 1 | *BCL2L12* | 0.0312 | 0.9085 |
| GO:2000051 | negative regulation of non-canonical Wnt signaling pathway | 4 | 1 | *ZNRF3* | 0.0123 | 0.8442 | 1 | *ZNRF3* | 0.0190 | 0.7011 |
| GO:2000095 | regulation of Wnt signaling pathway, planar cell polarity pathway | 4 | 1 | *ZNRF3* | 0.0055 | 0.5417 | 1 | *ZNRF3* | 0.0117 | 0.5856 |
| GO:2000582 | positive regulation of ATP-dependent microtubule motor activity, plus-end-directed | 5 | 1 | *DYNLL1* | 0.0019 | 0.3522 | 1 | *DYNLL1* | 0.0049 | 0.3936 |
| GO:2001140 | positive regulation of phospholipid transport | 4 | 1 | *TRIAP1* | 0.0019 | 0.3522 | 1 | *TRIAP1* | 0.0028 | 0.3087 |
| GO:2001184 | positive regulation of interleukin-12 secretion | 4 | 1 | *MAPK11* | 0.0018 | 0.3522 | 1 | *MDK* | 0.0028 | 0.3087 |
