# Supplementary material for: Adolescent anxiety and pain problems: A joint, genome-wide investigation and pathway-based analysis
Source: PLoS One. 2023 May 5;18(5):e0285263. doi: 10.1371/journal.pone.0285263 (PMC10162554; doi:10.1371/journal.pone.0285263)
Supplement: S5 Table — (DOCX) [file pone.0285263.s005.docx]

| **S5a Table. Top associations (*p*<1x10^-5^) of the genome-wide analysis** | | | | | | | |
| --- | --- | --- | --- | --- | --- | --- | --- |
| **QLSDC_Mean Pain** | | | | | | | |
| **Chromosome** | **SNP** | **Position (hg37)** | **Gene** | **Allele** | **Nr. of subjects** | **β** | **p-value** |
| 1 | rs57741754 | 41869335 | Intergenic | C | 749 | 0,1414 | 2,31E-06 |
| 1 | rs11588706 | 41864714 | Intergenic | C | 753 | 0,1397 | 2,73E-06 |
| 1 | rs6683743 | 41863559 | Intergenic | A | 754 | 0,1376 | 3,41E-06 |
| 1 | rs6660083 | 41863560 | Intergenic | G | 754 | 0,1376 | 3,41E-06 |
| 1 | rs66471067 | 41869962 | Intergenic | T | 749 | 0,1374 | 4,88E-06 |
| 1 | rs2997333 | 41863072 | Intergenic | C | 754 | 0,1346 | 5,24E-06 |
| 1 | rs2997334 | 41863323 | Intergenic | G | 754 | 0,1346 | 5,24E-06 |
| 1 | rs2996013 | 41863990 | Intergenic | G | 754 | 0,1346 | 5,24E-06 |
| 1 | rs11210026 | 41864971 | Intergenic | C | 754 | 0,1346 | 5,24E-06 |
| 1 | rs7536490 | 41866315 | Intergenic | G | 752 | 0,1354 | 6,58E-06 |
| 1 | rs11588202 | 41867481 | Intergenic | T | 751 | 0,135 | 7,28E-06 |
| 1 | rs3845572 | 41864364 | Intergenic | T | 754 | 0,1317 | 8,06E-06 |
| 1 | rs6600375 | 41861897 | Intergenic | T | 754 | 0,1311 | 9,44E-06 |
| 4 | rs6826379 | 182646272 | *TENM3* | A | 746 | 0,0947 | 9,52E-06 |
| 7 | rs2365575 | 140789753 | *TMEM178B* | G | 750 | 0,1422 | 3,16E-06 |
| 7 | rs7778658 | 140791306 | *TMEM178B* | A | 750 | 0,1422 | 3,16E-06 |
| 7 | rs970599 | 140793121 | *TMEM178B* | C | 750 | 0,1422 | 3,16E-06 |
| 7 | rs55949610 | 140798344 | *TMEM178B* | C | 753 | 0,1413 | 3,24E-06 |
| 7 | rs9640323 | 140795298 | *TMEM178B* | G | 752 | 0,1412 | 3,40E-06 |
| 7 | rs7806026 | 140783382 | *TMEM178B* | G | 749 | 0,1407 | 4,15E-06 |
| 7 | rs560981 | 140786681 | *TMEM178B* | T | 749 | 0,1407 | 4,15E-06 |
| 7 | rs55971084 | 140791320 | *TMEM178B* | A | 751 | 0,1706 | 4,44E-06 |
| 7 | rs17630683 | 140800262 | *TMEM178B* | G | 750 | 0,1710 | 6,10E-06 |
| 7 | rs7805034 | 140799480 | *TMEM178B* | A | 754 | 0,1331 | 7,10E-06 |
| 11 | rs1564989 | 24796875 | *LUZP2* | G | 747 | 0,1193 | 3,57E-06 |
| 11 | rs1564988 | 24796958 | *LUZP2* | G | 747 | 0,1193 | 3,57E-06 |
| 11 | rs1466602 | 24795946 | *LUZP2* | T | 746 | 0,1163 | 7,12E-06 |

| **S5b Table. Top associations (*p*<1x10^-5^) of the genome-wide analysis** | | | | | | | |
| --- | --- | --- | --- | --- | --- | --- | --- |
| **QLSDC_Mean Anxiety** | | | | | | | |
| **Chromosome** | **SNP** | **Position (hg37)** | **Gene** | **Allele** | **Nr. of subjects** | **β** | **p-value** |
| 1 | rs57741754 | 41869335 | Intergenic | C | 749 | 0,0664 | 1,57E-06 |
| 1 | rs11588706 | 41864714 | Intergenic | C | 753 | 0,0660 | 1,79E-06 |
| 1 | rs66471067 | 41869962 | Intergenic | T | 749 | 0,0653 | 2,45E-06 |
| 1 | rs7536490 | 41866315 | Intergenic | G | 752 | 0,0647 | 3,00E-06 |
| 1 | rs11588202 | 41867481 | Intergenic | T | 751 | 0,0646 | 3,00E-06 |
| 1 | rs6683743 | 41863559 | Intergenic | A | 754 | 0,0640 | 3,01E-06 |
| 1 | rs6660083 | 41863560 | Intergenic | G | 754 | 0,0640 | 3,01E-06 |
| 1 | rs2997333 | 41863072 | Intergenic | C | 754 | 0,0628 | 4,55E-06 |
| 1 | rs2997334 | 41863323 | Intergenic | G | 754 | 0,0628 | 4,55E-06 |
| 1 | rs2996013 | 41863990 | Intergenic | G | 754 | 0,0628 | 4,55E-06 |
| 1 | rs11210026 | 41864971 | Intergenic | C | 754 | 0,0628 | 4,55E-06 |
| 1 | rs6600375 | 41861897 | Intergenic | T | 754 | 0,0614 | 7,82E-06 |
| 1 | rs3845572 | 41864364 | Intergenic | T | 754 | 0,0613 | 8,01E-06 |
| 4 | rs12642634 | 146985627 | Intergenic | G | 754 | -0,0507 | 9,51E-06 |
| 5 | rs79899489 | 154912806 | Intergenic | G | 747 | 0,1603 | 3,97E-06 |
| 5 | rs1984562 | 154915985 | Intergenic | A | 747 | 0,1603 | 3,97E-06 |
| 5 | rs74891157 | 154926844 | Intergenic | G | 747 | 0,1603 | 3,97E-06 |
| 14 | rs710040 | 62125233 | Intergenic | T | 747 | 0,2515 | 4,74E-07 |
| 14 | rs4406966 | 22700656 | Intergenic | T | 751 | -0,0440 | 8,37E-06 |
| 14 | rs2331596 | 22700662 | Intergenic | G | 751 | -0,0440 | 8,37E-06 |
| 14 | rs7151220 | 22704222 | Intergenic | G | 747 | -0,0439 | 8,39E-06 |
