# Supplementary material for: Adolescent anxiety and pain problems: A joint, genome-wide investigation and pathway-based analysis
Source: PLoS One. 2023 May 5;18(5):e0285263. doi: 10.1371/journal.pone.0285263 (PMC10162554; doi:10.1371/journal.pone.0285263)
Supplement: S6 Table — (DOCX) [file pone.0285263.s006.docx]

| **S6a Table. Results of the pathway-based analysis of QLSCD_Mean Pain and QLSCD_Mean Anxiety (uncorrected p-value < 0.05).** | | | | | | | |
| --- | --- | --- | --- | --- | --- | --- | --- |
| **QLSCD_Mean Pain** | | | | | | | |
| **GO set ID** | **Description** | **Pathway size (nr of genes)** | **Status** | **Significant Genes (nr of)** | **Genes** | ***p-value*** | **FDR** |
| GO:0007156 | homophilic cell adhesion via plasma membrane adhesion molecules | 162 | enriched | 27 | *CDH13, ROBO2, DCHS2, IGSF21, PCDH7, PCDHGA1, PCDHGA10, PCDHGA11, PCDHGA12, PCDHGA2, PCDHGA3, PCDHGA4, PCDHGA5, PCDHGA6, PCDHGA7, PCDHGA8, PCDHGA9, PCDHGB1, PCDHGB2, PCDHGB3, PCDHGB4, PCDHGB6, PCDHGB7, PCDHGC3, PCDHGC4, PCDHGC5, PVRL2* | <0.0001 | 0.0012 |
| GO:0042921 | glucocorticoid receptor signaling pathway | 5 | enriched | 2 | *ARID1A, NEDD4* | <0.0001 | 0.0249 |
| GO:0050774 | negative regulation of dendrite morphogenesis | 9 | enriched | 2 | *NFATC4, GORASP1* | <0.0001 | 0.0417 |
| GO:0061951 | establishment of protein localization to plasma membrane | 4 | enriched | 2 | *GORASP1, MLLT4* | <0.0001 | 0.0936 |
| GO:0007076 | mitotic chromosome condensation | 17 | enriched | 3 | *AKAP8, AKAP8L, SMC4* | <0.0001 | 0.1073 |
| GO:0007155 | cell adhesion | 447 | enriched | 43 | *BCAM, TINAG, ADAM12, CCR3, CHL1, COL28A1, COL8A1, EPHA3, FAM65B, FER, IBSP, ICAM3, ITGAL, ITGB8, MLLT4, NTM, PARD3, PCDH7, PCDHGA1, PCDHGA10, PCDHGA11, PCDHGA12, PCDHGA2, PCDHGA3, PCDHGA4, PCDHGA5, PCDHGA6, PCDHGA7, PCDHGA8, PCDHGA9, PCDHGB1, PCDHGB2, PCDHGB3, PCDHGB4, PCDHGB6, PCDHGB7, PCDHGC3, PCDHGC4, PCDHGC5, PLXNC1, SELP, STAB2, WISP2* | <0.0001 | 0.1823 |
| GO:0046626 | regulation of insulin receptor signaling pathway | 12 | enriched | 3 | *CCND3, PIK3R1, SIK2* | 0.0001 | 0.2007 |
| GO:0045333 | cellular respiration | 17 | enriched | 3 | *NFATC4, CYP1A2, NDUFS1* | 0.0001 | 0.2025 |
| GO:0050808 | synapse organization | 46 | enriched | 6 | *PPFIA2, C1QA, PCDHGC3, PCDHGC4, PCDHGC5, PPFIBP2* | 0.0001 | 0.2025 |
| GO:0032053 | ciliary basal body organization | 3 | enriched | 2 | *CROCC, RTTN* | 0.0001 | 0.2061 |
| GO:0002503 | peptide antigen assembly with MHC class II protein complex | 3 | enriched | 1 | *HLA-DRA* | 0.0005 | 0.2802 |
| GO:0003205 | cardiac chamber development | 3 | enriched | 1 | *ARID1A* | 0.0010 | 0.2802 |
| GO:0003408 | optic cup formation involved in camera-type eye development | 3 | enriched | 1 | *ARID1A* | 0.0010 | 0.2802 |
| GO:0006974 | cellular response to DNA damage stimulus | 231 | enriched | 16 | *APLF, BRD4, BRE, CTC1, FBXO31, FOXO1, HUS1, IMMP2L, OARD1, RFWD3, SGK1, TOPBP1, TP53BP1, USP16, ZBTB38, ZNF771* | 0.0006 | 0.2802 |
| GO:0007020 | microtubule nucleation | 17 | enriched | 2 | *AKAP9, TUBGCP4* | 0.0008 | 0.2802 |
| GO:0007175 | negative regulation of epidermal growth factor-activated receptor activity | 14 | enriched | 3 | *CBLB, CBLC, SOCS5* | 0.0007 | 0.2802 |
| GO:0010569 | regulation of double-strand break repair via homologous recombination | 18 | enriched | 3 | *SETD2, TEX15, ZNF365* | 0.0009 | 0.2802 |
| GO:0010793 | regulation of mRNA export from nucleus | 6 | enriched | 2 | *AKAP8L, SETD2* | 0.0003 | 0.2802 |
| GO:0015711 | organic anion transport | 14 | enriched | 4 | *SLC22A10, SLC22A24, SLC22A25, SLC22A9* | 0.0004 | 0.2802 |
| GO:0016259 | selenocysteine metabolic process | 3 | enriched | 1 | *SEPHS2* | 0.0008 | 0.2802 |
| GO:0016260 | selenocysteine biosynthetic process | 2 | enriched | 1 | *SEPHS2* | 0.0005 | 0.2802 |
| GO:0030003 | cellular cation homeostasis | 2 | enriched | 1 | *ATP13A2* | 0.0006 | 0.2802 |
| GO:0031052 | chromosome breakage | 2 | enriched | 1 | *RFWD3* | 0.0009 | 0.2802 |
| GO:0033127 | regulation of histone phosphorylation | 2 | enriched | 2 | *AKAP8, AKAP8L* | 0.0006 | 0.2802 |
| GO:0034644 | cellular response to UV | 49 | enriched | 4 | *NFATC4, AURKB, NEDD4, PIK3R1* | 0.0003 | 0.2802 |
| GO:0035350 | FAD transmembrane transport | 2 | enriched | 1 | *SLC25A32* | 0.0007 | 0.2802 |
| GO:0035441 | cell migration involved in vasculogenesis | 2 | enriched | 1 | *SETD2* | 0.0009 | 0.2802 |
| GO:0042262 | DNA protection | 5 | enriched | 1 | *DCTPP1* | 0.0011 | 0.2802 |
| GO:0044839 | cell cycle G2/M phase transition | 2 | enriched | 2 | *AKAP8, AKAP8L* | 0.0006 | 0.2802 |
| GO:0048050 | post-embryonic eye morphogenesis | 3 | enriched | 1 | *MFAP2* | 0.0010 | 0.2802 |
| GO:0048332 | mesoderm morphogenesis | 2 | enriched | 1 | *SETD2* | 0.0008 | 0.2802 |
| GO:0048814 | regulation of dendrite morphogenesis | 21 | enriched | 4 | *KNDC1, NEDD4, NEDD4L, RAP2A* | 0.0008 | 0.2802 |
| GO:0051729 | germline cell cycle switching, mitotic to meiotic cell cycle | 2 | enriched | 1 | *YTHDC2* | 0.0008 | 0.2802 |
| GO:0052746 | inositol phosphorylation | 2 | enriched | 1 | *IPPK* | 0.0009 | 0.2802 |
| GO:0060491 | regulation of cell projection assembly | 3 | enriched | 1 | *FAM110C* | 0.0007 | 0.2802 |
| GO:0060977 | coronary vasculature morphogenesis | 3 | enriched | 2 | *SGCD, SETD2* | 0.0004 | 0.2802 |
| GO:0071344 | diphosphate metabolic process | 2 | enriched | 1 | *PPA1* | 0.0008 | 0.2802 |
| GO:0072393 | microtubule anchoring at microtubule organizing center | 2 | enriched | 1 | *BICD2* | 0.0010 | 0.2802 |
| GO:0090177 | establishment of planar polarity involved in neural tube closure | 2 | enriched | 1 | *CTHRC1* | 0.0006 | 0.2802 |
| GO:0097198 | histone H3-K36 trimethylation | 2 | enriched | 1 | *SETD2* | 0.0008 | 0.2802 |
| GO:1902775 | mitochondrial large ribosomal subunit assembly | 3 | enriched | 1 | *FASTKD2* | 0.0008 | 0.2802 |
| GO:1902850 | microtubule cytoskeleton organization involved in mitosis | 2 | enriched | 1 | *SETD2* | 0.0009 | 0.2802 |
| GO:1904874 | positive regulation of telomerase RNA localization to Cajal body | 15 | enriched | 2 | *CCT6A, NAF1* | 0.0004 | 0.2802 |
| GO:1905037 | autophagosome organization | 3 | enriched | 1 | *ATP13A2* | 0.0009 | 0.2802 |
| GO:1905165 | regulation of lysosomal protein catabolic process | 2 | enriched | 1 | *ATP13A2* | 0.0006 | 0.2802 |
| GO:1990575 | mitochondrial L-ornithine transmembrane transport | 3 | enriched | 1 | *SLC25A15* | 0.0006 | 0.2802 |
| GO:2000143 | negative regulation of DNA-templated transcription, initiation | 2 | enriched | 1 | *MORC1* | 0.0009 | 0.2802 |
| GO:2000297 | negative regulation of synapse maturation | 3 | enriched | 1 | *NFATC4* | 0.0008 | 0.2802 |
| GO:0001704 | formation of primary germ layer | 3 | enriched | 1 | *ARID1A* | 0.0011 | 0.2838 |
| GO:0008630 | intrinsic apoptotic signaling pathway in response to DNA damage | 45 | enriched | 3 | *NFATC4, PIK3R1, SFN* | 0.0013 | 0.3093 |
| GO:0051987 | positive regulation of attachment of spindle microtubules to kinetochore | 3 | enriched | 1 | *RCC2* | 0.0013 | 0.3093 |
| GO:1901407 | regulation of phosphorylation of RNA polymerase II C-terminal domain | 2 | enriched | 1 | *BRD4* | 0.0013 | 0.3093 |
| GO:1905123 | regulation of glucosylceramidase activity | 2 | enriched | 1 | *ATP13A2* | 0.0012 | 0.3093 |
| GO:2001255 | positive regulation of histone H3-K36 trimethylation | 3 | enriched | 1 | *BRD4* | 0.0013 | 0.3093 |
| GO:0043551 | regulation of phosphatidylinositol 3-kinase activity | 17 | enriched | 2 | *PIK3R1, SOCS5* | 0.0014 | 0.3272 |
| GO:0002553 | histamine secretion by mast cell | 3 | enriched | 1 | *SNAP23* | 0.0016 | 0.3451 |
| GO:0034728 | nucleosome organization | 3 | enriched | 1 | *SETD2* | 0.0016 | 0.3451 |
| GO:0070988 | demethylation | 4 | enriched | 2 | *CYP1A1, CYP51A1* | 0.0016 | 0.3451 |
| GO:1900037 | regulation of cellular response to hypoxia | 4 | enriched | 1 | *CHCHD2* | 0.0016 | 0.3451 |
| GO:0000454 | snoRNA guided rRNA pseudouridine synthesis | 3 | enriched | 1 | *NAF1* | 0.0021 | 0.3477 |
| GO:0006928 | movement of cell or subcellular component | 4 | enriched | 1 | *SPHKAP* | 0.0019 | 0.3477 |
| GO:0016344 | meiotic chromosome movement towards spindle pole | 2 | enriched | 1 | *ACTR3* | 0.0021 | 0.3477 |
| GO:0016556 | mRNA modification | 3 | enriched | 1 | *APOBEC2* | 0.0020 | 0.3477 |
| GO:0031065 | positive regulation of histone deacetylation | 11 | enriched | 2 | *AKAP8, AKAP8L* | 0.0018 | 0.3477 |
| GO:0032211 | negative regulation of telomere maintenance via telomerase | 20 | enriched | 2 | *CTC1, PINX1* | 0.0020 | 0.3477 |
| GO:0033206 | meiotic cytokinesis | 2 | enriched | 1 | *ACTR3* | 0.0021 | 0.3477 |
| GO:0035879 | plasma membrane lactate transport | 3 | enriched | 1 | *SLC16A1* | 0.0020 | 0.3477 |
| GO:0040020 | regulation of meiotic nuclear division | 5 | enriched | 2 | *GPR3, PDE3A* | 0.0017 | 0.3477 |
| GO:0040029 | regulation of gene expression, epigenetic | 14 | enriched | 2 | *HDAC5, MORC1* | 0.0019 | 0.3477 |
| GO:0042766 | nucleosome mobilization | 4 | enriched | 1 | *ARID1A* | 0.0021 | 0.3477 |
| GO:0050890 | cognition | 45 | enriched | 4 | *TUSC3, CHL1, GPR155, HLA-DRA* | 0.0020 | 0.3477 |
| GO:0055069 | zinc ion homeostasis | 5 | enriched | 2 | *ATP13A2, PARK2* | 0.0018 | 0.3477 |
| GO:0097676 | histone H3-K36 dimethylation | 4 | enriched | 1 | *SETD2* | 0.0019 | 0.3477 |
| GO:1904714 | regulation of chaperone-mediated autophagy | 5 | enriched | 1 | *ATP13A2* | 0.0019 | 0.3477 |
| GO:0008594 | photoreceptor cell morphogenesis | 3 | enriched | 1 | *C8orf37* | 0.0021 | 0.3500 |
| GO:1905166 | negative regulation of lysosomal protein catabolic process | 4 | enriched | 1 | *ATP13A2* | 0.0021 | 0.3500 |
| GO:0055001 | muscle cell development | 4 | enriched | 1 | *NFATC4* | 0.0023 | 0.3720 |
| GO:1904637 | cellular response to ionomycin | 4 | enriched | 1 | *NFATC4* | 0.0023 | 0.3720 |
| GO:1901843 | positive regulation of high voltage-gated calcium channel activity | 5 | enriched | 1 | *GBAS* | 0.0026 | 0.4015 |
| GO:0032212 | positive regulation of telomere maintenance via telomerase | 34 | enriched | 3 | *AURKB, CCT6A, NAF1* | 0.0028 | 0.4191 |
| GO:0072385 | minus-end-directed organelle transport along microtubule | 4 | enriched | 1 | *BICD2* | 0.0028 | 0.4191 |
| GO:0098909 | regulation of cardiac muscle cell action potential involved in regulation of contraction | 8 | enriched | 3 | *AKAP9, CAMK2D, RANGRF* | 0.0028 | 0.4191 |
| GO:1990116 | ribosome-associated ubiquitin-dependent protein catabolic process | 2 | enriched | 1 | *LTN1* | 0.0028 | 0.4191 |
| GO:0000493 | box H/ACA snoRNP assembly | 2 | enriched | 1 | *NAF1* | 0.0029 | 0.4231 |
| GO:1900103 | positive regulation of endoplasmic reticulum unfolded protein response | 5 | enriched | 1 | *PIK3R1* | 0.0029 | 0.4231 |
| GO:0008356 | asymmetric cell division | 5 | enriched | 2 | *ACTR3, PARD3* | 0.0031 | 0.4254 |
| GO:0010955 | negative regulation of protein processing | 15 | enriched | 2 | *GLG1, TMEM98* | 0.0031 | 0.4254 |
| GO:0015693 | magnesium ion transport | 13 | enriched | 2 | *TUSC3, CNNM4* | 0.0031 | 0.4254 |
| GO:0070125 | mitochondrial translational elongation | 89 | enriched | 5 | *MRPL33, MRPS17, MRPS28, MRPS31, MRPS33* | 0.0030 | 0.4254 |
| GO:0070126 | mitochondrial translational termination | 89 | enriched | 5 | *MRPL33, MRPS17, MRPS28, MRPS31, MRPS33* | 0.0031 | 0.4254 |
| GO:1902943 | positive regulation of voltage-gated chloride channel activity | 2 | enriched | 1 | *CFTR* | 0.0031 | 0.4254 |
| GO:0006344 | maintenance of chromatin silencing | 4 | enriched | 1 | *ARID1A* | 0.0033 | 0.4260 |
| GO:0043932 | ossification involved in bone remodeling | 5 | enriched | 1 | *CTHRC1* | 0.0032 | 0.4260 |
| GO:0048864 | stem cell development | 5 | enriched | 1 | *SETD2* | 0.0032 | 0.4260 |
| GO:0030001 | metal ion transport | 5 | enriched | 1 | *NDFIP2* | 0.0034 | 0.4325 |
| GO:0072344 | rescue of stalled ribosome | 5 | enriched | 1 | *LTN1* | 0.0034 | 0.4325 |
| GO:0097065 | anterior head development | 5 | enriched | 1 | *PFAS* | 0.0034 | 0.4325 |
| GO:0098779 | positive regulation of mitophagy in response to mitochondrial depolarization | 4 | enriched | 2 | *CDC37, PARK2* | 0.0034 | 0.4325 |
| GO:0006563 | L-serine metabolic process | 5 | enriched | 1 | *PSPH* | 0.0037 | 0.4559 |
| GO:0032465 | regulation of cytokinesis | 41 | enriched | 3 | *KIF13A, AURKB, SETD2* | 0.0037 | 0.4559 |
| GO:0061088 | regulation of sequestering of zinc ion | 5 | enriched | 1 | *SLC30A2* | 0.0037 | 0.4559 |
| GO:0098883 | synapse pruning | 8 | enriched | 3 | *C1QA, C1QB, C1QC* | 0.0038 | 0.4611 |
| GO:0032648 | regulation of interferon-beta production | 4 | enriched | 1 | *RNF216* | 0.0039 | 0.4638 |
| GO:0048599 | oocyte development | 12 | enriched | 2 | *YTHDC2, ZGLP1* | 0.0040 | 0.4666 |
| GO:1904751 | positive regulation of protein localization to nucleolus | 5 | enriched | 1 | *PINX1* | 0.0040 | 0.4666 |
| GO:0010389 | regulation of G2/M transition of mitotic cell cycle | 80 | enriched | 4 | *AKAP9, CTC1, FGFR1OP, HAUS2* | 0.0043 | 0.4922 |
| GO:0035377 | transepithelial water transport | 2 | enriched | 1 | *CFTR* | 0.0043 | 0.4922 |
| GO:0031547 | brain-derived neurotrophic factor receptor signaling pathway | 5 | enriched | 1 | *NFATC4* | 0.0046 | 0.5139 |
| GO:0043489 | RNA stabilization | 2 | enriched | 1 | *NAF1* | 0.0046 | 0.5139 |
| GO:0031571 | mitotic G1 DNA damage checkpoint | 8 | enriched | 2 | *FBXO31, RFWD3* | 0.0048 | 0.5335 |
| GO:0031344 | regulation of cell projection organization | 3 | enriched | 1 | *PIFO* | 0.0050 | 0.5552 |
| GO:0006734 | NADH metabolic process | 7 | enriched | 1 | *MDH1B* | 0.0055 | 0.5706 |
| GO:0009143 | nucleoside triphosphate catabolic process | 6 | enriched | 1 | *DCTPP1* | 0.0054 | 0.5706 |
| GO:0051225 | spindle assembly | 53 | enriched | 3 | *HAUS2, STARD9, TUBGCP4* | 0.0054 | 0.5706 |
| GO:0060669 | embryonic placenta morphogenesis | 6 | enriched | 1 | *SETD2* | 0.0053 | 0.5706 |
| GO:0072673 | lamellipodium morphogenesis | 5 | enriched | 1 | *WASF2* | 0.0054 | 0.5706 |
| GO:0090669 | telomerase RNA stabilization | 4 | enriched | 1 | *NAF1* | 0.0053 | 0.5706 |
| GO:0097176 | epoxide metabolic process | 3 | enriched | 1 | *EPHX3* | 0.0055 | 0.5706 |
| GO:0007098 | centrosome cycle | 39 | enriched | 3 | *CROCC, HAUS2, SLC16A1* | 0.0057 | 0.5736 |
| GO:0051036 | regulation of endosome size | 2 | enriched | 1 | *RAB5A* | 0.0056 | 0.5736 |
| GO:1901018 | positive regulation of potassium ion transmembrane transporter activity | 6 | enriched | 1 | *AKAP9* | 0.0057 | 0.5736 |
| GO:1905323 | telomerase holoenzyme complex assembly | 4 | enriched | 1 | *NAF1* | 0.0058 | 0.5859 |
| GO:0032410 | negative regulation of transporter activity | 5 | enriched | 1 | *NDFIP2* | 0.0060 | 0.5921 |
| GO:1903830 | magnesium ion transmembrane transport | 15 | enriched | 3 | *TUSC3, CNNM4, ZDHHC13* | 0.0063 | 0.6213 |
| GO:1904871 | positive regulation of protein localization to Cajal body | 9 | enriched | 1 | *CCT6A* | 0.0066 | 0.6474 |
| GO:0045132 | meiotic chromosome segregation | 5 | enriched | 1 | *SMC4* | 0.0068 | 0.6518 |
| GO:0045629 | negative regulation of T-helper 2 cell differentiation | 4 | enriched | 1 | *SOCS5* | 0.0067 | 0.6518 |
| GO:0051224 | negative regulation of protein transport | 6 | enriched | 1 | *NDFIP2* | 0.0069 | 0.6518 |
| GO:0051661 | maintenance of centrosome location | 6 | enriched | 1 | *AKAP9* | 0.0068 | 0.6518 |
| GO:0051383 | kinetochore organization | 6 | enriched | 1 | *SMC4* | 0.0070 | 0.6616 |
| GO:0010971 | positive regulation of G2/M transition of mitotic cell cycle | 27 | enriched | 2 | *BRD4, RCC2* | 0.0072 | 0.6711 |
| GO:0051415 | microtubule nucleation by interphase microtubule organizing center | 6 | enriched | 1 | *TUBGCP4* | 0.0073 | 0.6724 |
| GO:0035565 | regulation of pronephros size | 2 | enriched | 1 | *HNF1A* | 0.0074 | 0.6777 |
| GO:0044829 | positive regulation by host of viral genome replication | 6 | enriched | 1 | *YTHDC2* | 0.0074 | 0.6777 |
| GO:0045606 | positive regulation of epidermal cell differentiation | 7 | enriched | 1 | *SFN* | 0.0075 | 0.6791 |
| GO:0001667 | ameboidal-type cell migration | 6 | enriched | 1 | *WASF2* | 0.0077 | 0.6860 |
| GO:1900180 | regulation of protein localization to nucleus | 6 | enriched | 1 | *ATP13A2* | 0.0077 | 0.6860 |
| GO:0035333 | Notch receptor processing, ligand-dependent | 7 | enriched | 1 | *PSEN2* | 0.0078 | 0.6894 |
| GO:0034340 | response to type I interferon | 7 | enriched | 1 | *SETD2* | 0.0079 | 0.6902 |
| GO:0016126 | sterol biosynthetic process | 15 | enriched | 2 | *CYB5R2, CYP51A1* | 0.0080 | 0.6969 |
| GO:0034629 | cellular protein-containing complex localization | 9 | enriched | 2 | *MIOS, SGCD* | 0.0080 | 0.6969 |
| GO:1904851 | positive regulation of establishment of protein localization to telomere | 10 | enriched | 1 | *CCT6A* | 0.0086 | 0.7373 |
| GO:0051972 | regulation of telomerase activity | 6 | enriched | 1 | *PINX1* | 0.0087 | 0.7426 |
| GO:0048646 | anatomical structure formation involved in morphogenesis | 8 | enriched | 3 | *DLX5, DLX6, GATA3* | 0.0091 | 0.7570 |
| GO:0061024 | membrane organization | 134 | enriched | 7 | *ACTR3, CFTR, PIP5K1C, PMP2, RAB5A, SAR1A, SFN* | 0.0089 | 0.7570 |
| GO:1902570 | protein localization to nucleolus | 7 | enriched | 1 | *PINX1* | 0.0090 | 0.7570 |
| GO:0010032 | meiotic chromosome condensation | 6 | enriched | 1 | *SMC4* | 0.0092 | 0.7605 |
| GO:0006189 | de novo' IMP biosynthetic process | 6 | enriched | 1 | *PFAS* | 0.0093 | 0.7619 |
| GO:0006564 | L-serine biosynthetic process | 7 | enriched | 1 | *PSPH* | 0.0093 | 0.7619 |
| GO:0051454 | intracellular pH elevation | 4 | enriched | 1 | *CFTR* | 0.0096 | 0.7750 |
| GO:1901016 | regulation of potassium ion transmembrane transporter activity | 6 | enriched | 2 | *NEDD4, NEDD4L* | 0.0095 | 0.7750 |
| GO:0006154 | adenosine catabolic process | 3 | enriched | 1 | *ADAL* | 0.0102 | 0.7753 |
| GO:0006457 | protein folding | 139 | enriched | 8 | *CCT6A, CDC37, DNAJC25, GNAO1, GNAZ, LMAN2L, NUDC, TBCD* | 0.0098 | 0.7753 |
| GO:0010457 | centriole-centriole cohesion | 11 | enriched | 2 | *CROCC, RTTN* | 0.0102 | 0.7753 |
| GO:0010768 | negative regulation of transcription from RNA polymerase II promoter in response to UV-induced DNA damage | 2 | enriched | 1 | *NEDD4* | 0.0102 | 0.7753 |
| GO:0019075 | virus maturation | 2 | enriched | 1 | *MVB12B* | 0.0100 | 0.7753 |
| GO:0035623 | renal glucose absorption | 1 | enriched | 1 | *HNF1A* | 0.0102 | 0.7753 |
| GO:0043162 | ubiquitin-dependent protein catabolic process via the multivesicular body sorting pathway | 18 | enriched | 2 | *MVB12B, NEDD4* | 0.0102 | 0.7753 |
| GO:0046103 | inosine biosynthetic process | 3 | enriched | 1 | *ADAL* | 0.0102 | 0.7753 |
| GO:0051653 | spindle localization | 5 | enriched | 1 | *ACTR3* | 0.0098 | 0.7753 |
| GO:1900222 | negative regulation of amyloid-beta clearance | 8 | enriched | 1 | *CYP51A1* | 0.0099 | 0.7753 |
| GO:0018023 | peptidyl-lysine trimethylation | 8 | enriched | 1 | *SETD2* | 0.0104 | 0.7856 |
| GO:0015884 | folic acid transport | 8 | enriched | 1 | *SLC25A32* | 0.0108 | 0.8083 |
| GO:1902525 | regulation of protein monoubiquitination | 1 | enriched | 1 | *WDR48* | 0.0111 | 0.8221 |
| GO:1904358 | positive regulation of telomere maintenance via telomere lengthening | 5 | enriched | 1 | *NAF1* | 0.0111 | 0.8221 |
| GO:0031048 | chromatin silencing by small RNA | 3 | enriched | 1 | *FAM172A* | 0.0114 | 0.8377 |
| GO:0045191 | regulation of isotype switching | 4 | enriched | 1 | *APLF* | 0.0115 | 0.8414 |
| GO:0007091 | metaphase/anaphase transition of mitotic cell cycle | 6 | enriched | 1 | *CDC27* | 0.0118 | 0.8543 |
| GO:0039536 | negative regulation of RIG-I signaling pathway | 8 | enriched | 1 | *GPATCH3* | 0.0118 | 0.8543 |
| GO:0051106 | positive regulation of DNA ligation | 4 | enriched | 1 | *APLF* | 0.0125 | 0.8846 |
| GO:0071285 | cellular response to lithium ion | 14 | enriched | 2 | *NFATC4, FABP4* | 0.0124 | 0.8846 |
| GO:0071287 | cellular response to manganese ion | 9 | enriched | 2 | *ATP13A2, PARK2* | 0.0125 | 0.8846 |
| GO:0033157 | regulation of intracellular protein transport | 7 | enriched | 1 | *ATP13A2* | 0.0126 | 0.8876 |
| GO:0006108 | malate metabolic process | 8 | enriched | 1 | *MDH1B* | 0.0128 | 0.8972 |
| GO:0006903 | vesicle targeting | 8 | enriched | 1 | *SNAP23* | 0.0130 | 0.9020 |
| GO:0010833 | telomere maintenance via telomere lengthening | 9 | enriched | 1 | *CTC1* | 0.0130 | 0.9020 |
| GO:0003334 | keratinocyte development | 8 | enriched | 1 | *SFN* | 0.0131 | 0.9042 |
| GO:0007018 | microtubule-based movement | 78 | enriched | 6 | *KIF13A, BICD2, KIF17, KIF6, KIF9, STARD9* | 0.0134 | 0.9044 |
| GO:0010923 | negative regulation of phosphatase activity | 49 | enriched | 5 | *GPATCH2, CD2BP2, DLG2, PPP1R37, TMEM132D* | 0.0132 | 0.9044 |
| GO:2000650 | negative regulation of sodium ion transmembrane transporter activity | 9 | enriched | 4 | *CAMK2D, NEDD4, NEDD4L, PCSK9* | 0.0134 | 0.9044 |
| GO:0006107 | oxaloacetate metabolic process | 8 | enriched | 1 | *MDH1B* | 0.0136 | 0.9148 |
| GO:0019054 | modulation by virus of host cellular process | 7 | enriched | 1 | *KPNA4* | 0.0137 | 0.9163 |
| GO:0052548 | regulation of endopeptidase activity | 8 | enriched | 1 | *ATP13A2* | 0.0139 | 0.9260 |
| GO:0097752 | regulation of DNA stability | 4 | enriched | 1 | *DDI1* | 0.0141 | 0.9359 |
| GO:0070358 | actin polymerization-dependent cell motility | 7 | enriched | 1 | *ACTR3* | 0.0143 | 0.9399 |
| GO:0006105 | succinate metabolic process | 7 | enriched | 1 | *SDHB* | 0.0145 | 0.9466 |
| GO:0006663 | platelet activating factor biosynthetic process | 4 | enriched | 2 | *PLA2G4A, PLA2G5* | 0.0146 | 0.9509 |
| GO:0050691 | regulation of defense response to virus by host | 7 | enriched | 1 | *RNF216* | 0.0150 | 0.9709 |
| GO:1903008 | organelle disassembly | 2 | enriched | 1 | *KIF9* | 0.0152 | 0.9821 |
| GO:0000050 | urea cycle | 11 | enriched | 1 | *SLC25A15* | 0.0258 | 0.9869 |
| GO:0000244 | spliceosomal tri-snRNP complex assembly | 11 | enriched | 1 | *CD2BP2* | 0.0311 | 0.9869 |
| GO:0000350 | generation of catalytic spliceosome for second transesterification step | 2 | enriched | 1 | *PRPF18* | 0.0387 | 0.9869 |
| GO:0001678 | cellular glucose homeostasis | 19 | enriched | 2 | *FOXO1, PIK3R1* | 0.0281 | 0.9869 |
| GO:0001763 | morphogenesis of a branching structure | 10 | enriched | 1 | *SETD2* | 0.0237 | 0.9869 |
| GO:0001959 | regulation of cytokine-mediated signaling pathway | 7 | enriched | 1 | *ELF1* | 0.0159 | 0.9869 |
| GO:0003197 | endocardial cushion development | 9 | enriched | 1 | *NEDD4* | 0.0248 | 0.9869 |
| GO:0006099 | tricarboxylic acid cycle | 35 | enriched | 2 | *SDHB, MDH1B* | 0.0238 | 0.9869 |
| GO:0006119 | oxidative phosphorylation | 12 | enriched | 1 | *GBAS* | 0.0293 | 0.9869 |
| GO:0006259 | DNA metabolic process | 24 | enriched | 2 | *ERI1, TOPBP1* | 0.0431 | 0.9869 |
| GO:0006435 | threonyl-tRNA aminoacylation | 3 | enriched | 1 | *TARSL2* | 0.0460 | 0.9869 |
| GO:0006468 | protein phosphorylation | 476 | enriched | 24 | *PRKCB, ADCK3, AURKB, BRD4, CAMK2D, CCND3, CDC42BPA, FASTKD2, FER, HUS1, MAP3K1, MAP3K6, MARK4, MLKL, PHKG1, PHKG2, PIK3R1, RPS6KA2, SGK1, SIK2, TEC, TGFBR2, TSSK1B, TYK2* | 0.0246 | 0.9869 |
| GO:0006607 | NLS-bearing protein import into nucleus | 20 | enriched | 2 | *CBLB, KPNA4* | 0.0188 | 0.9869 |
| GO:0006778 | porphyrin-containing compound metabolic process | 2 | enriched | 2 | *CYP1A1, CYP1A2* | 0.0248 | 0.9869 |
| GO:0007034 | vacuolar transport | 9 | enriched | 1 | *NDFIP2* | 0.0209 | 0.9869 |
| GO:0007220 | Notch receptor processing | 9 | enriched | 1 | *PSEN2* | 0.0175 | 0.9869 |
| GO:0009113 | purine nucleobase biosynthetic process | 8 | enriched | 1 | *PFAS* | 0.0190 | 0.9869 |
| GO:0009403 | toxin biosynthetic process | 1 | enriched | 1 | *CYP1A2* | 0.0164 | 0.9869 |
| GO:0009820 | alkaloid metabolic process | 2 | enriched | 1 | *CYP1A2* | 0.0242 | 0.9869 |
| GO:0009972 | cytidine deamination | 12 | enriched | 1 | *APOBEC2* | 0.0486 | 0.9869 |
| GO:0010212 | response to ionizing radiation | 46 | enriched | 3 | *BRE, RFWD3, TOPBP1* | 0.0325 | 0.9869 |
| GO:0010592 | positive regulation of lamellipodium assembly | 21 | enriched | 2 | *FSCN1, WASF2* | 0.0427 | 0.9869 |
| GO:0010762 | regulation of fibroblast migration | 11 | enriched | 2 | *FER, RCC2* | 0.0287 | 0.9869 |
| GO:0010766 | negative regulation of sodium ion transport | 6 | enriched | 1 | *NEDD4* | 0.0382 | 0.9869 |
| GO:0010830 | regulation of myotube differentiation | 5 | enriched | 1 | *HDAC5* | 0.0494 | 0.9869 |
| GO:0010839 | negative regulation of keratinocyte proliferation | 12 | enriched | 1 | *SFN* | 0.0397 | 0.9869 |
| GO:0015718 | monocarboxylic acid transport | 13 | enriched | 2 | *SLC16A1, SLC16A5* | 0.0264 | 0.9869 |
| GO:0016125 | sterol metabolic process | 23 | enriched | 2 | *CYP51A1, FDX1L* | 0.0201 | 0.9869 |
| GO:0016570 | histone modification | 4 | enriched | 1 | *AURKB* | 0.0161 | 0.9869 |
| GO:0018026 | peptidyl-lysine monomethylation | 10 | enriched | 1 | *SETD2* | 0.0243 | 0.9869 |
| GO:0018894 | dibenzo-p-dioxin metabolic process | 2 | enriched | 1 | *CYP1A2* | 0.0262 | 0.9869 |
| GO:0022904 | respiratory electron transport chain | 17 | enriched | 2 | *SDHB, IMMP2L* | 0.0491 | 0.9869 |
| GO:0023051 | regulation of signaling | 2 | enriched | 1 | *LMO7* | 0.0399 | 0.9869 |
| GO:0030520 | intracellular estrogen receptor signaling pathway | 19 | enriched | 2 | *ESR1, ARID1A* | 0.0386 | 0.9869 |
| GO:0030900 | forebrain development | 47 | enriched | 4 | *ARID1A, DLC1, GNAO1, SETD2* | 0.0440 | 0.9869 |
| GO:0031116 | positive regulation of microtubule polymerization | 25 | enriched | 2 | *AKAP9, MET* | 0.0394 | 0.9869 |
| GO:0031125 | rRNA 3'-end processing | 1 | enriched | 1 | *ERI1* | 0.0348 | 0.9869 |
| GO:0031629 | synaptic vesicle fusion to presynaptic active zone membrane | 13 | enriched | 1 | *SNAP23* | 0.0485 | 0.9869 |
| GO:0031952 | regulation of protein autophosphorylation | 8 | enriched | 1 | *PPP2R5A* | 0.0185 | 0.9869 |
| GO:0032330 | regulation of chondrocyte differentiation | 10 | enriched | 1 | *GLG1* | 0.0358 | 0.9869 |
| GO:0032480 | negative regulation of type I interferon production | 30 | enriched | 2 | *GPATCH3, RNF216* | 0.0168 | 0.9869 |
| GO:0032727 | positive regulation of interferon-alpha production | 12 | enriched | 1 | *SETD2* | 0.0385 | 0.9869 |
| GO:0032958 | inositol phosphate biosynthetic process | 11 | enriched | 1 | *IPPK* | 0.0492 | 0.9869 |
| GO:0033314 | mitotic DNA replication checkpoint | 10 | enriched | 2 | *HUS1, TOPBP1* | 0.0228 | 0.9869 |
| GO:0033690 | positive regulation of osteoblast proliferation | 10 | enriched | 1 | *CTHRC1* | 0.0197 | 0.9869 |
| GO:0034333 | adherens junction assembly | 10 | enriched | 2 | *PIP5K1C, TBCD* | 0.0461 | 0.9869 |
| GO:0035459 | vesicle cargo loading | 13 | enriched | 1 | *KIF13A* | 0.0380 | 0.9869 |
| GO:0035522 | monoubiquitinated histone H2A deubiquitination | 4 | enriched | 1 | *USP16* | 0.0424 | 0.9869 |
| GO:0035562 | negative regulation of chromatin binding | 9 | enriched | 1 | *NFATC4* | 0.0229 | 0.9869 |
| GO:0035987 | endodermal cell differentiation | 28 | enriched | 2 | *COL8A1, SETD2* | 0.0348 | 0.9869 |
| GO:0036297 | interstrand cross-link repair | 50 | enriched | 4 | *FANCM, RFWD3, RPA3, WDR48* | 0.0198 | 0.9869 |
| GO:0036465 | synaptic vesicle recycling | 6 | enriched | 1 | *RAB5A* | 0.0378 | 0.9869 |
| GO:0038096 | Fc-gamma receptor signaling pathway involved in phagocytosis | 72 | enriched | 3 | *ACTR3, PIK3R1, WASF2* | 0.0413 | 0.9869 |
| GO:0038128 | ERBB2 signaling pathway | 31 | enriched | 2 | *CDC37, PIK3R1* | 0.0245 | 0.9869 |
| GO:0038155 | interleukin-23-mediated signaling pathway | 9 | enriched | 1 | *TYK2* | 0.0184 | 0.9869 |
| GO:0042987 | amyloid precursor protein catabolic process | 10 | enriched | 1 | *PSEN2* | 0.0220 | 0.9869 |
| GO:0043988 | histone H3-S28 phosphorylation | 3 | enriched | 1 | *AURKB* | 0.0198 | 0.9869 |
| GO:0044878 | mitotic cytokinesis checkpoint | 4 | enriched | 1 | *AURKB* | 0.0240 | 0.9869 |
| GO:0045184 | establishment of protein localization | 40 | enriched | 5 | *FAM65B, MCC, NLGN1, RAP2A, RCC2* | 0.0407 | 0.9869 |
| GO:0045627 | positive regulation of T-helper 1 cell differentiation | 6 | enriched | 1 | *SOCS5* | 0.0164 | 0.9869 |
| GO:0045717 | negative regulation of fatty acid biosynthetic process | 13 | enriched | 1 | *WDTC1* | 0.0422 | 0.9869 |
| GO:0045901 | positive regulation of translational elongation | 4 | enriched | 1 | *USP16* | 0.0279 | 0.9869 |
| GO:0045921 | positive regulation of exocytosis | 23 | enriched | 2 | *CFTR, RAB5A* | 0.0238 | 0.9869 |
| GO:0048048 | embryonic eye morphogenesis | 9 | enriched | 1 | *MFAP2* | 0.0184 | 0.9869 |
| GO:0048096 | chromatin-mediated maintenance of transcription | 11 | enriched | 1 | *ARID1A* | 0.0334 | 0.9869 |
| GO:0048539 | bone marrow development | 9 | enriched | 1 | *CTC1* | 0.0173 | 0.9869 |
| GO:0050847 | progesterone receptor signaling pathway | 8 | enriched | 1 | *NEDD4* | 0.0170 | 0.9869 |
| GO:0050855 | regulation of B cell receptor signaling pathway | 10 | enriched | 1 | *ELF1* | 0.0403 | 0.9869 |
| GO:0050891 | multicellular organismal water homeostasis | 8 | enriched | 1 | *CFTR* | 0.0468 | 0.9869 |
| GO:0051497 | negative regulation of stress fiber assembly | 26 | enriched | 4 | *ARHGAP28, DLC1, MET, WASF2* | 0.0351 | 0.9869 |
| GO:0051656 | establishment of organelle localization | 2 | enriched | 1 | *CROCC* | 0.0314 | 0.9869 |
| GO:0051974 | negative regulation of telomerase activity | 11 | enriched | 1 | *PINX1* | 0.0396 | 0.9869 |
| GO:0055065 | metal ion homeostasis | 3 | enriched | 1 | *CNNM4* | 0.0226 | 0.9869 |
| GO:0060039 | pericardium development | 9 | enriched | 1 | *SETD2* | 0.0229 | 0.9869 |
| GO:0060306 | regulation of membrane repolarization | 10 | enriched | 2 | *AKAP9, NEDD4L* | 0.0232 | 0.9869 |
| GO:0060338 | regulation of type I interferon-mediated signaling pathway | 9 | enriched | 1 | *CDC37* | 0.0492 | 0.9869 |
| GO:0060349 | bone morphogenesis | 31 | enriched | 2 | *GLG1, IFT80* | 0.0376 | 0.9869 |
| GO:0060396 | growth hormone receptor signaling pathway | 12 | enriched | 1 | *PIK3R1* | 0.0304 | 0.9869 |
| GO:0060561 | apoptotic process involved in morphogenesis | 6 | enriched | 1 | *PPP2R1B* | 0.0182 | 0.9869 |
| GO:0060674 | placenta blood vessel development | 11 | enriched | 2 | *ARID1A, ITGB8* | 0.0262 | 0.9869 |
| GO:0060732 | positive regulation of inositol phosphate biosynthetic process | 8 | enriched | 1 | *PTH1R* | 0.0371 | 0.9869 |
| GO:0061436 | establishment of skin barrier | 23 | enriched | 2 | *MET, SFN* | 0.0182 | 0.9869 |
| GO:0070106 | interleukin-27-mediated signaling pathway | 11 | enriched | 1 | *TYK2* | 0.0381 | 0.9869 |
| GO:0070166 | enamel mineralization | 11 | enriched | 2 | *CNNM4, FOXO1* | 0.0173 | 0.9869 |
| GO:0070198 | protein localization to chromosome, telomeric region | 10 | enriched | 1 | *PINX1* | 0.0329 | 0.9869 |
| GO:0070495 | negative regulation of thrombin-activated receptor signaling pathway | 3 | enriched | 1 | *MET* | 0.0383 | 0.9869 |
| GO:0070537 | histone H2A K63-linked deubiquitination | 3 | enriched | 1 | *USP16* | 0.0305 | 0.9869 |
| GO:0071048 | nuclear retention of unspliced pre-mRNA at the site of transcription | 2 | enriched | 1 | *PRPF18* | 0.0388 | 0.9869 |
| GO:0071320 | cellular response to cAMP | 53 | enriched | 4 | *ITPR2, AKAP9, CFTR, HCN1* | 0.0258 | 0.9869 |
| GO:0071322 | cellular response to carbohydrate stimulus | 1 | enriched | 1 | *PRKCB* | 0.0461 | 0.9869 |
| GO:0071476 | cellular hypotonic response | 7 | enriched | 1 | *CAB39* | 0.0290 | 0.9869 |
| GO:0071615 | oxidative deethylation | 1 | enriched | 1 | *CYP1A2* | 0.0164 | 0.9869 |
| GO:0071638 | negative regulation of monocyte chemotactic protein-1 production | 6 | enriched | 1 | *SOCS5* | 0.0198 | 0.9869 |
| GO:0071801 | regulation of podosome assembly | 3 | enriched | 1 | *KIF9* | 0.0212 | 0.9869 |
| GO:0072383 | plus-end-directed vesicle transport along microtubule | 7 | enriched | 1 | *KIF13A* | 0.0164 | 0.9869 |
| GO:0097376 | interneuron axon guidance | 1 | enriched | 1 | *DLX5* | 0.0215 | 0.9869 |
| GO:0097502 | mannosylation | 11 | enriched | 1 | *PIGV* | 0.0268 | 0.9869 |
| GO:0097699 | vascular endothelial cell response to fluid shear stress | 1 | enriched | 1 | *SOCS5* | 0.0204 | 0.9869 |
| GO:0098962 | regulation of postsynaptic neurotransmitter receptor activity | 13 | enriched | 1 | *AKAP9* | 0.0494 | 0.9869 |
| GO:0099172 | presynapse organization | 2 | enriched | 1 | *PPFIA2* | 0.0477 | 0.9869 |
| GO:0140014 | mitotic nuclear division | 3 | enriched | 1 | *USP16* | 0.0221 | 0.9869 |
| GO:0150093 | amyloid-beta clearance by transcytosis | 7 | enriched | 1 | *RAB5A* | 0.0495 | 0.9869 |
| GO:1900025 | negative regulation of substrate adhesion-dependent cell spreading | 13 | enriched | 2 | *KANK1, RCC2* | 0.0478 | 0.9869 |
| GO:1900027 | regulation of ruffle assembly | 10 | enriched | 1 | *RCC2* | 0.0287 | 0.9869 |
| GO:1900112 | regulation of histone H3-K9 trimethylation | 2 | enriched | 1 | *ZNF274* | 0.0407 | 0.9869 |
| GO:1901299 | negative regulation of hydrogen peroxide-mediated programmed cell death | 2 | enriched | 1 | *MET* | 0.0271 | 0.9869 |
| GO:1901838 | positive regulation of transcription of nucleolar large rRNA by RNA polymerase I | 10 | enriched | 1 | *IPPK* | 0.0309 | 0.9869 |
| GO:1902161 | positive regulation of cyclic nucleotide-gated ion channel activity | 1 | enriched | 1 | *CFTR* | 0.0447 | 0.9869 |
| GO:1902894 | negative regulation of pri-miRNA transcription by RNA polymerase II | 13 | enriched | 2 | *NFATC4, NFIB* | 0.0381 | 0.9869 |
| GO:1903003 | positive regulation of protein deubiquitination | 4 | enriched | 1 | *WDR48* | 0.0234 | 0.9869 |
| GO:1904357 | negative regulation of telomere maintenance via telomere lengthening | 10 | enriched | 1 | *PINX1* | 0.0289 | 0.9869 |
| GO:1904668 | positive regulation of ubiquitin protein ligase activity | 11 | enriched | 1 | *GORASP1* | 0.0260 | 0.9869 |
| GO:2000001 | regulation of DNA damage checkpoint | 8 | enriched | 1 | *RFWD3* | 0.0163 | 0.9869 |
| GO:2000051 | negative regulation of non-canonical Wnt signaling pathway | 4 | enriched | 1 | *IFT80* | 0.0181 | 0.9869 |
| GO:2000286 | receptor internalization involved in canonical Wnt signaling pathway | 3 | enriched | 1 | *RAB5A* | 0.0242 | 0.9869 |
| GO:2000583 | regulation of platelet-derived growth factor receptor-alpha signaling pathway | 3 | enriched | 1 | *CBLB* | 0.0182 | 0.9869 |

| **S6b Table. Results of the pathway-based analysis of QLSCD_Mean Pain and QLSCD_Mean Anxiety (uncorrected p-value < 0.05).** | | | | | | | |
| --- | --- | --- | --- | --- | --- | --- | --- |
| **QLSCD_Mean Anxiety** | | | | | | | |
| **GO set ID** | **Description** | **Pathway size (nr of genes)** | **Status** | **Significant Genes (nr of)** | **Genes** | ***p-value*** | **FDR** |
| GO:0007156 | homophilic cell adhesion via plasma membrane adhesion molecules | 162 | enriched | 26 | *PCDHGA1, PCDHGA2, PCDHGA3, PCDHGA4, PCDHGA5, PCDHGB1, PCDHGB2, PCDHGB3, PCDHGA10, PCDHGA11, PCDHGA12, PCDHGA6, PCDHGA7, PCDHGA8, PCDHGA9, PCDHGB4, PCDHGB6, PCDHGB7, PCDHGC3, PCDHGC4, PCDHGC5, ROBO2, DCHS2, IGSF21, NEXN, TENM3* | <0.0001 | <0.0001 |
| GO:0000154 | rRNA modification | 4 | enriched | 2 | *TFB1M, TSR3* | <0.0001 | 0.0403 |
| GO:0072385 | minus-end-directed organelle transport along microtubule | 4 | enriched | 2 | *BICD2, RAB6A* | <0.0001 | 0.0403 |
| GO:0002437 | inflammatory response to antigenic stimulus | 24 | enriched | 4 | *IL1F10, IL1RN, IL36B, IL36RN* | <0.0001 | 0.0494 |
| GO:0031571 | mitotic G1 DNA damage checkpoint | 8 | enriched | 2 | *FBXO31, RFWD3* | <0.0001 | 0.0494 |
| GO:0051646 | mitochondrion localization | 8 | enriched | 2 | *MUL1, MFN2* | <0.0001 | 0.1091 |
| GO:0071287 | cellular response to manganese ion | 9 | enriched | 2 | *A3GALT2, ATP13A2* | 0.0001 | 0.1843 |
| GO:0032053 | ciliary basal body organization | 3 | enriched | 2 | *RTTN, CROCC* | 0.0001 | 0.1952 |
| GO:0010466 | negative regulation of peptidase activity | 16 | enriched | 2 | *SERPINB3, SERPINB4* | 0.0001 | 0.2466 |
| GO:0010766 | negative regulation of sodium ion transport | 6 | enriched | 2 | *NEDD4, WNK1* | 0.0002 | 0.2505 |
| GO:0030162 | regulation of proteolysis | 12 | enriched | 2 | *SERPINB13, SERPINB4* | 0.0002 | 0.2505 |
| GO:0034472 | snRNA 3'-end processing | 9 | enriched | 2 | *INTS6, INTS8* | 0.0002 | 0.2505 |
| GO:2000637 | positive regulation of gene silencing by miRNA | 11 | enriched | 2 | *DHX9, PUM2* | 0.0002 | 0.2505 |
| GO:0034644 | cellular response to UV | 49 | enriched | 4 | *NEDD4, NFATC4, PIK3R1, TP53INP1* | 0.0002 | 0.2554 |
| GO:0006105 | succinate metabolic process | 7 | enriched | 2 | *ALDH5A1, SDHB* | 0.0008 | 0.3045 |
| GO:0006622 | protein targeting to lysosome | 16 | enriched | 2 | *NEDD4, ZFYVE16* | 0.0011 | 0.3045 |
| GO:0007518 | myoblast fate determination | 2 | enriched | 1 | *IFRD1* | 0.0009 | 0.3045 |
| GO:0010569 | regulation of double-strand break repair via homologous recombination | 18 | enriched | 3 | *TEX15, ZNF365, SETD2* | 0.0006 | 0.3045 |
| GO:0010821 | regulation of mitochondrion organization | 16 | enriched | 2 | *MUL1, ATP13A2* | 0.0008 | 0.3045 |
| GO:0014718 | positive regulation of satellite cell activation involved in skeletal muscle regeneration | 2 | enriched | 1 | *CAPN3* | 0.0009 | 0.3045 |
| GO:0015711 | organic anion transport | 14 | enriched | 4 | *SLC22A10, SLC22A24, SLC22A25, SLC22A9* | 0.0007 | 0.3045 |
| GO:0016180 | snRNA processing | 13 | enriched | 3 | *INTS10, INTS6, INTS8* | 0.0012 | 0.3045 |
| GO:0018872 | arsonoacetate metabolic process | 2 | enriched | 1 | *AS3MT* | 0.0009 | 0.3045 |
| GO:0021524 | visceral motor neuron differentiation | 3 | enriched | 1 | *ISL2* | 0.0010 | 0.3045 |
| GO:0021885 | forebrain cell migration | 3 | enriched | 1 | *EMX2* | 0.0011 | 0.3045 |
| GO:0030003 | cellular cation homeostasis | 2 | enriched | 1 | *ATP13A2* | 0.0008 | 0.3045 |
| GO:0030517 | negative regulation of axon extension | 17 | enriched | 2 | *IFRD1, KIAA0319* | 0.0010 | 0.3045 |
| GO:0030702 | chromatin silencing at centromere | 2 | enriched | 1 | *ZNFX1* | 0.0012 | 0.3045 |
| GO:0031052 | chromosome breakage | 2 | enriched | 1 | *RFWD3* | 0.0012 | 0.3045 |
| GO:0035441 | cell migration involved in vasculogenesis | 2 | enriched | 1 | *SETD2* | 0.0012 | 0.3045 |
| GO:0042264 | peptidyl-aspartic acid hydroxylation | 2 | enriched | 1 | *HIF1AN* | 0.0012 | 0.3045 |
| GO:0043000 | Golgi to plasma membrane CFTR protein transport | 2 | enriched | 1 | *KRT18* | 0.0008 | 0.3045 |
| GO:0045081 | negative regulation of interleukin-10 biosynthetic process | 2 | enriched | 1 | *TRIB2* | 0.0006 | 0.3045 |
| GO:0048332 | mesoderm morphogenesis | 2 | enriched | 1 | *SETD2* | 0.0011 | 0.3045 |
| GO:0050689 | negative regulation of defense response to virus by host | 3 | enriched | 1 | *MUL1* | 0.0005 | 0.3045 |
| GO:0052746 | inositol phosphorylation | 2 | enriched | 1 | *IPPK* | 0.0012 | 0.3045 |
| GO:0060977 | coronary vasculature morphogenesis | 3 | enriched | 2 | *SETD2, SGCD* | 0.0008 | 0.3045 |
| GO:0070315 | G1 to G0 transition involved in cell differentiation | 3 | enriched | 1 | *CAPN3* | 0.0010 | 0.3045 |
| GO:0071344 | diphosphate metabolic process | 2 | enriched | 1 | *PPA1* | 0.0011 | 0.3045 |
| GO:0071360 | cellular response to exogenous dsRNA | 17 | enriched | 2 | *MUL1, DHX9* | 0.0006 | 0.3045 |
| GO:0072393 | microtubule anchoring at microtubule organizing center | 2 | enriched | 1 | *BICD2* | 0.0012 | 0.3045 |
| GO:0097198 | histone H3-K36 trimethylation | 2 | enriched | 1 | *SETD2* | 0.0011 | 0.3045 |
| GO:0097250 | mitochondrial respirasome assembly | 3 | enriched | 1 | *COX7A2L* | 0.0011 | 0.3045 |
| GO:1901028 | regulation of mitochondrial outer membrane permeabilization involved in apoptotic signaling pathway | 2 | enriched | 1 | *MUL1* | 0.0004 | 0.3045 |
| GO:1902714 | negative regulation of interferon-gamma secretion | 3 | enriched | 1 | *IL36RN* | 0.0012 | 0.3045 |
| GO:1902775 | mitochondrial large ribosomal subunit assembly | 3 | enriched | 1 | *FASTKD2* | 0.0011 | 0.3045 |
| GO:1902850 | microtubule cytoskeleton organization involved in mitosis | 2 | enriched | 1 | *SETD2* | 0.0011 | 0.3045 |
| GO:1904030 | negative regulation of cyclin-dependent protein kinase activity | 4 | enriched | 1 | *CAMK2N1* | 0.0008 | 0.3045 |
| GO:1905165 | regulation of lysosomal protein catabolic process | 2 | enriched | 1 | *ATP13A2* | 0.0008 | 0.3045 |
| GO:1990456 | mitochondrion-endoplasmic reticulum membrane tethering | 2 | enriched | 1 | *PSEN2* | 0.0011 | 0.3045 |
| GO:1990481 | mRNA pseudouridine synthesis | 5 | enriched | 2 | *PUS7, TRUB1* | 0.0006 | 0.3045 |
| GO:1905037 | autophagosome organization | 3 | enriched | 1 | *ATP13A2* | 0.0013 | 0.3154 |
| GO:0048692 | negative regulation of axon extension involved in regeneration | 2 | enriched | 1 | *KIAA0319* | 0.0013 | 0.3203 |
| GO:0031048 | chromatin silencing by small RNA | 3 | enriched | 1 | *ZNFX1* | 0.0014 | 0.3236 |
| GO:0042270 | protection from natural killer cell mediated cytotoxicity | 5 | enriched | 1 | *SERPINB4* | 0.0015 | 0.3240 |
| GO:0048050 | post-embryonic eye morphogenesis | 3 | enriched | 1 | *MFAP2* | 0.0015 | 0.3240 |
| GO:0071472 | cellular response to salt stress | 3 | enriched | 1 | *CAPN3* | 0.0015 | 0.3240 |
| GO:1904761 | negative regulation of myofibroblast differentiation | 3 | enriched | 1 | *TP53INP1* | 0.0015 | 0.3240 |
| GO:0061428 | negative regulation of transcription from RNA polymerase II promoter in response to hypoxia | 4 | enriched | 1 | *HIF1AN* | 0.0016 | 0.3311 |
| GO:2000765 | regulation of cytoplasmic translation | 2 | enriched | 1 | *DHX9* | 0.0016 | 0.3413 |
| GO:1990502 | dense core granule maturation | 3 | enriched | 1 | *BAIAP3* | 0.0017 | 0.3570 |
| GO:1904925 | positive regulation of autophagy of mitochondrion in response to mitochondrial depolarization | 4 | enriched | 1 | *MUL1* | 0.0018 | 0.3599 |
| GO:1905123 | regulation of glucosylceramidase activity | 2 | enriched | 1 | *ATP13A2* | 0.0018 | 0.3599 |
| GO:0051987 | positive regulation of attachment of spindle microtubules to kinetochore | 3 | enriched | 1 | *RCC2* | 0.0019 | 0.3604 |
| GO:0071650 | negative regulation of chemokine (C-C motif) ligand 5 production | 4 | enriched | 1 | *MUL1* | 0.0019 | 0.3609 |
| GO:0032741 | positive regulation of interleukin-18 production | 4 | enriched | 1 | *DHX9* | 0.0020 | 0.3661 |
| GO:0050808 | synapse organization | 46 | enriched | 5 | *PCDHGC3, PCDHGC4, PCDHGC5, C1QA, CTNND2* | 0.0020 | 0.3661 |
| GO:0060669 | embryonic placenta morphogenesis | 6 | enriched | 2 | *SETD2, ZNF568* | 0.0020 | 0.3661 |
| GO:0060711 | labyrinthine layer development | 7 | enriched | 2 | *BIRC6, CDX2* | 0.0021 | 0.3661 |
| GO:1903008 | organelle disassembly | 2 | enriched | 2 | *DYRK3, KIF9* | 0.0020 | 0.3661 |
| GO:0019732 | antifungal humoral response | 5 | enriched | 1 | *IL36RN* | 0.0022 | 0.3718 |
| GO:0021846 | cell proliferation in forebrain | 12 | enriched | 2 | *EMX2, SIX3* | 0.0021 | 0.3718 |
| GO:0030423 | targeting of mRNA for destruction involved in RNA interference | 4 | enriched | 1 | *DHX9* | 0.0023 | 0.3718 |
| GO:0040020 | regulation of meiotic nuclear division | 5 | enriched | 2 | *GPR3, PDE3A* | 0.0023 | 0.3718 |
| GO:0045861 | negative regulation of proteolysis | 22 | enriched | 2 | *CAMK2N1, SERPINB3* | 0.0022 | 0.3718 |
| GO:2000297 | negative regulation of synapse maturation | 3 | enriched | 1 | *NFATC4* | 0.0022 | 0.3718 |
| GO:0007049 | cell cycle | 246 | enriched | 12 | *KIF13A, RINT1, BIRC6, BRE, CTCFL, DYRK3, KRT18, MARK4, MPLKIP, RCC2, UBE2I, USP39* | 0.0023 | 0.3766 |
| GO:1904714 | regulation of chaperone-mediated autophagy | 5 | enriched | 1 | *ATP13A2* | 0.0024 | 0.3843 |
| GO:0001915 | negative regulation of T cell mediated cytotoxicity | 6 | enriched | 2 | *IL7R, LILRB1* | 0.0025 | 0.3880 |
| GO:0002553 | histamine secretion by mast cell | 3 | enriched | 1 | *SNAP23* | 0.0025 | 0.3880 |
| GO:0006469 | negative regulation of protein kinase activity | 72 | enriched | 5 | *CAMK2N1, FABP4, TAF7, TRIB2, WNK1* | 0.0027 | 0.3880 |
| GO:0014706 | striated muscle tissue development | 5 | enriched | 2 | *EYA1, IFRD1* | 0.0028 | 0.3880 |
| GO:0022904 | respiratory electron transport chain | 17 | enriched | 4 | *ALDH5A1, ETFA, IMMP2L, SDHB* | 0.0028 | 0.3880 |
| GO:0034067 | protein localization to Golgi apparatus | 20 | enriched | 2 | *BICD2, RAB6A* | 0.0028 | 0.3880 |
| GO:0034728 | nucleosome organization | 3 | enriched | 1 | *SETD2* | 0.0026 | 0.3880 |
| GO:0055069 | zinc ion homeostasis | 5 | enriched | 1 | *ATP13A2* | 0.0027 | 0.3880 |
| GO:0097676 | histone H3-K36 dimethylation | 4 | enriched | 1 | *SETD2* | 0.0027 | 0.3880 |
| GO:1904528 | positive regulation of microtubule binding | 4 | enriched | 1 | *PPP2CB* | 0.0027 | 0.3880 |
| GO:0072197 | ureter morphogenesis | 6 | enriched | 1 | *EMX2* | 0.0028 | 0.3939 |
| GO:0060260 | regulation of transcription initiation from RNA polymerase II promoter | 2 | enriched | 1 | *TAF7* | 0.0031 | 0.4283 |
| GO:0035063 | nuclear speck organization | 4 | enriched | 1 | *DYRK3* | 0.0033 | 0.4437 |
| GO:0071569 | protein ufmylation | 5 | enriched | 1 | *UFL1* | 0.0034 | 0.4500 |
| GO:1990592 | protein K69-linked ufmylation | 5 | enriched | 1 | *UFL1* | 0.0034 | 0.4500 |
| GO:0046459 | short-chain fatty acid metabolic process | 2 | enriched | 1 | *ALDH5A1* | 0.0035 | 0.4592 |
| GO:0097284 | hepatocyte apoptotic process | 11 | enriched | 2 | *KRT18, KRT8* | 0.0035 | 0.4592 |
| GO:0035425 | autocrine signaling | 7 | enriched | 1 | *SERPINB3* | 0.0037 | 0.4708 |
| GO:0021520 | spinal cord motor neuron cell fate specification | 7 | enriched | 1 | *ISL2* | 0.0038 | 0.4773 |
| GO:0032465 | regulation of cytokinesis | 41 | enriched | 3 | *KIF13A, BIRC6, SETD2* | 0.0038 | 0.4773 |
| GO:1900103 | positive regulation of endoplasmic reticulum unfolded protein response | 5 | enriched | 1 | *PIK3R1* | 0.0039 | 0.4883 |
| GO:1902751 | positive regulation of cell cycle G2/M phase transition | 3 | enriched | 1 | *DYRK3* | 0.0040 | 0.4883 |
| GO:0048666 | neuron development | 47 | enriched | 6 | *GLI2, ISL2, TDP2, TENM3, TENM4, WNK1* | 0.0040 | 0.4890 |
| GO:0050794 | regulation of cellular process | 2 | enriched | 1 | *WNK1* | 0.0041 | 0.4890 |
| GO:0097022 | lymphocyte migration into lymph node | 2 | enriched | 1 | *WNK1* | 0.0041 | 0.4890 |
| GO:0034622 | cellular protein-containing complex assembly | 22 | enriched | 2 | *DHX9, MBD2* | 0.0042 | 0.4907 |
| GO:0010951 | negative regulation of endopeptidase activity | 140 | enriched | 8 | *BIRC6, SERPINB12, SERPINB13, SERPINB3, SERPINB4, SERPINB7, SERPINE3, SPOCK3* | 0.0045 | 0.5112 |
| GO:0048864 | stem cell development | 5 | enriched | 1 | *SETD2* | 0.0045 | 0.5112 |
| GO:2000171 | negative regulation of dendrite development | 5 | enriched | 1 | *KIAA0319* | 0.0044 | 0.5112 |
| GO:0098883 | synapse pruning | 8 | enriched | 3 | *C1QA, C1QB, C1QC* | 0.0046 | 0.5264 |
| GO:0007018 | microtubule-based movement | 78 | enriched | 8 | *KIF13A, DNAH11, BICD2, DNAH14, KIF17, KIF9, STARD9, WDR34* | 0.0048 | 0.5428 |
| GO:0006107 | oxaloacetate metabolic process | 8 | enriched | 2 | *MDH1B, PCK1* | 0.0050 | 0.5565 |
| GO:0061088 | regulation of sequestering of zinc ion | 5 | enriched | 1 | *SLC30A2* | 0.0051 | 0.5676 |
| GO:0046726 | positive regulation by virus of viral protein levels in host cell | 4 | enriched | 1 | *STAU1* | 0.0055 | 0.5911 |
| GO:0097084 | vascular smooth muscle cell development | 7 | enriched | 1 | *SGCB* | 0.0054 | 0.5911 |
| GO:1904751 | positive regulation of protein localization to nucleolus | 5 | enriched | 1 | *PINX1* | 0.0055 | 0.5911 |
| GO:0034497 | protein localization to phagophore assembly site | 12 | enriched | 2 | *TRAPPC8, MFN2* | 0.0058 | 0.6184 |
| GO:2000373 | positive regulation of DNA topoisomerase (ATP-hydrolyzing) activity | 5 | enriched | 1 | *DHX9* | 0.0059 | 0.6188 |
| GO:0008594 | photoreceptor cell morphogenesis | 3 | enriched | 1 | *C8orf37* | 0.0060 | 0.6189 |
| GO:0050691 | regulation of defense response to virus by host | 7 | enriched | 2 | *DHX9, IL15* | 0.0061 | 0.6189 |
| GO:0071447 | cellular response to hydroperoxide | 7 | enriched | 2 | *CD36, TP53INP1* | 0.0061 | 0.6189 |
| GO:1902749 | regulation of cell cycle G2/M phase transition | 5 | enriched | 1 | *NEK10* | 0.0060 | 0.6189 |
| GO:0031344 | regulation of cell projection organization | 3 | enriched | 1 | *PIFO* | 0.0065 | 0.6379 |
| GO:0032259 | methylation | 54 | enriched | 4 | *AS3MT, PRDM8, SETD9, TPMT* | 0.0065 | 0.6379 |
| GO:0046040 | IMP metabolic process | 4 | enriched | 1 | *NT5C2* | 0.0065 | 0.6379 |
| GO:0048671 | negative regulation of collateral sprouting | 7 | enriched | 1 | *IFRD1* | 0.0065 | 0.6379 |
| GO:0048935 | peripheral nervous system neuron development | 10 | enriched | 2 | *ETV1, ISL2* | 0.0065 | 0.6379 |
| GO:0033633 | negative regulation of cell-cell adhesion mediated by integrin | 2 | enriched | 1 | *WNK1* | 0.0067 | 0.6418 |
| GO:1902510 | regulation of apoptotic DNA fragmentation | 3 | enriched | 1 | *APAF1* | 0.0067 | 0.6418 |
| GO:0009450 | gamma-aminobutyric acid catabolic process | 2 | enriched | 1 | *ALDH5A1* | 0.0068 | 0.6446 |
| GO:0070934 | CRD-mediated mRNA stabilization | 5 | enriched | 1 | *DHX9* | 0.0068 | 0.6446 |
| GO:0035630 | bone mineralization involved in bone maturation | 6 | enriched | 1 | *CCDC154* | 0.0070 | 0.6601 |
| GO:1905166 | negative regulation of lysosomal protein catabolic process | 4 | enriched | 2 | *ATP13A2, MGAT3* | 0.0071 | 0.6601 |
| GO:0006734 | NADH metabolic process | 7 | enriched | 1 | *MDH1B* | 0.0073 | 0.6604 |
| GO:0007006 | mitochondrial membrane organization | 4 | enriched | 1 | *MFN2* | 0.0072 | 0.6604 |
| GO:0007129 | synapsis | 21 | enriched | 3 | *TEX15, CCNE2, SUN1* | 0.0072 | 0.6604 |
| GO:0060628 | regulation of ER to Golgi vesicle-mediated transport | 6 | enriched | 1 | *RINT1* | 0.0073 | 0.6604 |
| GO:0048102 | autophagic cell death | 7 | enriched | 1 | *TP53INP1* | 0.0074 | 0.6644 |
| GO:0042921 | glucocorticoid receptor signaling pathway | 5 | enriched | 1 | *NEDD4* | 0.0075 | 0.6669 |
| GO:1903432 | regulation of TORC1 signaling | 5 | enriched | 1 | *DYRK3* | 0.0078 | 0.6873 |
| GO:0010659 | cardiac muscle cell apoptotic process | 4 | enriched | 1 | *APAF1* | 0.0080 | 0.7040 |
| GO:0045661 | regulation of myoblast differentiation | 6 | enriched | 1 | *CAPN3* | 0.0081 | 0.7040 |
| GO:0072673 | lamellipodium morphogenesis | 5 | enriched | 1 | *WASF2* | 0.0081 | 0.7053 |
| GO:0010457 | centriole-centriole cohesion | 11 | enriched | 2 | *RTTN, CROCC* | 0.0085 | 0.7168 |
| GO:0021796 | cerebral cortex regionalization | 7 | enriched | 1 | *EMX2* | 0.0084 | 0.7168 |
| GO:0034115 | negative regulation of heterotypic cell-cell adhesion | 10 | enriched | 2 | *IL1RN, WNK1* | 0.0084 | 0.7168 |
| GO:0006195 | purine nucleotide catabolic process | 13 | enriched | 2 | *NT5C2, XDH* | 0.0087 | 0.7214 |
| GO:0009946 | proximal/distal axis specification | 1 | enriched | 1 | *SIX3* | 0.0087 | 0.7214 |
| GO:0051301 | cell division | 342 | enriched | 13 | *KIF13A, BIRC6, BRE, CCNE2, CDC27, DYRK3, HAUS2, MARK4, MPLKIP, RCC2, SKA3, UBE2I, USP39* | 0.0086 | 0.7214 |
| GO:0023016 | signal transduction by trans-phosphorylation | 3 | enriched | 1 | *WNK1* | 0.0089 | 0.7318 |
| GO:0006986 | response to unfolded protein | 51 | enriched | 3 | *DNAJB4, FAF2, MFN2* | 0.0090 | 0.7358 |
| GO:0018125 | peptidyl-cysteine methylation | 3 | enriched | 1 | *RAB6A* | 0.0091 | 0.7385 |
| GO:2000767 | positive regulation of cytoplasmic translation | 7 | enriched | 1 | *DHX9* | 0.0092 | 0.7456 |
| GO:0045453 | bone resorption | 24 | enriched | 2 | *CCDC154, PTH1R* | 0.0095 | 0.7632 |
| GO:0032258 | cytoplasm to vacuole transport by the Cvt pathway | 2 | enriched | 1 | *TRAPPC8* | 0.0100 | 0.7681 |
| GO:0035549 | positive regulation of interferon-beta secretion | 5 | enriched | 1 | *DHX9* | 0.0100 | 0.7681 |
| GO:0035617 | stress granule disassembly | 5 | enriched | 1 | *DYRK3* | 0.0097 | 0.7681 |
| GO:0055001 | muscle cell development | 4 | enriched | 1 | *NFATC4* | 0.0099 | 0.7681 |
| GO:0060117 | auditory receptor cell development | 6 | enriched | 1 | *SLC4A7* | 0.0097 | 0.7681 |
| GO:1902065 | response to L-glutamate | 6 | enriched | 1 | *UFL1* | 0.0100 | 0.7681 |
| GO:1904637 | cellular response to ionomycin | 4 | enriched | 1 | *NFATC4* | 0.0099 | 0.7681 |
| GO:0034340 | response to type I interferon | 7 | enriched | 1 | *SETD2* | 0.0104 | 0.7830 |
| GO:0035333 | Notch receptor processing, ligand-dependent | 7 | enriched | 1 | *PSEN2* | 0.0103 | 0.7830 |
| GO:1900180 | regulation of protein localization to nucleus | 6 | enriched | 1 | *ATP13A2* | 0.0104 | 0.7830 |
| GO:0001956 | positive regulation of neurotransmitter secretion | 7 | enriched | 2 | *BAIAP3, STX1A* | 0.0107 | 0.7884 |
| GO:0016458 | gene silencing | 7 | enriched | 1 | *SCMH1* | 0.0108 | 0.7884 |
| GO:0043538 | regulation of actin phosphorylation | 1 | enriched | 1 | *TWF1* | 0.0106 | 0.7884 |
| GO:0080135 | regulation of cellular response to stress | 6 | enriched | 2 | *ADCY8, DYRK3* | 0.0107 | 0.7884 |
| GO:1904894 | positive regulation of receptor signaling pathway via STAT | 4 | enriched | 2 | *IL7R, MGAT5* | 0.0105 | 0.7884 |
| GO:0010637 | negative regulation of mitochondrial fusion | 7 | enriched | 1 | *MUL1* | 0.0110 | 0.8009 |
| GO:0001667 | ameboidal-type cell migration | 6 | enriched | 1 | *WASF2* | 0.0111 | 0.8011 |
| GO:0009404 | toxin metabolic process | 7 | enriched | 1 | *AS3MT* | 0.0113 | 0.8050 |
| GO:0071420 | cellular response to histamine | 8 | enriched | 3 | *DIAPH1, GABRB2, GABRB3* | 0.0113 | 0.8050 |
| GO:0090114 | COPII-coated vesicle budding | 5 | enriched | 1 | *SEC31B* | 0.0114 | 0.8050 |
| GO:0010960 | magnesium ion homeostasis | 7 | enriched | 1 | *CNNM2* | 0.0117 | 0.8176 |
| GO:0046085 | adenosine metabolic process | 6 | enriched | 1 | *NT5C2* | 0.0117 | 0.8176 |
| GO:0097264 | self proteolysis | 6 | enriched | 1 | *CAPN3* | 0.0116 | 0.8176 |
| GO:1902570 | protein localization to nucleolus | 7 | enriched | 1 | *PINX1* | 0.0118 | 0.8184 |
| GO:0016050 | vesicle organization | 13 | enriched | 2 | *SAR1A, ZFYVE16* | 0.0120 | 0.8284 |
| GO:0038116 | chemokine (C-C motif) ligand 21 signaling pathway | 4 | enriched | 1 | *WNK1* | 0.0123 | 0.8370 |
| GO:0043266 | regulation of potassium ion transport | 9 | enriched | 1 | *OR13F1* | 0.0123 | 0.8370 |
| GO:0033145 | positive regulation of intracellular steroid hormone receptor signaling pathway | 1 | enriched | 1 | *UBE2I* | 0.0124 | 0.8378 |
| GO:1903755 | positive regulation of SUMO transferase activity | 1 | enriched | 1 | *UBE2I* | 0.0124 | 0.8378 |
| GO:0010828 | positive regulation of glucose transmembrane transport | 7 | enriched | 1 | *BRAF* | 0.0128 | 0.8398 |
| GO:0042276 | error-prone translesion synthesis | 20 | enriched | 2 | *POLI, RFC2* | 0.0128 | 0.8398 |
| GO:0051972 | regulation of telomerase activity | 6 | enriched | 1 | *PINX1* | 0.0127 | 0.8398 |
| GO:0061734 | parkin-mediated stimulation of mitophagy in response to mitochondrial depolarization | 5 | enriched | 1 | *MFN2* | 0.0128 | 0.8398 |
| GO:1901016 | regulation of potassium ion transmembrane transporter activity | 6 | enriched | 2 | *NEDD4, NEDD4L* | 0.0126 | 0.8398 |
| GO:0018023 | peptidyl-lysine trimethylation | 8 | enriched | 1 | *SETD2* | 0.0134 | 0.8656 |
| GO:0050774 | negative regulation of dendrite morphogenesis | 9 | enriched | 1 | *NFATC4* | 0.0133 | 0.8656 |
| GO:0060760 | positive regulation of response to cytokine stimulus | 7 | enriched | 1 | *DHX9* | 0.0134 | 0.8656 |
| GO:0000296 | spermine transport | 1 | enriched | 1 | *TAF7* | 0.0141 | 0.9020 |
| GO:0044691 | tooth eruption | 7 | enriched | 1 | *CCDC154* | 0.0142 | 0.9020 |
| GO:2000650 | negative regulation of sodium ion transmembrane transporter activity | 9 | enriched | 2 | *NEDD4, NEDD4L* | 0.0144 | 0.9135 |
| GO:0007195 | adenylate cyclase-inhibiting dopamine receptor signaling pathway | 9 | enriched | 1 | *OR13F1* | 0.0147 | 0.9279 |
| GO:0001960 | negative regulation of cytokine-mediated signaling pathway | 8 | enriched | 1 | *IL36RN* | 0.0150 | 0.9290 |
| GO:0003404 | optic vesicle morphogenesis | 2 | enriched | 1 | *SIX3* | 0.0151 | 0.9290 |
| GO:0006083 | acetate metabolic process | 1 | enriched | 1 | *ALDH5A1* | 0.0153 | 0.9290 |
| GO:0006681 | galactosylceramide metabolic process | 1 | enriched | 1 | *ALDH5A1* | 0.0153 | 0.9290 |
| GO:0010830 | regulation of myotube differentiation | 5 | enriched | 1 | *HDAC5* | 0.0153 | 0.9290 |
| GO:0038001 | paracrine signaling | 7 | enriched | 1 | *SERPINB3* | 0.0153 | 0.9290 |
| GO:1900246 | positive regulation of RIG-I signaling pathway | 8 | enriched | 1 | *PUM2* | 0.0153 | 0.9290 |
| GO:1902894 | negative regulation of pri-miRNA transcription by RNA polymerase II | 13 | enriched | 2 | *LILRB4, NFATC4* | 0.0151 | 0.9290 |
| GO:2001235 | positive regulation of apoptotic signaling pathway | 22 | enriched | 2 | *APAF1, NFATC4* | 0.0155 | 0.9310 |
| GO:0051983 | regulation of chromosome segregation | 8 | enriched | 1 | *PUM2* | 0.0155 | 0.9317 |
| GO:0010793 | regulation of mRNA export from nucleus | 6 | enriched | 1 | *SETD2* | 0.0157 | 0.9324 |
| GO:0018406 | protein C-linked glycosylation via 2'-alpha-mannosyl-L-tryptophan | 5 | enriched | 1 | *DPY19L4* | 0.0157 | 0.9324 |
| GO:1901299 | negative regulation of hydrogen peroxide-mediated programmed cell death | 2 | enriched | 1 | *MET* | 0.0165 | 0.9744 |
| GO:0000018 | regulation of DNA recombination | 6 | enriched | 1 | *IL7R* | 0.0387 | 0.9920 |
| GO:0000023 | maltose metabolic process | 3 | enriched | 1 | *GANC* | 0.0472 | 0.9920 |
| GO:0000045 | autophagosome assembly | 56 | enriched | 3 | *TRAPPC8, MAP1LC3B, TP53INP1* | 0.0319 | 0.9920 |
| GO:0000350 | generation of catalytic spliceosome for second transesterification step | 2 | enriched | 1 | *PRPF18* | 0.0492 | 0.9920 |
| GO:0000380 | alternative mRNA splicing, via spliceosome | 17 | enriched | 3 | *CDK13, CELF4, DHX9* | 0.0401 | 0.9920 |
| GO:0001763 | morphogenesis of a branching structure | 10 | enriched | 1 | *SETD2* | 0.0305 | 0.9920 |
| GO:0001963 | synaptic transmission, dopaminergic | 15 | enriched | 1 | *OR13F1* | 0.0410 | 0.9920 |
| GO:0002082 | regulation of oxidative phosphorylation | 11 | enriched | 1 | *COX7A2L* | 0.0344 | 0.9920 |
| GO:0003197 | endocardial cushion development | 9 | enriched | 2 | *GATA4, NEDD4* | 0.0304 | 0.9920 |
| GO:0006108 | malate metabolic process | 8 | enriched | 1 | *MDH1B* | 0.0169 | 0.9920 |
| GO:0006120 | mitochondrial electron transport, NADH to ubiquinone | 49 | enriched | 2 | *NDUFB2, NDUFB8* | 0.0338 | 0.9920 |
| GO:0006261 | DNA-dependent DNA replication | 22 | enriched | 2 | *BAZ1A, RFC2* | 0.0239 | 0.9920 |
| GO:0006346 | methylation-dependent chromatin silencing | 10 | enriched | 1 | *MBD2* | 0.0271 | 0.9920 |
| GO:0006353 | DNA-templated transcription, termination | 10 | enriched | 1 | *DHX9* | 0.0424 | 0.9920 |
| GO:0006468 | protein phosphorylation | 476 | enriched | 22 | *BRAF, FER, NEK10, RPS6KA2, ADCK2, ADCK3, BIRC6, CCNE2, CDC42BPA, CDK13, DYRK3, FASTKD2, FYN, MAP3K1, MAPK10, MAPKAPK2, MARK4, MLKL, PIK3R1, TGFBR2, TRIB2, WNK1* | 0.0172 | 0.9920 |
| GO:0006650 | glycerophospholipid metabolic process | 5 | enriched | 1 | *ALDH5A1* | 0.0188 | 0.9920 |
| GO:0006890 | retrograde vesicle-mediated transport, Golgi to endoplasmic reticulum | 81 | enriched | 4 | *RINT1, BICD2, NBAS, RAB6A* | 0.0347 | 0.9920 |
| GO:0006903 | vesicle targeting | 8 | enriched | 1 | *SNAP23* | 0.0172 | 0.9920 |
| GO:0006995 | cellular response to nitrogen starvation | 12 | enriched | 1 | *MAP1LC3B* | 0.0340 | 0.9920 |
| GO:0007091 | metaphase/anaphase transition of mitotic cell cycle | 6 | enriched | 1 | *CDC27* | 0.0194 | 0.9920 |
| GO:0007155 | cell adhesion | 447 | enriched | 31 | *PCDHGA1, PCDHGA2, PCDHGA3, PCDHGA4, PCDHGA5, PCDHGB1, PCDHGB2, PCDHGB3, COL15A1, FER, PCDHGA10, PCDHGA11, PCDHGA12, PCDHGA6, PCDHGA7, PCDHGA8, PCDHGA9, PCDHGB4, PCDHGB6, PCDHGB7, PCDHGC3, PCDHGC4, PCDHGC5, BCAM, CD36, CTNND2, FAM65B, GRHL2, ITGB8, PLXNC1, SEMA5A* | 0.0424 | 0.9920 |
| GO:0007165 | signal transduction | 974 | depleted | 26 | *COL15A1, ESR1, RCAN1, RPS6KA2, RSU1, ADCY8, ARHGAP28, BCAM, BRE, CBLC, CTNND2, FAM19A5, GABRB2, GABRB3, GPR123, IL15, IL7R, LILRB1, LILRB4, MAPK10, MET, TENM3, TENM4, TRIM63, WNT8B, ZFYVE16* | 0.0231 | 0.9920 |
| GO:0007188 | adenylate cyclase-modulating G protein-coupled receptor signaling pathway | 61 | enriched | 4 | *GNAO1, OR13F1, PTH1R, WASF2* | 0.0182 | 0.9920 |
| GO:0007220 | Notch receptor processing | 9 | enriched | 1 | *PSEN2* | 0.0227 | 0.9920 |
| GO:0008053 | mitochondrial fusion | 16 | enriched | 2 | *FAM73A, MFN2* | 0.0274 | 0.9920 |
| GO:0008635 | activation of cysteine-type endopeptidase activity involved in apoptotic process by cytochrome c | 7 | enriched | 1 | *APAF1* | 0.0223 | 0.9920 |
| GO:0009056 | catabolic process | 9 | enriched | 1 | *TMEM150A* | 0.0188 | 0.9920 |
| GO:0009268 | response to pH | 9 | enriched | 1 | *CLCN7* | 0.0383 | 0.9920 |
| GO:0009408 | response to heat | 43 | enriched | 2 | *DNAJB4, TP53INP1* | 0.0438 | 0.9920 |
| GO:0010501 | RNA secondary structure unwinding | 8 | enriched | 1 | *DHX9* | 0.0271 | 0.9920 |
| GO:0010762 | regulation of fibroblast migration | 11 | enriched | 2 | *FER, RCC2* | 0.0355 | 0.9920 |
| GO:0010768 | negative regulation of transcription from RNA polymerase II promoter in response to UV-induced DNA damage | 2 | enriched | 1 | *NEDD4* | 0.0193 | 0.9920 |
| GO:0010950 | positive regulation of endopeptidase activity | 10 | enriched | 1 | *SERPINB3* | 0.0180 | 0.9920 |
| GO:0014016 | neuroblast differentiation | 3 | enriched | 1 | *SIX3* | 0.0381 | 0.9920 |
| GO:0014063 | negative regulation of serotonin secretion | 4 | enriched | 2 | *HTR1B, LILRB1* | 0.0317 | 0.9920 |
| GO:0017185 | peptidyl-lysine hydroxylation | 6 | enriched | 1 | *PLOD1* | 0.0286 | 0.9920 |
| GO:0018026 | peptidyl-lysine monomethylation | 10 | enriched | 1 | *SETD2* | 0.0312 | 0.9920 |
| GO:0019262 | N-acetylneuraminate catabolic process | 6 | enriched | 1 | *NPL* | 0.0182 | 0.9920 |
| GO:0021797 | forebrain anterior/posterior pattern specification | 4 | enriched | 1 | *SIX3* | 0.0286 | 0.9920 |
| GO:0030239 | myofibril assembly | 12 | enriched | 1 | *CAPN3* | 0.0468 | 0.9920 |
| GO:0031125 | rRNA 3'-end processing | 1 | enriched | 1 | *ERI1* | 0.0292 | 0.9920 |
| GO:0031167 | rRNA methylation | 16 | enriched | 3 | *C7orf60, TFB1M, WBSCR22* | 0.0221 | 0.9920 |
| GO:0031547 | brain-derived neurotrophic factor receptor signaling pathway | 5 | enriched | 1 | *NFATC4* | 0.0181 | 0.9920 |
| GO:0032330 | regulation of chondrocyte differentiation | 10 | enriched | 1 | *GLG1* | 0.0443 | 0.9920 |
| GO:0032434 | regulation of proteasomal ubiquitin-dependent protein catabolic process | 11 | enriched | 1 | *UFL1* | 0.0367 | 0.9920 |
| GO:0032609 | interferon-gamma production | 9 | enriched | 2 | *IL12RB2, LILRB1* | 0.0412 | 0.9920 |
| GO:0032700 | negative regulation of interleukin-17 production | 11 | enriched | 1 | *IL36RN* | 0.0235 | 0.9920 |
| GO:0032727 | positive regulation of interferon-alpha production | 12 | enriched | 1 | *SETD2* | 0.0491 | 0.9920 |
| GO:0032760 | positive regulation of tumor necrosis factor production | 50 | enriched | 4 | *ARFGEF2, CD36, NFATC4, PIK3R1* | 0.0411 | 0.9920 |
| GO:0032781 | positive regulation of ATPase activity | 40 | enriched | 3 | *DNAJB4, MYL3, MYL4* | 0.0421 | 0.9920 |
| GO:0032981 | mitochondrial respiratory chain complex I assembly | 64 | enriched | 3 | *NDUFAF6, NDUFB2, NDUFB8* | 0.0291 | 0.9920 |
| GO:0033045 | regulation of sister chromatid segregation | 1 | enriched | 1 | *RMI2* | 0.0222 | 0.9920 |
| GO:0033157 | regulation of intracellular protein transport | 7 | enriched | 1 | *ATP13A2* | 0.0183 | 0.9920 |
| GO:0033173 | calcineurin-NFAT signaling cascade | 12 | enriched | 3 | *RCAN1, NFATC2, NFATC4* | 0.0230 | 0.9920 |
| GO:0033234 | negative regulation of protein sumoylation | 11 | enriched | 1 | *CAPN3* | 0.0282 | 0.9920 |
| GO:0033235 | positive regulation of protein sumoylation | 10 | enriched | 2 | *MUL1, PIAS1* | 0.0289 | 0.9920 |
| GO:0033555 | multicellular organismal response to stress | 8 | enriched | 1 | *KIAA0319* | 0.0189 | 0.9920 |
| GO:0034058 | endosomal vesicle fusion | 8 | enriched | 2 | *VPS39, VPS8* | 0.0246 | 0.9920 |
| GO:0034976 | response to endoplasmic reticulum stress | 73 | enriched | 4 | *PPP2CB, NHLRC1, PIK3R1, UFL1* | 0.0351 | 0.9920 |
| GO:0035459 | vesicle cargo loading | 13 | enriched | 1 | *KIF13A* | 0.0465 | 0.9920 |
| GO:0035548 | negative regulation of interferon-beta secretion | 1 | enriched | 1 | *LILRB1* | 0.0239 | 0.9920 |
| GO:0042147 | retrograde transport, endosome to Golgi | 74 | enriched | 4 | *BAIAP3, DENND2A, RAB6A, TMEM87A* | 0.0471 | 0.9920 |
| GO:0042692 | muscle cell differentiation | 13 | enriched | 2 | *RORA, IFRD1* | 0.0396 | 0.9920 |
| GO:0042711 | maternal behavior | 12 | enriched | 1 | *MBD2* | 0.0487 | 0.9920 |
| GO:0042987 | amyloid precursor protein catabolic process | 10 | enriched | 1 | *PSEN2* | 0.0282 | 0.9920 |
| GO:0043007 | maintenance of rDNA | 2 | enriched | 1 | *RMI2* | 0.0429 | 0.9920 |
| GO:0043405 | regulation of MAP kinase activity | 12 | enriched | 1 | *TRIB2* | 0.0414 | 0.9920 |
| GO:0044030 | regulation of DNA methylation | 12 | enriched | 2 | *GRHL2, MBD2* | 0.0288 | 0.9920 |
| GO:0044806 | G-quadruplex DNA unwinding | 7 | enriched | 1 | *DHX9* | 0.0181 | 0.9920 |
| GO:0045671 | negative regulation of osteoclast differentiation | 23 | enriched | 2 | *LILRB4, PIK3R1* | 0.0367 | 0.9920 |
| GO:0045824 | negative regulation of innate immune response | 11 | enriched | 1 | *MUL1* | 0.0318 | 0.9920 |
| GO:0046580 | negative regulation of Ras protein signal transduction | 26 | enriched | 2 | *PPP2CB, MFN2* | 0.0203 | 0.9920 |
| GO:0046636 | negative regulation of alpha-beta T cell activation | 2 | enriched | 1 | *LILRB1* | 0.0486 | 0.9920 |
| GO:0046833 | positive regulation of RNA export from nucleus | 7 | enriched | 1 | *DHX9* | 0.0176 | 0.9920 |
| GO:0048048 | embryonic eye morphogenesis | 9 | enriched | 1 | *MFAP2* | 0.0237 | 0.9920 |
| GO:0048148 | behavioral response to cocaine | 17 | enriched | 2 | *HOMER1, OR13F1* | 0.0497 | 0.9920 |
| GO:0048560 | establishment of anatomical structure orientation | 1 | enriched | 1 | *TTC8* | 0.0343 | 0.9920 |
| GO:0048665 | neuron fate specification | 16 | enriched | 2 | *EYA1, ISL2* | 0.0284 | 0.9920 |
| GO:0048814 | regulation of dendrite morphogenesis | 21 | enriched | 3 | *KNDC1, NEDD4, NEDD4L* | 0.0336 | 0.9920 |
| GO:0048841 | regulation of axon extension involved in axon guidance | 2 | enriched | 2 | *SEMA3A, PLXNA4* | 0.0341 | 0.9920 |
| GO:0051028 | mRNA transport | 42 | enriched | 3 | *BICD2, DHX9, LRPPRC* | 0.0173 | 0.9920 |
| GO:0051493 | regulation of cytoskeleton organization | 30 | enriched | 2 | *DIAPH1, NEXN* | 0.0474 | 0.9920 |
| GO:0051497 | negative regulation of stress fiber assembly | 26 | enriched | 3 | *ARHGAP28, MET, WASF2* | 0.0326 | 0.9920 |
| GO:0051967 | negative regulation of synaptic transmission, glutamatergic | 14 | enriched | 2 | *HTR1B, OR13F1* | 0.0288 | 0.9920 |
| GO:0052548 | regulation of endopeptidase activity | 8 | enriched | 1 | *ATP13A2* | 0.0187 | 0.9920 |
| GO:0055013 | cardiac muscle cell development | 9 | enriched | 1 | *SGCB* | 0.0237 | 0.9920 |
| GO:0060039 | pericardium development | 9 | enriched | 1 | *SETD2* | 0.0359 | 0.9920 |
| GO:0060158 | phospholipase C-activating dopamine receptor signaling pathway | 13 | enriched | 1 | *OR13F1* | 0.0400 | 0.9920 |
| GO:0060396 | growth hormone receptor signaling pathway | 12 | enriched | 1 | *PIK3R1* | 0.0374 | 0.9920 |
| GO:0060712 | spongiotrophoblast layer development | 8 | enriched | 2 | *BIRC6, ZFAT* | 0.0230 | 0.9920 |
| GO:0060732 | positive regulation of inositol phosphate biosynthetic process | 8 | enriched | 1 | *PTH1R* | 0.0467 | 0.9920 |
| GO:0060989 | lipid tube assembly involved in organelle fusion | 1 | enriched | 1 | *PCDHGA3* | 0.0358 | 0.9920 |
| GO:0061061 | muscle structure development | 10 | enriched | 1 | *CAPN3* | 0.0319 | 0.9920 |
| GO:0061074 | regulation of neural retina development | 3 | enriched | 1 | *SIX3* | 0.0177 | 0.9920 |
| GO:0070198 | protein localization to chromosome, telomeric region | 10 | enriched | 1 | *PINX1* | 0.0455 | 0.9920 |
| GO:0070269 | pyroptosis | 10 | enriched | 1 | *DHX9* | 0.0414 | 0.9920 |
| GO:0070495 | negative regulation of thrombin-activated receptor signaling pathway | 3 | enriched | 1 | *MET* | 0.0183 | 0.9920 |
| GO:0071048 | nuclear retention of unspliced pre-mRNA at the site of transcription | 2 | enriched | 1 | *PRPF18* | 0.0499 | 0.9920 |
| GO:0071285 | cellular response to lithium ion | 14 | enriched | 2 | *FABP4, NFATC4* | 0.0318 | 0.9920 |
| GO:0071801 | regulation of podosome assembly | 3 | enriched | 1 | *KIF9* | 0.0288 | 0.9920 |
| GO:0072383 | plus-end-directed vesicle transport along microtubule | 7 | enriched | 1 | *KIF13A* | 0.0226 | 0.9920 |
| GO:0072432 | response to G1 DNA damage checkpoint signaling | 1 | enriched | 1 | *APAF1* | 0.0316 | 0.9920 |
| GO:0090110 | COPII-coated vesicle cargo loading | 13 | enriched | 2 | *SAR1A, SEC31B* | 0.0241 | 0.9920 |
| GO:0090150 | establishment of protein localization to membrane | 9 | enriched | 1 | *BRAF* | 0.0415 | 0.9920 |
| GO:0097402 | neuroblast migration | 2 | enriched | 1 | *SIX3* | 0.0213 | 0.9920 |
| GO:1900025 | negative regulation of substrate adhesion-dependent cell spreading | 13 | enriched | 3 | *EFNA5, KANK1, RCC2* | 0.0401 | 0.9920 |
| GO:1900027 | regulation of ruffle assembly | 10 | enriched | 1 | *RCC2* | 0.0364 | 0.9920 |
| GO:1900364 | negative regulation of mRNA polyadenylation | 8 | enriched | 1 | *ZC3H14* | 0.0205 | 0.9920 |
| GO:1901838 | positive regulation of transcription of nucleolar large rRNA by RNA polymerase I | 10 | enriched | 1 | *IPPK* | 0.0419 | 0.9920 |
| GO:1901987 | regulation of cell cycle phase transition | 3 | enriched | 1 | *SIX3* | 0.0333 | 0.9920 |
| GO:1902692 | regulation of neuroblast proliferation | 3 | enriched | 1 | *SIX3* | 0.0361 | 0.9920 |
| GO:1902741 | positive regulation of interferon-alpha secretion | 9 | enriched | 1 | *DHX9* | 0.0344 | 0.9920 |
| GO:1903038 | negative regulation of leukocyte cell-cell adhesion | 6 | enriched | 1 | *WNK1* | 0.0488 | 0.9920 |
| GO:1903288 | positive regulation of potassium ion import across plasma membrane | 7 | enriched | 1 | *WNK1* | 0.0438 | 0.9920 |
| GO:1903608 | protein localization to cytoplasmic stress granule | 7 | enriched | 1 | *DHX9* | 0.0193 | 0.9920 |
| GO:1904357 | negative regulation of telomere maintenance via telomere lengthening | 10 | enriched | 1 | *PINX1* | 0.0376 | 0.9920 |
| GO:1905461 | positive regulation of vascular associated smooth muscle cell apoptotic process | 7 | enriched | 1 | *MFN2* | 0.0424 | 0.9920 |
| GO:2000001 | regulation of DNA damage checkpoint | 8 | enriched | 1 | *RFWD3* | 0.0215 | 0.9920 |
| GO:2000651 | positive regulation of sodium ion transmembrane transporter activity | 8 | enriched | 1 | *WNK1* | 0.0452 | 0.9920 |
| GO:2001193 | positive regulation of gamma-delta T cell activation involved in immune response | 1 | enriched | 1 | *LILRB1* | 0.0239 | 0.9920 |
| GO:2001213 | negative regulation of vasculogenesis | 1 | enriched | 1 | *XDH* | 0.0497 | 0.9920 |
| GO:2001214 | positive regulation of vasculogenesis | 10 | enriched | 1 | *HIF1AN* | 0.0297 | 0.9920 |
| GO:2001224 | positive regulation of neuron migration | 14 | enriched | 2 | *SEMA3A, FBXO31* | 0.0265 | 0.9920 |
