# Supplementary material for: Adolescent anxiety and pain problems: A joint, genome-wide investigation and pathway-based analysis
Source: PLoS One. 2023 May 5;18(5):e0285263. doi: 10.1371/journal.pone.0285263 (PMC10162554; doi:10.1371/journal.pone.0285263)
Supplement: S7 Table — (DOCX) [file pone.0285263.s007.docx]

| **S7 Table. Overlapping enriched pathways between QLSCD_Mean Pain and QLSCD_Mean Anxiety (uncorrected p-value < 0.05).** | | | | | | | | | | |
| --- | --- | --- | --- | --- | --- | --- | --- | --- | --- | --- |
| **GO set ID** | **Description** | **Pathway size (nr of genes)** | **QLSCD_Mean Pain** | | | | **QLSCD_Mean Anxiety** | | | |
|  |  |  | **Enriched genes (nr.)** | **Genes** | ***p-value*** | **FDR** | **Enriched genes (nr.)** | **Genes** | ***p-value*** | **FDR** |
| GO:0000350 | generation of catalytic spliceosome for second transesterification step | 2 | 1 | *PRPF18* | 0.0387 | 0.9869 | 1 | *PRPF18* | 0.0492 | 0.9920 |
| GO:0001667 | ameboidal-type cell migration | 6 | 1 | *WASF2* | 0.0077 | 0.6860 | 1 | *WASF2* | 0.0111 | 0.8011 |
| GO:0001763 | morphogenesis of a branching structure | 10 | 1 | *SETD2* | 0.0237 | 0.9869 | 1 | *SETD2* | 0.0305 | 0.9920 |
| GO:0002553 | histamine secretion by mast cell | 3 | 1 | *SNAP23* | 0.0016 | 0.3451 | 1 | *SNAP23* | 0.0025 | 0.3880 |
| GO:0003197 | endocardial cushion development | 9 | 1 | *NEDD4* | 0.0248 | 0.9869 | 2 | *GATA4, NEDD4* | 0.0304 | 0.9920 |
| GO:0006105 | succinate metabolic process | 7 | 1 | *SDHB* | 0.0145 | 0.9466 | 2 | *ALDH5A1, SDHB* | 0.0008 | 0.3045 |
| GO:0006107 | oxaloacetate metabolic process | 8 | 1 | *MDH1B* | 0.0136 | 0.9148 | 2 | *MDH1B, PCK1* | 0.0050 | 0.5565 |
| GO:0006108 | malate metabolic process | 8 | 1 | *MDH1B* | 0.0128 | 0.8972 | 1 | *MDH1B* | 0.0169 | 0.9920 |
| GO:0006468 | protein phosphorylation | 476 | 24 | *PRKCB, ADCK3, AURKB, BRD4, CAMK2D, CCND3, CDC42BPA, FASTKD2, FER, HUS1, MAP3K1, MAP3K6, MARK4, MLKL, PHKG1, PHKG2, PIK3R1, RPS6KA2, SGK1, SIK2, TEC, TGFBR2, TSSK1B, TYK2* | 0.0246 | 0.9869 | 22 | *BRAF, FER, NEK10, RPS6KA2, ADCK2, ADCK3, BIRC6, CCNE2, CDC42BPA, CDK13, DYRK3, FASTKD2, FYN, MAP3K1, MAPK10, MAPKAPK2, MARK4, MLKL, PIK3R1, TGFBR2, TRIB2, WNK1* | 0.0172 | 0.9920 |
| GO:0006734 | NADH metabolic process | 7 | 1 | *MDH1B* | 0.0055 | 0.5706 | 1 | *MDH1B* | 0.0073 | 0.6604 |
| GO:0006903 | vesicle targeting | 8 | 1 | *SNAP23* | 0.0130 | 0.9020 | 1 | *SNAP23* | 0.0172 | 0.9920 |
| GO:0007018 | microtubule-based movement | 78 | 6 | *KIF13A, BICD2, KIF17, KIF6, KIF9, STARD9* | 0.0134 | 0.9044 | 8 | *KIF13A, DNAH11, BICD2, DNAH14, KIF17, KIF9, STARD9, WDR34* | 0.0048 | 0.5428 |
| GO:0007091 | metaphase/anaphase transition of mitotic cell cycle | 6 | 1 | *CDC27* | 0.0118 | 0.8543 | 1 | *CDC27* | 0.0194 | 0.9920 |
| GO:0007155 | cell adhesion | 447 | 43 | *BCAM, TINAG, ADAM12, CCR3, CHL1, COL28A1, COL8A1, EPHA3, FAM65B, FER, IBSP, ICAM3, ITGAL, ITGB8, MLLT4, NTM, PARD3, PCDH7, PCDHGA1, PCDHGA10, PCDHGA11, PCDHGA12, PCDHGA2, PCDHGA3, PCDHGA4, PCDHGA5, PCDHGA6, PCDHGA7, PCDHGA8, PCDHGA9, PCDHGB1, PCDHGB2, PCDHGB3, PCDHGB4, PCDHGB6, PCDHGB7, PCDHGC3, PCDHGC4, PCDHGC5, PLXNC1, SELP, STAB2, WISP2* | <0.0001 | 0.1823 | 31 | *PCDHGA1, PCDHGA2, PCDHGA3, PCDHGA4, PCDHGA5, PCDHGB1, PCDHGB2, PCDHGB3, COL15A1, FER, PCDHGA10, PCDHGA11, PCDHGA12, PCDHGA6, PCDHGA7, PCDHGA8, PCDHGA9, PCDHGB4, PCDHGB6, PCDHGB7, PCDHGC3, PCDHGC4, PCDHGC5, BCAM, CD36, CTNND2, FAM65B, GRHL2, ITGB8, PLXNC1, SEMA5A* | 0.0424 | 0.9920 |
| GO:0007156 | homophilic cell adhesion via plasma membrane adhesion molecules | 162 | 27 | *CDH13, ROBO2, DCHS2, IGSF21, PCDH7, PCDHGA1, PCDHGA10, PCDHGA11, PCDHGA12, PCDHGA2, PCDHGA3, PCDHGA4, PCDHGA5, PCDHGA6, PCDHGA7, PCDHGA8, PCDHGA9, PCDHGB1, PCDHGB2, PCDHGB3, PCDHGB4, PCDHGB6, PCDHGB7, PCDHGC3, PCDHGC4, PCDHGC5, PVRL2* | <0.0001 | 0.0012 | 26 | *PCDHGA1, PCDHGA2, PCDHGA3, PCDHGA4, PCDHGA5, PCDHGB1, PCDHGB2, PCDHGB3, PCDHGA10, PCDHGA11, PCDHGA12, PCDHGA6, PCDHGA7, PCDHGA8, PCDHGA9, PCDHGB4, PCDHGB6, PCDHGB7, PCDHGC3, PCDHGC4, PCDHGC5, ROBO2, DCHS2, IGSF21, NEXN, TENM3* | <0.0001 | <0.0001 |
| GO:0007220 | Notch receptor processing | 9 | 1 | *PSEN2* | 0.0175 | 0.9869 | 1 | *PSEN2* | 0.0227 | 0.9920 |
| GO:0008594 | photoreceptor cell morphogenesis | 3 | 1 | *C8orf37* | 0.0021 | 0.3500 | 1 | *C8orf37* | 0.0060 | 0.6189 |
| GO:0010457 | centriole-centriole cohesion | 11 | 2 | *CROCC, RTTN* | 0.0102 | 0.7753 | 2 | *RTTN, CROCC* | 0.0085 | 0.7168 |
| GO:0010569 | regulation of double-strand break repair via homologous recombination | 18 | 3 | *SETD2, TEX15, ZNF365* | 0.0009 | 0.2802 | 3 | *TEX15, ZNF365, SETD2* | 0.0006 | 0.3045 |
| GO:0010762 | regulation of fibroblast migration | 11 | 2 | *FER, RCC2* | 0.0287 | 0.9869 | 2 | *FER, RCC2* | 0.0355 | 0.9920 |
| GO:0010766 | negative regulation of sodium ion transport | 6 | 1 | *NEDD4* | 0.0382 | 0.9869 | 2 | *NEDD4, WNK1* | 0.0002 | 0.2505 |
| GO:0010768 | negative regulation of transcription from RNA polymerase II promoter in response to UV-induced DNA damage | 2 | 1 | *NEDD4* | 0.0102 | 0.7753 | 1 | *NEDD4* | 0.0193 | 0.9920 |
| GO:0010793 | regulation of mRNA export from nucleus | 6 | 2 | *AKAP8L, SETD2* | 0.0003 | 0.2802 | 1 | *SETD2* | 0.0157 | 0.9324 |
| GO:0010830a | regulation of myotube differentiation | 5 | 1 | *HDAC5* | 0.0494 | 0.9869 | 1 | *HDAC5* | 0.0153 | 0.9290 |
| GO:0015711 | organic anion transport | 14 | 4 | *SLC22A10, SLC22A24, SLC22A25, SLC22A9* | 0.0004 | 0.2802 | 4 | *SLC22A10, SLC22A24, SLC22A25, SLC22A9* | 0.0007 | 0.3045 |
| GO:0018023 | peptidyl-lysine trimethylation | 8 | 1 | *SETD2* | 0.0104 | 0.7856 | 1 | *SETD2* | 0.0134 | 0.8656 |
| GO:0018026 | peptidyl-lysine monomethylation | 10 | 1 | *SETD2* | 0.0243 | 0.9869 | 1 | *SETD2* | 0.0312 | 0.9920 |
| GO:0022904 | respiratory electron transport chain | 17 | 2 | *SDHB, IMMP2L* | 0.0491 | 0.9869 | 4 | *ALDH5A1, ETFA, IMMP2L, SDHB* | 0.0028 | 0.3880 |
| GO:0030003 | cellular cation homeostasis | 2 | 1 | *ATP13A2* | 0.0006 | 0.2802 | 1 | *ATP13A2* | 0.0008 | 0.3045 |
| GO:0031048 | chromatin silencing by small RNA | 3 | 1 | *FAM172A* | 0.0114 | 0.8377 | 1 | *ZNFX1* | 0.0014 | 0.3236 |
| GO:0031052 | chromosome breakage | 2 | 1 | *RFWD3* | 0.0009 | 0.2802 | 1 | *RFWD3* | 0.0012 | 0.3045 |
| GO:0031125 | rRNA 3'-end processing | 1 | 1 | *ERI1* | 0.0348 | 0.9869 | 1 | *ERI1* | 0.0292 | 0.9920 |
| GO:0031344 | regulation of cell projection organization | 3 | 1 | *PIFO* | 0.0050 | 0.5552 | 1 | *PIFO* | 0.0065 | 0.6379 |
| GO:0031547 | brain-derived neurotrophic factor receptor signaling pathway | 5 | 1 | *NFATC4* | 0.0046 | 0.5139 | 1 | *NFATC4* | 0.0181 | 0.9920 |
| GO:0031571 | mitotic G1 DNA damage checkpoint | 8 | 2 | *FBXO31, RFWD3* | 0.0048 | 0.5335 | 2 | *FBXO31, RFWD3* | <0.0001 | 0.0494 |
| GO:0032053 | ciliary basal body organization | 3 | 2 | *CROCC, RTTN* | 0.0001 | 0.2061 | 2 | *RTTN, CROCC* | 0.0001 | 0.1952 |
| GO:0032330 | regulation of chondrocyte differentiation | 10 | 1 | *GLG1* | 0.0358 | 0.9869 | 1 | *GLG1* | 0.0443 | 0.9920 |
| GO:0032465 | regulation of cytokinesis | 41 | 3 | *KIF13A, AURKB, SETD2* | 0.0037 | 0.4559 | 3 | *KIF13A, BIRC6, SETD2* | 0.0038 | 0.4773 |
| GO:0032727 | positive regulation of interferon-alpha production | 12 | 1 | *SETD2* | 0.0385 | 0.9869 | 1 | *SETD2* | 0.0491 | 0.9920 |
| GO:0033157 | regulation of intracellular protein transport | 7 | 1 | *ATP13A2* | 0.0126 | 0.8876 | 1 | *ATP13A2* | 0.0183 | 0.9920 |
| GO:0034340 | response to type I interferon | 7 | 1 | *SETD2* | 0.0079 | 0.6902 | 1 | *SETD2* | 0.0104 | 0.7830 |
| GO:0034644 | cellular response to UV | 49 | 4 | *NFATC4, AURKB, NEDD4, PIK3R1* | 0.0003 | 0.2802 | 4 | *NEDD4, NFATC4, PIK3R1, TP53INP1* | 0.0002 | 0.2554 |
| GO:0034728 | nucleosome organization | 3 | 1 | *SETD2* | 0.0016 | 0.3451 | 1 | *SETD2* | 0.0026 | 0.3880 |
| GO:0035333 | Notch receptor processing, ligand-dependent | 7 | 1 | *PSEN2* | 0.0078 | 0.6894 | 1 | *PSEN2* | 0.0103 | 0.7830 |
| GO:0035441 | cell migration involved in vasculogenesis | 2 | 1 | *SETD2* | 0.0009 | 0.2802 | 1 | *SETD2* | 0.0012 | 0.3045 |
| GO:0035459 | vesicle cargo loading | 13 | 1 | *KIF13A* | 0.0380 | 0.9869 | 1 | *KIF13A* | 0.0465 | 0.9920 |
| GO:0040020 | regulation of meiotic nuclear division | 5 | 2 | *GPR3, PDE3A* | 0.0017 | 0.3477 | 2 | *GPR3, PDE3A* | 0.0023 | 0.3718 |
| GO:0042921 | glucocorticoid receptor signaling pathway | 5 | 2 | *ARID1A, NEDD4* | <0.0001 | 0.0249 | 1 | *NEDD4* | 0.0075 | 0.6669 |
| GO:0042987 | amyloid precursor protein catabolic process | 10 | 1 | *PSEN2* | 0.0220 | 0.9869 | 1 | *PSEN2* | 0.0282 | 0.9920 |
| GO:0048048 | embryonic eye morphogenesis | 9 | 1 | *MFAP2* | 0.0184 | 0.9869 | 1 | *MFAP2* | 0.0237 | 0.9920 |
| GO:0048050 | post-embryonic eye morphogenesis | 3 | 1 | *MFAP2* | 0.0010 | 0.2802 | 1 | *MFAP2* | 0.0015 | 0.3240 |
| GO:0048332 | mesoderm morphogenesis | 2 | 1 | *SETD2* | 0.0008 | 0.2802 | 1 | *SETD2* | 0.0011 | 0.3045 |
| GO:0048814 | regulation of dendrite morphogenesis | 21 | 4 | *KNDC1, NEDD4, NEDD4L, RAP2A* | 0.0008 | 0.2802 | 3 | *KNDC1, NEDD4, NEDD4L* | 0.0336 | 0.9920 |
| GO:0048864 | stem cell development | 5 | 1 | *SETD2* | 0.0032 | 0.4260 | 1 | *SETD2* | 0.0045 | 0.5112 |
| GO:0050691 | regulation of defense response to virus by host | 7 | 1 | *RNF216* | 0.0150 | 0.9709 | 2 | *DHX9, IL15* | 0.0061 | 0.6189 |
| GO:0050774 | negative regulation of dendrite morphogenesis | 9 | 2 | *NFATC4, GORASP1* | <0.0001 | 0.0417 | 1 | *NFATC4* | 0.0133 | 0.8656 |
| GO:0050808 | synapse organization | 46 | 6 | *PPFIA2, C1QA, PCDHGC3, PCDHGC4, PCDHGC5, PPFIBP2* | 0.0001 | 0.2025 | 5 | *PCDHGC3, PCDHGC4, PCDHGC5, C1QA, CTNND2* | 0.0020 | 0.3661 |
| GO:0051497 | negative regulation of stress fiber assembly | 26 | 4 | *ARHGAP28, DLC1, MET, WASF2* | 0.0351 | 0.9869 | 3 | *ARHGAP28, MET, WASF2* | 0.0326 | 0.9920 |
| GO:0051972 | regulation of telomerase activity | 6 | 1 | *PINX1* | 0.0087 | 0.7426 | 1 | *PINX1* | 0.0127 | 0.8398 |
| GO:0051987 | positive regulation of attachment of spindle microtubules to kinetochore | 3 | 1 | *RCC2* | 0.0013 | 0.3093 | 1 | *RCC2* | 0.0019 | 0.3604 |
| GO:0052548 | regulation of endopeptidase activity | 8 | 1 | *ATP13A2* | 0.0139 | 0.9260 | 1 | *ATP13A2* | 0.0187 | 0.9920 |
| GO:0052746 | inositol phosphorylation | 2 | 1 | *IPPK* | 0.0009 | 0.2802 | 1 | *IPPK* | 0.0012 | 0.3045 |
| GO:0055001 | muscle cell development | 4 | 1 | *NFATC4* | 0.0023 | 0.3720 | 1 | *NFATC4* | 0.0099 | 0.7681 |
| GO:0055069 | zinc ion homeostasis | 5 | 2 | *ATP13A2, PARK2* | 0.0018 | 0.3477 | 1 | *ATP13A2* | 0.0027 | 0.3880 |
| GO:0060039 | pericardium development | 9 | 1 | *SETD2* | 0.0229 | 0.9869 | 1 | *SETD2* | 0.0359 | 0.9920 |
| GO:0060396 | growth hormone receptor signaling pathway | 12 | 1 | *PIK3R1* | 0.0304 | 0.9869 | 1 | *PIK3R1* | 0.0374 | 0.9920 |
| GO:0060669 | embryonic placenta morphogenesis | 6 | 1 | *SETD2* | 0.0053 | 0.5706 | 2 | *SETD2, ZNF568* | 0.0020 | 0.3661 |
| GO:0060732 | positive regulation of inositol phosphate biosynthetic process | 8 | 1 | *PTH1R* | 0.0371 | 0.9869 | 1 | *PTH1R* | 0.0467 | 0.9920 |
| GO:0060977 | coronary vasculature morphogenesis | 3 | 2 | *SGCD, SETD2* | 0.0004 | 0.2802 | 2 | *SETD2, SGCD* | 0.0008 | 0.3045 |
| GO:0061088 | regulation of sequestering of zinc ion | 5 | 1 | *SLC30A2* | 0.0037 | 0.4559 | 1 | *SLC30A2* | 0.0051 | 0.5676 |
| GO:0070198 | protein localization to chromosome, telomeric region | 10 | 1 | *PINX1* | 0.0329 | 0.9869 | 1 | *PINX1* | 0.0455 | 0.9920 |
| GO:0070495 | negative regulation of thrombin-activated receptor signaling pathway | 3 | 1 | *MET* | 0.0383 | 0.9869 | 1 | *MET* | 0.0183 | 0.9920 |
| GO:0071048 | nuclear retention of unspliced pre-mRNA at the site of transcription | 2 | 1 | *PRPF18* | 0.0388 | 0.9869 | 1 | *PRPF18* | 0.0499 | 0.9920 |
| GO:0071285 | cellular response to lithium ion | 14 | 2 | *NFATC4, FABP4* | 0.0124 | 0.8846 | 2 | *FABP4, NFATC4* | 0.0318 | 0.9920 |
| GO:0071287 | cellular response to manganese ion | 9 | 2 | *ATP13A2, PARK2* | 0.0125 | 0.8846 | 2 | *A3GALT2, ATP13A2* | 0.0001 | 0.1843 |
| GO:0071344 | diphosphate metabolic process | 2 | 1 | *PPA1* | 0.0008 | 0.2802 | 1 | *PPA1* | 0.0011 | 0.3045 |
| GO:0071801 | regulation of podosome assembly | 3 | 1 | *KIF9* | 0.0212 | 0.9869 | 1 | *KIF9* | 0.0288 | 0.9920 |
| GO:0072383 | plus-end-directed vesicle transport along microtubule | 7 | 1 | *KIF13A* | 0.0164 | 0.9869 | 1 | *KIF13A* | 0.0226 | 0.9920 |
| GO:0072385 | minus-end-directed organelle transport along microtubule | 4 | 1 | *BICD2* | 0.0028 | 0.4191 | 2 | *BICD2, RAB6A* | <0.0001 | 0.0403 |
| GO:0072393 | microtubule anchoring at microtubule organizing center | 2 | 1 | *BICD2* | 0.0010 | 0.2802 | 1 | *BICD2* | 0.0012 | 0.3045 |
| GO:0072673 | lamellipodium morphogenesis | 5 | 1 | *WASF2* | 0.0054 | 0.5706 | 1 | *WASF2* | 0.0081 | 0.7053 |
| GO:0097198 | histone H3-K36 trimethylation | 2 | 1 | *SETD2* | 0.0008 | 0.2802 | 1 | *SETD2* | 0.0011 | 0.3045 |
| GO:0097676 | histone H3-K36 dimethylation | 4 | 1 | *SETD2* | 0.0019 | 0.3477 | 1 | *SETD2* | 0.0027 | 0.3880 |
| GO:0098883 | synapse pruning | 8 | 3 | *C1QA, C1QB, C1QC* | 0.0038 | 0.4611 | 3 | *C1QA, C1QB, C1QC* | 0.0046 | 0.5264 |
| GO:1900025 | negative regulation of substrate adhesion-dependent cell spreading | 13 | 2 | *KANK1, RCC2* | 0.0478 | 0.9869 | 3 | *EFNA5, KANK1, RCC2* | 0.0401 | 0.9920 |
| GO:1900027 | regulation of ruffle assembly | 10 | 1 | *RCC2* | 0.0287 | 0.9869 | 1 | *RCC2* | 0.0364 | 0.9920 |
| GO:1900103 | positive regulation of endoplasmic reticulum unfolded protein response | 5 | 1 | *PIK3R1* | 0.0029 | 0.4231 | 1 | *PIK3R1* | 0.0039 | 0.4883 |
| GO:1900180 | regulation of protein localization to nucleus | 6 | 1 | *ATP13A2* | 0.0077 | 0.6860 | 1 | *ATP13A2* | 0.0104 | 0.7830 |
| GO:1901016 | regulation of potassium ion transmembrane transporter activity | 6 | 2 | *NEDD4, NEDD4L* | 0.0095 | 0.7750 | 2 | *NEDD4, NEDD4L* | 0.0126 | 0.8398 |
| GO:1901299 | negative regulation of hydrogen peroxide-mediated programmed cell death | 2 | 1 | *MET* | 0.0271 | 0.9869 | 1 | *MET* | 0.0165 | 0.9744 |
| GO:1901838 | positive regulation of transcription of nucleolar large rRNA by RNA polymerase I | 10 | 1 | *IPPK* | 0.0309 | 0.9869 | 1 | *IPPK* | 0.0419 | 0.9920 |
| GO:1902570 | protein localization to nucleolus | 7 | 1 | *PINX1* | 0.0090 | 0.7570 | 1 | *PINX1* | 0.0118 | 0.8184 |
| GO:1902775 | mitochondrial large ribosomal subunit assembly | 3 | 1 | *FASTKD2* | 0.0008 | 0.2802 | 1 | *FASTKD2* | 0.0011 | 0.3045 |
| GO:1902850 | microtubule cytoskeleton organization involved in mitosis | 2 | 1 | *SETD2* | 0.0009 | 0.2802 | 1 | *SETD2* | 0.0011 | 0.3045 |
| GO:1902894 | negative regulation of pri-miRNA transcription by RNA polymerase II | 13 | 2 | *NFATC4, NFIB* | 0.0381 | 0.9869 | 2 | *LILRB4, NFATC4* | 0.0151 | 0.9290 |
| GO:1903008 | organelle disassembly | 2 | 1 | *KIF9* | 0.0152 | 0.9821 | 2 | *DYRK3, KIF9* | 0.0020 | 0.3661 |
| GO:1904357 | negative regulation of telomere maintenance via telomere lengthening | 10 | 1 | *PINX1* | 0.0289 | 0.9869 | 1 | *PINX1* | 0.0376 | 0.9920 |
| GO:1904637 | cellular response to ionomycin | 4 | 1 | *NFATC4* | 0.0023 | 0.3720 | 1 | *NFATC4* | 0.0099 | 0.7681 |
| GO:1904714 | regulation of chaperone-mediated autophagy | 5 | 1 | *ATP13A2* | 0.0019 | 0.3477 | 1 | *ATP13A2* | 0.0024 | 0.3843 |
| GO:1904751 | positive regulation of protein localization to nucleolus | 5 | 1 | *PINX1* | 0.0040 | 0.4666 | 1 | *PINX1* | 0.0055 | 0.5911 |
| GO:1905037 | autophagosome organization | 3 | 1 | *ATP13A2* | 0.0009 | 0.2802 | 1 | *ATP13A2* | 0.0013 | 0.3154 |
| GO:1905123 | regulation of glucosylceramidase activity | 2 | 1 | *ATP13A2* | 0.0012 | 0.3093 | 1 | *ATP13A2* | 0.0018 | 0.3599 |
| GO:1905165 | regulation of lysosomal protein catabolic process | 2 | 1 | *ATP13A2* | 0.0006 | 0.2802 | 1 | *ATP13A2* | 0.0008 | 0.3045 |
| GO:1905166 | negative regulation of lysosomal protein catabolic process | 4 | 1 | *ATP13A2* | 0.0021 | 0.3500 | 2 | *ATP13A2, MGAT3* | 0.0071 | 0.6601 |
| GO:2000001 | regulation of DNA damage checkpoint | 8 | 1 | *RFWD3* | 0.0163 | 0.9869 | 1 | *RFWD3* | 0.0215 | 0.9920 |
| GO:2000297 | negative regulation of synapse maturation | 3 | 1 | *NFATC4* | 0.0008 | 0.2802 | 1 | *NFATC4* | 0.0022 | 0.3718 |
| GO:2000650 | negative regulation of sodium ion transmembrane transporter activity | 9 | 4 | *CAMK2D, NEDD4, NEDD4L, PCSK9* | 0.0134 | 0.9044 | 2 | *NEDD4, NEDD4L* | 0.0144 | 0.9135 |
| a Replication of overlapping enriched pathways between QNTS_Mean Pain and QNTS_Mean Anxiety (uncorrected p-value < 0.05). | | | | | | | | | | |
