# Supplementary material for: Adolescent anxiety and pain problems: A joint, genome-wide investigation and pathway-based analysis
Source: PLoS One. 2023 May 5;18(5):e0285263. doi: 10.1371/journal.pone.0285263 (PMC10162554; doi:10.1371/journal.pone.0285263)
Supplement: S8 Table — (DOCX) [file pone.0285263.s008.docx]

| **S8a Table. Results of the pathway-based analysis of Mean Pain (uncorrected *p-value* < 0.05).** | | | | | | | |
| --- | --- | --- | --- | --- | --- | --- | --- |
| **GO set ID** | **Description** | **Pathway size (nr of genes)** | **Status** | **Significant Genes (nr of)** | **Genes** | ***p-value*** | **FDR** |
| GO:0000086 | G2/M transition of mitotic cell cycle | 130 | enriched | 9 | *PPP2R1A, AKAP9, CCNY, CEP192, CNTRL, DYNLL1, FGFR1OP, HAUS2, PPP1R12B* | 0.0079 | 0.8319 |
| GO:0000454 | snoRNA guided rRNA pseudouridine synthesis | 3 | enriched | 1 | *NAF1* | 0.0138 | 0.9830 |
| GO:0000493 | box H/ACA snoRNP assembly | 2 | enriched | 1 | *NAF1* | 0.0093 | 0.8957 |
| GO:0001667 | ameboidal-type cell migration | 6 | enriched | 1 | *WASF2* | 0.0330 | 0.9830 |
| GO:0001704 | formation of primary germ layer | 3 | enriched | 1 | *ARID1A* | 0.0057 | 0.7864 |
| GO:0001913 | T cell mediated cytotoxicity | 11 | enriched | 2 | *CTSC, HLA-A* | 0.0193 | 0.9830 |
| GO:0001915 | negative regulation of T cell mediated cytotoxicity | 6 | enriched | 1 | *HLA-G* | 0.0322 | 0.9830 |
| GO:0001916 | positive regulation of T cell mediated cytotoxicity | 27 | enriched | 3 | *FAM49B, HLA-A, HLA-G* | 0.0069 | 0.7892 |
| GO:0001953 | negative regulation of cell-matrix adhesion | 19 | enriched | 2 | *PIK3R1, THBS1* | 0.0171 | 0.9830 |
| GO:0002476 | antigen processing and presentation of endogenous peptide antigen via MHC class Ib | 11 | enriched | 2 | *HLA-A, HLA-G* | 0.0009 | 0.4811 |
| GO:0002480 | antigen processing and presentation of exogenous peptide antigen via MHC class I, TAP-independent | 8 | enriched | 2 | *HLA-A, HLA-G* | 0.0003 | 0.3133 |
| GO:0002486 | antigen processing and presentation of endogenous peptide antigen via MHC class I via ER pathway, TAP-independent | 11 | enriched | 2 | *HLA-A, HLA-G* | 0.0009 | 0.4811 |
| GO:0002495 | antigen processing and presentation of peptide antigen via MHC class II | 2 | enriched | 1 | *marzo8* | 0.0068 | 0.7864 |
| GO:0002503 | peptide antigen assembly with MHC class II protein complex | 3 | enriched | 1 | *HLA-DRA* | 0.0067 | 0.7864 |
| GO:0002534 | cytokine production involved in inflammatory response | 3 | enriched | 1 | *SEH1L* | 0.0263 | 0.9830 |
| GO:0002544 | chronic inflammatory response | 8 | enriched | 2 | *THBS1, VCAM1* | 0.0121 | 0.9275 |
| GO:0002553 | histamine secretion by mast cell | 3 | enriched | 1 | *SNAP23* | 0.0068 | 0.7864 |
| GO:0002605 | negative regulation of dendritic cell antigen processing and presentation | 4 | enriched | 1 | *THBS1* | 0.0112 | 0.9124 |
| GO:0002645 | positive regulation of tolerance induction | 2 | enriched | 1 | *HLA-G* | 0.0050 | 0.7864 |
| GO:0002666 | positive regulation of T cell tolerance induction | 4 | enriched | 2 | *TGFBR2, HLA-G* | 0.0029 | 0.7489 |
| GO:0002762 | negative regulation of myeloid leukocyte differentiation | 4 | enriched | 3 | *NME1, NME1-NME2, NME2* | 0.0239 | 0.9830 |
| GO:0002767 | immune response-inhibiting cell surface receptor signaling pathway | 3 | enriched | 1 | *HLA-G* | 0.0062 | 0.7864 |
| GO:0003205 | cardiac chamber development | 3 | enriched | 1 | *ARID1A* | 0.0065 | 0.7864 |
| GO:0003278 | apoptotic process involved in heart morphogenesis | 3 | enriched | 1 | *CYR61* | 0.0085 | 0.8491 |
| GO:0003408 | optic cup formation involved in camera-type eye development | 3 | enriched | 1 | *ARID1A* | 0.0064 | 0.7864 |
| GO:0006154 | adenosine catabolic process | 3 | enriched | 1 | *ADAL* | 0.0252 | 0.9830 |
| GO:0006189 | de novo' IMP biosynthetic process | 6 | enriched | 1 | *PFAS* | 0.0282 | 0.9830 |
| GO:0006259 | DNA metabolic process | 24 | enriched | 4 | *ERI1, KPNA2, NME1, TOPBP1* | 0.0099 | 0.9124 |
| GO:0006344 | maintenance of chromatin silencing | 4 | enriched | 1 | *ARID1A* | 0.0131 | 0.9707 |
| GO:0006428 | isoleucyl-tRNA aminoacylation | 2 | enriched | 1 | *IARS2* | 0.0054 | 0.7864 |
| GO:0006448 | regulation of translational elongation | 2 | enriched | 1 | *GRM5* | 0.0111 | 0.9124 |
| GO:0006450 | regulation of translational fidelity | 6 | enriched | 1 | *GATC* | 0.0290 | 0.9830 |
| GO:0006457 | protein folding | 139 | enriched | 12 | *CCT6A, CCT8, CDC37, DNAJC25, ERP27, GNAO1, GNAZ, LMAN2L, NUDC, PDCL, SIL1, TBCD* | 0.0086 | 0.8491 |
| GO:0006468 | protein phosphorylation | 476 | enriched | 39 | *MAPK10, PRKCI, TGFBR2, ACVR2B, ADCK3, AURKB, BRD4, CAMK2B, CAMK2D, CCND3, CDC42BPA, CDK15, CLK3, DMPK, FASTKD2, FER, FRK, HCK, HUS1, MAP3K1, MAP3K6, MAP3K8, MAPK1, MARK4, MLKL, PHKG1, PHKG2, PIK3R1, PRKCB, RPS6KA2, SGK1, SIK2, SRPK2, STK31, TAF1L, TEC, TRIO, TSSK1B, TYK2* | 0.0050 | 0.7864 |
| GO:0006476 | protein deacetylation | 11 | enriched | 2 | *HDAC10, HDAC5* | 0.0229 | 0.9830 |
| GO:0006508 | proteolysis | 414 | depleted | 21 | *AGBL1, ADAM12, ADAM23, ADAMTS18, ADAMTSL3, AGA, AQPEP, C2, CAPN12, CASP7, CFB, CTSC, CTSE, DPP6, KLK4, LTA4H, MASP1, SCRN3, TINAG, TPP2, UNC5CL* | 0.0322 | 0.9830 |
| GO:0006563 | L-serine metabolic process | 5 | enriched | 1 | *PSPH* | 0.0162 | 0.9830 |
| GO:0006564 | L-serine biosynthetic process | 7 | enriched | 1 | *PSPH* | 0.0441 | 0.9830 |
| GO:0006607 | NLS-bearing protein import into nucleus | 20 | enriched | 3 | *CBLB, KPNA2, KPNA4* | 0.0103 | 0.9124 |
| GO:0006663 | platelet activating factor biosynthetic process | 4 | enriched | 2 | *PLA2G4A, PLA2G5* | 0.0080 | 0.8319 |
| GO:0006734 | NADH metabolic process | 7 | enriched | 1 | *MDH1B* | 0.0274 | 0.9830 |
| GO:0006744 | ubiquinone biosynthetic process | 15 | enriched | 2 | *ADCK3, COQ5* | 0.0041 | 0.7489 |
| GO:0006778 | porphyrin-containing compound metabolic process | 2 | enriched | 2 | *CYP1A1, CYP1A2* | 0.0017 | 0.6135 |
| GO:0006892 | post-Golgi vesicle-mediated transport | 19 | enriched | 3 | *ARL3, SNAP23, SORCS1* | 0.0171 | 0.9830 |
| GO:0006928 | movement of cell or subcellular component | 4 | enriched | 1 | *SPHKAP* | 0.0468 | 0.9830 |
| GO:0007020 | microtubule nucleation | 17 | enriched | 3 | *AKAP9, TUBGCP4, TUBGCP6* | 0.0034 | 0.7489 |
| GO:0007041 | lysosomal transport | 18 | enriched | 4 | *ARSB, NEDD4, PCSK9, USE1* | 0.0032 | 0.7489 |
| GO:0007076 | mitotic chromosome condensation | 17 | enriched | 3 | *AKAP8, AKAP8L, SMC4* | 0.0009 | 0.4811 |
| GO:0007091 | metaphase/anaphase transition of mitotic cell cycle | 6 | enriched | 1 | *CDC27* | 0.0376 | 0.9830 |
| GO:0007155 | cell adhesion | 447 | enriched | 62 | *BCAM, PLXNC1, ADAM12, ADAM23, ARVCF, CCR3, CHL1, COL28A1, COL8A1, CTNNA1, CTNND2, CYR61, ENG, EPHA3, FAM65B, FER, HCK, IBSP, ICAM3, IGFBP7, ITGAL, ITGB8, MLLT4, NEDD9, NME1-NME2, NME2, NOV, NTM, PARD3, PCDH7, PCDHGA1, PCDHGA10, PCDHGA11, PCDHGA12, PCDHGA2, PCDHGA3, PCDHGA4, PCDHGA5, PCDHGA6, PCDHGA7, PCDHGA8, PCDHGA9, PCDHGB1, PCDHGB2, PCDHGB3, PCDHGB4, PCDHGB6, PCDHGB7, PCDHGC3, PCDHGC4, PCDHGC5, PXN, ROBO1, SELP, SSPN, STAB2, SVEP1, SYMPK, THBS1, TINAG, VCAM1, WISP2* | 0.0010 | 0.5046 |
| GO:0007156 | homophilic cell adhesion via plasma membrane adhesion molecules | 162 | enriched | 31 | *ROBO2, CDH13, DCHS2, CDHR2, CNTN4, IGSF21, PCDH7, PCDHGA1, PCDHGA10, PCDHGA11, PCDHGA12, PCDHGA2, PCDHGA3, PCDHGA4, PCDHGA5, PCDHGA6, PCDHGA7, PCDHGA8, PCDHGA9, PCDHGB1, PCDHGB2, PCDHGB3, PCDHGB4, PCDHGB6, PCDHGB7, PCDHGC3, PCDHGC4, PCDHGC5, PLXNB2, PVRL2, ROBO1* | 0.0001 | 0.1541 |
| GO:0007175 | negative regulation of epidermal growth factor-activated receptor activity | 14 | enriched | 4 | *CBLB, CBLC, SOCS5, ZFYVE28* | 0.0084 | 0.8491 |
| GO:0007178 | transmembrane receptor protein serine/threonine kinase signaling pathway | 10 | enriched | 2 | *ACVR2B, LTBP1* | 0.0293 | 0.9830 |
| GO:0007206 | phospholipase C-activating G protein-coupled glutamate receptor signaling pathway | 2 | enriched | 1 | *GRM5* | 0.0109 | 0.9124 |
| GO:0007275 | multicellular organism development | 513 | depleted | 23 | *CDH13, CENPE, ARHGDIB, ARVCF, C6orf58, CCHCR1, CUX1, DDX25, DOPEY2, EDARADD, EPHA3, FLT1, GNRHR, HHAT, KIT, MET, MORC1, PDGFD, PRR15, TSHZ2, TSSK1B, ZGLP1, ZNF267* | 0.0323 | 0.9830 |
| GO:0007399 | nervous system development | 294 | depleted | 17 | *CHRM3, RBFOX1, BPNT1, CNTN4, CSGALNACT1, DLX5, DLX6, FGF14, GAP43, GRIK1, LPPR1, MARK4, MBD5, NLGN1, RAPGEF5, ROBO1, SEMA6A* | 0.0456 | 0.9830 |
| GO:0007406 | negative regulation of neuroblast proliferation | 5 | enriched | 1 | *CTNNA1* | 0.0483 | 0.9830 |
| GO:0007568 | aging | 170 | enriched | 18 | *CYP1A1, TGFBR2, APOD, AURKB, C1QA, CTNNA1, CTSC, GNAO1, KMO, KRTAP4-5, KRTAP4-7, KRTAP4-8, KRTAP4-9, MAPK1, NPY2R, PTGS2, PTH1R, VCAM1* | 0.0041 | 0.7489 |
| GO:0008356 | asymmetric cell division | 5 | enriched | 2 | *ACTR3, PARD3* | 0.0166 | 0.9830 |
| GO:0008594 | photoreceptor cell morphogenesis | 3 | enriched | 1 | *C8orf37* | 0.0048 | 0.7864 |
| GO:0008630 | intrinsic apoptotic signaling pathway in response to DNA damage | 45 | enriched | 3 | *NFATC4, PIK3R1, SFN* | 0.0452 | 0.9830 |
| GO:0009143 | nucleoside triphosphate catabolic process | 6 | enriched | 1 | *DCTPP1* | 0.0297 | 0.9830 |
| GO:0009308 | amine metabolic process | 8 | enriched | 2 | *CYP1A1, VCAM1* | 0.0166 | 0.9830 |
| GO:0009624 | response to nematode | 3 | enriched | 1 | *CYP1A1* | 0.0121 | 0.9275 |
| GO:0009812 | flavonoid metabolic process | 5 | enriched | 1 | *CYP1A1* | 0.0293 | 0.9830 |
| GO:0010032 | meiotic chromosome condensation | 6 | enriched | 1 | *SMC4* | 0.0309 | 0.9830 |
| GO:0010038 | response to metal ion | 13 | enriched | 3 | *GPHN, NEDD4L, SNCB* | 0.0410 | 0.9830 |
| GO:0010041 | response to iron(III) ion | 3 | enriched | 1 | *CYP1A1* | 0.0098 | 0.9124 |
| GO:0010389 | regulation of G2/M transition of mitotic cell cycle | 80 | enriched | 9 | *PPP2R1A, AKAP9, CEP192, CNTRL, CTC1, DYNLL1, FGFR1OP, HAUS2, KCNH5* | <0.0001 | 0.0322 |
| GO:0010457 | centriole-centriole cohesion | 11 | enriched | 3 | *CNTLN, CROCC, RTTN* | 0.0016 | 0.6081 |
| GO:0010482 | regulation of epidermal cell division | 2 | enriched | 1 | *SFN* | 0.0273 | 0.9830 |
| GO:0010518 | positive regulation of phospholipase activity | 8 | enriched | 2 | *CYR61, PLA2G5* | 0.0011 | 0.5319 |
| GO:0010569 | regulation of double-strand break repair via homologous recombination | 18 | enriched | 3 | *SETD2, TEX15, ZNF365* | 0.0149 | 0.9830 |
| GO:0010596 | negative regulation of endothelial cell migration | 22 | enriched | 3 | *KRIT1, SP100, THBS1* | 0.0094 | 0.9024 |
| GO:0010626 | negative regulation of Schwann cell proliferation | 5 | enriched | 1 | *RNF10* | 0.0189 | 0.9830 |
| GO:0010748 | negative regulation of long-chain fatty acid import across plasma membrane | 4 | enriched | 1 | *THBS1* | 0.0102 | 0.9124 |
| GO:0010751 | negative regulation of nitric oxide mediated signal transduction | 3 | enriched | 1 | *THBS1* | 0.0061 | 0.7864 |
| GO:0010754 | negative regulation of cGMP-mediated signaling | 6 | enriched | 3 | *PDE11A, PDE2A, THBS1* | 0.0051 | 0.7864 |
| GO:0010757 | negative regulation of plasminogen activation | 6 | enriched | 1 | *THBS1* | 0.0274 | 0.9830 |
| GO:0010760 | negative regulation of macrophage chemotaxis | 7 | enriched | 1 | *C5* | 0.0432 | 0.9830 |
| GO:0010768 | negative regulation of transcription from RNA polymerase II promoter in response to UV-induced DNA damage | 2 | enriched | 1 | *NEDD4* | 0.0154 | 0.9830 |
| GO:0010793 | regulation of mRNA export from nucleus | 6 | enriched | 2 | *AKAP8L, SETD2* | 0.0022 | 0.6905 |
| GO:0010830 | regulation of myotube differentiation | 5 | enriched | 2 | *DMPK, HDAC5* | 0.0003 | 0.3133 |
| GO:0010923 | negative regulation of phosphatase activity | 49 | enriched | 8 | *FARP1, GPATCH2, CCDC8, CD2BP2, CEP192, DLG2, PPP1R37, TMEM132D* | 0.0007 | 0.4811 |
| GO:0010941 | regulation of cell death | 7 | enriched | 1 | *NOA1* | 0.0425 | 0.9830 |
| GO:0010955 | negative regulation of protein processing | 15 | enriched | 2 | *GLG1, TMEM98* | 0.0342 | 0.9830 |
| GO:0015711 | organic anion transport | 14 | enriched | 4 | *SLC22A10, SLC22A24, SLC22A25, SLC22A9* | 0.0060 | 0.7864 |
| GO:0015866 | ADP transport | 6 | enriched | 1 | *SLC25A42* | 0.0293 | 0.9830 |
| GO:0016259 | selenocysteine metabolic process | 3 | enriched | 1 | *SEPHS2* | 0.0062 | 0.7864 |
| GO:0016260 | selenocysteine biosynthetic process | 2 | enriched | 1 | *SEPHS2* | 0.0042 | 0.7489 |
| GO:0016344 | meiotic chromosome movement towards spindle pole | 2 | enriched | 1 | *ACTR3* | 0.0061 | 0.7864 |
| GO:0016556 | mRNA modification | 3 | enriched | 1 | *APOBEC2* | 0.0113 | 0.9124 |
| GO:0016926 | protein desumoylation | 7 | enriched | 1 | *SENP6* | 0.0489 | 0.9830 |
| GO:0017143 | insecticide metabolic process | 2 | enriched | 1 | *CYP1A1* | 0.0070 | 0.7892 |
| GO:0017144 | drug metabolic process | 25 | enriched | 3 | *CYP1A1, ADAL, CYP1A2* | 0.0289 | 0.9830 |
| GO:0018106 | peptidyl-histidine phosphorylation | 2 | enriched | 2 | *NME1-NME2, NME2* | 0.0260 | 0.9830 |
| GO:0019054 | modulation by virus of host cellular process | 7 | enriched | 2 | *KPNA2, KPNA4* | 0.0015 | 0.6081 |
| GO:0019075 | virus maturation | 2 | enriched | 1 | *MVB12B* | 0.0301 | 0.9830 |
| GO:0019370 | leukotriene biosynthetic process | 10 | enriched | 3 | *ALOX5, LTA4H, PLA2G5* | 0.0037 | 0.7489 |
| GO:0030001 | metal ion transport | 5 | enriched | 1 | *NDFIP2* | 0.0187 | 0.9830 |
| GO:0030003 | cellular cation homeostasis | 2 | enriched | 1 | *ATP13A2* | 0.0030 | 0.7489 |
| GO:0030048 | actin filament-based movement | 19 | enriched | 3 | *MYO6, MYO9B, WASF2* | 0.0079 | 0.8319 |
| GO:0030330 | DNA damage response, signal transduction by p53 class mediator | 16 | enriched | 2 | *MYO6, TRIAP1* | 0.0266 | 0.9830 |
| GO:0031016 | pancreas development | 24 | enriched | 4 | *ACVR2B, ALDH1A2, HNF1A, MET* | 0.0181 | 0.9830 |
| GO:0031048 | chromatin silencing by small RNA | 3 | enriched | 1 | *FAM172A* | 0.0377 | 0.9830 |
| GO:0031052 | chromosome breakage | 2 | enriched | 1 | *RFWD3* | 0.0040 | 0.7489 |
| GO:0031065 | positive regulation of histone deacetylation | 11 | enriched | 2 | *AKAP8, AKAP8L* | 0.0117 | 0.9194 |
| GO:0031087 | deadenylation-independent decapping of nuclear-transcribed mRNA | 5 | enriched | 1 | *EDC3* | 0.0234 | 0.9830 |
| GO:0031344 | regulation of cell projection organization | 3 | enriched | 1 | *PIFO* | 0.0450 | 0.9830 |
| GO:0031424 | keratinization | 181 | enriched | 21 | *KRT39, KRT40, KRT82, KRTAP1-1, KRTAP1-3, KRTAP1-4, KRTAP1-5, KRTAP2-1, KRTAP2-2, KRTAP2-3, KRTAP2-4, KRTAP3-1, KRTAP3-2, KRTAP3-3, KRTAP4-11, KRTAP4-5, KRTAP4-6, KRTAP4-7, KRTAP4-8, KRTAP4-9, SFN* | <0.0001 | <0.0001 |
| GO:0031547 | brain-derived neurotrophic factor receptor signaling pathway | 5 | enriched | 1 | *NFATC4* | 0.0311 | 0.9830 |
| GO:0031571 | mitotic G1 DNA damage checkpoint | 8 | enriched | 2 | *FBXO31, RFWD3* | 0.0300 | 0.9830 |
| GO:0031642 | negative regulation of myelination | 8 | enriched | 2 | *CTSC, TMEM98* | 0.0468 | 0.9830 |
| GO:0032008 | positive regulation of TOR signaling | 29 | enriched | 3 | *MIOS, RPTOR, SEH1L* | 0.0359 | 0.9830 |
| GO:0032053 | ciliary basal body organization | 3 | enriched | 2 | *CROCC, RTTN* | 0.0017 | 0.6135 |
| GO:0032094 | response to food | 20 | enriched | 2 | *CYP1A1, SLC16A1* | 0.0249 | 0.9830 |
| GO:0032211 | negative regulation of telomere maintenance via telomerase | 20 | enriched | 2 | *CTC1, PINX1* | 0.0220 | 0.9830 |
| GO:0032212 | positive regulation of telomere maintenance via telomerase | 34 | enriched | 5 | *AURKB, CCT6A, CCT8, MAPK1, NAF1* | 0.0045 | 0.7660 |
| GO:0032259 | methylation | 54 | enriched | 5 | *COMT, COQ5, CYP1A2, NDUFAF5, SETD9* | 0.0348 | 0.9830 |
| GO:0032410 | negative regulation of transporter activity | 5 | enriched | 1 | *NDFIP2* | 0.0245 | 0.9830 |
| GO:0032425 | positive regulation of mismatch repair | 3 | enriched | 1 | *HDAC10* | 0.0248 | 0.9830 |
| GO:0032438 | melanosome organization | 22 | enriched | 3 | *DTNBP1, KIF13A, RAB38* | 0.0086 | 0.8491 |
| GO:0032543 | mitochondrial translation | 31 | enriched | 4 | *GATC, IARS2, MRPS17, NOA1* | 0.0001 | 0.1588 |
| GO:0032648 | regulation of interferon-beta production | 4 | enriched | 1 | *RNF216* | 0.0186 | 0.9830 |
| GO:0032914 | positive regulation of transforming growth factor beta1 production | 6 | enriched | 1 | *THBS1* | 0.0229 | 0.9830 |
| GO:0032927 | positive regulation of activin receptor signaling pathway | 7 | enriched | 1 | *ACVR2B* | 0.0446 | 0.9830 |
| GO:0033127 | regulation of histone phosphorylation | 2 | enriched | 2 | *AKAP8, AKAP8L* | 0.0015 | 0.6081 |
| GO:0033157 | regulation of intracellular protein transport | 7 | enriched | 1 | *ATP13A2* | 0.0492 | 0.9830 |
| GO:0033206 | meiotic cytokinesis | 2 | enriched | 1 | *ACTR3* | 0.0061 | 0.7864 |
| GO:0033574 | response to testosterone | 31 | enriched | 4 | *FOXP2, NME1, PSPH, THBS1* | 0.0353 | 0.9830 |
| GO:0033690 | positive regulation of osteoblast proliferation | 10 | enriched | 2 | *CTHRC1, CYR61* | 0.0009 | 0.4811 |
| GO:0034198 | cellular response to amino acid starvation | 40 | enriched | 5 | *C7orf60, GCN1L1, MAPK1, MIOS, SEH1L* | 0.0022 | 0.6905 |
| GO:0034340 | response to type I interferon | 7 | enriched | 2 | *SETD2, SP100* | 0.0080 | 0.8321 |
| GO:0034629 | cellular protein-containing complex localization | 9 | enriched | 3 | *SGCD, MIOS, SEH1L* | 0.0009 | 0.4811 |
| GO:0034644 | cellular response to UV | 49 | enriched | 7 | *AURKB, NEDD4, NFATC4, PIK3R1, PTGS2, TMEM161A, TRIAP1* | 0.0001 | 0.1626 |
| GO:0034728 | nucleosome organization | 3 | enriched | 1 | *SETD2* | 0.0068 | 0.7864 |
| GO:0035063 | nuclear speck organization | 4 | enriched | 1 | *SRPK2* | 0.0140 | 0.9830 |
| GO:0035333 | Notch receptor processing, ligand-dependent | 7 | enriched | 1 | *PSEN2* | 0.0430 | 0.9830 |
| GO:0035349 | coenzyme A transmembrane transport | 2 | enriched | 1 | *SLC25A42* | 0.0034 | 0.7489 |
| GO:0035350 | FAD transmembrane transport | 2 | enriched | 1 | *SLC25A32* | 0.0040 | 0.7489 |
| GO:0035377 | transepithelial water transport | 2 | enriched | 1 | *CFTR* | 0.0109 | 0.9124 |
| GO:0035441 | cell migration involved in vasculogenesis | 2 | enriched | 1 | *SETD2* | 0.0040 | 0.7489 |
| GO:0035556 | intracellular signal transduction | 364 | enriched | 25 | *MAPK10, PRKCI, CAB39, CDC42BPA, CHN2, DEPDC1, DMPK, FER, GPR155, MAPK1, MAPK11, MAPK12, MARK4, NRG3, PDZD8, PRKCB, PSEN2, RPS6KA2, SGK1, SIK2, SOCS5, SRPK2, TEC, TSSK1B, TYK2* | 0.0473 | 0.9830 |
| GO:0035565 | regulation of pronephros size | 2 | enriched | 1 | *HNF1A* | 0.0198 | 0.9830 |
| GO:0035623 | renal glucose absorption | 1 | enriched | 1 | *HNF1A* | 0.0251 | 0.9830 |
| GO:0035655 | interleukin-18-mediated signaling pathway | 7 | enriched | 1 | *ALOX5* | 0.0481 | 0.9830 |
| GO:0035825 | homologous recombination | 1 | enriched | 1 | *HDAC10* | 0.0296 | 0.9830 |
| GO:0035879 | plasma membrane lactate transport | 3 | enriched | 1 | *SLC16A1* | 0.0063 | 0.7864 |
| GO:0036037 | CD8-positive, alpha-beta T cell activation | 3 | enriched | 1 | *HLA-A* | 0.0071 | 0.7892 |
| GO:0038018 | Wnt receptor catabolic process | 2 | enriched | 1 | *ZNRF3* | 0.0115 | 0.9160 |
| GO:0040020 | regulation of meiotic nuclear division | 5 | enriched | 2 | *GPR3, PDE3A* | 0.0144 | 0.9830 |
| GO:0040029 | regulation of gene expression, epigenetic | 14 | enriched | 2 | *HDAC5, MORC1* | 0.0207 | 0.9830 |
| GO:0042262 | DNA protection | 5 | enriched | 1 | *DCTPP1* | 0.0059 | 0.7864 |
| GO:0042270 | protection from natural killer cell mediated cytotoxicity | 5 | enriched | 1 | *HLA-G* | 0.0217 | 0.9830 |
| GO:0042376 | phylloquinone catabolic process | 3 | enriched | 1 | *CBR3* | 0.0036 | 0.7489 |
| GO:0042461 | photoreceptor cell development | 4 | enriched | 1 | *ARL3* | 0.0114 | 0.9124 |
| GO:0042590 | antigen processing and presentation of exogenous peptide antigen via MHC class I | 4 | enriched | 1 | *HLA-A* | 0.0129 | 0.9668 |
| GO:0042633 | hair cycle | 15 | enriched | 5 | *KRTAP4-5, KRTAP4-7, KRTAP4-8, KRTAP4-9, PTGS2* | <0.0001 | 0.0060 |
| GO:0042759 | long-chain fatty acid biosynthetic process | 22 | enriched | 5 | *ALOX5, CYP1A1, CYP1A2, LTA4H, PTGS2* | 0.0020 | 0.6812 |
| GO:0042766 | nucleosome mobilization | 4 | enriched | 2 | *ARID1A, BPTF* | 0.0001 | 0.1541 |
| GO:0042904 | 9-cis-retinoic acid biosynthetic process | 4 | enriched | 2 | *CYP1A1, ALDH1A2* | 0.0173 | 0.9830 |
| GO:0042921 | glucocorticoid receptor signaling pathway | 5 | enriched | 2 | *ARID1A, NEDD4* | <0.0001 | 0.1523 |
| GO:0043065 | positive regulation of apoptotic process | 329 | depleted | 9 | *ALDH1A2, FOXO1, PLEKHG5, PSEN2, PTGS2, REST, RPS6KA2, TNFAIP8, TRIO* | 0.0309 | 0.9830 |
| GO:0043154 | negative regulation of cysteine-type endopeptidase activity involved in apoptotic process | 71 | enriched | 6 | *BIRC7, PTGS2, SFN, THBS1, TNFAIP8, TRIAP1* | 0.0261 | 0.9830 |
| GO:0043279 | response to alkaloid | 6 | enriched | 1 | *SRSF9* | 0.0294 | 0.9830 |
| GO:0043489 | RNA stabilization | 2 | enriched | 1 | *NAF1* | 0.0121 | 0.9275 |
| GO:0043506 | regulation of JUN kinase activity | 2 | enriched | 1 | *DTNBP1* | 0.0071 | 0.7892 |
| GO:0043551 | regulation of phosphatidylinositol 3-kinase activity | 17 | enriched | 2 | *PIK3R1, SOCS5* | 0.0202 | 0.9830 |
| GO:0043666 | regulation of phosphoprotein phosphatase activity | 41 | enriched | 7 | *PPP2R1A, DMPK, PPP1R3B, PPP2R1B, PPP2R2B, PPP2R5A, PPP6R2* | 0.0015 | 0.6081 |
| GO:0043932 | ossification involved in bone remodeling | 5 | enriched | 1 | *CTHRC1* | 0.0190 | 0.9830 |
| GO:0044829 | positive regulation by host of viral genome replication | 6 | enriched | 1 | *YTHDC2* | 0.0296 | 0.9830 |
| GO:0044839 | cell cycle G2/M phase transition | 2 | enriched | 2 | *AKAP8, AKAP8L* | 0.0015 | 0.6081 |
| GO:0045132 | meiotic chromosome segregation | 5 | enriched | 1 | *SMC4* | 0.0220 | 0.9830 |
| GO:0045191 | regulation of isotype switching | 4 | enriched | 1 | *APLF* | 0.0460 | 0.9830 |
| GO:0045333 | cellular respiration | 17 | enriched | 4 | *CYP1A2, NDUFS1, NDUFS4, NFATC4* | <0.0001 | 0.1186 |
| GO:0045606 | positive regulation of epidermal cell differentiation | 7 | enriched | 1 | *SFN* | 0.0427 | 0.9830 |
| GO:0045629 | negative regulation of T-helper 2 cell differentiation | 4 | enriched | 1 | *SOCS5* | 0.0328 | 0.9830 |
| GO:0045921 | positive regulation of exocytosis | 23 | enriched | 3 | *CFTR, RAB5A, VSNL1* | 0.0413 | 0.9830 |
| GO:0046103 | inosine biosynthetic process | 3 | enriched | 1 | *ADAL* | 0.0252 | 0.9830 |
| GO:0046626 | regulation of insulin receptor signaling pathway | 12 | enriched | 3 | *CCND3, PIK3R1, SIK2* | 0.0024 | 0.6957 |
| GO:0048050 | post-embryonic eye morphogenesis | 3 | enriched | 1 | *MFAP2* | 0.0058 | 0.7864 |
| GO:0048254 | snoRNA localization | 6 | enriched | 1 | *ZNHIT6* | 0.0266 | 0.9830 |
| GO:0048332 | mesoderm morphogenesis | 2 | enriched | 1 | *SETD2* | 0.0040 | 0.7489 |
| GO:0048599 | oocyte development | 12 | enriched | 2 | *YTHDC2, ZGLP1* | 0.0325 | 0.9830 |
| GO:0048814 | regulation of dendrite morphogenesis | 21 | enriched | 4 | *KNDC1, NEDD4, NEDD4L, RAP2A* | 0.0153 | 0.9830 |
| GO:0048864 | stem cell development | 5 | enriched | 1 | *SETD2* | 0.0173 | 0.9830 |
| GO:0050665 | hydrogen peroxide biosynthetic process | 8 | enriched | 2 | *CYP1A1, CYP1A2* | 0.0095 | 0.9024 |
| GO:0050774 | negative regulation of dendrite morphogenesis | 9 | enriched | 2 | *GORASP1, NFATC4* | 0.0009 | 0.4811 |
| GO:0050808 | synapse organization | 46 | enriched | 10 | *GRM5, PPFIA2, C1QA, CTNND2, LRRTM2, PCDHGC3, PCDHGC4, PCDHGC5, PPFIBP2, SNCB* | <0.0001 | 0.0023 |
| GO:0050890 | cognition | 45 | enriched | 7 | *GRM5, CBR3, CHL1, DOPEY2, GPR155, HLA-DRA, TUSC3* | 0.0006 | 0.4291 |
| GO:0051036 | regulation of endosome size | 2 | enriched | 1 | *RAB5A* | 0.0155 | 0.9830 |
| GO:0051046 | regulation of secretion | 2 | enriched | 1 | *MYO6* | 0.0072 | 0.7962 |
| GO:0051106 | positive regulation of DNA ligation | 4 | enriched | 1 | *APLF* | 0.0455 | 0.9830 |
| GO:0051149 | positive regulation of muscle cell differentiation | 25 | enriched | 3 | *CTNNA1, MAPK11, MAPK12* | 0.0055 | 0.7864 |
| GO:0051224 | negative regulation of protein transport | 6 | enriched | 1 | *NDFIP2* | 0.0315 | 0.9830 |
| GO:0051225 | spindle assembly | 53 | enriched | 4 | *HAUS2, STARD9, TUBGCP4, TUBGCP6* | 0.0357 | 0.9830 |
| GO:0051232 | meiotic spindle elongation | 1 | enriched | 1 | *PPP2R1A* | 0.0288 | 0.9830 |
| GO:0051271 | negative regulation of cellular component movement | 7 | enriched | 2 | *ACTN4, SP100* | 0.0402 | 0.9830 |
| GO:0051315 | attachment of mitotic spindle microtubules to kinetochore | 10 | enriched | 2 | *CENPE, SEH1L* | 0.0248 | 0.9830 |
| GO:0051383 | kinetochore organization | 6 | enriched | 1 | *SMC4* | 0.0301 | 0.9830 |
| GO:0051415 | microtubule nucleation by interphase microtubule organizing center | 6 | enriched | 2 | *TUBGCP4, TUBGCP6* | 0.0044 | 0.7633 |
| GO:0051454 | intracellular pH elevation | 4 | enriched | 1 | *CFTR* | 0.0380 | 0.9830 |
| GO:0051653 | spindle localization | 5 | enriched | 1 | *ACTR3* | 0.0308 | 0.9830 |
| GO:0051661 | maintenance of centrosome location | 6 | enriched | 1 | *AKAP9* | 0.0316 | 0.9830 |
| GO:0051729 | germline cell cycle switching, mitotic to meiotic cell cycle | 2 | enriched | 1 | *YTHDC2* | 0.0040 | 0.7489 |
| GO:0051823 | regulation of synapse structural plasticity | 5 | enriched | 2 | *CAMK2B, DMPK* | 0.0118 | 0.9251 |
| GO:0051895 | negative regulation of focal adhesion assembly | 18 | enriched | 4 | *APOD, DLC1, RCC2, THBS1* | 0.0102 | 0.9124 |
| GO:0051972 | regulation of telomerase activity | 6 | enriched | 1 | *PINX1* | 0.0389 | 0.9830 |
| GO:0051987 | positive regulation of attachment of spindle microtubules to kinetochore | 3 | enriched | 1 | *RCC2* | 0.0057 | 0.7864 |
| GO:0052746 | inositol phosphorylation | 2 | enriched | 1 | *IPPK* | 0.0040 | 0.7489 |
| GO:0055001 | muscle cell development | 4 | enriched | 1 | *NFATC4* | 0.0169 | 0.9830 |
| GO:0055065 | metal ion homeostasis | 3 | enriched | 1 | *CNNM4* | 0.0463 | 0.9830 |
| GO:0055069 | zinc ion homeostasis | 5 | enriched | 2 | *ATP13A2, PARK2* | 0.0132 | 0.9726 |
| GO:0060044 | negative regulation of cardiac muscle cell proliferation | 13 | enriched | 3 | *TGFBR2, KCNK2, MAPK11* | 0.0138 | 0.9830 |
| GO:0060155 | platelet dense granule organization | 8 | enriched | 2 | *DTNBP1, RAB38* | 0.0066 | 0.7864 |
| GO:0060159 | regulation of dopamine receptor signaling pathway | 4 | enriched | 1 | *DTNBP1* | 0.0181 | 0.9830 |
| GO:0060294 | cilium movement involved in cell motility | 10 | enriched | 2 | *RSPH6A, TEKT5* | 0.0417 | 0.9830 |
| GO:0060333 | interferon-gamma-mediated signaling pathway | 69 | enriched | 9 | *CAMK2B, CAMK2D, HCK, HLA-A, HLA-DRA, HLA-G, SP100, TRIM8, VCAM1* | 0.0197 | 0.9830 |
| GO:0060491 | regulation of cell projection assembly | 3 | enriched | 1 | *FAM110C* | 0.0063 | 0.7864 |
| GO:0060591 | chondroblast differentiation | 5 | enriched | 1 | *CYR61* | 0.0188 | 0.9830 |
| GO:0060613 | fat pad development | 7 | enriched | 1 | *ARRDC3* | 0.0463 | 0.9830 |
| GO:0060669 | embryonic placenta morphogenesis | 6 | enriched | 1 | *SETD2* | 0.0297 | 0.9830 |
| GO:0060829 | negative regulation of canonical Wnt signaling pathway involved in neural plate anterior/posterior pattern formation | 1 | enriched | 1 | *TBX18* | 0.0326 | 0.9830 |
| GO:0060836 | lymphatic endothelial cell differentiation | 6 | enriched | 1 | *ACVR2B* | 0.0296 | 0.9830 |
| GO:0060840 | artery development | 7 | enriched | 1 | *ACVR2B* | 0.0437 | 0.9830 |
| GO:0060841 | venous blood vessel development | 4 | enriched | 1 | *ACVR2B* | 0.0111 | 0.9124 |
| GO:0060977 | coronary vasculature morphogenesis | 3 | enriched | 2 | *SGCD, SETD2* | 0.0023 | 0.6945 |
| GO:0061084 | negative regulation of protein refolding | 4 | enriched | 1 | *PDCL* | 0.0188 | 0.9830 |
| GO:0061088 | regulation of sequestering of zinc ion | 5 | enriched | 1 | *SLC30A2* | 0.0202 | 0.9830 |
| GO:0061298 | retina vasculature development in camera-type eye | 7 | enriched | 1 | *ACVR2B* | 0.0437 | 0.9830 |
| GO:0061470 | T follicular helper cell differentiation | 4 | enriched | 1 | *RC3H2* | 0.0100 | 0.9124 |
| GO:0061951 | establishment of protein localization to plasma membrane | 4 | enriched | 2 | *GORASP1, MLLT4* | 0.0003 | 0.3133 |
| GO:0070681 | glutaminyl-tRNAGln biosynthesis via transamidation | 3 | enriched | 1 | *GATC* | 0.0074 | 0.8082 |
| GO:0070940 | dephosphorylation of RNA polymerase II C-terminal domain | 5 | enriched | 1 | *RPRD1A* | 0.0252 | 0.9830 |
| GO:0070988 | demethylation | 4 | enriched | 2 | *CYP1A1, CYP51A1* | <0.0001 | 0.1393 |
| GO:0071344 | diphosphate metabolic process | 2 | enriched | 1 | *PPA1* | 0.0040 | 0.7489 |
| GO:0071539 | protein localization to centrosome | 19 | enriched | 2 | *CCDC41, CEP192* | 0.0437 | 0.9830 |
| GO:0071901 | negative regulation of protein serine/threonine kinase activity | 17 | enriched | 3 | *DTNBP1, RPTOR, SFN* | 0.0022 | 0.6905 |
| GO:0072344 | rescue of stalled ribosome | 5 | enriched | 1 | *LTN1* | 0.0227 | 0.9830 |
| GO:0072385 | minus-end-directed organelle transport along microtubule | 4 | enriched | 1 | *BICD2* | 0.0126 | 0.9540 |
| GO:0072393 | microtubule anchoring at microtubule organizing center | 2 | enriched | 1 | *BICD2* | 0.0041 | 0.7489 |
| GO:0072593 | reactive oxygen species metabolic process | 31 | enriched | 3 | *CYR61, NDUFS1, NDUFS4* | 0.0109 | 0.9124 |
| GO:0072673 | lamellipodium morphogenesis | 5 | enriched | 1 | *WASF2* | 0.0219 | 0.9830 |
| GO:0075506 | entry of viral genome into host nucleus through nuclear pore complex via importin | 2 | enriched | 1 | *KPNA2* | 0.0254 | 0.9830 |
| GO:0080121 | AMP transport | 2 | enriched | 1 | *SLC25A42* | 0.0034 | 0.7489 |
| GO:0090051 | negative regulation of cell migration involved in sprouting angiogenesis | 15 | enriched | 2 | *HDAC5, THBS1* | 0.0272 | 0.9830 |
| GO:0090169 | regulation of spindle assembly | 2 | enriched | 1 | *SENP6* | 0.0051 | 0.7864 |
| GO:0090177 | establishment of planar polarity involved in neural tube closure | 2 | enriched | 1 | *CTHRC1* | 0.0032 | 0.7489 |
| GO:0090197 | positive regulation of chemokine secretion | 12 | enriched | 2 | *C5, CHIA* | 0.0462 | 0.9830 |
| GO:0090222 | centrosome-templated microtubule nucleation | 2 | enriched | 1 | *CEP192* | 0.0042 | 0.7489 |
| GO:0090234 | regulation of kinetochore assembly | 3 | enriched | 1 | *SENP6* | 0.0075 | 0.8131 |
| GO:0090327 | negative regulation of locomotion involved in locomotory behavior | 2 | enriched | 1 | *ARRDC3* | 0.0048 | 0.7864 |
| GO:0090669 | telomerase RNA stabilization | 4 | enriched | 1 | *NAF1* | 0.0237 | 0.9830 |
| GO:0097051 | establishment of protein localization to endoplasmic reticulum membrane | 3 | enriched | 1 | *RAB3GAP2* | 0.0085 | 0.8491 |
| GO:0097065 | anterior head development | 5 | enriched | 1 | *PFAS* | 0.0188 | 0.9830 |
| GO:0097176 | epoxide metabolic process | 3 | enriched | 1 | *EPHX3* | 0.0245 | 0.9830 |
| GO:0097198 | histone H3-K36 trimethylation | 2 | enriched | 1 | *SETD2* | 0.0040 | 0.7489 |
| GO:0097267 | omega-hydroxylase P450 pathway | 10 | enriched | 2 | *CYP1A1, CYP1A2* | 0.0196 | 0.9830 |
| GO:0097676 | histone H3-K36 dimethylation | 4 | enriched | 1 | *SETD2* | 0.0106 | 0.9124 |
| GO:0097699 | vascular endothelial cell response to fluid shear stress | 1 | enriched | 1 | *SOCS5* | 0.0397 | 0.9830 |
| GO:0097711 | ciliary basal body-plasma membrane docking | 95 | enriched | 9 | *PPP2R1A, AKAP9, CCDC41, CEP192, CNTRL, DYNLL1, FGFR1OP, HAUS2, MARK4* | 0.0002 | 0.2357 |
| GO:0097752 | regulation of DNA stability | 4 | enriched | 1 | *DDI1* | 0.0364 | 0.9830 |
| GO:0098779 | positive regulation of mitophagy in response to mitochondrial depolarization | 4 | enriched | 2 | *CDC37, PARK2* | 0.0149 | 0.9830 |
| GO:0098883 | synapse pruning | 8 | enriched | 3 | *C1QA, C1QB, C1QC* | 0.0368 | 0.9830 |
| GO:0098909 | regulation of cardiac muscle cell action potential involved in regulation of contraction | 8 | enriched | 3 | *AKAP9, CAMK2D, RANGRF* | 0.0195 | 0.9830 |
| GO:0099151 | regulation of postsynaptic density assembly | 12 | enriched | 4 | *NRXN1, GRID2, LRRTM2, PTPRD* | 0.0125 | 0.9496 |
| GO:0099553 | trans-synaptic signaling by endocannabinoid, modulating synaptic transmission | 3 | enriched | 1 | *GRM5* | 0.0214 | 0.9830 |
| GO:0099606 | microtubule plus-end directed mitotic chromosome migration | 1 | enriched | 1 | *CENPE* | 0.0317 | 0.9830 |
| GO:0099607 | lateral attachment of mitotic spindle microtubules to kinetochore | 1 | enriched | 1 | *CENPE* | 0.0317 | 0.9830 |
| GO:0106048 | spermidine deacetylation | 2 | enriched | 1 | *HDAC10* | 0.0141 | 0.9830 |
| GO:1900025 | negative regulation of substrate adhesion-dependent cell spreading | 13 | enriched | 3 | *ACTN4, KANK1, RCC2* | 0.0129 | 0.9668 |
| GO:1900037 | regulation of cellular response to hypoxia | 4 | enriched | 1 | *CHCHD2* | 0.0111 | 0.9124 |
| GO:1900087 | positive regulation of G1/S transition of mitotic cell cycle | 32 | enriched | 4 | *CYP1A1, CCND3, KMT2E, RPTOR* | 0.0304 | 0.9830 |
| GO:1900103 | positive regulation of endoplasmic reticulum unfolded protein response | 5 | enriched | 1 | *PIK3R1* | 0.0189 | 0.9830 |
| GO:1900180 | regulation of protein localization to nucleus | 6 | enriched | 1 | *ATP13A2* | 0.0296 | 0.9830 |
| GO:1900186 | negative regulation of clathrin-dependent endocytosis | 4 | enriched | 1 | *SH3GL3* | 0.0443 | 0.9830 |
| GO:1901016 | regulation of potassium ion transmembrane transporter activity | 6 | enriched | 2 | *NEDD4, NEDD4L* | 0.0420 | 0.9830 |
| GO:1901018 | positive regulation of potassium ion transmembrane transporter activity | 6 | enriched | 2 | *AKAP9, ANK2* | 0.0289 | 0.9830 |
| GO:1901407 | regulation of phosphorylation of RNA polymerase II C-terminal domain | 2 | enriched | 1 | *BRD4* | 0.0045 | 0.7660 |
| GO:1901843 | positive regulation of high voltage-gated calcium channel activity | 5 | enriched | 2 | *FGF14, GBAS* | 0.0112 | 0.9124 |
| GO:1902525 | regulation of protein monoubiquitination | 1 | enriched | 1 | *WDR48* | 0.0257 | 0.9830 |
| GO:1902570 | protein localization to nucleolus | 7 | enriched | 1 | *PINX1* | 0.0476 | 0.9830 |
| GO:1902723 | negative regulation of skeletal muscle satellite cell proliferation | 4 | enriched | 1 | *SIX5* | 0.0136 | 0.9830 |
| GO:1902775 | mitochondrial large ribosomal subunit assembly | 3 | enriched | 1 | *FASTKD2* | 0.0062 | 0.7864 |
| GO:1902850 | microtubule cytoskeleton organization involved in mitosis | 2 | enriched | 1 | *SETD2* | 0.0040 | 0.7489 |
| GO:1902938 | regulation of intracellular calcium activated chloride channel activity | 2 | enriched | 1 | *GRM5* | 0.0113 | 0.9124 |
| GO:1902943 | positive regulation of voltage-gated chloride channel activity | 2 | enriched | 1 | *CFTR* | 0.0111 | 0.9124 |
| GO:1902952 | positive regulation of dendritic spine maintenance | 4 | enriched | 1 | *ZNF804A* | 0.0478 | 0.9830 |
| GO:1903008 | organelle disassembly | 2 | enriched | 1 | *KIF9* | 0.0318 | 0.9830 |
| GO:1903061 | positive regulation of protein lipidation | 4 | enriched | 1 | *RAB3GAP2* | 0.0162 | 0.9830 |
| GO:1903373 | positive regulation of endoplasmic reticulum tubular network organization | 4 | enriched | 1 | *RAB3GAP2* | 0.0212 | 0.9830 |
| GO:1903441 | protein localization to ciliary membrane | 5 | enriched | 1 | *ARL3* | 0.0190 | 0.9830 |
| GO:1903538 | regulation of meiotic cell cycle process involved in oocyte maturation | 2 | enriched | 1 | *PPP2R1A* | 0.0294 | 0.9830 |
| GO:1903588 | negative regulation of blood vessel endothelial cell proliferation involved in sprouting angiogenesis | 4 | enriched | 1 | *THBS1* | 0.0096 | 0.9024 |
| GO:1903671 | negative regulation of sprouting angiogenesis | 9 | enriched | 2 | *SEMA6A, THBS1* | 0.0304 | 0.9830 |
| GO:1904358 | positive regulation of telomere maintenance via telomere lengthening | 5 | enriched | 1 | *NAF1* | 0.0414 | 0.9830 |
| GO:1904637 | cellular response to ionomycin | 4 | enriched | 1 | *NFATC4* | 0.0202 | 0.9830 |
| GO:1904684 | negative regulation of metalloendopeptidase activity | 2 | enriched | 1 | *TIMP3* | 0.0463 | 0.9830 |
| GO:1904714 | regulation of chaperone-mediated autophagy | 5 | enriched | 1 | *ATP13A2* | 0.0087 | 0.8491 |
| GO:1904751 | positive regulation of protein localization to nucleolus | 5 | enriched | 1 | *PINX1* | 0.0213 | 0.9830 |
| GO:1904851 | positive regulation of establishment of protein localization to telomere | 10 | enriched | 2 | *CCT6A, CCT8* | 0.0135 | 0.9830 |
| GO:1904871 | positive regulation of protein localization to Cajal body | 9 | enriched | 2 | *CCT6A, CCT8* | 0.0105 | 0.9124 |
| GO:1904874 | positive regulation of telomerase RNA localization to Cajal body | 15 | enriched | 3 | *CCT6A, CCT8, NAF1* | 0.0022 | 0.6905 |
| GO:1905037 | autophagosome organization | 3 | enriched | 1 | *ATP13A2* | 0.0061 | 0.7864 |
| GO:1905123 | regulation of glucosylceramidase activity | 2 | enriched | 1 | *ATP13A2* | 0.0040 | 0.7489 |
| GO:1905165 | regulation of lysosomal protein catabolic process | 2 | enriched | 1 | *ATP13A2* | 0.0041 | 0.7489 |
| GO:1905166 | negative regulation of lysosomal protein catabolic process | 4 | enriched | 1 | *ATP13A2* | 0.0067 | 0.7864 |
| GO:1905323 | telomerase holoenzyme complex assembly | 4 | enriched | 1 | *NAF1* | 0.0243 | 0.9830 |
| GO:1905437 | positive regulation of histone H3-K4 trimethylation | 2 | enriched | 1 | *KMT2E* | 0.0040 | 0.7489 |
| GO:1905799 | regulation of intraciliary retrograde transport | 2 | enriched | 1 | *TCTEX1D2* | 0.0055 | 0.7864 |
| GO:1990001 | inhibition of cysteine-type endopeptidase activity involved in apoptotic process | 7 | enriched | 1 | *BIRC7* | 0.0423 | 0.9830 |
| GO:1990116 | ribosome-associated ubiquitin-dependent protein catabolic process | 2 | enriched | 1 | *LTN1* | 0.0061 | 0.7864 |
| GO:1990575 | mitochondrial L-ornithine transmembrane transport | 3 | enriched | 1 | *SLC25A15* | 0.0065 | 0.7864 |
| GO:2000051 | negative regulation of non-canonical Wnt signaling pathway | 4 | enriched | 2 | *IFT80, ZNRF3* | 0.0008 | 0.4811 |
| GO:2000095 | regulation of Wnt signaling pathway, planar cell polarity pathway | 4 | enriched | 1 | *ZNRF3* | 0.0343 | 0.9830 |
| GO:2000143 | negative regulation of DNA-templated transcription, initiation | 2 | enriched | 1 | *MORC1* | 0.0040 | 0.7489 |
| GO:2000286 | receptor internalization involved in canonical Wnt signaling pathway | 3 | enriched | 1 | *RAB5A* | 0.0340 | 0.9830 |
| GO:2000297 | negative regulation of synapse maturation | 3 | enriched | 1 | *NFATC4* | 0.0077 | 0.8255 |
| GO:2000353 | positive regulation of endothelial cell apoptotic process | 18 | enriched | 3 | *PRKCI, HLA-G, THBS1* | 0.0081 | 0.8321 |
| GO:2000568 | positive regulation of memory T cell activation | 1 | enriched | 1 | *FAM49B* | 0.0371 | 0.9830 |
| GO:2000582 | positive regulation of ATP-dependent microtubule motor activity, plus-end-directed | 5 | enriched | 1 | *DYNLL1* | 0.0184 | 0.9830 |
| GO:2000628 | regulation of miRNA metabolic process | 4 | enriched | 1 | *RC3H2* | 0.0117 | 0.9194 |
| GO:2001027 | negative regulation of endothelial cell chemotaxis | 3 | enriched | 1 | *THBS1* | 0.0016 | 0.6081 |
| GO:2001045 | negative regulation of integrin-mediated signaling pathway | 2 | enriched | 1 | *CTNNA1* | 0.0096 | 0.9024 |
| GO:2001140 | positive regulation of phospholipid transport | 4 | enriched | 1 | *TRIAP1* | 0.0292 | 0.9830 |
| GO:2001184 | positive regulation of interleukin-12 secretion | 4 | enriched | 1 | *MAPK11* | 0.0112 | 0.9124 |
| GO:2001199 | negative regulation of dendritic cell differentiation | 6 | enriched | 1 | *HLA-G* | 0.0296 | 0.9830 |
| GO:2001236 | regulation of extrinsic apoptotic signaling pathway | 4 | enriched | 1 | *TRAF1* | 0.0109 | 0.9124 |
| GO:2001241 | positive regulation of extrinsic apoptotic signaling pathway in absence of ligand | 11 | enriched | 3 | *PPP2R1A, CTNNA1, PPP2R1B* | 0.0004 | 0.3133 |
| GO:2001255 | positive regulation of histone H3-K36 trimethylation | 3 | enriched | 1 | *BRD4* | 0.0050 | 0.7864 |
| GO:2001301 | lipoxin biosynthetic process | 5 | enriched | 1 | *ALOX5* | 0.0218 | 0.9830 |

| **S8b Table. Results of the pathway-based analysis of Mean Anxiety (uncorrected *p-value* < 0.05).** | | | | | | | |
| --- | --- | --- | --- | --- | --- | --- | --- |
| **GO set ID** | **Description** | **Pathway size (nr of genes)** | **Status** | **Significant Genes (nr of)** | **Genes** | ***p-value*** | **FDR** |
| GO:0000045 | autophagosome assembly | 56 | enriched | 6 | *AMBRA1, ATG13, MAP1LC3B, SYNPO2, TP53INP1, TRAPPC8* | 0.0054 | 0.7417 |
| GO:0000154 | rRNA modification | 4 | enriched | 2 | *TFB1M, TSR3* | 0.0002 | 0.2757 |
| GO:0000296 | spermine transport | 1 | enriched | 1 | *TAF7* | 0.0322 | 0.9785 |
| GO:0000422 | autophagy of mitochondrion | 37 | enriched | 4 | *AMBRA1, ATG13, BNIP3, MAP1LC3B* | 0.0081 | 0.7883 |
| GO:0000423 | mitophagy | 6 | enriched | 2 | *AMBRA1, ATG13* | 0.0002 | 0.2757 |
| GO:0000720 | pyrimidine dimer repair by nucleotide-excision repair | 4 | enriched | 1 | *HMGN1* | 0.0211 | 0.9785 |
| GO:0001678 | cellular glucose homeostasis | 19 | enriched | 3 | *FOXO1, NUCKS1, PIK3R1* | 0.0495 | 0.9785 |
| GO:0001836 | release of cytochrome c from mitochondria | 22 | enriched | 2 | *BAK1, BCL2L1* | 0.0465 | 0.9785 |
| GO:0001886 | endothelial cell morphogenesis | 11 | enriched | 2 | *ID1, MET* | 0.0161 | 0.9579 |
| GO:0001915 | negative regulation of T cell mediated cytotoxicity | 6 | enriched | 2 | *IL7R, LILRB1* | 0.0219 | 0.9785 |
| GO:0001960 | negative regulation of cytokine-mediated signaling pathway | 8 | enriched | 2 | *IL36RN, PXDN* | 0.0374 | 0.9785 |
| GO:0002028 | regulation of sodium ion transport | 18 | enriched | 3 | *SLC8A1, DMPK, WNK1* | 0.0356 | 0.9785 |
| GO:0002232 | leukocyte chemotaxis involved in inflammatory response | 5 | enriched | 1 | *MDK* | 0.0278 | 0.9785 |
| GO:0002268 | follicular dendritic cell differentiation | 2 | enriched | 1 | *NFKB2* | 0.0061 | 0.7417 |
| GO:0002352 | B cell negative selection | 2 | enriched | 1 | *BAK1* | 0.0037 | 0.7417 |
| GO:0002367 | cytokine production involved in immune response | 3 | enriched | 1 | *SCIMP* | 0.0180 | 0.9699 |
| GO:0002437 | inflammatory response to antigenic stimulus | 24 | enriched | 4 | *IL1F10, IL1RN, IL36B, IL36RN* | 0.0029 | 0.7162 |
| GO:0002521 | leukocyte differentiation | 3 | enriched | 1 | *RRAS* | 0.0087 | 0.7883 |
| GO:0002553 | histamine secretion by mast cell | 3 | enriched | 1 | *SNAP23* | 0.0145 | 0.9210 |
| GO:0002732 | positive regulation of dendritic cell cytokine production | 2 | enriched | 1 | *SCIMP* | 0.0098 | 0.7883 |
| GO:0003402 | planar cell polarity pathway involved in axis elongation | 4 | depleted | 0 |  | 0.0158 | 0.9513 |
| GO:0006045 | N-acetylglucosamine biosynthetic process | 2 | enriched | 1 | *NANP* | 0.0061 | 0.7417 |
| GO:0006083 | acetate metabolic process | 1 | enriched | 1 | *ALDH5A1* | 0.0351 | 0.9785 |
| GO:0006105 | succinate metabolic process | 7 | enriched | 2 | *ALDH5A1, SDHB* | 0.0093 | 0.7883 |
| GO:0006107 | oxaloacetate metabolic process | 8 | enriched | 2 | *MDH1B, PCK1* | 0.0420 | 0.9785 |
| GO:0006123 | mitochondrial electron transport, cytochrome c to oxygen | 20 | enriched | 3 | *COX4I2, COX6A1, COX7A2L* | 0.0002 | 0.2757 |
| GO:0006206 | pyrimidine nucleobase metabolic process | 2 | enriched | 1 | *TYMP* | 0.0219 | 0.9785 |
| GO:0006213 | pyrimidine nucleoside metabolic process | 3 | enriched | 1 | *TYMP* | 0.0409 | 0.9785 |
| GO:0006261 | DNA-dependent DNA replication | 22 | enriched | 3 | *BAZ1A, POLQ, RFC2* | 0.0131 | 0.9114 |
| GO:0006323 | DNA packaging | 6 | enriched | 1 | *KAT6A* | 0.0394 | 0.9785 |
| GO:0006355 | regulation of transcription, DNA-templated | 781 | enriched | 52 | *ESRRG, RORA, BACH1, ESR1, RCAN1, ACVR2B, CHD7, CHEK2, CREM, FUBP1, GATA4, HKR1, INS, NFATC2, NFKB2, PHF1, PITX3, RNF141, SETD2, SOX7, ZBTB38, ZFAT, ZNF124, ZNF232, ZNF253, ZNF266, ZNF282, ZNF296, ZNF383, ZNF398, ZNF420, ZNF426, ZNF439, ZNF484, ZNF506, ZNF559, ZNF560, ZNF585A, ZNF585B, ZNF669, ZNF670, ZNF679, ZNF682, ZNF695, ZNF699, ZNF713, ZNF727, ZNF736, ZNF747, ZNF768, ZNF786, ZNF850* | 0.0058 | 0.7417 |
| GO:0006398 | mRNA 3'-end processing by stem-loop binding and cleavage | 5 | enriched | 2 | *CPSF3, LSM11* | 0.0398 | 0.9785 |
| GO:0006450 | regulation of translational fidelity | 6 | enriched | 1 | *GATC* | 0.0417 | 0.9785 |
| GO:0006469 | negative regulation of protein kinase activity | 72 | enriched | 8 | *C14orf166, CAMK2N1, FABP4, ITGB1BP1, TAF7, TRIB2, UBASH3B, WNK1* | 0.0176 | 0.9622 |
| GO:0006511 | ubiquitin-dependent protein catabolic process | 274 | enriched | 18 | *BIRC6, CBLC, CDC27, CUL2, FBXL15, FBXL2, FBXO8, KCTD10, NEDD4, NEDD4L, RNF150, RNF167, UBE2I, UBE3B, UBR3, USP16, USP6, ZNRF3* | 0.0417 | 0.9785 |
| GO:0006622 | protein targeting to lysosome | 16 | enriched | 2 | *NEDD4, ZFYVE16* | 0.0250 | 0.9785 |
| GO:0006660 | phosphatidylserine catabolic process | 2 | enriched | 1 | *ABHD12* | 0.0063 | 0.7417 |
| GO:0006681 | galactosylceramide metabolic process | 1 | enriched | 1 | *ALDH5A1* | 0.0351 | 0.9785 |
| GO:0006696 | ergosterol biosynthetic process | 5 | enriched | 1 | *C14orf1* | 0.0492 | 0.9785 |
| GO:0006744 | ubiquinone biosynthetic process | 15 | enriched | 2 | *ADCK3, COQ5* | 0.0308 | 0.9785 |
| GO:0006890 | retrograde vesicle-mediated transport, Golgi to endoplasmic reticulum | 81 | enriched | 7 | *BICD2, GBF1, KIF1C, NBAS, RAB6A, RINT1, SURF4* | 0.0138 | 0.9210 |
| GO:0006892 | post-Golgi vesicle-mediated transport | 19 | enriched | 3 | *GBF1, SNAP23, VAMP8* | 0.0250 | 0.9785 |
| GO:0006903 | vesicle targeting | 8 | enriched | 2 | *CLASP2, SNAP23* | 0.0016 | 0.5962 |
| GO:0006933 | negative regulation of cell adhesion involved in substrate-bound cell migration | 4 | enriched | 1 | *ITGB1BP1* | 0.0169 | 0.9622 |
| GO:0007006 | mitochondrial membrane organization | 4 | enriched | 1 | *MFN2* | 0.0399 | 0.9785 |
| GO:0007018 | microtubule-based movement | 78 | enriched | 12 | *DNAH11, BICD2, DNAH14, KIF13A, KIF16B, KIF17, KIF1C, KIF26B, KIF9, KIFC1, STARD9, WDR34* | 0.0076 | 0.7883 |
| GO:0007030 | Golgi organization | 112 | enriched | 9 | *DYM, CIT, CLASP2, GBF1, MYO18A, RAB2A, RAB7L1, SURF4, TRAPPC8* | 0.0322 | 0.9785 |
| GO:0007156 | homophilic cell adhesion via plasma membrane adhesion molecules | 162 | enriched | 28 | *ROBO2, PCDHGA1, PCDHGA2, PCDHGA3, PCDHGA4, PCDHGA5, PCDHGB1, PCDHGB2, PCDHGB3, CDH12, DCHS2, IGSF21, NEXN, PCDH1, PCDHGA10, PCDHGA11, PCDHGA12, PCDHGA6, PCDHGA7, PCDHGA8, PCDHGA9, PCDHGB4, PCDHGB6, PCDHGB7, PCDHGC3, PCDHGC4, PCDHGC5, TENM3* | 0.0007 | 0.4102 |
| GO:0007165 | signal transduction | 974 | depleted | 56 | *ARHGAP42, COL15A1, ESR1, PAK7, RCAN1, RPS6KA2, RSU1, TENM4, ACVR2B, ADCY8, ALK, ARHGAP25, ARHGAP28, ARHGAP8, BCAM, BRE, CAMK2B, CBLC, CD4, CHRM4, CHRNE, CNGB3, CREM, CSNK1G3, CTNND2, ERBB4, FAM19A5, GABRB2, GABRB3, GPR123, GUCY1A2, IGF1R, IL15, IL7R, IMPA2, INS-IGF2, LILRB1, LILRB4, MAPK10, MDK, MET, PDE9A, PSD, RASL11A, REM1, SH3GL3, TENM3, TGFA, TLE2, TOM1L1, TRIM63, TXNRD1, TYMP, VLDLR, WNT8B, ZFYVE16* | 0.0131 | 0.9114 |
| GO:0007166 | cell surface receptor signaling pathway | 209 | enriched | 21 | *MARCO, ADAP1, CBLC, CD4, CDA, CHRM4, CLEC1B, EDN2, F2, GFRA1, GP1BA, GPR123, IL12RB2, IL7R, LILRA1, MET, MLKL, PRMT1, PTH1R, TDP2, TSPAN12* | 0.0347 | 0.9785 |
| GO:0007195 | adenylate cyclase-inhibiting dopamine receptor signaling pathway | 9 | enriched | 3 | *ADCY5, DRD3, OR13F1* | 0.0027 | 0.7162 |
| GO:0007518 | myoblast fate determination | 2 | enriched | 1 | *IFRD1* | 0.0056 | 0.7417 |
| GO:0008053 | mitochondrial fusion | 16 | enriched | 3 | *BAK1, FAM73A, MFN2* | 0.0062 | 0.7417 |
| GO:0008582 | regulation of synaptic growth at neuromuscular junction | 4 | enriched | 2 | *MUSK, SIX4* | 0.0004 | 0.3036 |
| GO:0008594 | photoreceptor cell morphogenesis | 3 | enriched | 1 | *C8orf37* | 0.0194 | 0.9785 |
| GO:0008630 | intrinsic apoptotic signaling pathway in response to DNA damage | 45 | enriched | 6 | *BAK1, BCL2L1, CHEK2, MOAP1, NFATC4, PIK3R1* | 0.0108 | 0.8151 |
| GO:0008635 | activation of cysteine-type endopeptidase activity involved in apoptotic process by cytochrome c | 7 | enriched | 2 | *APAF1, BAK1* | 0.0008 | 0.4382 |
| GO:0008637 | apoptotic mitochondrial changes | 18 | enriched | 3 | *BCL2L1, NDUFS1, PPP2CB* | 0.0171 | 0.9622 |
| GO:0008645 | hexose transmembrane transport | 13 | enriched | 4 | *SLC2A8, SLC45A3, SLC5A10, SLC5A9* | 0.0016 | 0.5962 |
| GO:0009060 | aerobic respiration | 30 | enriched | 4 | *COX6A1, SDHB, SIRT3, SURF1* | 0.0228 | 0.9785 |
| GO:0009236 | cobalamin biosynthetic process | 3 | enriched | 1 | *MMAB* | 0.0108 | 0.8151 |
| GO:0009395 | phospholipid catabolic process | 9 | enriched | 2 | *ABHD12, LIPG* | 0.0285 | 0.9785 |
| GO:0009450 | gamma-aminobutyric acid catabolic process | 2 | enriched | 1 | *ALDH5A1* | 0.0260 | 0.9785 |
| GO:0009620 | response to fungus | 6 | enriched | 1 | *BAK1* | 0.0448 | 0.9785 |
| GO:0009946 | proximal/distal axis specification | 1 | enriched | 1 | *SIX3* | 0.0274 | 0.9785 |
| GO:0010046 | response to mycotoxin | 3 | enriched | 1 | *BAK1* | 0.0083 | 0.7883 |
| GO:0010225 | response to UV-C | 10 | enriched | 2 | *BAK1, HMGN1* | 0.0027 | 0.7162 |
| GO:0010458 | exit from mitosis | 12 | enriched | 2 | *CLASP2, PPP2R2D* | 0.0091 | 0.7883 |
| GO:0010466 | negative regulation of peptidase activity | 16 | enriched | 3 | *SERPINB3, SERPINB4, UMODL1* | 0.0100 | 0.7883 |
| GO:0010569 | regulation of double-strand break repair via homologous recombination | 18 | enriched | 3 | *ZNF365, SETD2, TEX15* | 0.0195 | 0.9785 |
| GO:0010621 | negative regulation of transcription by transcription factor localization | 3 | enriched | 1 | *ID1* | 0.0056 | 0.7417 |
| GO:0010637 | negative regulation of mitochondrial fusion | 7 | enriched | 2 | *BNIP3, MUL1* | 0.0041 | 0.7417 |
| GO:0010659 | cardiac muscle cell apoptotic process | 4 | enriched | 2 | *APAF1, BNIP3* | 0.0018 | 0.5962 |
| GO:0010667 | negative regulation of cardiac muscle cell apoptotic process | 21 | enriched | 3 | *AMBRA1, GATA4, MDK* | 0.0457 | 0.9785 |
| GO:0010766 | negative regulation of sodium ion transport | 6 | enriched | 2 | *NEDD4, WNK1* | 0.0031 | 0.7162 |
| GO:0010768 | negative regulation of transcription from RNA polymerase II promoter in response to UV-induced DNA damage | 2 | enriched | 1 | *NEDD4* | 0.0163 | 0.9605 |
| GO:0010821 | regulation of mitochondrion organization | 16 | enriched | 3 | *ATP13A2, MUL1, PDE2A* | 0.0193 | 0.9785 |
| GO:0010830 | regulation of myotube differentiation | 5 | enriched | 2 | *DMPK, HDAC5* | 0.0002 | 0.2757 |
| GO:0010961 | cellular magnesium ion homeostasis | 5 | enriched | 1 | *SLC41A1* | 0.0458 | 0.9785 |
| GO:0010996 | response to auditory stimulus | 14 | enriched | 3 | *ABHD12, KIAA0319, MDK* | 0.0001 | 0.2757 |
| GO:0014016 | neuroblast differentiation | 3 | enriched | 2 | *BCHE, SIX3* | 0.0006 | 0.3872 |
| GO:0014706 | striated muscle tissue development | 5 | enriched | 2 | *EYA1, IFRD1* | 0.0205 | 0.9785 |
| GO:0014718 | positive regulation of satellite cell activation involved in skeletal muscle regeneration | 2 | enriched | 1 | *CAPN3* | 0.0057 | 0.7417 |
| GO:0014816 | skeletal muscle satellite cell differentiation | 5 | enriched | 2 | *CDON, MYLK2* | 0.0134 | 0.9154 |
| GO:0014819 | regulation of skeletal muscle contraction | 4 | depleted | 0 |  | 0.0460 | 0.9785 |
| GO:0015711 | organic anion transport | 14 | enriched | 4 | *SLC22A10, SLC22A24, SLC22A25, SLC22A9* | 0.0168 | 0.9622 |
| GO:0015742 | alpha-ketoglutarate transport | 4 | enriched | 1 | *SLC25A11* | 0.0181 | 0.9699 |
| GO:0015770 | sucrose transport | 4 | enriched | 1 | *SLC45A3* | 0.0251 | 0.9785 |
| GO:0016071 | mRNA metabolic process | 5 | enriched | 1 | *TBRG4* | 0.0303 | 0.9785 |
| GO:0016180 | snRNA processing | 13 | enriched | 4 | *INTS10, INTS6, INTS7, INTS8* | 0.0109 | 0.8179 |
| GO:0016601 | Rac protein signal transduction | 21 | enriched | 3 | *FARP2, NCKAP1, WASF2* | 0.0103 | 0.7883 |
| GO:0018125 | peptidyl-cysteine methylation | 3 | enriched | 1 | *RAB6A* | 0.0198 | 0.9785 |
| GO:0018406 | protein C-linked glycosylation via 2'-alpha-mannosyl-L-tryptophan | 5 | enriched | 1 | *DPY19L4* | 0.0384 | 0.9785 |
| GO:0018872 | arsonoacetate metabolic process | 2 | enriched | 1 | *AS3MT* | 0.0056 | 0.7417 |
| GO:0019046 | release from viral latency | 2 | enriched | 1 | *NUCKS1* | 0.0090 | 0.7883 |
| GO:0019050 | suppression by virus of host apoptotic process | 2 | enriched | 1 | *BCL2L1* | 0.0061 | 0.7417 |
| GO:0019287 | isopentenyl diphosphate biosynthetic process, mevalonate pathway | 3 | enriched | 1 | *MVK* | 0.0170 | 0.9622 |
| GO:0019695 | choline metabolic process | 4 | enriched | 1 | *BCHE* | 0.0260 | 0.9785 |
| GO:0019732 | antifungal humoral response | 5 | enriched | 1 | *IL36RN* | 0.0213 | 0.9785 |
| GO:0021520 | spinal cord motor neuron cell fate specification | 7 | enriched | 1 | *ISL2* | 0.0432 | 0.9785 |
| GO:0021524 | visceral motor neuron differentiation | 3 | enriched | 1 | *ISL2* | 0.0086 | 0.7883 |
| GO:0021542 | dentate gyrus development | 16 | enriched | 2 | *EMX2, MDK* | 0.0148 | 0.9327 |
| GO:0021681 | cerebellar granular layer development | 3 | enriched | 1 | *MDK* | 0.0103 | 0.7883 |
| GO:0021842 | chemorepulsion involved in interneuron migration from the subpallium to the cortex | 2 | depleted | 0 |  | 0.0303 | 0.9785 |
| GO:0021846 | cell proliferation in forebrain | 12 | enriched | 3 | *DISC1, EMX2, SIX3* | 0.0302 | 0.9785 |
| GO:0021885 | forebrain cell migration | 3 | enriched | 1 | *EMX2* | 0.0090 | 0.7883 |
| GO:0022904 | respiratory electron transport chain | 17 | enriched | 4 | *ALDH5A1, ETFA, IMMP2L, SDHB* | 0.0420 | 0.9785 |
| GO:0023016 | signal transduction by trans-phosphorylation | 3 | enriched | 1 | *WNK1* | 0.0376 | 0.9785 |
| GO:0030003 | cellular cation homeostasis | 2 | enriched | 1 | *ATP13A2* | 0.0053 | 0.7417 |
| GO:0030162 | regulation of proteolysis | 12 | enriched | 2 | *SERPINB13, SERPINB4* | 0.0069 | 0.7540 |
| GO:0030193 | regulation of blood coagulation | 8 | enriched | 2 | *F2, GP1BA* | 0.0037 | 0.7417 |
| GO:0030330 | DNA damage response, signal transduction by p53 class mediator | 16 | enriched | 2 | *MYO6, TRIAP1* | 0.0341 | 0.9785 |
| GO:0030423 | targeting of mRNA for destruction involved in RNA interference | 4 | enriched | 1 | *DHX9* | 0.0177 | 0.9622 |
| GO:0030517 | negative regulation of axon extension | 17 | enriched | 3 | *IFRD1, KIAA0319, RTN4* | 0.0069 | 0.7540 |
| GO:0030702 | chromatin silencing at centromere | 2 | enriched | 1 | *ZNFX1* | 0.0064 | 0.7417 |
| GO:0030837 | negative regulation of actin filament polymerization | 17 | enriched | 4 | *KANK1, PFN1, SSH2, TWF1* | 0.0308 | 0.9785 |
| GO:0030910 | olfactory placode formation | 3 | enriched | 1 | *SIX4* | 0.0094 | 0.7883 |
| GO:0030948 | negative regulation of vascular endothelial growth factor receptor signaling pathway | 13 | enriched | 2 | *NEDD4, PTPN1* | 0.0178 | 0.9622 |
| GO:0030961 | peptidyl-arginine hydroxylation | 1 | enriched | 1 | *NDUFAF5* | 0.0390 | 0.9785 |
| GO:0031048 | chromatin silencing by small RNA | 3 | enriched | 1 | *ZNFX1* | 0.0092 | 0.7883 |
| GO:0031052 | chromosome breakage | 2 | enriched | 1 | *RFWD3* | 0.0064 | 0.7417 |
| GO:0031293 | membrane protein intracellular domain proteolysis | 18 | enriched | 3 | *ADAM17, HM13, PSEN2* | 0.0211 | 0.9785 |
| GO:0031365 | N-terminal protein amino acid modification | 4 | enriched | 1 | *METAP1* | 0.0362 | 0.9785 |
| GO:0031532 | actin cytoskeleton reorganization | 55 | enriched | 7 | *CDC42BPA, FER, FARP2, GPR65, MINK1, PHACTR1, PTPN1* | 0.0436 | 0.9785 |
| GO:0031571 | mitotic G1 DNA damage checkpoint | 8 | enriched | 2 | *FBXO31, RFWD3* | 0.0009 | 0.4459 |
| GO:0032012 | regulation of ARF protein signal transduction | 17 | enriched | 6 | *ARFGEF2, FBXO8, GBF1, IQSEC3, PSD, PSD3* | 0.0017 | 0.5962 |
| GO:0032053 | ciliary basal body organization | 3 | enriched | 2 | *CROCC, RTTN* | 0.0032 | 0.7162 |
| GO:0032100 | positive regulation of appetite | 4 | enriched | 1 | *NPY* | 0.0142 | 0.9210 |
| GO:0032147 | activation of protein kinase activity | 92 | enriched | 10 | *PAK7, ACVR2B, ATG13, MAP3K1, MAP3K8, MINK1, TGFBR2, TOM1L1, TPX2, WNK1* | 0.0146 | 0.9269 |
| GO:0032232 | negative regulation of actin filament bundle assembly | 4 | enriched | 1 | *PFN1* | 0.0139 | 0.9210 |
| GO:0032233 | positive regulation of actin filament bundle assembly | 13 | enriched | 3 | *ID1, PFN1, SYNPO2* | 0.0044 | 0.7417 |
| GO:0032258 | cytoplasm to vacuole transport by the Cvt pathway | 2 | enriched | 1 | *TRAPPC8* | 0.0129 | 0.9114 |
| GO:0032259 | methylation | 54 | enriched | 7 | *AS3MT, COQ5, METTL5, NDUFAF5, PRDM8, SETD9, TPMT* | 0.0005 | 0.3125 |
| GO:0032330 | regulation of chondrocyte differentiation | 10 | enriched | 3 | *GLG1, KIAA1715, MDK* | <0.0001 | 0.2757 |
| GO:0032465 | regulation of cytokinesis | 41 | enriched | 5 | *AHCTF1, BCL2L1, BIRC6, KIF13A, SETD2* | 0.0021 | 0.6556 |
| GO:0032727 | positive regulation of interferon-alpha production | 12 | enriched | 2 | *IRF3, SETD2* | 0.0085 | 0.7883 |
| GO:0032741 | positive regulation of interleukin-18 production | 4 | enriched | 1 | *DHX9* | 0.0165 | 0.9622 |
| GO:0032781 | positive regulation of ATPase activity | 40 | enriched | 6 | *DNAJB4, HSCB, MYL3, MYL4, PFN1, TOR1AIP1* | 0.0193 | 0.9785 |
| GO:0032886 | regulation of microtubule-based process | 9 | enriched | 3 | *CLASP2, DIAPH1, MACF1* | 0.0117 | 0.8588 |
| GO:0032971 | regulation of muscle filament sliding | 2 | enriched | 1 | *MYLK2* | 0.0052 | 0.7417 |
| GO:0033132 | negative regulation of glucokinase activity | 5 | enriched | 1 | *COX11* | 0.0189 | 0.9785 |
| GO:0033145 | positive regulation of intracellular steroid hormone receptor signaling pathway | 1 | enriched | 1 | *UBE2I* | 0.0306 | 0.9785 |
| GO:0033157 | regulation of intracellular protein transport | 7 | enriched | 2 | *ATP13A2, PTPN1* | 0.0018 | 0.5962 |
| GO:0033633 | negative regulation of cell-cell adhesion mediated by integrin | 2 | enriched | 1 | *WNK1* | 0.0249 | 0.9785 |
| GO:0034067 | protein localization to Golgi apparatus | 20 | enriched | 3 | *BICD2, GBF1, RAB6A* | 0.0016 | 0.5962 |
| GO:0034154 | toll-like receptor 7 signaling pathway | 5 | enriched | 1 | *SCIMP* | 0.0363 | 0.9785 |
| GO:0034453 | microtubule anchoring | 13 | enriched | 2 | *CEP350, CLASP2* | 0.0192 | 0.9785 |
| GO:0034472 | snRNA 3'-end processing | 9 | enriched | 3 | *INTS6, INTS7, INTS8* | 0.0022 | 0.6720 |
| GO:0034497 | protein localization to phagophore assembly site | 12 | enriched | 3 | *ATG13, MFN2, TRAPPC8* | 0.0004 | 0.3036 |
| GO:0034498 | early endosome to Golgi transport | 12 | enriched | 2 | *RAB6A, SURF4* | 0.0475 | 0.9785 |
| GO:0034644 | cellular response to UV | 49 | enriched | 6 | *BAK1, NEDD4, NFATC4, PIK3R1, TP53INP1, TRIAP1* | 0.0001 | 0.2757 |
| GO:0034727 | piecemeal microautophagy of the nucleus | 3 | enriched | 1 | *ATG13* | 0.0100 | 0.7883 |
| GO:0034728 | nucleosome organization | 3 | enriched | 1 | *SETD2* | 0.0142 | 0.9210 |
| GO:0035063 | nuclear speck organization | 4 | enriched | 1 | *DYRK3* | 0.0199 | 0.9785 |
| GO:0035425 | autocrine signaling | 7 | enriched | 1 | *SERPINB3* | 0.0357 | 0.9785 |
| GO:0035441 | cell migration involved in vasculogenesis | 2 | enriched | 1 | *SETD2* | 0.0064 | 0.7417 |
| GO:0035522 | monoubiquitinated histone H2A deubiquitination | 4 | enriched | 1 | *USP16* | 0.0215 | 0.9785 |
| GO:0035630 | bone mineralization involved in bone maturation | 6 | enriched | 1 | *CCDC154* | 0.0474 | 0.9785 |
| GO:0036151 | phosphatidylcholine acyl-chain remodeling | 27 | enriched | 5 | *DBI, PLA2G4A, PLA2G6, PLB1, PNPLA8* | 0.0149 | 0.9327 |
| GO:0038018 | Wnt receptor catabolic process | 2 | enriched | 1 | *ZNRF3* | 0.0192 | 0.9785 |
| GO:0038123 | toll-like receptor TLR1:TLR2 signaling pathway | 4 | enriched | 1 | *SCIMP* | 0.0167 | 0.9622 |
| GO:0038129 | ERBB3 signaling pathway | 1 | depleted | 0 |  | 0.0478 | 0.9785 |
| GO:0039530 | MDA-5 signaling pathway | 3 | enriched | 1 | *IRF3* | 0.0134 | 0.9154 |
| GO:0040012 | regulation of locomotion | 6 | enriched | 1 | *CHRM4* | 0.0468 | 0.9785 |
| GO:0040020 | regulation of meiotic nuclear division | 5 | enriched | 2 | *GPR3, PDE3A* | 0.0203 | 0.9785 |
| GO:0042048 | olfactory behavior | 7 | enriched | 2 | *CHD7, UBR3* | 0.0144 | 0.9210 |
| GO:0042147 | retrograde transport, endosome to Golgi | 74 | enriched | 9 | *BAIAP3, BET1L, DENND2A, GBF1, GOSR2, RAB6A, RAB7L1, RP11-156P1.2, TMEM87A* | 0.0127 | 0.9078 |
| GO:0042264 | peptidyl-aspartic acid hydroxylation | 2 | enriched | 1 | *HIF1AN* | 0.0064 | 0.7417 |
| GO:0042270 | protection from natural killer cell mediated cytotoxicity | 5 | enriched | 1 | *SERPINB4* | 0.0165 | 0.9622 |
| GO:0042742 | defense response to bacterium | 124 | enriched | 9 | *ANXA3, DEFB115, DEFB116, DEFB118, DEFB119, DEFB121, DEFB123, DEFB124, LYZ* | 0.0025 | 0.7053 |
| GO:0042780 | tRNA 3'-end processing | 4 | enriched | 1 | *SSB* | 0.0437 | 0.9785 |
| GO:0042921 | glucocorticoid receptor signaling pathway | 5 | enriched | 1 | *NEDD4* | 0.0442 | 0.9785 |
| GO:0043000 | Golgi to plasma membrane CFTR protein transport | 2 | enriched | 1 | *KRT18* | 0.0052 | 0.7417 |
| GO:0043243 | positive regulation of protein complex disassembly | 5 | enriched | 2 | *BNIP3, IGF1R* | 0.0274 | 0.9785 |
| GO:0043266 | regulation of potassium ion transport | 9 | enriched | 2 | *DRD3, OR13F1* | 0.0482 | 0.9785 |
| GO:0043279 | response to alkaloid | 6 | enriched | 2 | *BCHE, SRSF9* | 0.0002 | 0.2757 |
| GO:0043379 | memory T cell differentiation | 1 | enriched | 1 | *TSC1* | 0.0347 | 0.9785 |
| GO:0043392 | negative regulation of DNA binding | 32 | enriched | 3 | *ID1, IFI16, WFIKKN2* | 0.0479 | 0.9785 |
| GO:0043403 | skeletal muscle tissue regeneration | 17 | enriched | 2 | *ENO3, IFRD1* | 0.0157 | 0.9483 |
| GO:0043408 | regulation of MAPK cascade | 22 | enriched | 5 | *ID1, MUSK, SYNGAP1, ULK4, VRK2* | 0.0003 | 0.2757 |
| GO:0043496 | regulation of protein homodimerization activity | 5 | enriched | 1 | *BAK1* | 0.0259 | 0.9785 |
| GO:0043538 | regulation of actin phosphorylation | 1 | enriched | 1 | *TWF1* | 0.0277 | 0.9785 |
| GO:0043651 | linoleic acid metabolic process | 17 | enriched | 2 | *ELOVL3, PNPLA8* | 0.0320 | 0.9785 |
| GO:0043666 | regulation of phosphoprotein phosphatase activity | 41 | enriched | 7 | *RCAN1, DMPK, PPP1R3B, PPP1R7, PPP2R2B, PPP2R2D, TSC1* | 0.0172 | 0.9622 |
| GO:0043687 | post-translational protein modification | 334 | depleted | 10 | *SUMF1, FSTL1, CUL2, FBXL15, FBXO31, IGFBP7, LTBP1, PSMA1, RAB2A, WSB2* | 0.0397 | 0.9785 |
| GO:0044346 | fibroblast apoptotic process | 3 | enriched | 1 | *BAK1* | 0.0095 | 0.7883 |
| GO:0044528 | regulation of mitochondrial mRNA stability | 4 | enriched | 1 | *TBRG4* | 0.0151 | 0.9329 |
| GO:0044773 | mitotic DNA damage checkpoint | 3 | enriched | 2 | *CHEK2, STK33* | 0.0263 | 0.9785 |
| GO:0044830 | modulation by host of viral RNA genome replication | 2 | enriched | 1 | *FBXL2* | 0.0064 | 0.7417 |
| GO:0045081 | negative regulation of interleukin-10 biosynthetic process | 2 | enriched | 1 | *TRIB2* | 0.0047 | 0.7417 |
| GO:0045214 | sarcomere organization | 40 | enriched | 7 | *ACTN2, CAPN3, CSRP1, KRT8, MYOM1, MYOZ2, SIX4* | 0.0069 | 0.7540 |
| GO:0045299 | otolith mineralization | 2 | enriched | 1 | *OTOL1* | 0.0063 | 0.7417 |
| GO:0045333 | cellular respiration | 17 | enriched | 3 | *COX4I2, NDUFS1, NFATC4* | 0.0122 | 0.8811 |
| GO:0045351 | type I interferon biosynthetic process | 2 | enriched | 1 | *IRF3* | 0.0049 | 0.7417 |
| GO:0045590 | negative regulation of regulatory T cell differentiation | 5 | enriched | 1 | *MDK* | 0.0189 | 0.9785 |
| GO:0045602 | negative regulation of endothelial cell differentiation | 6 | enriched | 2 | *ID1, XDH* | 0.0078 | 0.7883 |
| GO:0045786 | negative regulation of cell cycle | 40 | enriched | 5 | *RPS6KA2, CAMK2N1, LILRB1, RNF167, THAP5* | 0.0244 | 0.9785 |
| GO:0045824 | negative regulation of innate immune response | 11 | enriched | 2 | *IFI16, MUL1* | 0.0208 | 0.9785 |
| GO:0045861 | negative regulation of proteolysis | 22 | enriched | 4 | *CAMK2N1, F2, INS, SERPINB3* | 0.0066 | 0.7432 |
| GO:0045862 | positive regulation of proteolysis | 19 | enriched | 2 | *BAK1, CAPN3* | 0.0356 | 0.9785 |
| GO:0045901 | positive regulation of translational elongation | 4 | enriched | 1 | *USP16* | 0.0136 | 0.9210 |
| GO:0045943 | positive regulation of transcription by RNA polymerase I | 12 | enriched | 2 | *HEATR1, RASL11A* | 0.0396 | 0.9785 |
| GO:0046040 | IMP metabolic process | 4 | enriched | 1 | *NT5C2* | 0.0341 | 0.9785 |
| GO:0046338 | phosphatidylethanolamine catabolic process | 2 | enriched | 1 | *PNPLA8* | 0.0099 | 0.7883 |
| GO:0046459 | short-chain fatty acid metabolic process | 2 | enriched | 1 | *ALDH5A1* | 0.0178 | 0.9622 |
| GO:0046464 | acylglycerol catabolic process | 4 | enriched | 1 | *ABHD12* | 0.0301 | 0.9785 |
| GO:0046580 | negative regulation of Ras protein signal transduction | 26 | enriched | 4 | *MFN2, PPP2CB, SYNGAP1, TIMP2* | 0.0183 | 0.9727 |
| GO:0046626 | regulation of insulin receptor signaling pathway | 12 | enriched | 2 | *NUCKS1, PIK3R1* | 0.0078 | 0.7883 |
| GO:0046661 | male sex differentiation | 4 | enriched | 1 | *SIX4* | 0.0177 | 0.9622 |
| GO:0046726 | positive regulation by virus of viral protein levels in host cell | 4 | enriched | 1 | *STAU1* | 0.0291 | 0.9785 |
| GO:0046850 | regulation of bone remodeling | 5 | enriched | 1 | *MDK* | 0.0322 | 0.9785 |
| GO:0046902 | regulation of mitochondrial membrane permeability | 11 | enriched | 3 | *BAK1, BCL2L1, BNIP3* | 0.0002 | 0.2757 |
| GO:0048050 | post-embryonic eye morphogenesis | 3 | enriched | 1 | *MFAP2* | 0.0103 | 0.7883 |
| GO:0048102 | autophagic cell death | 7 | enriched | 2 | *BNIP3, TP53INP1* | 0.0032 | 0.7162 |
| GO:0048205 | COPI coating of Golgi vesicle | 6 | enriched | 1 | *GBF1* | 0.0472 | 0.9785 |
| GO:0048332 | mesoderm morphogenesis | 2 | enriched | 1 | *SETD2* | 0.0064 | 0.7417 |
| GO:0048514 | blood vessel morphogenesis | 19 | enriched | 3 | *FLT1, ID1, NEDD4* | 0.0463 | 0.9785 |
| GO:0048597 | post-embryonic camera-type eye morphogenesis | 3 | enriched | 2 | *BAK1, HMGN1* | 0.0007 | 0.4102 |
| GO:0048671 | negative regulation of collateral sprouting | 7 | enriched | 1 | *IFRD1* | 0.0407 | 0.9785 |
| GO:0048692 | negative regulation of axon extension involved in regeneration | 2 | enriched | 1 | *KIAA0319* | 0.0068 | 0.7540 |
| GO:0048713 | regulation of oligodendrocyte differentiation | 3 | enriched | 1 | *SLC45A3* | 0.0142 | 0.9210 |
| GO:0048747 | muscle fiber development | 18 | enriched | 2 | *SGCB, WFIKKN2* | 0.0257 | 0.9785 |
| GO:0048856 | anatomical structure development | 48 | enriched | 8 | *SPOCK3, EYA1, HNF4G, IGF1R, SIX3, SIX4, SIX5, SPOCK1* | 0.0239 | 0.9785 |
| GO:0048864 | stem cell development | 5 | enriched | 1 | *SETD2* | 0.0292 | 0.9785 |
| GO:0050678 | regulation of epithelial cell proliferation | 14 | enriched | 2 | *HMGN1, SIX4* | 0.0099 | 0.7883 |
| GO:0050689 | negative regulation of defense response to virus by host | 3 | enriched | 1 | *MUL1* | 0.0066 | 0.7432 |
| GO:0050691 | regulation of defense response to virus by host | 7 | enriched | 2 | *DHX9, IL15* | 0.0460 | 0.9785 |
| GO:0050774 | negative regulation of dendrite morphogenesis | 9 | enriched | 2 | *ID1, NFATC4* | 0.0004 | 0.3036 |
| GO:0050794 | regulation of cellular process | 2 | enriched | 1 | *WNK1* | 0.0184 | 0.9727 |
| GO:0050795 | regulation of behavior | 10 | enriched | 4 | *RELN, AHI1, HTR1B, MDK* | 0.0019 | 0.6370 |
| GO:0050808 | synapse organization | 46 | enriched | 8 | *C1QA, CTNND2, PCDHGC3, PCDHGC4, PCDHGC5, PPFIA4, TSC1, WRB* | 0.0014 | 0.5962 |
| GO:0050908 | detection of light stimulus involved in visual perception | 18 | depleted | 0 |  | 0.0437 | 0.9785 |
| GO:0051046 | regulation of secretion | 2 | enriched | 1 | *MYO6* | 0.0115 | 0.8500 |
| GO:0051301 | cell division | 342 | enriched | 29 | *PARD3B, AHCTF1, BIRC6, BRE, CABLES1, CCNE2, CDC27, CDK14, CHEK2, CINP, CKAP5, CLASP2, DYRK3, HAUS2, KIF13A, KIFC1, MAD1L1, MARK4, MPLKIP, NUF2, PDS5A, PPP2R2D, RCC2, SKA3, SYCE3, TPX2, UBE2I, USP16, USP39* | 0.0082 | 0.7883 |
| GO:0051451 | myoblast migration | 9 | enriched | 2 | *ITGB1BP1, SIX4* | 0.0015 | 0.5962 |
| GO:0051497 | negative regulation of stress fiber assembly | 26 | enriched | 5 | *ARHGAP28, CLASP2, MET, PFN1, WASF2* | 0.0009 | 0.4623 |
| GO:0051646 | mitochondrion localization | 8 | enriched | 2 | *MFN2, MUL1* | 0.0017 | 0.5962 |
| GO:0051695 | actin filament uncapping | 1 | enriched | 1 | *ACTN2* | 0.0342 | 0.9785 |
| GO:0051823 | regulation of synapse structural plasticity | 5 | enriched | 3 | *CAMK2B, CTNNA2, DMPK* | 0.0151 | 0.9329 |
| GO:0051838 | cytolysis by host of symbiont cells | 1 | enriched | 1 | *F2* | 0.0319 | 0.9785 |
| GO:0051881 | regulation of mitochondrial membrane potential | 28 | enriched | 4 | *BAK1, BCL2L1, MUL1, NDUFS1* | 0.0021 | 0.6638 |
| GO:0051895 | negative regulation of focal adhesion assembly | 18 | enriched | 4 | *CLASP2, ITGB1BP1, MMP14, RCC2* | 0.0003 | 0.2757 |
| GO:0051967 | negative regulation of synaptic transmission, glutamatergic | 14 | enriched | 5 | *DRD3, HTR1B, HTR2A, OR13F1, PLA2G6* | 0.0377 | 0.9785 |
| GO:0051969 | regulation of transmission of nerve impulse | 4 | enriched | 1 | *TYMP* | 0.0464 | 0.9785 |
| GO:0051987 | positive regulation of attachment of spindle microtubules to kinetochore | 3 | enriched | 1 | *RCC2* | 0.0129 | 0.9114 |
| GO:0052651 | monoacylglycerol catabolic process | 5 | enriched | 1 | *ABHD12* | 0.0358 | 0.9785 |
| GO:0052746 | inositol phosphorylation | 2 | enriched | 1 | *IPPK* | 0.0064 | 0.7417 |
| GO:0055001 | muscle cell development | 4 | enriched | 1 | *NFATC4* | 0.0459 | 0.9785 |
| GO:0055013 | cardiac muscle cell development | 9 | enriched | 3 | *SLC8A1, ACTN2, SGCB* | 0.0031 | 0.7162 |
| GO:0055065 | metal ion homeostasis | 3 | enriched | 1 | *COX11* | 0.0211 | 0.9785 |
| GO:0055069 | zinc ion homeostasis | 5 | enriched | 1 | *ATP13A2* | 0.0215 | 0.9785 |
| GO:0055085 | transmembrane transport | 282 | depleted | 11 | *LRRC8B, SLC35F4, ABCG5, FLVCR2, GJA8, PSMA1, SLC22A10, SLC22A24, SLC22A25, SLC44A5, SLC6A3* | 0.0435 | 0.9785 |
| GO:0060154 | cellular process regulating host cell cycle in response to virus | 3 | enriched | 1 | *BCL2L1* | 0.0198 | 0.9785 |
| GO:0060260 | regulation of transcription initiation from RNA polymerase II promoter | 2 | enriched | 1 | *TAF7* | 0.0153 | 0.9407 |
| GO:0060426 | lung vasculature development | 6 | enriched | 1 | *ID1* | 0.0313 | 0.9785 |
| GO:0060575 | intestinal epithelial cell differentiation | 9 | enriched | 3 | *CDX2, GATA4, NPY* | 0.0132 | 0.9114 |
| GO:0060628 | regulation of ER to Golgi vesicle-mediated transport | 6 | enriched | 1 | *RINT1* | 0.0482 | 0.9785 |
| GO:0060669 | embryonic placenta morphogenesis | 6 | enriched | 2 | *SETD2, ZNF568* | 0.0197 | 0.9785 |
| GO:0060711 | labyrinthine layer development | 7 | enriched | 2 | *BIRC6, CDX2* | 0.0219 | 0.9785 |
| GO:0060836 | lymphatic endothelial cell differentiation | 6 | enriched | 1 | *ACVR2B* | 0.0474 | 0.9785 |
| GO:0060841 | venous blood vessel development | 4 | enriched | 1 | *ACVR2B* | 0.0186 | 0.9785 |
| GO:0060977 | coronary vasculature morphogenesis | 3 | enriched | 2 | *SETD2, SGCD* | 0.0060 | 0.7417 |
| GO:0061055 | myotome development | 3 | enriched | 1 | *SIX4* | 0.0100 | 0.7883 |
| GO:0061086 | negative regulation of histone H3-K27 methylation | 4 | enriched | 1 | *PHF1* | 0.0257 | 0.9785 |
| GO:0061087 | positive regulation of histone H3-K27 methylation | 5 | enriched | 1 | *PHF1* | 0.0366 | 0.9785 |
| GO:0061088 | regulation of sequestering of zinc ion | 5 | enriched | 1 | *SLC30A2* | 0.0342 | 0.9785 |
| GO:0061162 | establishment of monopolar cell polarity | 2 | enriched | 1 | *GBF1* | 0.0088 | 0.7883 |
| GO:0061197 | fungiform papilla morphogenesis | 2 | enriched | 1 | *SIX4* | 0.0125 | 0.8980 |
| GO:0061428 | negative regulation of transcription from RNA polymerase II promoter in response to hypoxia | 4 | enriched | 1 | *HIF1AN* | 0.0143 | 0.9210 |
| GO:0061551 | trigeminal ganglion development | 5 | enriched | 2 | *SEMA3A, SIX4* | 0.0061 | 0.7417 |
| GO:0061640 | cytoskeleton-dependent cytokinesis | 22 | enriched | 4 | *CECR2, ROPN1B, SEPT14, SEPT2* | 0.0025 | 0.7053 |
| GO:0070084 | protein initiator methionine removal | 3 | enriched | 1 | *METAP1* | 0.0280 | 0.9785 |
| GO:0070124 | mitochondrial translational initiation | 2 | enriched | 1 | *MTIF3* | 0.0321 | 0.9785 |
| GO:0070242 | thymocyte apoptotic process | 5 | enriched | 2 | *BAK1, GLI3* | 0.0287 | 0.9785 |
| GO:0070262 | peptidyl-serine dephosphorylation | 15 | enriched | 3 | *PPP2CB, PPP2R2B, PPP2R2D* | 0.0195 | 0.9785 |
| GO:0070269 | pyroptosis | 10 | enriched | 2 | *AIM2, DHX9* | 0.0388 | 0.9785 |
| GO:0070315 | G1 to G0 transition involved in cell differentiation | 3 | enriched | 1 | *CAPN3* | 0.0085 | 0.7883 |
| GO:0070537 | histone H2A K63-linked deubiquitination | 3 | enriched | 1 | *USP16* | 0.0139 | 0.9210 |
| GO:0070586 | cell-cell adhesion involved in gastrulation | 1 | enriched | 1 | *RIC8A* | 0.0253 | 0.9785 |
| GO:0070681 | glutaminyl-tRNAGln biosynthesis via transamidation | 3 | enriched | 1 | *GATC* | 0.0103 | 0.7883 |
| GO:0070838 | divalent metal ion transport | 2 | enriched | 1 | *SLC41A1* | 0.0064 | 0.7417 |
| GO:0070895 | negative regulation of transposon integration | 2 | enriched | 1 | *ZNF93* | 0.0085 | 0.7883 |
| GO:0070934 | CRD-mediated mRNA stabilization | 5 | enriched | 1 | *DHX9* | 0.0399 | 0.9785 |
| GO:0071226 | cellular response to molecule of fungal origin | 4 | enriched | 1 | *SCIMP* | 0.0227 | 0.9785 |
| GO:0071287 | cellular response to manganese ion | 9 | enriched | 3 | *A3GALT2, ATP13A2, TH* | 0.0030 | 0.7162 |
| GO:0071312 | cellular response to alkaloid | 8 | enriched | 3 | *BCL2L1, HTR1B, TH* | 0.0437 | 0.9785 |
| GO:0071344 | diphosphate metabolic process | 2 | enriched | 1 | *PPA1* | 0.0064 | 0.7417 |
| GO:0071360 | cellular response to exogenous dsRNA | 17 | enriched | 3 | *DHX9, IRF3, MUL1* | 0.0004 | 0.3125 |
| GO:0071423 | malate transmembrane transport | 2 | enriched | 1 | *SLC25A11* | 0.0056 | 0.7417 |
| GO:0071447 | cellular response to hydroperoxide | 7 | enriched | 3 | *CD36, OXR1, TP53INP1* | 0.0251 | 0.9785 |
| GO:0071472 | cellular response to salt stress | 3 | enriched | 1 | *CAPN3* | 0.0103 | 0.7883 |
| GO:0071569 | protein ufmylation | 5 | enriched | 1 | *UFL1* | 0.0280 | 0.9785 |
| GO:0071596 | ubiquitin-dependent protein catabolic process via the N-end rule pathway | 3 | enriched | 1 | *UBR3* | 0.0139 | 0.9210 |
| GO:0071599 | otic vesicle development | 6 | enriched | 2 | *AHI1, WRB* | 0.0049 | 0.7417 |
| GO:0071650 | negative regulation of chemokine (C-C motif) ligand 5 production | 4 | enriched | 1 | *MUL1* | 0.0159 | 0.9513 |
| GO:0071654 | positive regulation of chemokine (C-C motif) ligand 1 production | 1 | enriched | 1 | *TSLP* | 0.0322 | 0.9785 |
| GO:0071673 | positive regulation of smooth muscle cell chemotaxis | 4 | enriched | 1 | *MDK* | 0.0149 | 0.9327 |
| GO:0071816 | tail-anchored membrane protein insertion into ER membrane | 6 | enriched | 2 | *GET4, WRB* | 0.0430 | 0.9785 |
| GO:0071888 | macrophage apoptotic process | 2 | enriched | 1 | *IRF3* | 0.0054 | 0.7417 |
| GO:0072095 | regulation of branch elongation involved in ureteric bud branching | 3 | enriched | 1 | *SIX4* | 0.0095 | 0.7883 |
| GO:0072107 | positive regulation of ureteric bud formation | 4 | enriched | 1 | *SIX4* | 0.0176 | 0.9622 |
| GO:0072197 | ureter morphogenesis | 6 | enriched | 1 | *EMX2* | 0.0482 | 0.9785 |
| GO:0072385 | minus-end-directed organelle transport along microtubule | 4 | enriched | 2 | *BICD2, RAB6A* | 0.0001 | 0.2757 |
| GO:0072393 | microtubule anchoring at microtubule organizing center | 2 | enriched | 1 | *BICD2* | 0.0064 | 0.7417 |
| GO:0072657 | protein localization to membrane | 19 | enriched | 3 | *CAPN3, RAB7L1, STX1A* | 0.0396 | 0.9785 |
| GO:0072673 | lamellipodium morphogenesis | 5 | enriched | 1 | *WASF2* | 0.0447 | 0.9785 |
| GO:0086097 | phospholipase C-activating angiotensin-activated signaling pathway | 2 | enriched | 1 | *ACTN2* | 0.0447 | 0.9785 |
| GO:0090164 | asymmetric Golgi ribbon formation | 2 | enriched | 1 | *MYO18A* | 0.0173 | 0.9622 |
| GO:0090166 | Golgi disassembly | 6 | enriched | 1 | *GBF1* | 0.0485 | 0.9785 |
| GO:0090201 | negative regulation of release of cytochrome c from mitochondria | 19 | enriched | 2 | *BCL2L1, TRIAP1* | 0.0286 | 0.9785 |
| GO:0090216 | positive regulation of 1-phosphatidylinositol-4-phosphate 5-kinase activity | 1 | enriched | 1 | *DGKZ* | 0.0331 | 0.9785 |
| GO:0090218 | positive regulation of lipid kinase activity | 2 | enriched | 1 | *F2* | 0.0396 | 0.9785 |
| GO:0090315 | negative regulation of protein targeting to membrane | 6 | enriched | 1 | *ITGB1BP1* | 0.0416 | 0.9785 |
| GO:0097022 | lymphocyte migration into lymph node | 2 | enriched | 1 | *WNK1* | 0.0184 | 0.9727 |
| GO:0097035 | regulation of membrane lipid distribution | 5 | enriched | 1 | *TRIAP1* | 0.0137 | 0.9210 |
| GO:0097084 | vascular smooth muscle cell development | 7 | enriched | 1 | *SGCB* | 0.0461 | 0.9785 |
| GO:0097111 | endoplasmic reticulum-Golgi intermediate compartment organization | 2 | enriched | 1 | *GBF1* | 0.0090 | 0.7883 |
| GO:0097198 | histone H3-K36 trimethylation | 2 | enriched | 1 | *SETD2* | 0.0064 | 0.7417 |
| GO:0097202 | activation of cysteine-type endopeptidase activity | 14 | enriched | 2 | *BAK1, IFI16* | 0.0430 | 0.9785 |
| GO:0097250 | mitochondrial respirasome assembly | 3 | enriched | 1 | *COX7A2L* | 0.0092 | 0.7883 |
| GO:0097284 | hepatocyte apoptotic process | 11 | enriched | 3 | *BCL2L1, KRT18, KRT8* | 0.0004 | 0.3036 |
| GO:0097300 | programmed necrotic cell death | 5 | enriched | 1 | *IRF3* | 0.0493 | 0.9785 |
| GO:0097676 | histone H3-K36 dimethylation | 4 | enriched | 1 | *SETD2* | 0.0187 | 0.9785 |
| GO:0097681 | double-strand break repair via alternative nonhomologous end joining | 3 | enriched | 1 | *POLQ* | 0.0113 | 0.8401 |
| GO:0098528 | skeletal muscle fiber differentiation | 2 | enriched | 1 | *SIX4* | 0.0044 | 0.7417 |
| GO:0098655 | cation transmembrane transport | 28 | enriched | 4 | *ATP13A2, CHRNE, CNGB3, SLC41A3* | 0.0453 | 0.9785 |
| GO:0098780 | response to mitochondrial depolarisation | 6 | enriched | 2 | *AMBRA1, ATG13* | 0.0002 | 0.2757 |
| GO:0098880 | maintenance of postsynaptic specialization structure | 2 | enriched | 1 | *SYNGAP1* | 0.0064 | 0.7417 |
| GO:0098883 | synapse pruning | 8 | enriched | 3 | *C1QA, C1QB, C1QC* | 0.0436 | 0.9785 |
| GO:0098885 | modification of postsynaptic actin cytoskeleton | 5 | enriched | 2 | *CTNNA2, PFN1* | 0.0207 | 0.9785 |
| GO:0106015 | negative regulation of inflammatory response to wounding | 2 | enriched | 1 | *MDK* | 0.0032 | 0.7162 |
| GO:0106016 | positive regulation of inflammatory response to wounding | 2 | enriched | 1 | *MDK* | 0.0033 | 0.7162 |
| GO:0106091 | glial cell projection elongation | 2 | enriched | 1 | *MDK* | 0.0042 | 0.7417 |
| GO:0140014 | mitotic nuclear division | 3 | enriched | 1 | *USP16* | 0.0099 | 0.7883 |
| GO:1900025 | negative regulation of substrate adhesion-dependent cell spreading | 13 | enriched | 4 | *EFNA5, ITGB1BP1, KANK1, RCC2* | 0.0054 | 0.7417 |
| GO:1900103 | positive regulation of endoplasmic reticulum unfolded protein response | 5 | enriched | 2 | *BAK1, PIK3R1* | 0.0001 | 0.2757 |
| GO:1901028 | regulation of mitochondrial outer membrane permeabilization involved in apoptotic signaling pathway | 2 | enriched | 1 | *MUL1* | 0.0032 | 0.7162 |
| GO:1901214 | regulation of neuron death | 17 | enriched | 3 | *RAB7L1, RRAS2, TSC1* | 0.0489 | 0.9785 |
| GO:1901666 | positive regulation of NAD+ ADP-ribosyltransferase activity | 3 | enriched | 1 | *HMGN1* | 0.0087 | 0.7883 |
| GO:1901842 | negative regulation of high voltage-gated calcium channel activity | 5 | enriched | 1 | *REM1* | 0.0255 | 0.9785 |
| GO:1902202 | regulation of hepatocyte growth factor receptor signaling pathway | 2 | enriched | 1 | *PTPN1* | 0.0172 | 0.9622 |
| GO:1902236 | negative regulation of endoplasmic reticulum stress-induced intrinsic apoptotic signaling pathway | 19 | enriched | 2 | *BCL2L1, PTPN1* | 0.0471 | 0.9785 |
| GO:1902254 | negative regulation of intrinsic apoptotic signaling pathway by p53 class mediator | 5 | depleted | 0 |  | 0.0449 | 0.9785 |
| GO:1902262 | apoptotic process involved in blood vessel morphogenesis | 4 | enriched | 1 | *BAK1* | 0.0174 | 0.9622 |
| GO:1902510 | regulation of apoptotic DNA fragmentation | 3 | enriched | 1 | *APAF1* | 0.0312 | 0.9785 |
| GO:1902714 | negative regulation of interferon-gamma secretion | 3 | enriched | 1 | *IL36RN* | 0.0093 | 0.7883 |
| GO:1902723 | negative regulation of skeletal muscle satellite cell proliferation | 4 | enriched | 1 | *SIX5* | 0.0166 | 0.9622 |
| GO:1902725 | negative regulation of satellite cell differentiation | 4 | enriched | 1 | *SIX4* | 0.0143 | 0.9210 |
| GO:1902749 | regulation of cell cycle G2/M phase transition | 5 | enriched | 1 | *NEK10* | 0.0338 | 0.9785 |
| GO:1902751 | positive regulation of cell cycle G2/M phase transition | 3 | enriched | 1 | *DYRK3* | 0.0202 | 0.9785 |
| GO:1902775 | mitochondrial large ribosomal subunit assembly | 3 | enriched | 1 | *FASTKD2* | 0.0090 | 0.7883 |
| GO:1902850 | microtubule cytoskeleton organization involved in mitosis | 2 | enriched | 1 | *SETD2* | 0.0064 | 0.7417 |
| GO:1903008 | organelle disassembly | 2 | enriched | 2 | *DYRK3, KIF9* | 0.0056 | 0.7417 |
| GO:1903039 | positive regulation of leukocyte cell-cell adhesion | 3 | enriched | 1 | *MDK* | 0.0103 | 0.7883 |
| GO:1903409 | reactive oxygen species biosynthetic process | 2 | enriched | 1 | *GBF1* | 0.0073 | 0.7883 |
| GO:1903432 | regulation of TORC1 signaling | 5 | enriched | 1 | *DYRK3* | 0.0454 | 0.9785 |
| GO:1903441 | protein localization to ciliary membrane | 5 | enriched | 1 | *RAB7L1* | 0.0321 | 0.9785 |
| GO:1903608 | protein localization to cytoplasmic stress granule | 7 | enriched | 2 | *SSB, DHX9* | 0.0016 | 0.5962 |
| GO:1903690 | negative regulation of wound healing, spreading of epidermal cells | 4 | enriched | 1 | *CLASP2* | 0.0375 | 0.9785 |
| GO:1903755 | positive regulation of SUMO transferase activity | 1 | enriched | 1 | *UBE2I* | 0.0306 | 0.9785 |
| GO:1903830 | magnesium ion transmembrane transport | 15 | enriched | 2 | *CNNM2, SLC41A1* | 0.0217 | 0.9785 |
| GO:1903896 | positive regulation of IRE1-mediated unfolded protein response | 7 | enriched | 2 | *BAK1, PTPN1* | 0.0010 | 0.4769 |
| GO:1904030 | negative regulation of cyclin-dependent protein kinase activity | 4 | enriched | 1 | *CAMK2N1* | 0.0092 | 0.7883 |
| GO:1904036 | negative regulation of epithelial cell apoptotic process | 3 | enriched | 1 | *MDK* | 0.0051 | 0.7417 |
| GO:1904211 | membrane protein proteolysis involved in retrograde protein transport, ER to cytosol | 2 | enriched | 1 | *HM13* | 0.0064 | 0.7417 |
| GO:1904220 | regulation of serine C-palmitoyltransferase activity | 1 | enriched | 1 | *SPTSSB* | 0.0462 | 0.9785 |
| GO:1904313 | response to methamphetamine hydrochloride | 2 | enriched | 1 | *PITX3* | 0.0040 | 0.7417 |
| GO:1904515 | positive regulation of TORC2 signaling | 2 | enriched | 1 | *SIK3* | 0.0085 | 0.7883 |
| GO:1904528 | positive regulation of microtubule binding | 4 | enriched | 1 | *PPP2CB* | 0.0297 | 0.9785 |
| GO:1904659 | glucose transmembrane transport | 25 | enriched | 4 | *MFSD4, SLC2A8, SLC5A10, SLC5A9* | 0.0453 | 0.9785 |
| GO:1904714 | regulation of chaperone-mediated autophagy | 5 | enriched | 1 | *ATP13A2* | 0.0220 | 0.9785 |
| GO:1904751 | positive regulation of protein localization to nucleolus | 5 | enriched | 1 | *PINX1* | 0.0359 | 0.9785 |
| GO:1904761 | negative regulation of myofibroblast differentiation | 3 | enriched | 1 | *TP53INP1* | 0.0103 | 0.7883 |
| GO:1904893 | negative regulation of receptor signaling pathway via STAT | 1 | depleted | 0 |  | 0.0482 | 0.9785 |
| GO:1904894 | positive regulation of receptor signaling pathway via STAT | 4 | enriched | 3 | *IL7R, MGAT5, TSLP* | 0.0025 | 0.7053 |
| GO:1904925 | positive regulation of autophagy of mitochondrion in response to mitochondrial depolarization | 4 | enriched | 1 | *MUL1* | 0.0155 | 0.9431 |
| GO:1904977 | lymphatic endothelial cell migration | 1 | enriched | 1 | *LGALS8* | 0.0312 | 0.9785 |
| GO:1905037 | autophagosome organization | 3 | enriched | 1 | *ATP13A2* | 0.0098 | 0.7883 |
| GO:1905123 | regulation of glucosylceramidase activity | 2 | enriched | 1 | *ATP13A2* | 0.0087 | 0.7883 |
| GO:1905165 | regulation of lysosomal protein catabolic process | 2 | enriched | 1 | *ATP13A2* | 0.0052 | 0.7417 |
| GO:1905166 | negative regulation of lysosomal protein catabolic process | 4 | enriched | 2 | *ATP13A2, MGAT3* | 0.0322 | 0.9785 |
| GO:1905279 | regulation of retrograde transport, endosome to Golgi | 2 | enriched | 1 | *RAB7L1* | 0.0064 | 0.7417 |
| GO:1905333 | regulation of gastric motility | 3 | enriched | 1 | *TYMP* | 0.0249 | 0.9785 |
| GO:1905555 | positive regulation blood vessel branching | 3 | enriched | 1 | *MDK* | 0.0093 | 0.7883 |
| GO:1905775 | negative regulation of DNA helicase activity | 2 | enriched | 1 | *MNAT1* | 0.0130 | 0.9114 |
| GO:1990048 | anterograde neuronal dense core vesicle transport | 5 | enriched | 1 | *KIF1C* | 0.0443 | 0.9785 |
| GO:1990049 | retrograde neuronal dense core vesicle transport | 5 | enriched | 1 | *KIF1C* | 0.0483 | 0.9785 |
| GO:1990116 | ribosome-associated ubiquitin-dependent protein catabolic process | 2 | enriched | 1 | *LTN1* | 0.0378 | 0.9785 |
| GO:1990456 | mitochondrion-endoplasmic reticulum membrane tethering | 2 | enriched | 1 | *PSEN2* | 0.0064 | 0.7417 |
| GO:1990481 | mRNA pseudouridine synthesis | 5 | enriched | 2 | *PUS7, TRUB1* | 0.0082 | 0.7883 |
| GO:1990502 | dense core granule maturation | 3 | enriched | 1 | *BAIAP3* | 0.0118 | 0.8591 |
| GO:1990592 | protein K69-linked ufmylation | 5 | enriched | 1 | *UFL1* | 0.0281 | 0.9785 |
| GO:2000171 | negative regulation of dendrite development | 5 | enriched | 1 | *KIAA0319* | 0.0291 | 0.9785 |
| GO:2000297 | negative regulation of synapse maturation | 3 | enriched | 1 | *NFATC4* | 0.0208 | 0.9785 |
| GO:2000327 | positive regulation of nuclear receptor transcription coactivator activity | 3 | enriched | 1 | *CNOT6* | 0.0437 | 0.9785 |
| GO:2000373 | positive regulation of DNA topoisomerase (ATP-hydrolyzing) activity | 5 | enriched | 1 | *DHX9* | 0.0368 | 0.9785 |
| GO:2000541 | positive regulation of protein geranylgeranylation | 1 | enriched | 1 | *MUSK* | 0.0392 | 0.9785 |
| GO:2000582 | positive regulation of ATP-dependent microtubule motor activity, plus-end-directed | 5 | enriched | 1 | *DYNLL1* | 0.0317 | 0.9785 |
| GO:2000637 | positive regulation of gene silencing by miRNA | 11 | enriched | 2 | *DHX9, PUM2* | 0.0061 | 0.7417 |
| GO:2000646 | positive regulation of receptor catabolic process | 3 | enriched | 1 | *PTPN1* | 0.0223 | 0.9785 |
| GO:2000765 | regulation of cytoplasmic translation | 2 | enriched | 1 | *DHX9* | 0.0081 | 0.7883 |
| GO:2001044 | regulation of integrin-mediated signaling pathway | 4 | enriched | 1 | *ITGB1BP1* | 0.0173 | 0.9622 |
| GO:2001140 | positive regulation of phospholipid transport | 4 | enriched | 1 | *TRIAP1* | 0.0155 | 0.9431 |
| GO:2001184 | positive regulation of interleukin-12 secretion | 4 | enriched | 1 | *MDK* | 0.0160 | 0.9545 |
| GO:2001224 | positive regulation of neuron migration | 14 | enriched | 3 | *SEMA3A, FBXO31, MDK* | 0.0027 | 0.7162 |
| GO:2001235 | positive regulation of apoptotic signaling pathway | 22 | enriched | 3 | *APAF1, INCA1, NFATC4* | 0.0076 | 0.7883 |
