# Supplementary material for: Adolescent anxiety and pain problems: A joint, genome-wide investigation and pathway-based analysis
Source: PLoS One. 2023 May 5;18(5):e0285263. doi: 10.1371/journal.pone.0285263 (PMC10162554; doi:10.1371/journal.pone.0285263)
Supplement: S9 Table — (DOCX) [file pone.0285263.s009.docx]

| **S9 Table. Overlapping enriched pathways between Mean Pain and Mean Anxiety (uncorrected p-value < 0.05).** | | | | | | | | | | |
| --- | --- | --- | --- | --- | --- | --- | --- | --- | --- | --- |
| **GO set ID** | **Description** | **Pathway size (nr of genes)** | **Mean Pain** | | | | **Mean Anxiety** | | | |
|  |  |  | **Enriched genes (nr.)** | **Genes** | ***p-value*** | **FDR** | **Enriched genes (nr.)** | **Genes** | ***p-value*** | **FDR** |
| GO:0001915 | negative regulation of T cell mediated cytotoxicity | 6 | 1 | *HLA-G* | 0.0322 | 0.9830 | 2 | *IL7R, LILRB1* | 0.0219 | 0.9785 |
| GO:0002553b | histamine secretion by mast cell | 3 | 1 | *SNAP23* | 0.0068 | 0.7864 | 1 | *SNAP23* | 0.0145 | 0.9210 |
| GO:0006450a | regulation of translational fidelity | 6 | 1 | *GATC* | 0.0290 | 0.9830 | 1 | *GATC* | 0.0417 | 0.9785 |
| GO:0006744 | ubiquinone biosynthetic process | 15 | 2 | *ADCK3, COQ5* | 0.0041 | 0.7489 | 2 | *ADCK3, COQ5* | 0.0308 | 0.9785 |
| GO:0006892 | post-Golgi vesicle-mediated transport | 19 | 3 | *ARL3, SNAP23, SORCS1* | 0.0171 | 0.9830 | 3 | *GBF1, SNAP23, VAMP8* | 0.0250 | 0.9785 |
| GO:0007156b | homophilic cell adhesion via plasma membrane adhesion molecules | 162 | 31 | *ROBO2, CDH13, DCHS2, CDHR2, CNTN4, IGSF21, PCDH7, PCDHGA1, PCDHGA10, PCDHGA11, PCDHGA12, PCDHGA2, PCDHGA3, PCDHGA4, PCDHGA5, PCDHGA6, PCDHGA7, PCDHGA8, PCDHGA9, PCDHGB1, PCDHGB2, PCDHGB3, PCDHGB4, PCDHGB6, PCDHGB7, PCDHGC3, PCDHGC4, PCDHGC5, PLXNB2, PVRL2, ROBO1* | 0.0001 | 0.1541 | 28 | *ROBO2, PCDHGA1, PCDHGA2, PCDHGA3, PCDHGA4, PCDHGA5, PCDHGB1, PCDHGB2, PCDHGB3, CDH12, DCHS2, IGSF21, NEXN, PCDH1, PCDHGA10, PCDHGA11, PCDHGA12, PCDHGA6, PCDHGA7, PCDHGA8, PCDHGA9, PCDHGB4, PCDHGB6, PCDHGB7, PCDHGC3, PCDHGC4, PCDHGC5, TENM3* | 0.0007 | 0.4102 |
| GO:0008594b | photoreceptor cell morphogenesis | 3 | 1 | *C8orf37* | 0.0048 | 0.7864 | 1 | *C8orf37* | 0.0194 | 0.9785 |
| GO:0008630 | intrinsic apoptotic signaling pathway in response to DNA damage | 45 | 3 | *NFATC4, PIK3R1, SFN* | 0.0452 | 0.9830 | 6 | *BAK1, BCL2L1, CHEK2, MOAP1, NFATC4, PIK3R1* | 0.0108 | 0.8151 |
| GO:0010569b | regulation of double-strand break repair via homologous recombination | 18 | 3 | *SETD2, TEX15, ZNF365* | 0.0149 | 0.9830 | 3 | *ZNF365, SETD2, TEX15* | 0.0195 | 0.9785 |
| GO:0010768b | negative regulation of transcription from RNA polymerase II promoter in response to UV-induced DNA damage | 2 | 1 | *NEDD4* | 0.0154 | 0.9830 | 1 | *NEDD4* | 0.0163 | 0.9605 |
| GO:0010830a,b | regulation of myotube differentiation | 5 | 2 | *DMPK, HDAC5* | 0.0003 | 0.3133 | 2 | *DMPK, HDAC5* | 0.0002 | 0.2757 |
| GO:0015711b | organic anion transport | 14 | 4 | *SLC22A10, SLC22A24, SLC22A25, SLC22A9* | 0.0060 | 0.7864 | 4 | *SLC22A10, SLC22A24, SLC22A25, SLC22A9* | 0.0168 | 0.9622 |
| GO:0030003b | cellular cation homeostasis | 2 | 1 | *ATP13A2* | 0.0030 | 0.7489 | 1 | *ATP13A2* | 0.0053 | 0.7417 |
| GO:0030330a | DNA damage response, signal transduction by p53 class mediator | 16 | 2 | *MYO6, TRIAP1* | 0.0266 | 0.9830 | 2 | *MYO6, TRIAP1* | 0.0341 | 0.9785 |
| GO:0031048b | chromatin silencing by small RNA | 3 | 1 | *FAM172A* | 0.0377 | 0.9830 | 1 | *ZNFX1* | 0.0092 | 0.7883 |
| GO:0031052b | chromosome breakage | 2 | 1 | *RFWD3* | 0.0040 | 0.7489 | 1 | *RFWD3* | 0.0064 | 0.7417 |
| GO:0031571b | mitotic G1 DNA damage checkpoint | 8 | 2 | *FBXO31, RFWD3* | 0.0300 | 0.9830 | 2 | *FBXO31, RFWD3* | 0.0009 | 0.4459 |
| GO:0032053b | ciliary basal body organization | 3 | 2 | *CROCC, RTTN* | 0.0017 | 0.6135 | 2 | *CROCC, RTTN* | 0.0032 | 0.7162 |
| GO:0032259 | methylation | 54 | 5 | *COMT, COQ5, CYP1A2, NDUFAF5, SETD9* | 0.0348 | 0.9830 | 7 | *AS3MT, COQ5, METTL5, NDUFAF5, PRDM8, SETD9, TPMT* | 0.0005 | 0.3125 |
| GO:0033157b | regulation of intracellular protein transport | 7 | 1 | *ATP13A2* | 0.0492 | 0.9830 | 2 | *ATP13A2, PTPN1* | 0.0018 | 0.5962 |
| GO:0034644b | cellular response to UV | 49 | 7 | *AURKB, NEDD4, NFATC4, PIK3R1, PTGS2, TMEM161A, TRIAP1* | 0.0001 | 0.1626 | 6 | *BAK1, NEDD4, NFATC4, PIK3R1, TP53INP1, TRIAP1* | 0.0001 | 0.2757 |
| GO:0034728b | nucleosome organization | 3 | 1 | *SETD2* | 0.0068 | 0.7864 | 1 | *SETD2* | 0.0142 | 0.9210 |
| GO:0035063 | nuclear speck organization | 4 | 1 | *SRPK2* | 0.0140 | 0.9830 | 1 | *DYRK3* | 0.0199 | 0.9785 |
| GO:0035441b | cell migration involved in vasculogenesis | 2 | 1 | *SETD2* | 0.0040 | 0.7489 | 1 | *SETD2* | 0.0064 | 0.7417 |
| GO:0038018a | Wnt receptor catabolic process | 2 | 1 | *ZNRF3* | 0.0115 | 0.9160 | 1 | *ZNRF3* | 0.0192 | 0.9785 |
| GO:0040020b | regulation of meiotic nuclear division | 5 | 2 | *GPR3, PDE3A* | 0.0144 | 0.9830 | 2 | *GPR3, PDE3A* | 0.0203 | 0.9785 |
| GO:0042270 | protection from natural killer cell mediated cytotoxicity | 5 | 1 | *HLA-G* | 0.0217 | 0.9830 | 1 | *SERPINB4* | 0.0165 | 0.9622 |
| GO:0042921b | glucocorticoid receptor signaling pathway | 5 | 2 | *ARID1A, NEDD4* | <0.0001 | 0.1523 | 1 | *NEDD4* | 0.0442 | 0.9785 |
| GO:0043279a | response to alkaloid | 6 | 1 | *SRSF9* | 0.0294 | 0.9830 | 2 | *BCHE, SRSF9* | 0.0002 | 0.2757 |
| GO:0043666a | regulation of phosphoprotein phosphatase activity | 41 | 7 | *PPP2R1A, DMPK, PPP1R3B, PPP2R1B, PPP2R2B, PPP2R5A, PPP6R2* | 0.0015 | 0.6081 | 7 | *RCAN1, DMPK, PPP1R3B, PPP1R7, PPP2R2B, PPP2R2D, TSC1* | 0.0172 | 0.9622 |
| GO:0045333 | cellular respiration | 17 | 4 | *CYP1A2, NDUFS1, NDUFS4, NFATC4* | <0.0001 | 0.1186 | 3 | *COX4I2, NDUFS1, NFATC4* | 0.0122 | 0.8811 |
| GO:0046626 | regulation of insulin receptor signaling pathway | 12 | 3 | *CCND3, PIK3R1, SIK2* | 0.0024 | 0.6957 | 2 | *NUCKS1, PIK3R1* | 0.0078 | 0.7883 |
| GO:0048050b | post-embryonic eye morphogenesis | 3 | 1 | *MFAP2* | 0.0058 | 0.7864 | 1 | *MFAP2* | 0.0103 | 0.7883 |
| GO:0048332b | mesoderm morphogenesis | 2 | 1 | *SETD2* | 0.0040 | 0.7489 | 1 | *SETD2* | 0.0064 | 0.7417 |
| GO:0048864b | stem cell development | 5 | 1 | *SETD2* | 0.0173 | 0.9830 | 1 | *SETD2* | 0.0292 | 0.9785 |
| GO:0050774b | negative regulation of dendrite morphogenesis | 9 | 2 | *GORASP1, NFATC4* | 0.0009 | 0.4811 | 2 | *ID1, NFATC4* | 0.0004 | 0.3036 |
| GO:0050808b | synapse organization | 46 | 10 | *GRM5, PPFIA2, C1QA, CTNND2, LRRTM2, PCDHGC3, PCDHGC4, PCDHGC5, PPFIBP2, SNCB* | <0.0001 | 0.0023 | 8 | *C1QA, CTNND2, PCDHGC3, PCDHGC4, PCDHGC5, PPFIA4, TSC1, WRB* | 0.0014 | 0.5962 |
| GO:0051046a | regulation of secretion | 2 | 1 | *MYO6* | 0.0072 | 0.7962 | 1 | *MYO6* | 0.0115 | 0.8500 |
| GO:0051823a | regulation of synapse structural plasticity | 5 | 2 | *CAMK2B, DMPK* | 0.0118 | 0.9251 | 3 | *CAMK2B, CTNNA2, DMPK* | 0.0151 | 0.9329 |
| GO:0051895a | negative regulation of focal adhesion assembly | 18 | 4 | *APOD, DLC1, RCC2, THBS1* | 0.0102 | 0.9124 | 4 | *CLASP2, ITGB1BP1, MMP14, RCC2* | 0.0003 | 0.2757 |
| GO:0051987b | positive regulation of attachment of spindle microtubules to kinetochore | 3 | 1 | *RCC2* | 0.0057 | 0.7864 | 1 | *RCC2* | 0.0129 | 0.9114 |
| GO:0052746b | inositol phosphorylation | 2 | 1 | *IPPK* | 0.0040 | 0.7489 | 1 | *IPPK* | 0.0064 | 0.7417 |
| GO:0055001b | muscle cell development | 4 | 1 | *NFATC4* | 0.0169 | 0.9830 | 1 | *NFATC4* | 0.0459 | 0.9785 |
| GO:0055065 | metal ion homeostasis | 3 | 1 | *CNNM4* | 0.0463 | 0.9830 | 1 | *COX11* | 0.0211 | 0.9785 |
| GO:0055069b | zinc ion homeostasis | 5 | 2 | *ATP13A2, PARK2* | 0.0132 | 0.9726 | 1 | *ATP13A2* | 0.0215 | 0.9785 |
| GO:0060669b | embryonic placenta morphogenesis | 6 | 1 | *SETD2* | 0.0297 | 0.9830 | 2 | *SETD2, ZNF568* | 0.0197 | 0.9785 |
| GO:0060836a | lymphatic endothelial cell differentiation | 6 | 1 | *ACVR2B* | 0.0296 | 0.9830 | 1 | *ACVR2B* | 0.0474 | 0.9785 |
| GO:0060841a | venous blood vessel development | 4 | 1 | *ACVR2B* | 0.0111 | 0.9124 | 1 | *ACVR2B* | 0.0186 | 0.9785 |
| GO:0060977b | coronary vasculature morphogenesis | 3 | 2 | *SGCD, SETD2* | 0.0023 | 0.6945 | 2 | *SETD2, SGCD* | 0.0060 | 0.7417 |
| GO:0061088b | regulation of sequestering of zinc ion | 5 | 1 | *SLC30A2* | 0.0202 | 0.9830 | 1 | *SLC30A2* | 0.0342 | 0.9785 |
| GO:0070681a | glutaminyl-tRNAGln biosynthesis via transamidation | 3 | 1 | *GATC* | 0.0074 | 0.8082 | 1 | *GATC* | 0.0103 | 0.7883 |
| GO:0071344b | diphosphate metabolic process | 2 | 1 | *PPA1* | 0.0040 | 0.7489 | 1 | *PPA1* | 0.0064 | 0.7417 |
| GO:0072385b | minus-end-directed organelle transport along microtubule | 4 | 1 | *BICD2* | 0.0126 | 0.9540 | 2 | *BICD2, RAB6A* | 0.0001 | 0.2757 |
| GO:0072393b | microtubule anchoring at microtubule organizing center | 2 | 1 | *BICD2* | 0.0041 | 0.7489 | 1 | *BICD2* | 0.0064 | 0.7417 |
| GO:0072673b | lamellipodium morphogenesis | 5 | 1 | *WASF2* | 0.0219 | 0.9830 | 1 | *WASF2* | 0.0447 | 0.9785 |
| GO:0097198b | histone H3-K36 trimethylation | 2 | 1 | *SETD2* | 0.0040 | 0.7489 | 1 | *SETD2* | 0.0064 | 0.7417 |
| GO:0097676b | histone H3-K36 dimethylation | 4 | 1 | *SETD2* | 0.0106 | 0.9124 | 1 | *SETD2* | 0.0187 | 0.9785 |
| GO:0098883b | synapse pruning | 8 | 3 | *C1QA, C1QB, C1QC* | 0.0368 | 0.9830 | 3 | *C1QA, C1QB, C1QC* | 0.0436 | 0.9785 |
| GO:1900025b | negative regulation of substrate adhesion-dependent cell spreading | 13 | 3 | *ACTN4, KANK1, RCC2* | 0.0129 | 0.9668 | 4 | *EFNA5, ITGB1BP1, KANK1, RCC2* | 0.0054 | 0.7417 |
| GO:1900103b | positive regulation of endoplasmic reticulum unfolded protein response | 5 | 1 | *PIK3R1* | 0.0189 | 0.9830 | 2 | *BAK1, PIK3R1* | 0.0001 | 0.2757 |
| GO:1902723a | negative regulation of skeletal muscle satellite cell proliferation | 4 | 1 | *SIX5* | 0.0136 | 0.9830 | 1 | *SIX5* | 0.0166 | 0.9622 |
| GO:1902775b | mitochondrial large ribosomal subunit assembly | 3 | 1 | *FASTKD2* | 0.0062 | 0.7864 | 1 | *FASTKD2* | 0.0090 | 0.7883 |
| GO:1902850b | microtubule cytoskeleton organization involved in mitosis | 2 | 1 | *SETD2* | 0.0040 | 0.7489 | 1 | *SETD2* | 0.0064 | 0.7417 |
| GO:1903008b | organelle disassembly | 2 | 1 | *KIF9* | 0.0318 | 0.9830 | 2 | *DYRK3, KIF9* | 0.0056 | 0.7417 |
| GO:1903441a | protein localization to ciliary membrane | 5 | 1 | *ARL3* | 0.0190 | 0.9830 | 1 | *RAB7L1* | 0.0321 | 0.9785 |
| GO:1904714b | regulation of chaperone-mediated autophagy | 5 | 1 | *ATP13A2* | 0.0087 | 0.8491 | 1 | *ATP13A2* | 0.0220 | 0.9785 |
| GO:1904751b | positive regulation of protein localization to nucleolus | 5 | 1 | *PINX1* | 0.0213 | 0.9830 | 1 | *PINX1* | 0.0359 | 0.9785 |
| GO:1905037b | autophagosome organization | 3 | 1 | *ATP13A2* | 0.0061 | 0.7864 | 1 | *ATP13A2* | 0.0098 | 0.7883 |
| GO:1905123b | regulation of glucosylceramidase activity | 2 | 1 | *ATP13A2* | 0.0040 | 0.7489 | 1 | *ATP13A2* | 0.0087 | 0.7883 |
| GO:1905165b | regulation of lysosomal protein catabolic process | 2 | 1 | *ATP13A2* | 0.0041 | 0.7489 | 1 | *ATP13A2* | 0.0052 | 0.7417 |
| GO:1905166b | negative regulation of lysosomal protein catabolic process | 4 | 1 | *ATP13A2* | 0.0067 | 0.7864 | 2 | *ATP13A2, MGAT3* | 0.0322 | 0.9785 |
| GO:1990116 | ribosome-associated ubiquitin-dependent protein catabolic process | 2 | 1 | *LTN1* | 0.0061 | 0.7864 | 1 | *LTN1* | 0.0378 | 0.9785 |
| GO:2000297b | negative regulation of synapse maturation | 3 | 1 | *NFATC4* | 0.0077 | 0.8255 | 1 | *NFATC4* | 0.0208 | 0.9785 |
| GO:2000582a | positive regulation of ATP-dependent microtubule motor activity, plus-end-directed | 5 | 1 | *DYNLL1* | 0.0184 | 0.9830 | 1 | *DYNLL1* | 0.0317 | 0.9785 |
| GO:2001140a | positive regulation of phospholipid transport | 4 | 1 | *TRIAP1* | 0.0292 | 0.9830 | 1 | *TRIAP1* | 0.0155 | 0.9431 |
| GO:2001184a | positive regulation of interleukin-12 secretion | 4 | 1 | *MAPK11* | 0.0112 | 0.9124 | 1 | *MDK* | 0.0160 | 0.9545 |
| a Replication of overlapping enriched pathways between QNTS_Mean Pain and QNTS_Mean Anxiety (uncorrected p-value < 0.05). | | | | | | | | | | |
| b Replication of overlapping enriched pathways between QLSCD_Mean Pain and QLSCD_Mean Anxiety (uncorrected p-value < 0.05). | | | | | | | | | | |
