# Supplementary figures and images for: Adolescent anxiety and pain problems: A joint, genome-wide investigation and pathway-based analysis
Source: PLoS One. 2023 May 5;18(5):e0285263. doi: 10.1371/journal.pone.0285263 (PMC10162554; doi:10.1371/journal.pone.0285263)

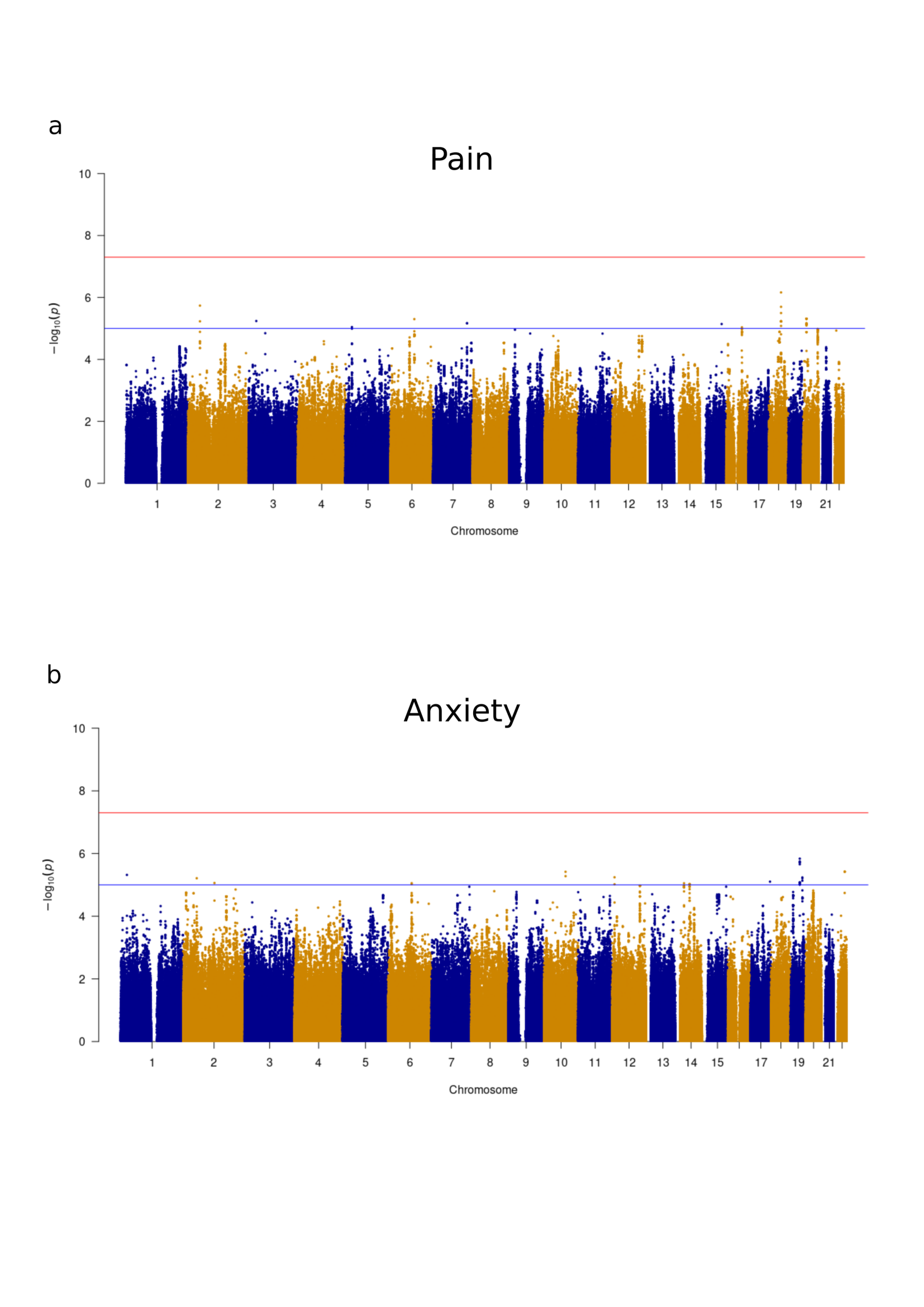

Supplement: S1 Fig — (TIF) [file pone.0285263.s010.tif]

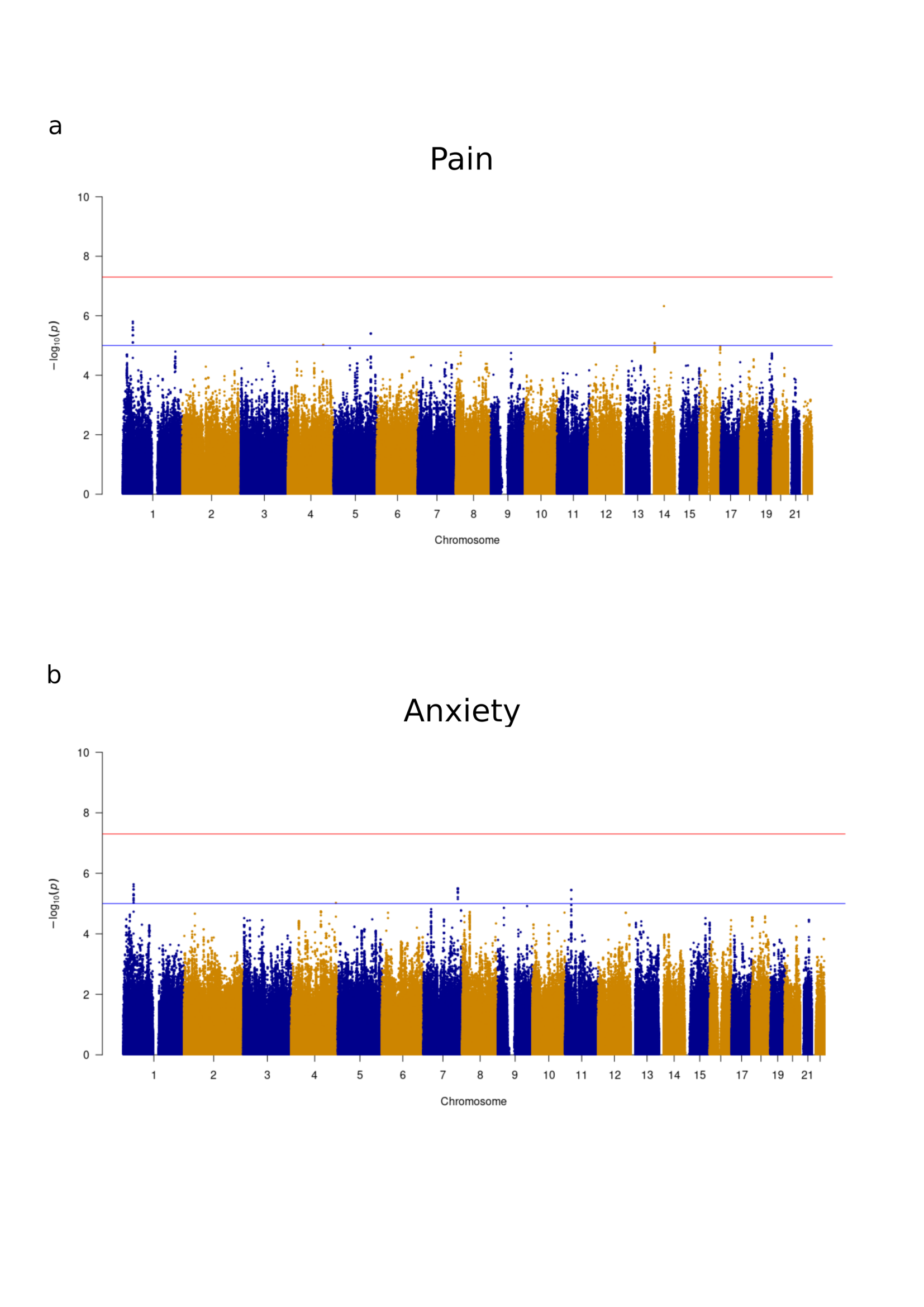

Supplement: S2 Fig — (TIF) [file pone.0285263.s011.tif]
